# Correction: The structural landscape and diversity of *Pyricularia oryzae* MAX effectors revisited

**DOI:** 10.1371/journal.ppat.1014532

**Published:** 2026-08-20

**Authors:** Mounia Lahfa, Philippe Barthe, Karine de Guillen, Stella Cesari, Mouna Raji, Thomas Kroj, Marie Le Naour—Vernet, François Hoh, Pierre Gladieux, Christian Roumestand, Jérôme Gracy, Nathalie Declerck, André Padilla

In Fig 5, the position of SS1 is incorrect. Please see the correct Fig 5 here.

**Fig 5:**
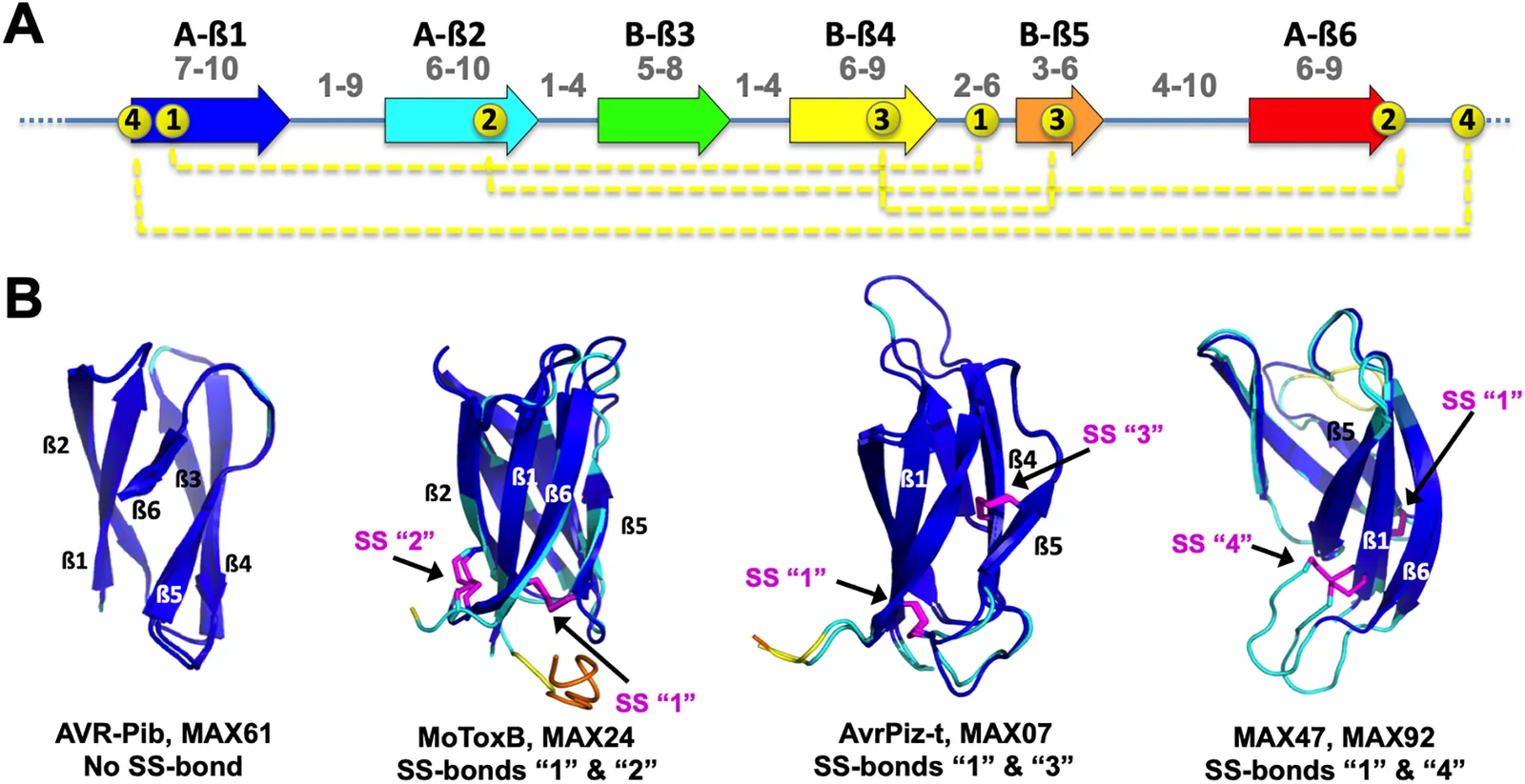
MAX domain structural features. (A) Average size range (indicated in grey) of ß strands (arrows) and connecting loops forming the central ß sandwich of the canonical MAX core, and of the N-ter and C-ter extensions. Average ranges are calculated in S7b Table. The 4 different types of disulfide bonds observed in MAX structures and models are indicated by dotted yellow lines. (B) Four different sets of structural models illustrating the variability of disulfide bond patterns. The AF2 models are colored according to their pLDDT score, by blue for high accuracy (>90), cyan for backbone at good accuracy (> 70), yellow for low confidence (> 50 and < 70) and orange for disordered (< 50). The disulfide bonds are shown in magenta, except for the AVR-Pib structure, which does not have a disulfide bond.
