# Supplementary material for: The structural landscape and diversity of Pyricularia oryzae MAX effectors revisited
Source: PLoS Pathog. 2024 May 6;20(5):e1012176. doi: 10.1371/journal.ppat.1012176 (PMC11132498; doi:10.1371/journal.ppat.1012176)
Supplement: S2 Fig — (PDF) [file ppat.1012176.s002.pdf]

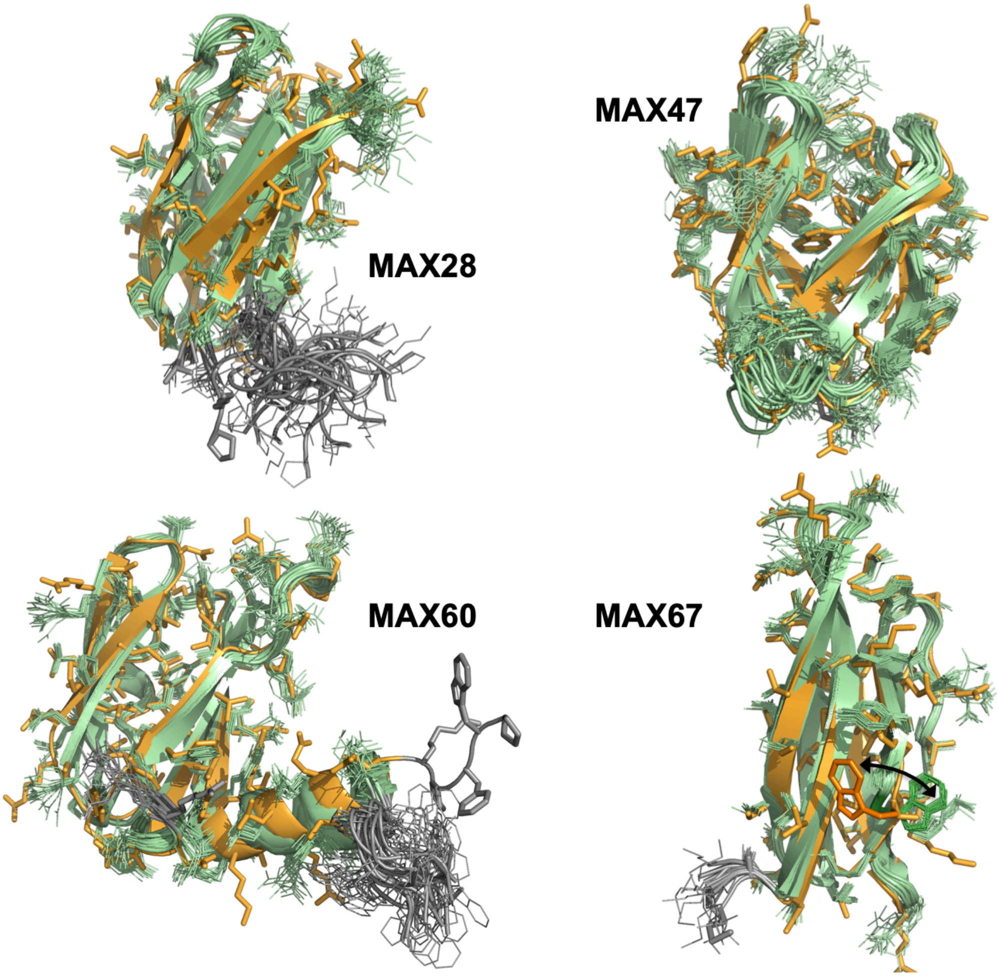

S2 Fig. NMR structures and AF models

Superimposition of the 20 conformers of the NMR structure (green) and AF model (orange). Side-chains are shown by lines and sticks. The black arrow shows the different orientation of the C-terminal W77 in the AF model and in the NMR structure of MAX67.
