# Supplementary material for: The structural landscape and diversity of Pyricularia oryzae MAX effectors revisited
Source: PLoS Pathog. 2024 May 6;20(5):e1012176. doi: 10.1371/journal.ppat.1012176 (PMC11132498; doi:10.1371/journal.ppat.1012176)
Supplement: S4 Fig — (PDF) [file ppat.1012176.s004.pdf]

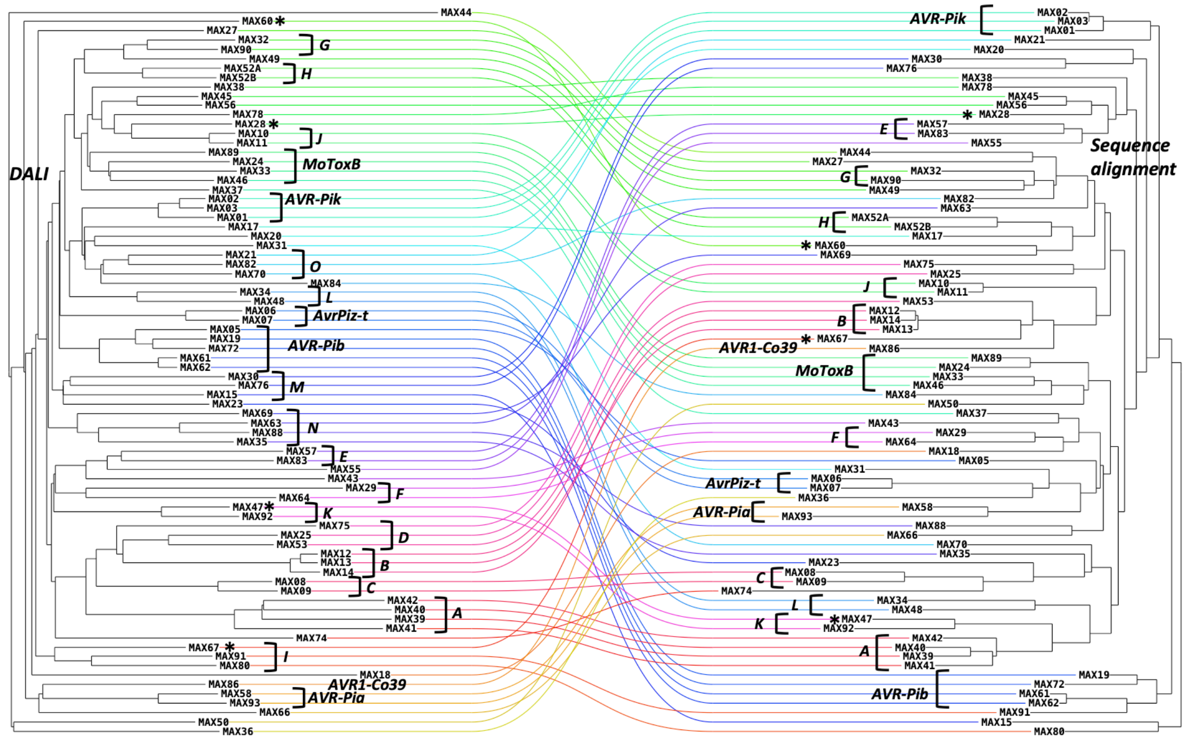

S4 Fig. Comparison of the similarity trees of MAX effectors based on the Dali Z-score (left) and sequence alignment (right) of the AF MAX models. *A line of a specific color connects each AF_MAX model in the Dali and the FastME trees. Sequences were aligned using MAFFT. The MAX effectors with an experimental 3D structure are indicated by their name, or a star for the four novel MAX structures*
