# Supplementary material for: The structural landscape and diversity of Pyricularia oryzae MAX effectors revisited
Source: PLoS Pathog. 2024 May 6;20(5):e1012176. doi: 10.1371/journal.ppat.1012176 (PMC11132498; doi:10.1371/journal.ppat.1012176)
Supplement: S5 Fig — (PDF) [file ppat.1012176.s005.pdf]

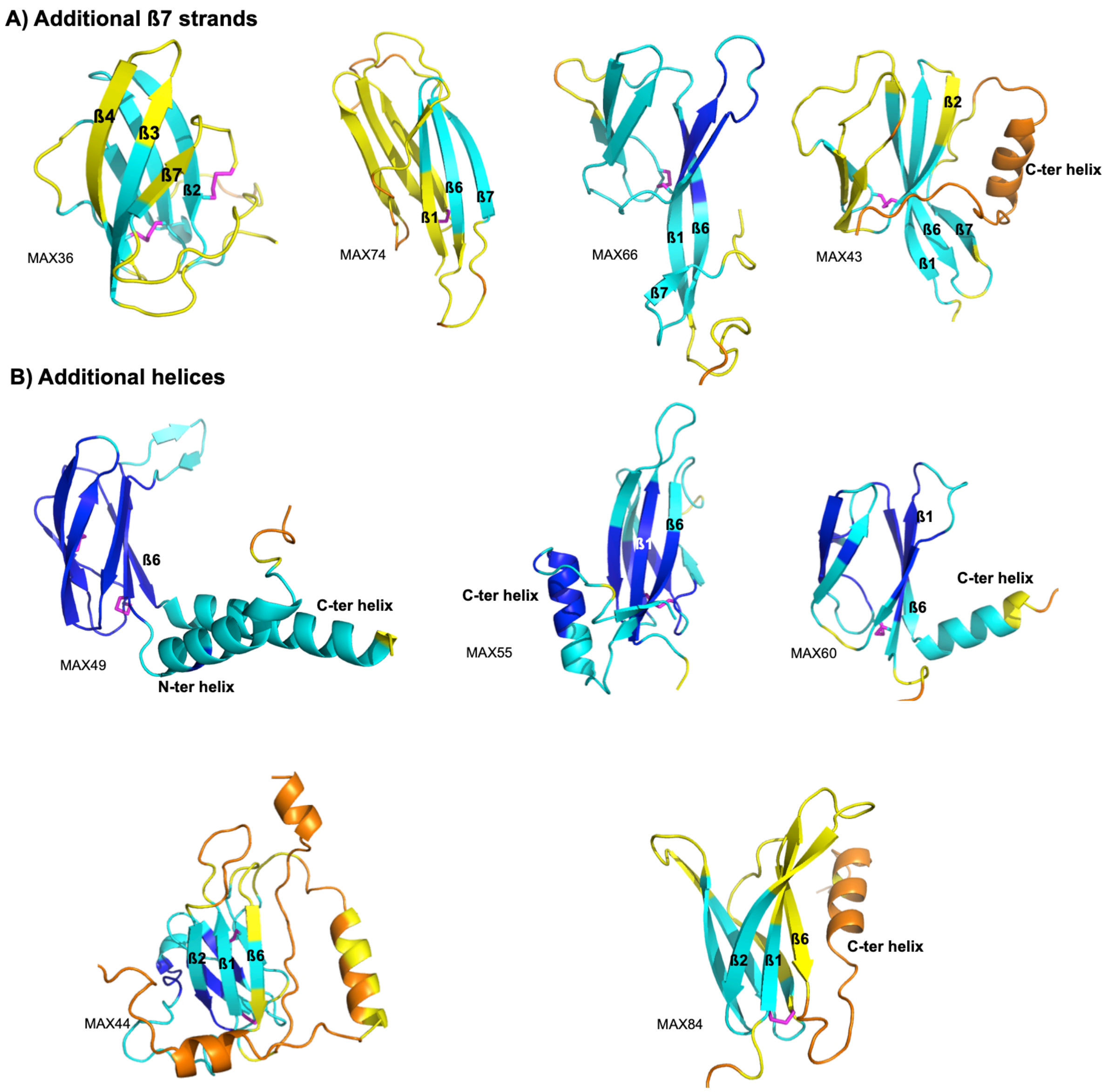

S5 Fig. Singleton MAX effectors with structured extensions

A) MAX effectors with ß7 additional strand. For MAX36 the ß7 strand runs parallel to ß3 and the C-terminal cysteine residue makes a disulfide bond with a cysteine residue in ß2.

B) MAX effectors with additional helices. In the case of MAX49 the N- and C-terminal helices are packed against each other. C-terminal helices of MAX55 and MAX60 are well defined in the AlphaFold models while they have low pLDDT scores in MAX44 and MAX84 models.
