# Supplementary material for: The structural landscape and diversity of Pyricularia oryzae MAX effectors revisited
Source: PLoS Pathog. 2024 May 6;20(5):e1012176. doi: 10.1371/journal.ppat.1012176 (PMC11132498; doi:10.1371/journal.ppat.1012176)
Supplement: S7 Fig — (PDF) [file ppat.1012176.s007.pdf]

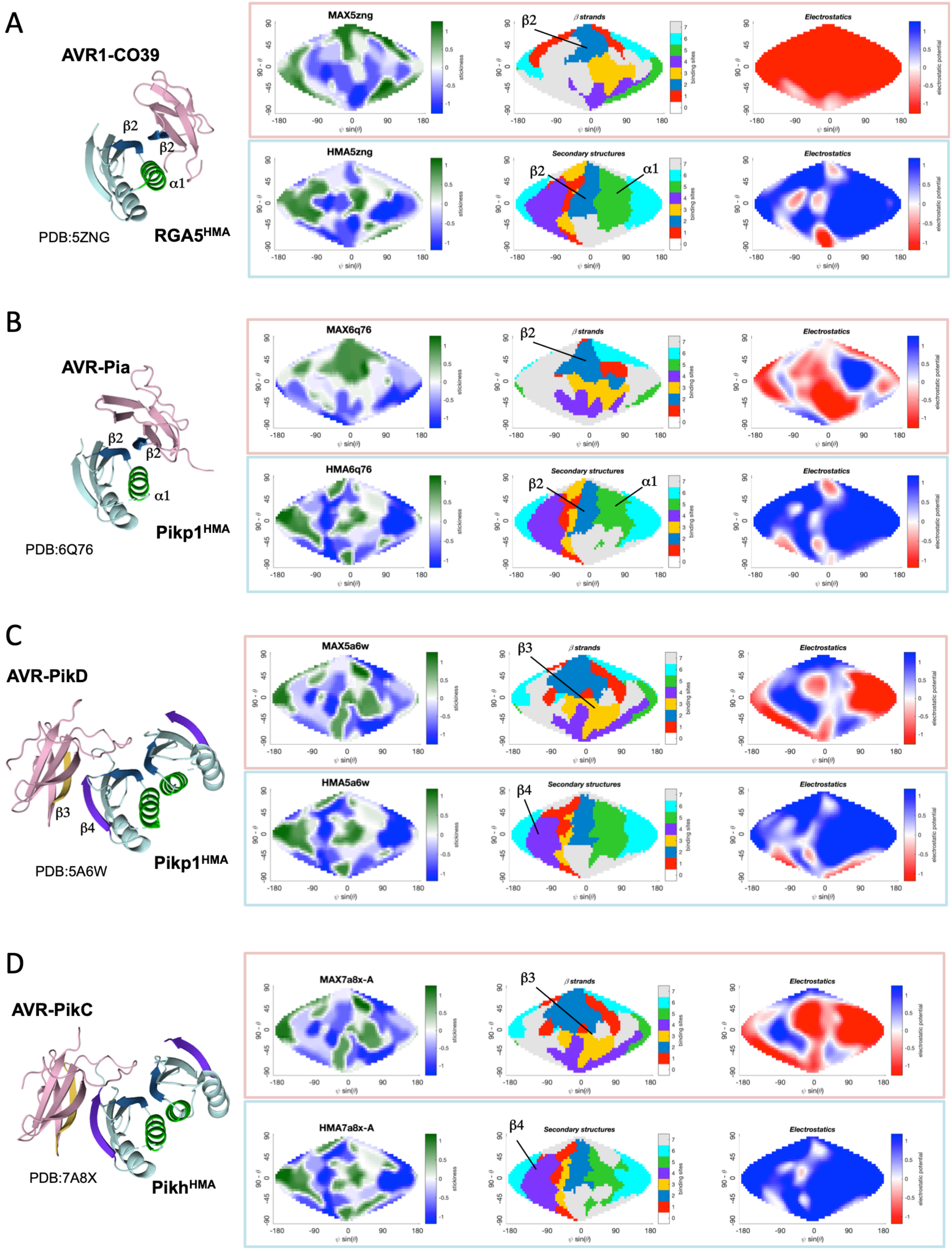

A
AVR1-CO39
β2
β2
α1
PDB:5ZNG
RGA5HMA
MAX5zng
β strands
Electrostatics
HMA5zng
Secondary structures
β2
α1
stickiness
binding sites
electrostatic potential
90 - θ
ψ sin(θ)
B
AVR-Pia
β2
β2
α1
PDB:6Q76
Pikp1HMA
MAX6q76
HMA6q76
C
AVR-PikD
β3
β4
PDB:5A6W
Pikp1HMA
MAX5a6w
HMA5a6w
D
AVR-PikC
PDB:7A8X
PikhHMA
MAX7a8x-A
HMA7a8x-A

E

5ZNG

AVR1-CO39

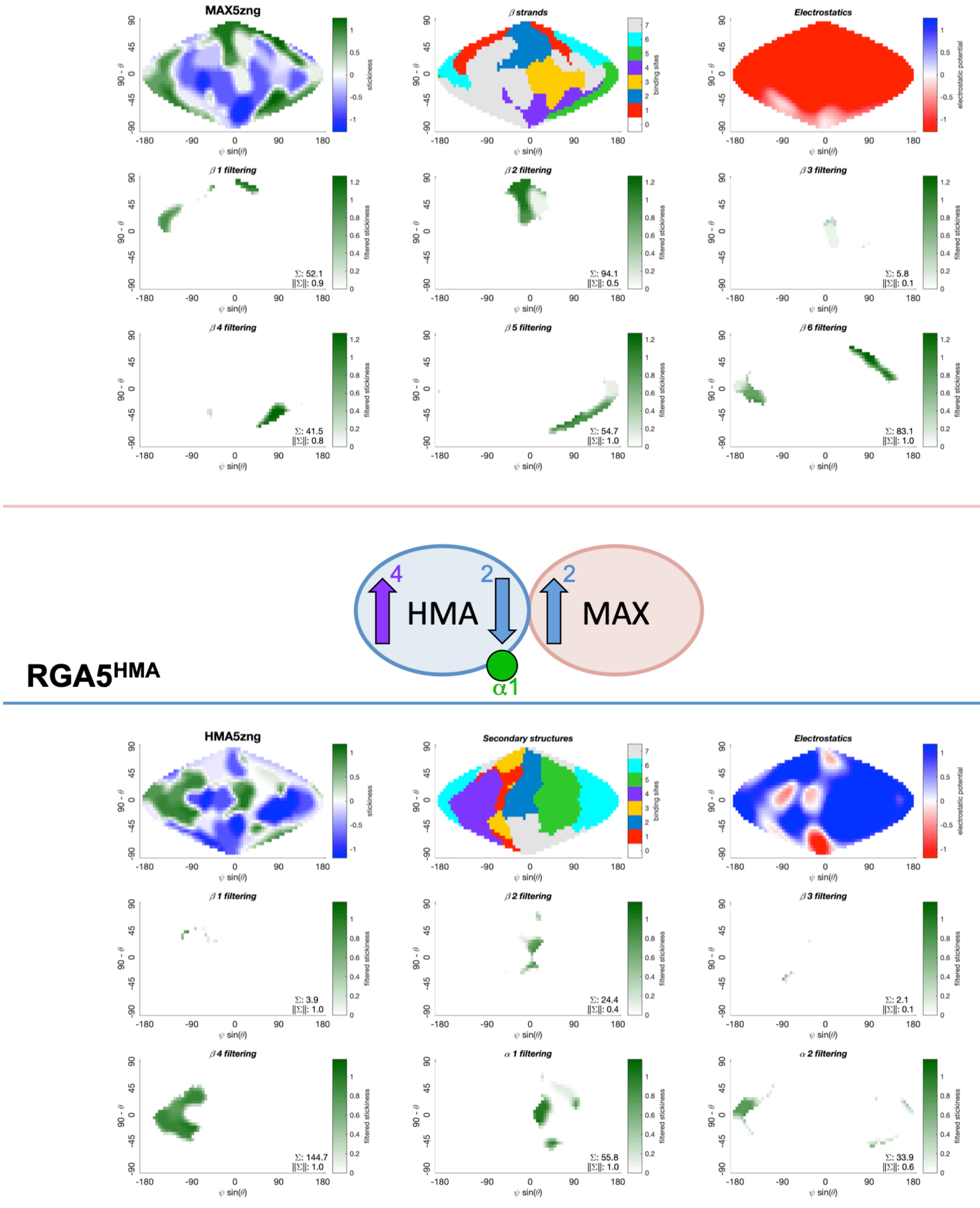

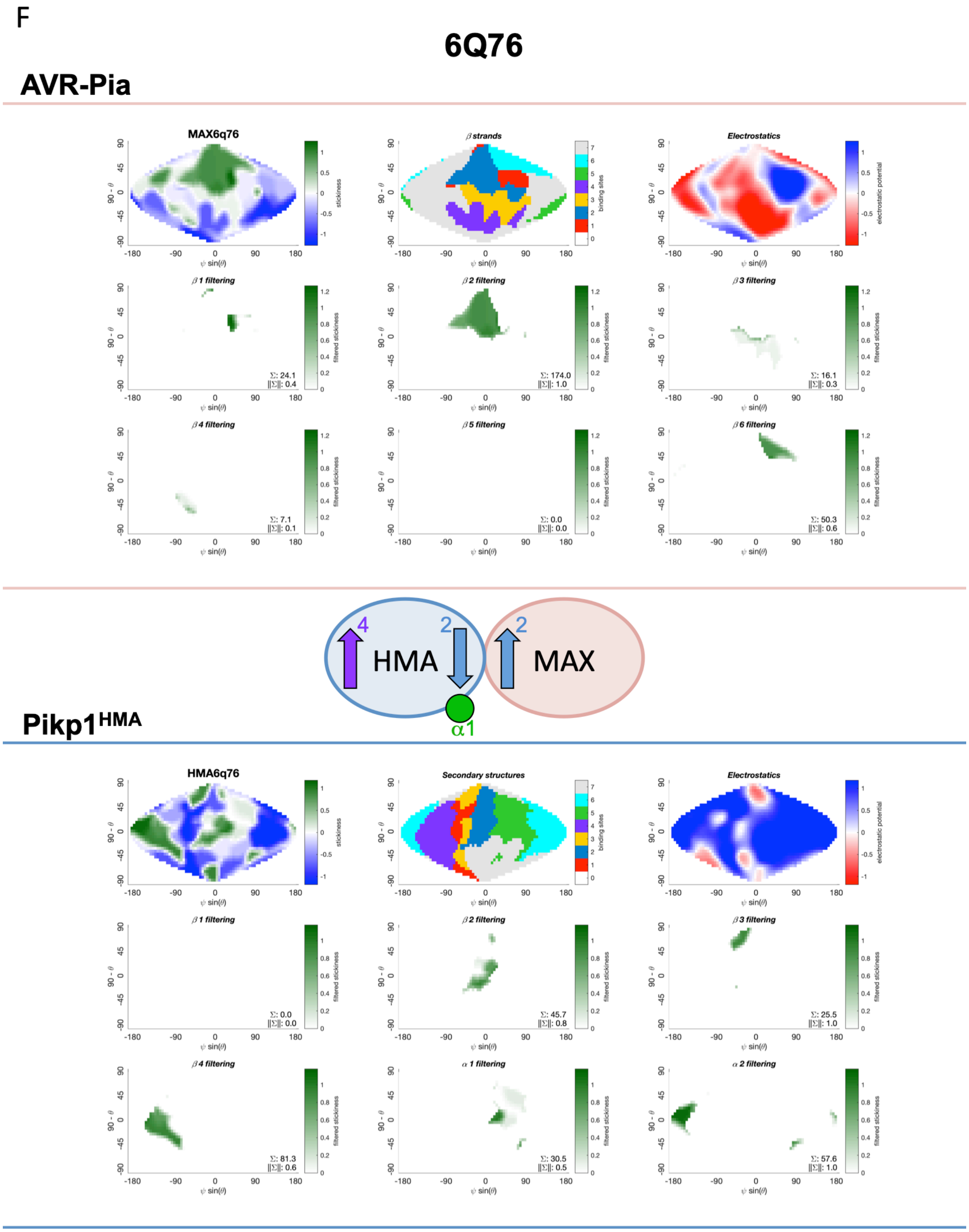

F
6Q76
AVR-Pia
MAX6q76
β strands
Electrostatics
β1 filtering
Σ: 24.1
||Σ||: 0.4
β2 filtering
Σ: 174.0
||Σ||: 1.0
β3 filtering
Σ: 16.1
||Σ||: 0.3
β4 filtering
Σ: 7.1
||Σ||: 0.1
β5 filtering
Σ: 0.0
||Σ||: 0.0
β6 filtering
Σ: 50.3
||Σ||: 0.6
HMA
MAX
4
2
2
α1
Pikp1HMA
HMA6q76
Secondary structures
Electrostatics
β1 filtering
Σ: 0.0
||Σ||: 0.0
β2 filtering
Σ: 45.7
||Σ||: 0.8
β3 filtering
Σ: 25.5
||Σ||: 1.0
β4 filtering
Σ: 81.3
||Σ||: 0.6
α1 filtering
Σ: 30.5
||Σ||: 0.5
α2 filtering
Σ: 57.6
||Σ||: 1.0
stickiness
binding sites
electrostatic potential
filtered stickiness
ψ sin(θ)
90 - θ

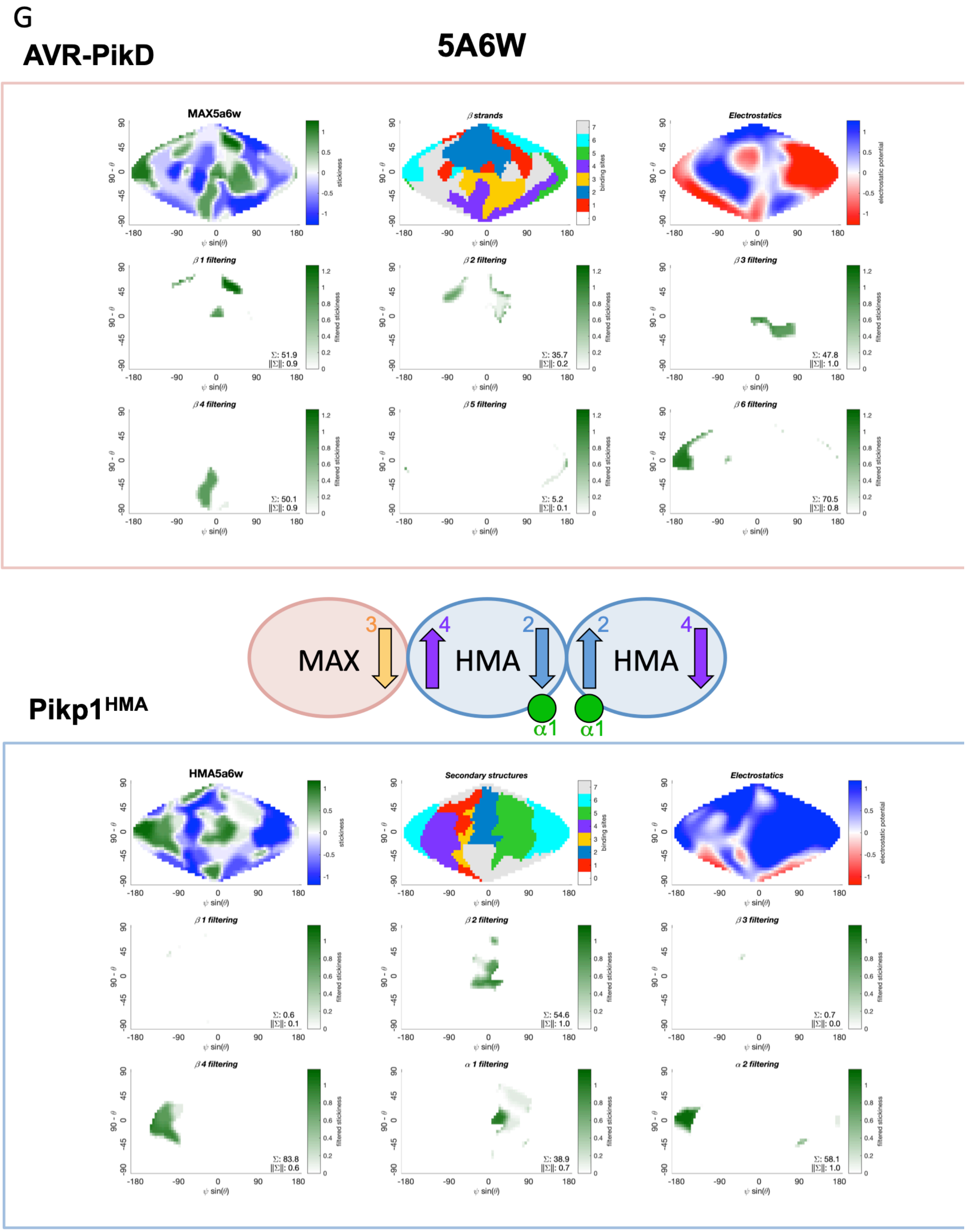
G
AVR-PikD
5A6W
MAX5a6w
β strands
Electrostatics
β1 filtering
β2 filtering
β3 filtering
β4 filtering
β5 filtering
β6 filtering
Σ: 51.9
||Σ||: 0.9
Σ: 35.7
||Σ||: 0.2
Σ: 47.8
||Σ||: 1.0
Σ: 50.1
||Σ||: 0.9
Σ: 5.2
||Σ||: 0.1
Σ: 70.5
||Σ||: 0.8
stickiness
binding sites
electrostatic potential
filtered stickiness
90 - θ
ψ sin(θ)
MAX
HMA
HMA
3
4
2
2
4
α1
α1
Pikp1HMA
HMA5a6w
Secondary structures
Electrostatics
β1 filtering
β2 filtering
β3 filtering
β4 filtering
α1 filtering
α2 filtering
Σ: 0.6
||Σ||: 0.1
Σ: 54.6
||Σ||: 1.0
Σ: 0.7
||Σ||: 0.0
Σ: 83.8
||Σ||: 0.6
Σ: 38.9
||Σ||: 0.7
Σ: 58.1
||Σ||: 1.0

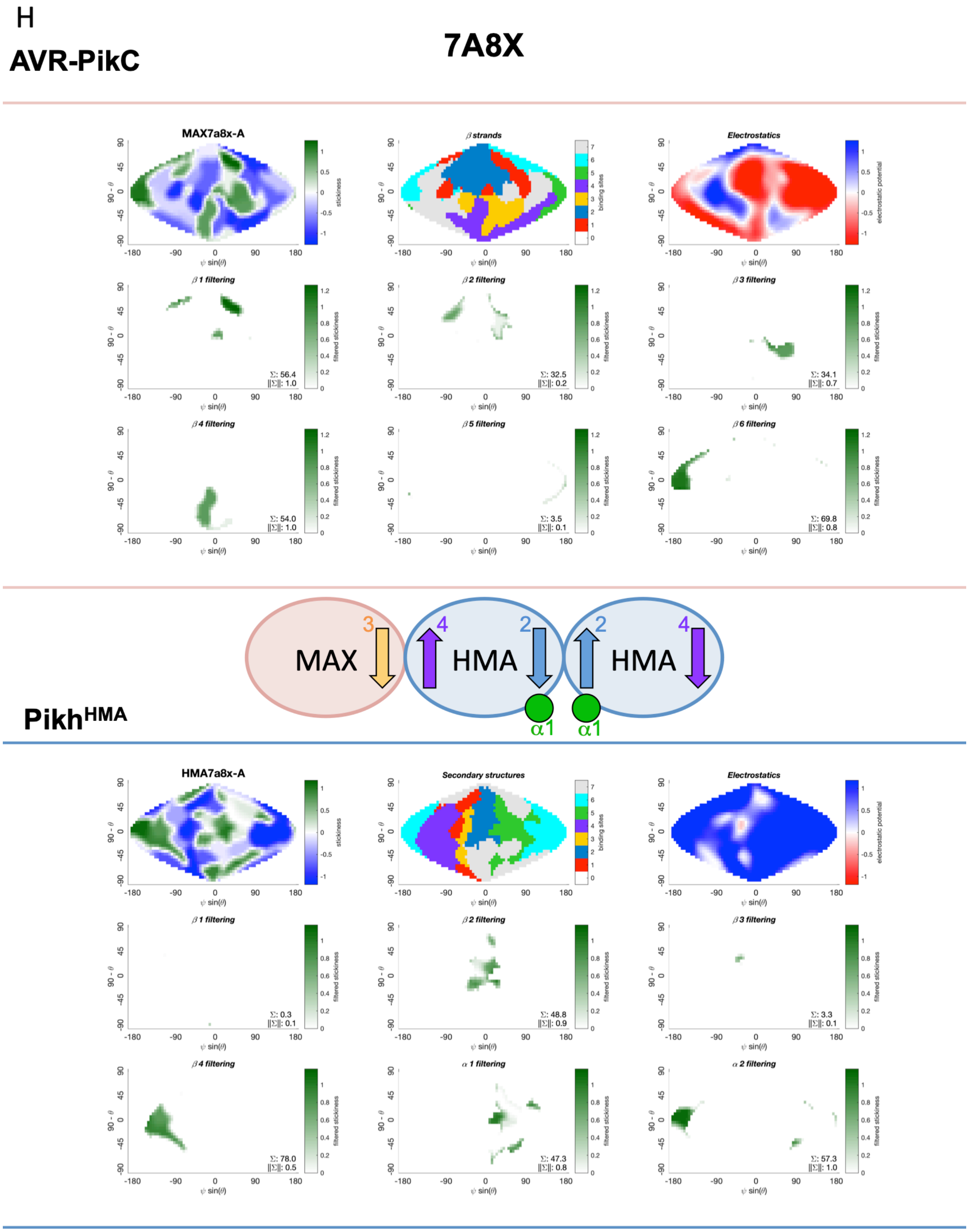
H
AVR-PikC
7A8X
MAX7a8x-A
β strands
Electrostatics
β1 filtering
β2 filtering
β3 filtering
β4 filtering
β5 filtering
β6 filtering
Σ: 56.4
||Σ||: 1.0
Σ: 32.5
||Σ||: 0.2
Σ: 34.1
||Σ||: 0.7
Σ: 54.0
||Σ||: 1.0
Σ: 3.5
||Σ||: 0.1
Σ: 69.8
||Σ||: 0.8
MAX
HMA
HMA
3
4
2
2
4
α1
α1
Pikh^HMA
HMA7a8x-A
Secondary structures
Electrostatics
β1 filtering
β2 filtering
β3 filtering
β4 filtering
α1 filtering
α2 filtering
Σ: 0.3
||Σ||: 0.1
Σ: 48.8
||Σ||: 0.9
Σ: 3.3
||Σ||: 0.1
Σ: 78.0
||Σ||: 0.5
Σ: 47.3
||Σ||: 0.8
Σ: 57.3
||Σ||: 1.0
90 - θ
ψ sin(θ)
stickiness
binding sites
electrostatic potential
filtered stickiness

S7 Fig. Comparison of crystal structures and interaction surfaces of MAX-HMA complexes.

Cartoon representation using Pymol of MAX effectors (in light pink) and Heavy Metal Associated (HMA) domains (in pale cyan) observed in the complex structure of A) Avr1-CO39/RGA5^{HMA} (PDB 5ZNG), B) Avr-Pia/Pikp-1^{HMA} (PDB 6Q76), C) Avr-PikD/Pikp-1^{HMA} (PDB 5A6W) and D) Avr-PikC/Pikh^{HMA} (PDB 7A8X) are shown next to the 2D maps of the surface properties (stickiness (left panels), secondary structure (central panels) and electrostatic potential (right panels)) calculated using SURFMAP of the MAX core (upper panels) and HMA domains (lower panels), as detailed in Fig 8. Secondary structures predominating at the MAX/HMA interface are indicated in the structures and colored according to the color code used in the 2D maps (central panels) where β-strand 1, 2, 3, 4, 5 and 6 of the MAX core, or β-strand 1, 2, 3, 4, α-helix 1 and 2 of the HMA domain are highlighted in red, dark cyan, yellow, purple, green and bright cyan, respectively. E), F), G), H) 2D surface maps as shown above in A), B), C), D), respectively, and additional panels showing stickiness maps filtered for each secondary structural element.
