# Supplementary material for: The structural landscape and diversity of Pyricularia oryzae MAX effectors revisited
Source: PLoS Pathog. 2024 May 6;20(5):e1012176. doi: 10.1371/journal.ppat.1012176 (PMC11132498; doi:10.1371/journal.ppat.1012176)
Supplement: S8 Fig — (PDF) [file ppat.1012176.s008.pdf]

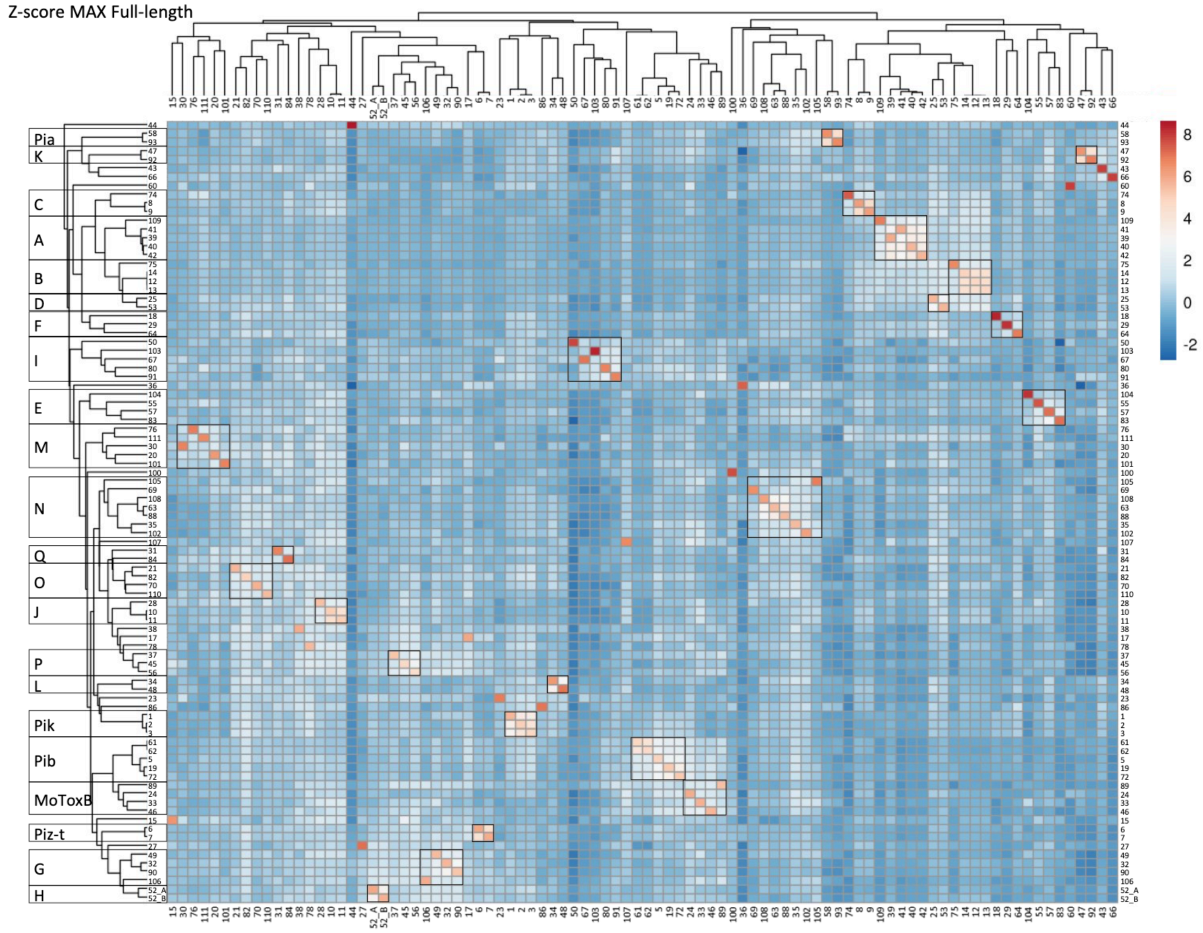

S8 Fig. Dali Z-score clustering Heatmap

All the 89 MAX AF2 models were used including the 12 additional AF2 models (MAX100 to MAX111) reported in S8a Table. They were submitted to an all-to-all Dali analysis and the Z-score matrix was introduced in Clustvis web tool [1]. The heatmap was computed with default options, including row centering, unit variance scaling applied to rows, and clustering using correlation distance and average linkage applied to both rows and columns. MAX groups are reported on the left and labeled as in Fig 7. Additional groups P and Q are indicated.

REFERENCES

1. Metsalu T, Vilo J. ClustVis: a web tool for visualizing clustering of multivariate data using Principal Component Analysis and heatmap. Nucleic Acids Res. 2015;43: W566–W570. doi:10.1093/nar/gkv468
