# Supplementary material for: The structural landscape and diversity of Pyricularia oryzae MAX effectors revisited
Source: PLoS Pathog. 2024 May 6;20(5):e1012176. doi: 10.1371/journal.ppat.1012176 (PMC11132498; doi:10.1371/journal.ppat.1012176)
Supplement: S4 Table — (PDF) [file ppat.1012176.s012.pdf]

S4a Table: Refinement statistics of MAX effector NMR structures

	MAX47	MAX60	MAX67	MAX28
**NMR distance and dihedral constraints**				
Distance constraints				
Total NOE	1379	2115	1389	1269
Intra-residue	337	475	246	221
Inter-residue				
Sequential ($\|i-j\|$ = 1)	413	556	376	350
Medium-range ($\|i-j\|$ < 4)	124	393	164	159
Long-range ($\|i-j\|$ > 5)	505	691	603	539
Hydrogen bonds	56	64	64	48
Disulfide bonds restraints	6	3	3	3
Total dihedral angle restraints				
ϕ	55	64	50	0
ψ	55	64	50	0
χ_1	21	43	23	0
**Structure statistics**				
Violations (mean and s.d.)				
Max. distance constraint violation (Å)	0.16 ± 0.03	0.16 ± 0.02	0.12 ± 0.02	0.22 ± 0.04
Max. dihedral angle violation (º)	2.04 ± 0.90	2.38 ± 0.76	1.35 ± 0.42	n. d.
Deviations from idealized geometry				
Bond lengths (Å)	0.0116 ± 0.0005	0.0117 ± 0.0003	0.0116 ± 0.0003	0.0118 ± 0.0003
Bond angles (º)	1.1135 ± 0.0316	1.2043 ± 0.0305	1.1140 ± 0.0287	1.1340 ± 0.0354
Impropers (º)	1.3765 ± 0.0865	1.3153 ± 0.0618	1.2634 ± 0.0786	1.4213 ± 0.0804
**Ramachandran plot (%)**				
Most favoured region	82.8	86.1	89.5	79.0
Additionally allowed region	16.2	13.5	10.5	20.4
Generously allowed region	0.6	0.4	0.0	0.3
Disallowed region	0.4	0.0	0.0	0.3
**Average pairwise** r.m.s.**d (Å)** (1)				
Backbone atoms of 20 NMR conformers	0.59 ± 0.12	0.42 ± 0.10	0.30 ± 0.08	0.51 ± 0.11
Heavy atoms of 20 NMR conformers	1.31 ± 0.19	0.95 ± 0.12	0.87 ± 0.13	0.94 ± 0.13

(1) Average pairwise root mean square deviation (r.m.s.d) between backbone atoms of the 20 best refined NMR conformers calculated for residues 42-100 (MAX47), 29-102 (MAX60), 22-76 (MAX67) and 38-99 (MAX28).

S4b Table: AF model and NMR structure superimposition

MAX28: AlphaFold model *versus* 20 NMR conformers

**Average pairwise *r.m.s.* deviation** (Å)**	
Backbone	1.42 ± 0.05
Heavy	2.84 ± 0.04

** " Pairwise r.m.s.d. calculated among 20 refined structures for residues 38-99 against the AlphaFold model."

MAX47: AlphaFold model *versus* 20 NMR conformers

**Average pairwise *r.m.s.* deviation** (Å)**	
Backbone	1.35 ± 0.11
Heavy	2.17 ± 0.10

** " Pairwise r.m.s.d. calculated among 20 refined structures for residues 42-100 against the AlphaFold model."

MAX60: AlphaFold model *versus* 20 NMR conformers

**Average pairwise *r.m.s.* deviation** (Å)**	
Backbone	1.11 ± 0.07
Heavy	2.06 ± 0.07

** " Pairwise r.m.s.d. calculated among 20 refined structures for residues 29-102 against the AlphaFold model."

MAX67: AlphaFold model *versus* 20 NMR conformers

**Average pairwise *r.m.s.* deviation** (Å)**	
Backbone	0.99 ± 0.04
Heavy	2.07 ± 0.04

** " Pairwise r.m.s.d. calculated among 20 refined structures for residues 22-76 against the AlphaFold model."
