# Supplementary material for: The structural landscape and diversity of Pyricularia oryzae MAX effectors revisited
Source: PLoS Pathog. 2024 May 6;20(5):e1012176. doi: 10.1371/journal.ppat.1012176 (PMC11132498; doi:10.1371/journal.ppat.1012176)
Supplement: S1 Supplementary Methods — (PDF) [file ppat.1012176.s017.pdf]

S1 Supplementary Methods

Crystallographic structure determination of MoToxB

The structure was determined at 1,38 Å resolution by molecular replacement.

Protein expression and purification. The sequence for the mature protein (residues 21-90 for MoToxB, UniProt L7J9X7: identical to the *P. oryzae* gene strain Br58_090910T0 or the TH16_EuGene_00135161 gene in GEMO database [1]) was inserted into the pET-SP vector [1,2]. The plasmid pET-SP-MoToxB was used to transform E. coli BL21 (DE3) cells (Invitrogen). Bacteria were grown on auto inducing minimal media C-750501 [3] containing ampicillin 100 µg/mL. Cells were grown with shaking at 200 rpm at 30˚C for 24h. To generate isotopically labeled samples for NMR spectroscopy, we used $^{15}NH_4Cl$ as the primary nitrogen. Cells were harvested by centrifugation 5000 x g for 20 minutes using Thermo Scientific SORVALL LYNX 6000 and re-suspended in lysis buffer (200mM TrisHCl pH8, 200mM Sucrose, 0.05mM EDTA, 50µM lysozyme). After 30 minutes incubation, cell debris were removed by centrifugation at 12 000 g for 30 min at 4°C. The His6-tagged proteins were isolated from the filtrate using a 5 mL Ni-NTA HisTrap HP (Healthcare Life Sciences) with an imidazole gradient from 5mM to 500 mM over 10 column volumes (CV). Fractions containing the protein were identified by SDS-PAGE gel analysis and pooled. The protein was further purified by gel filtration using a Superdex S75 26/60 (GE Healthcare) column in buffer A (20 mM TrisHCl, pH 8.0, 150 mM NaCl, 1mM DTT) and pure fractions were pooled. The protein was cleaved using TEV protease at 35 µg/ml during an overnight dialysis bath in buffer A at 4°C. The cleaved protein recovered after further gel filtration and verification of fractions by SDS PAGE was pooled. All chromatography steps were performed using an AKTA purifier (GE Healthcare Life Sciences).

Crystallisation. The purified protein was concentrated to 10 mg/ml in 20 mM TrisHCl, pH 8.0, 150 mM NaCl, 1mM DTT. Crystallization trials were performed at 25°C using the hanging-drop vapor-diffusion method in 96 microplates and Mosquito HTS robot with 100 nl of protein mixed with 100 nl of reservoir. After 3 weeks, crystals were obtained from condition 0,1M Hepes pH 7.5, 1.6M Ammonium Sulfate,

1,6M PEG 1000. Crystals were cryo-protected with oil (Paratone, Hampton research), and freezed in liquid N_2.

X-ray Data collection, processing and structure determination.

X-ray diffraction data were collected on a Pixel detector (PILATUS3 2M) by the autonomous ESRF beamline MASSIF-1 [4] using automatic protocols for the location and optimal centering of crystals. The beam diameter was selected automatically to match the crystal volume of highest homogeneous quality. Strategy calculations accounted for flux and crystal volume in the parameter prediction for complete data sets. Data were auto-processed by XDS [5], AIMLESS [6], from the CCP4/CCP4i programs suite [7,8]. *MotoxB* crystals belong to the *C*121 space group and contain two molecules in the asymmetric unit. The structure was determined at 1,38 Å resolution by Molecular replacement using PHASER from PHENIX (Table A) and a preliminary NMR model (see below). After model building using ARP/WARP [9], COOT [10] and refinement by REFINE from PHENIX [11] the final structure had a $R(\%)/R(\%)_{free}$ ratio of 0.143 / 0.171.

Preliminary Solution Structure of MoToxB used for molecular replacement.

The ^{15}N-labeled protein sample was dialyzed against 20 mM Na-Phosphate buffer (pH 6.8), 150 mM NaCl and 1 mM DTT, overnight at 4°C. The sample was concentrated to 760 µM in 220 µL volume with 5% D_2O for the lock. NMR experiments were carried out at 10°C on a Bruker Avance III 700 MHz spectrometer, equipped with 5 mm z-gradient TCI cryoprobe. Backbone and side chains ^1H and ^{15}N resonance assignments were obtained through 3D [^1H,^{15}N] NOESY-HSQC (mixing time 150 ms) and TOCSY-HSQC (isotropic mixing: 50 ms) experiments. Water suppression was achieved with the WATERGATE sequence [12]. ^1H chemical shifts were directly referenced to the methyl resonance of DSS, while ^{15}N chemical shifts were referenced indirectly to the absolute $^{15}N/^1H$ frequency ratio. All NMR spectra were processed with Topspin 3.6 (Bruker) and analyzed with Cindy 2.1 (Padilla, www.cbs.cnrs.fr). Assignments of amide ^1H, ^{15}N chemical shifts are given in Fig A. NOE cross-peaks identified on the 3D [^1H, ^{15}N] NOESY-HSQC experiment were assigned through several runs of automated NMR structure calculations with CYANA 3 [13,14]. For the final list of restraints, distance values redundant with

covalent geometry were eliminated and two disulfide bonds that were consistent with short distances between cysteine residues Cys25-Cys66 and Cys41-Cys86 were added. A total of 200 three-dimensional structures were generated using the torsion angle dynamics protocol of CYANA 3 from the NOEs and 2 disulfide bond restraints. The best structure (based on the final target penalty function value) was used for molecular replacement.

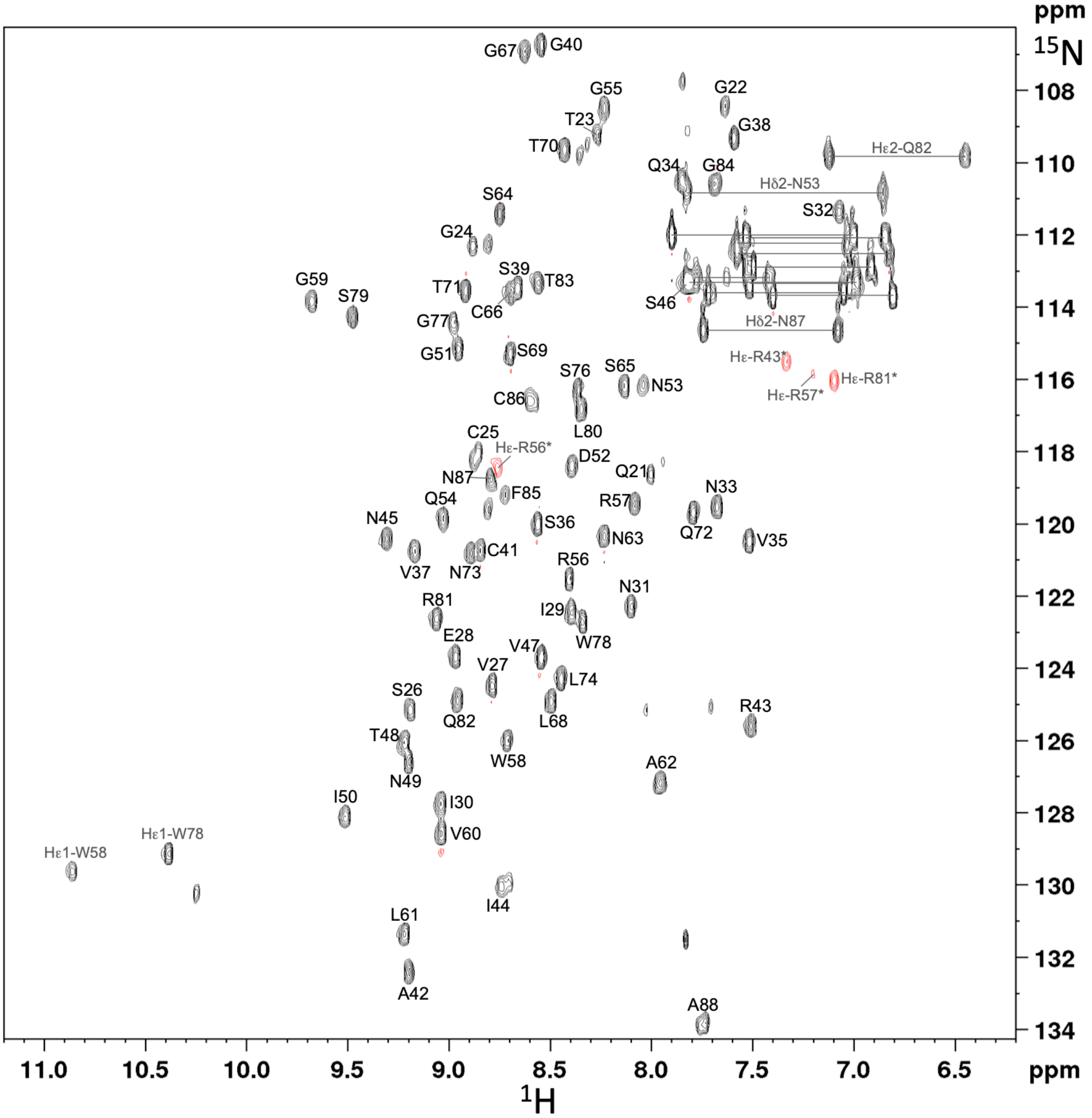

Fig A. *[^{1}H, ^{15}N] HSQC of ^{15}N-labeled MoToxB (0.76 mM) in 20 mM Na-phosphate pH 6.8, 150 mM NaCl, 1 mM DTT recorded at 283K and at 700 MHz.*

Table A. X-ray data collection and refinement statistics.

	MoToxB
Wavelength	0.996
Resolution range (Å)	34.59-1.38 (1.43-1.38)
Space group	*C*121
Unit cell	
a, b, c (Å)	73.341, 24.760, 68.613
α, β, γ (°)	90, 100.88, 90
Total reflections	59102 (2877)
Unique reflections	23545 (1686)
Multiplicity	2.5 (1.7)
Completeness (%)	93.7 (70)
Mean I/sigma(I)	14 (4,5)
Wilson B-factor (Å^2)	12
R-merge	0.038 (0.11)
$CC_{1/2}$	0.997 (0.967)
Reflections used in refinement	22356
Reflections used for R-free	1189
R-work	0.143
R-free	0.171
Number of non-hydrogen atoms	1118
macromolecules	970
Heterogen atoms	19
solvent	120
Protein residues	135
RMS(bonds Å)	0.0062
RMS(angles °)	0.98
Ramachandran favored (%)	100
Ramachandran allowed(%)	0
Ramachandran outliers (%)	0
Rotamer outliers (%)	0
Clashscore	3.06
Average B-factor (Å^2)	15.03
macromolecules	13.04
solvent	35.56

#R_{merge} = S_{hkl} S_i $|I_i(hkl)$ - $(I(hkl))|$ / S_{hkl} S_i $I_i(hkl)$, where $I_i(hkl)$ is the *i*th observation of reflection *hkl* and <*I(hkl)*> is the weighted average intensity for all observations of reflection *hkl*.

REFERENCES

1. de Guillen K, Ortiz-Vallejo D, Gracy J, Fournier E, Kroj T, Padilla A. Structure Analysis Uncovers a Highly Diverse but Structurally Conserved Effector Family in Phytopathogenic Fungi. PLOS Pathog. 2015;11: e1005228.
2. Ortiz D, de Guillen K, Cesari S, Chalvon V, Gracy J, Padilla A, et al. Recognition of the *Magnaporthe oryzae* Effector AVR-Pia by the Decoy Domain of the Rice NLR Immune Receptor RGA5. Plant Cell. 2017;29: 156–168. doi:10.1105/tpc.16.00435

3. Studier FW. Protein production by auto-induction in high-density shaking cultures. Protein Expr Purif. 2005;41: 207–234.
4. Svensson O, Gilski M, Nurizzo D, Bowler MW. Multi-position data collection and dynamic beam sizing: recent improvements to the automatic data-collection algorithms on MASSIF-1. Acta Crystallogr Sect Struct Biol. 2018;74: 433–440. doi:10.1107/S2059798318003728
5. Kabsch W. Integration, scaling, space-group assignment and post-refinement. Acta Crystallogr D Biol Crystallogr. 2010;66: 133–144. doi:10.1107/S0907444909047374
6. Evans PR. An introduction to data reduction: space-group determination, scaling and intensity statistics. Acta Crystallogr D Biol Crystallogr. 2011;67: 282–292. doi:10.1107/S090744491003982X
7. Winn MD, Ballard CC, Cowtan KD, Dodson EJ, Emsley P, Evans PR, et al. Overview of the *CCP* 4 suite and current developments. Acta Crystallogr D Biol Crystallogr. 2011;67: 235–242. doi:10.1107/S0907444910045749
8. Potterton E, Briggs P, Turkenburg M, Dodson E. A graphical user interface to the *CCP* 4 program suite. Acta Crystallogr D Biol Crystallogr. 2003;59: 1131–1137. doi:10.1107/S0907444903008126
9. Langer G, Cohen SX, Lamzin VS, Perrakis A. Automated macromolecular model building for X-ray crystallography using ARP/wARP version 7. Nat Protoc. 2008;3: 1171–1179. doi:10.1038/nprot.2008.91
10. Emsley P, Lohkamp B, Scott WG, Cowtan K. Features and development of *Coot*. Acta Crystallogr D Biol Crystallogr. 2010;66: 486–501. doi:10.1107/S0907444910007493
11. Adams PD, Afonine PV, Bunkóczi G, Chen VB, Davis IW, Echols N, et al. *PHENIX* : a comprehensive Python-based system for macromolecular structure solution. Acta Crystallogr D Biol Crystallogr. 2010;66: 213–221. doi:10.1107/S0907444909052925
12. Piotto M, Saudek V, Sklenár V. Gradient-tailored excitation for single-quantum NMR spectroscopy of aqueous solutions. J Biomol NMR. 1992;2: 661–665.
13. Güntert P. Automated NMR structure calculation with CYANA. Methods Mol Biol Clifton NJ. 2004;278: 353–378. doi:10.1385/1-59259-809-9:353
14. Güntert P, Buchner L. Combined automated NOE assignment and structure calculation with CYANA. J Biomol NMR. 2015 [cited 18 Apr 2015]. doi:10.1007/s10858-015-9924-9
