# Supplementary figures and images for: The structural landscape and diversity of Pyricularia oryzae MAX effectors revisited (part 2 of 2)
Source: PLoS Pathog. 2024 May 6;20(5):e1012176. doi: 10.1371/journal.ppat.1012176 (PMC11132498; doi:10.1371/journal.ppat.1012176)

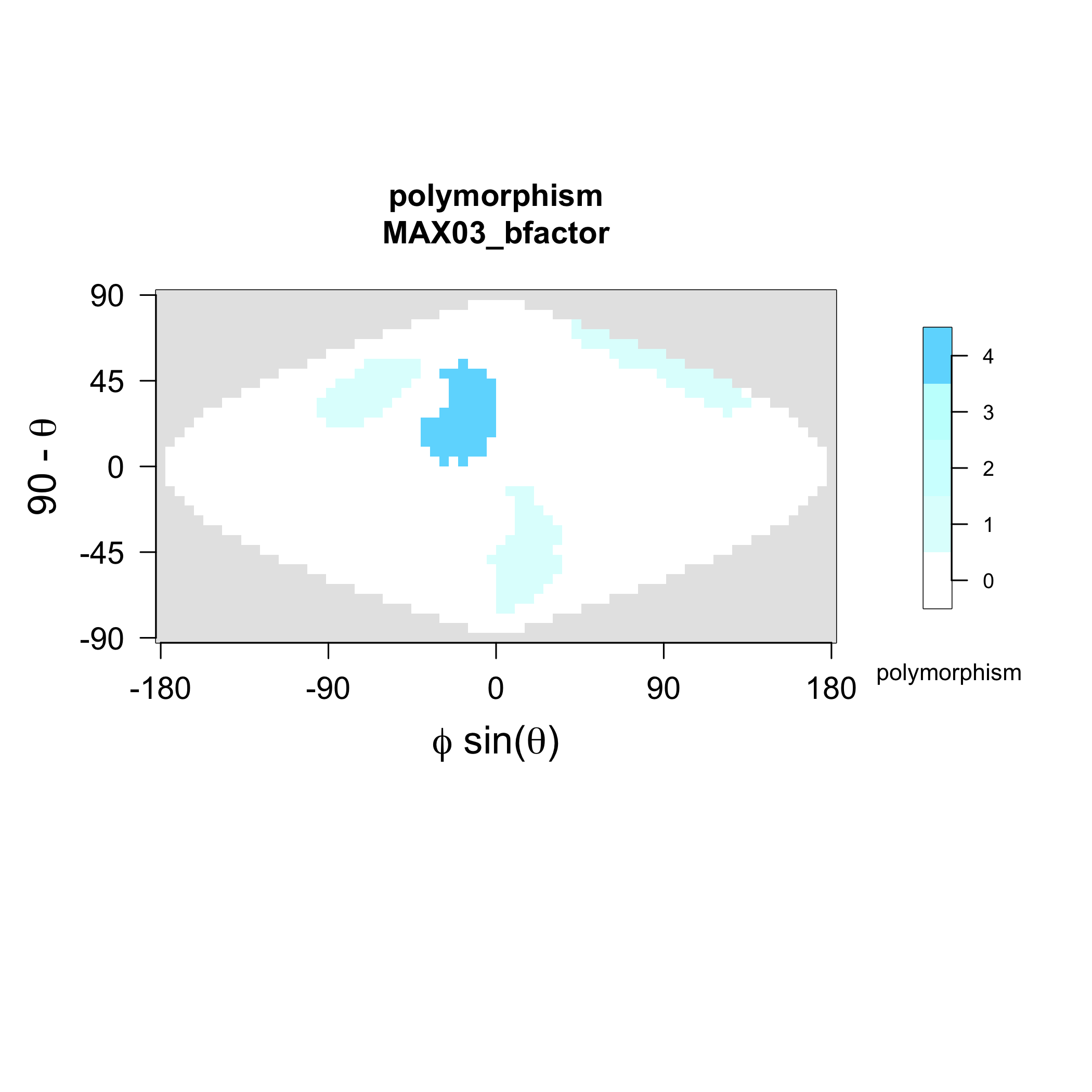

Supplement: S2 File — (ZIP) [file ppat.1012176.s019.zip › S2_File/POLYMORPHISM/MAX03_polymorphism.png]

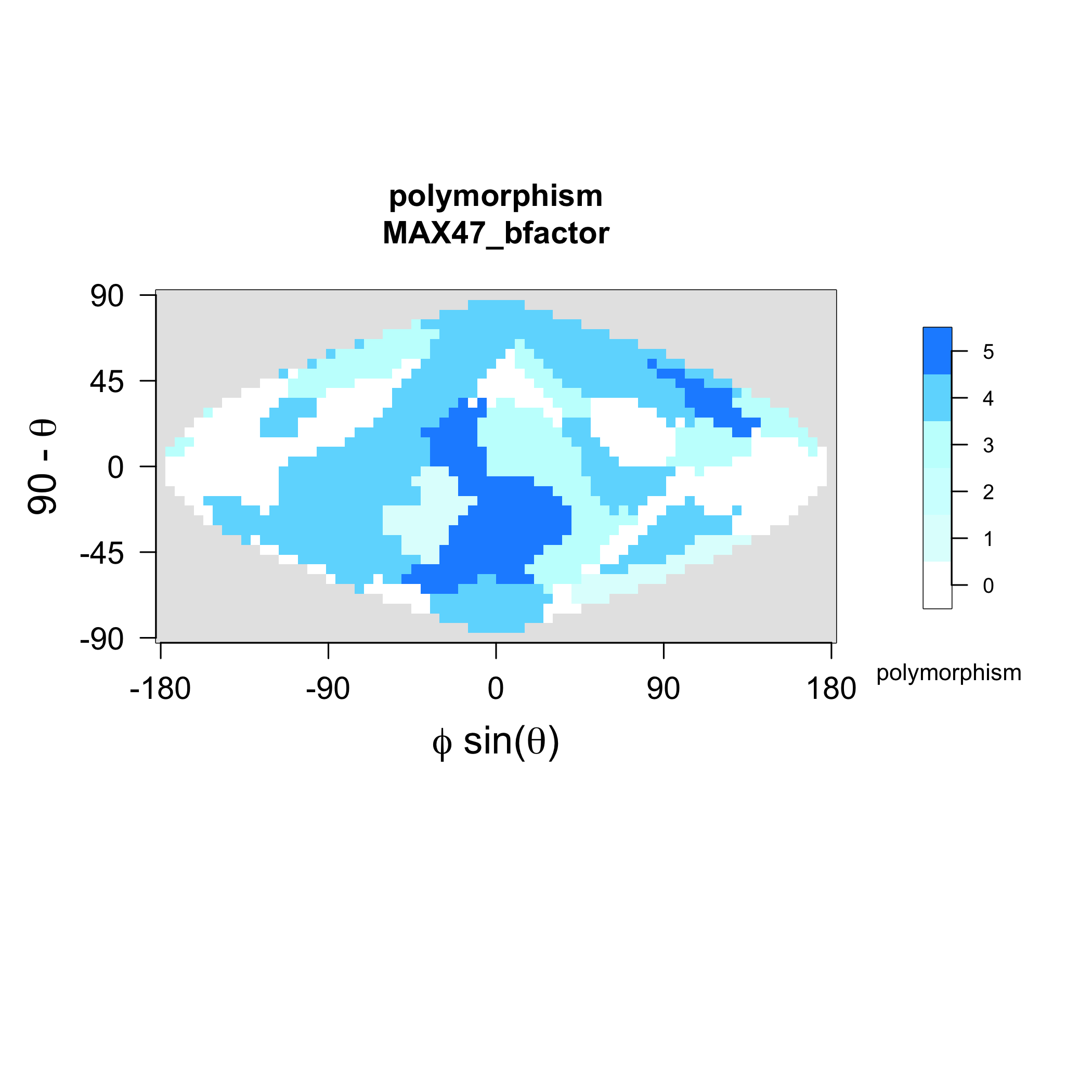

Supplement: S2 File — (ZIP) [file ppat.1012176.s019.zip › S2_File/POLYMORPHISM/MAX47_polymorphism.png]

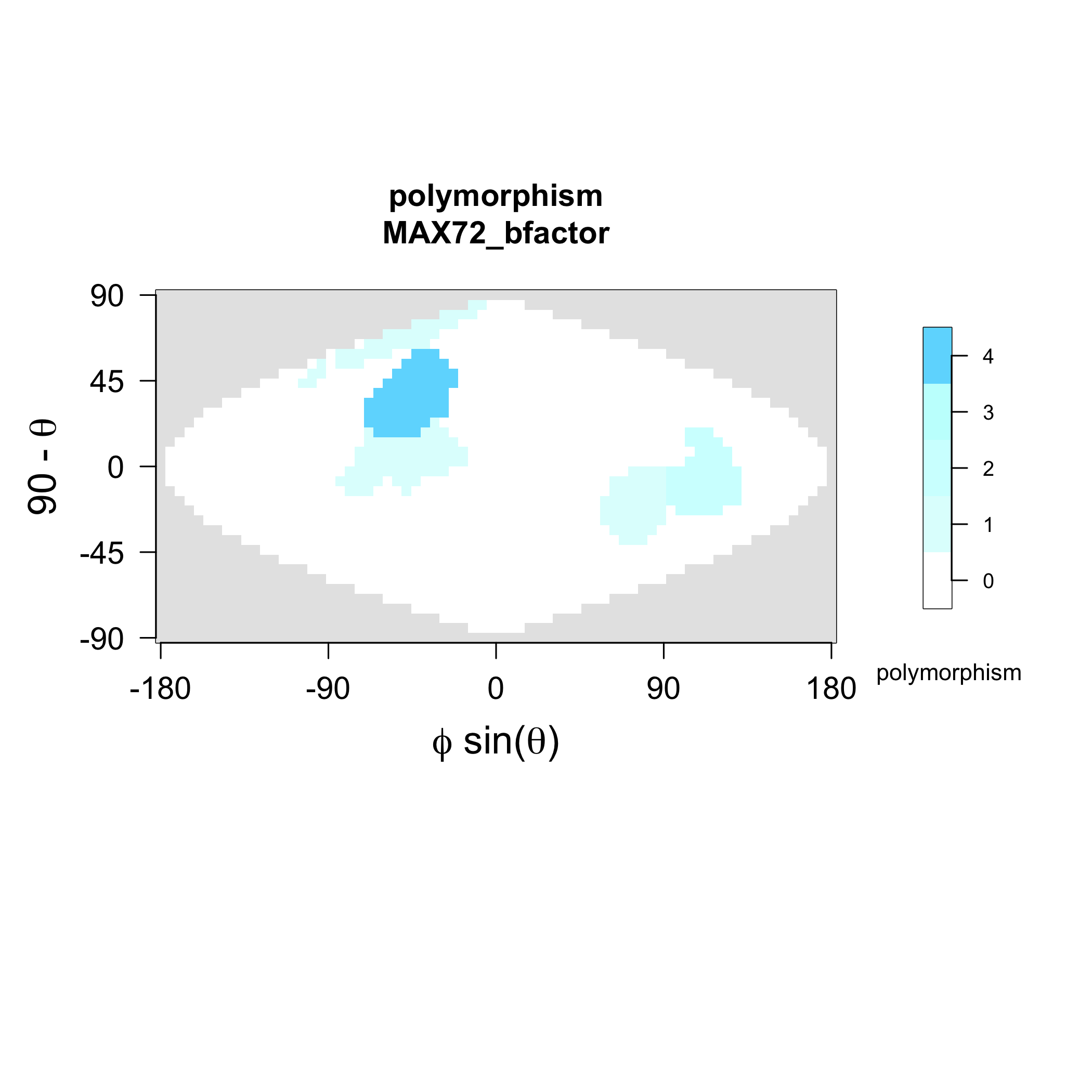

Supplement: S2 File — (ZIP) [file ppat.1012176.s019.zip › S2_File/POLYMORPHISM/MAX72_polymorphism.png]

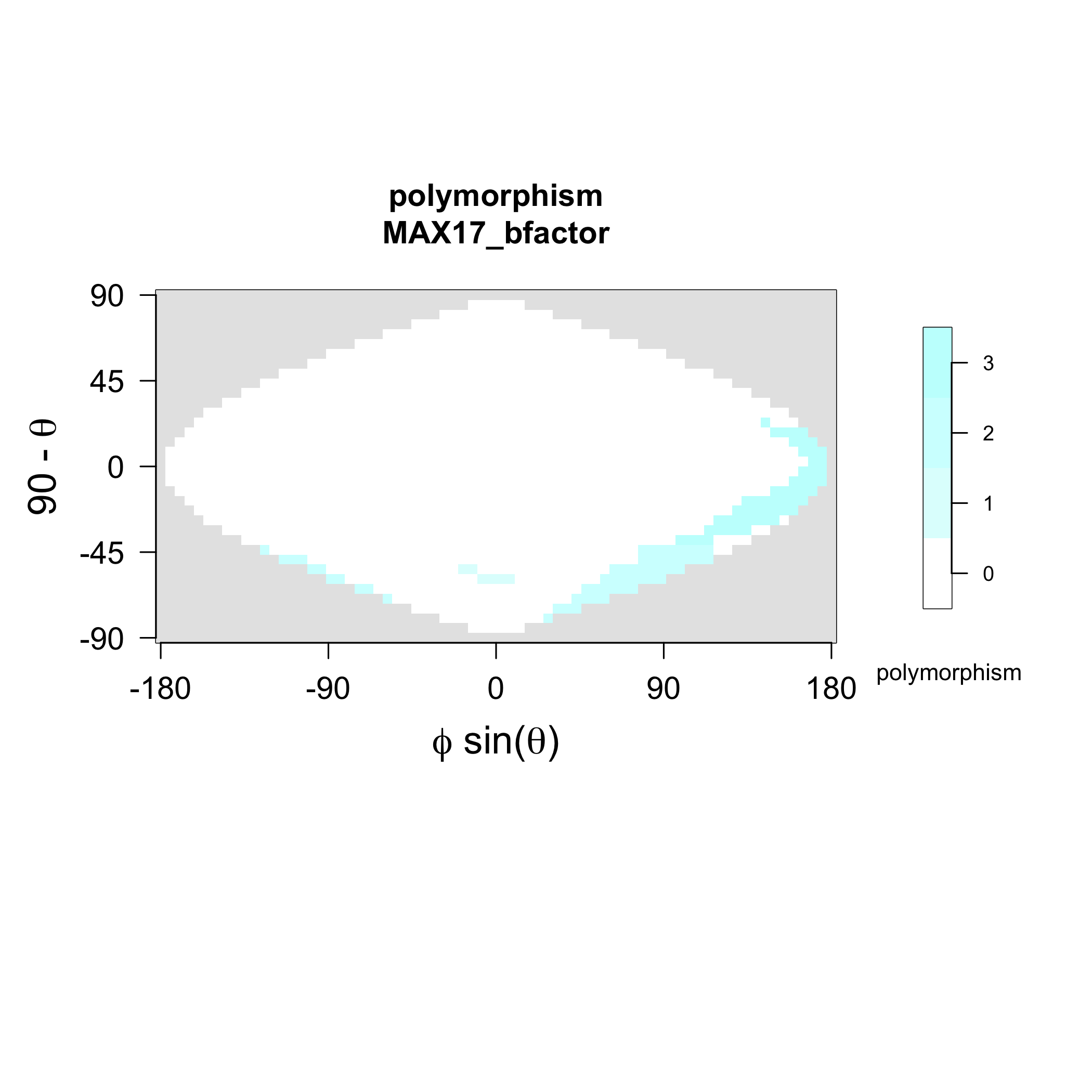

Supplement: S2 File — (ZIP) [file ppat.1012176.s019.zip › S2_File/POLYMORPHISM/MAX17_polymorphism.png]

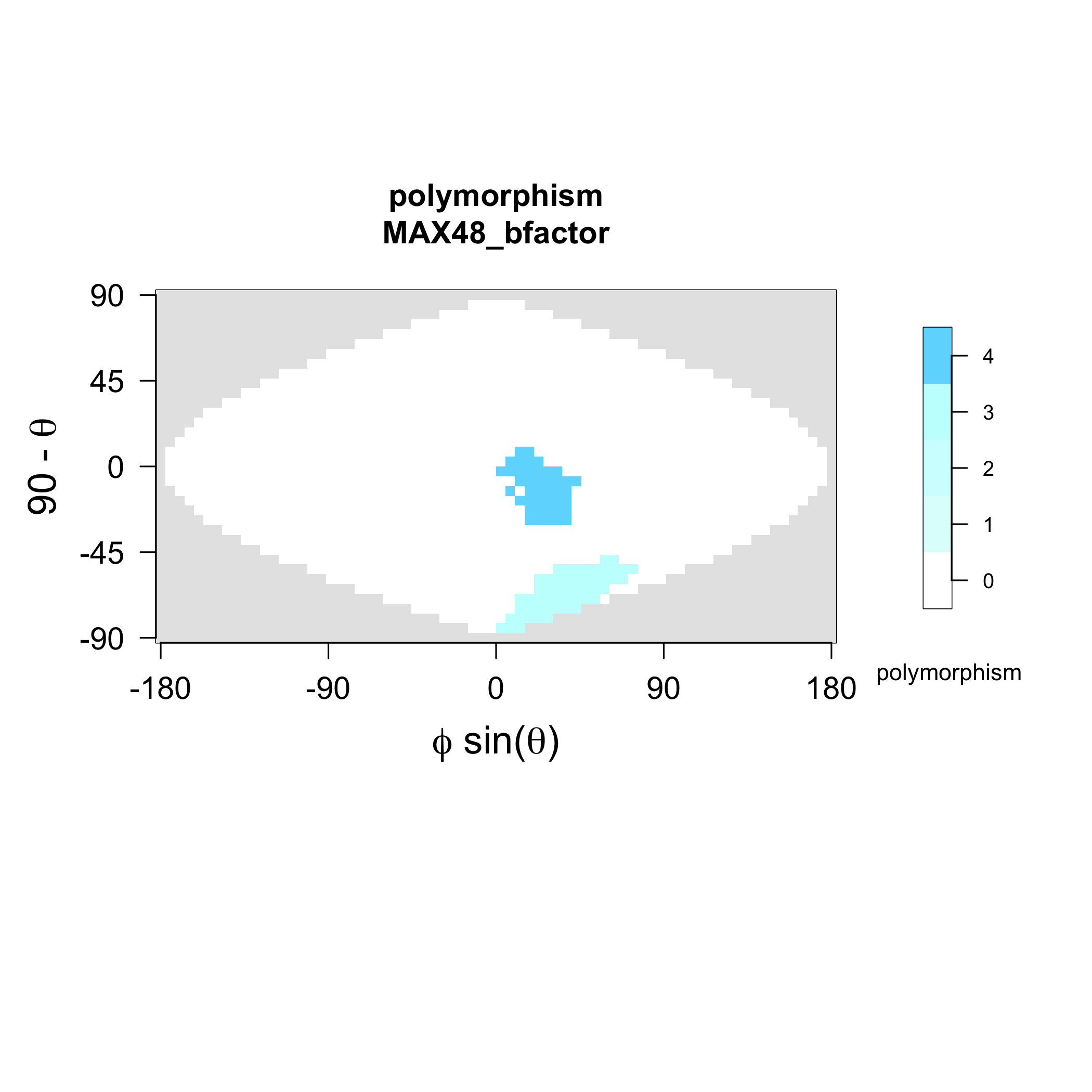

Supplement: S2 File — (ZIP) [file ppat.1012176.s019.zip › S2_File/POLYMORPHISM/MAX48_polymorphism.png]

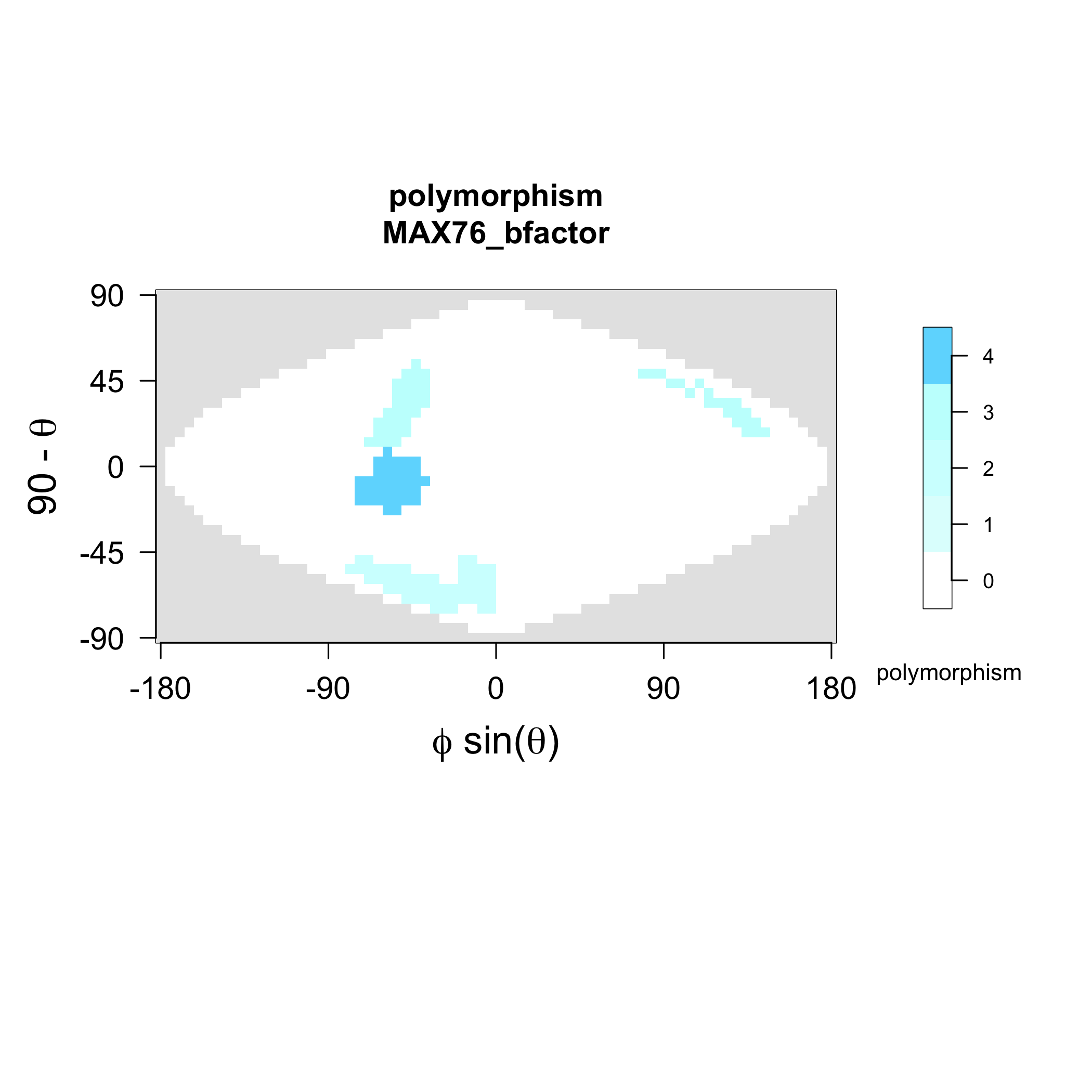

Supplement: S2 File — (ZIP) [file ppat.1012176.s019.zip › S2_File/POLYMORPHISM/MAX76_polymorphism.png]

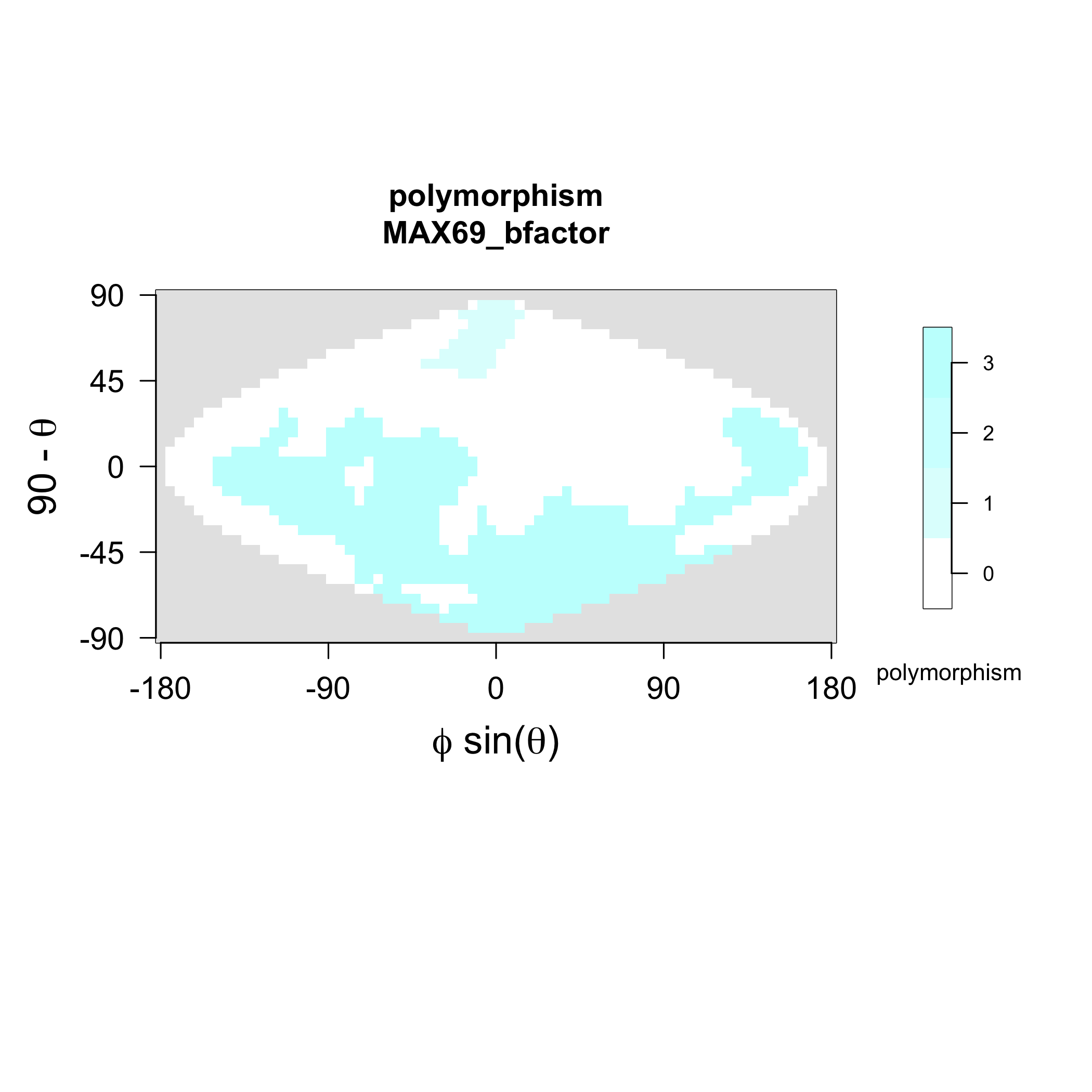

Supplement: S2 File — (ZIP) [file ppat.1012176.s019.zip › S2_File/POLYMORPHISM/MAX69_polymorphism.png]

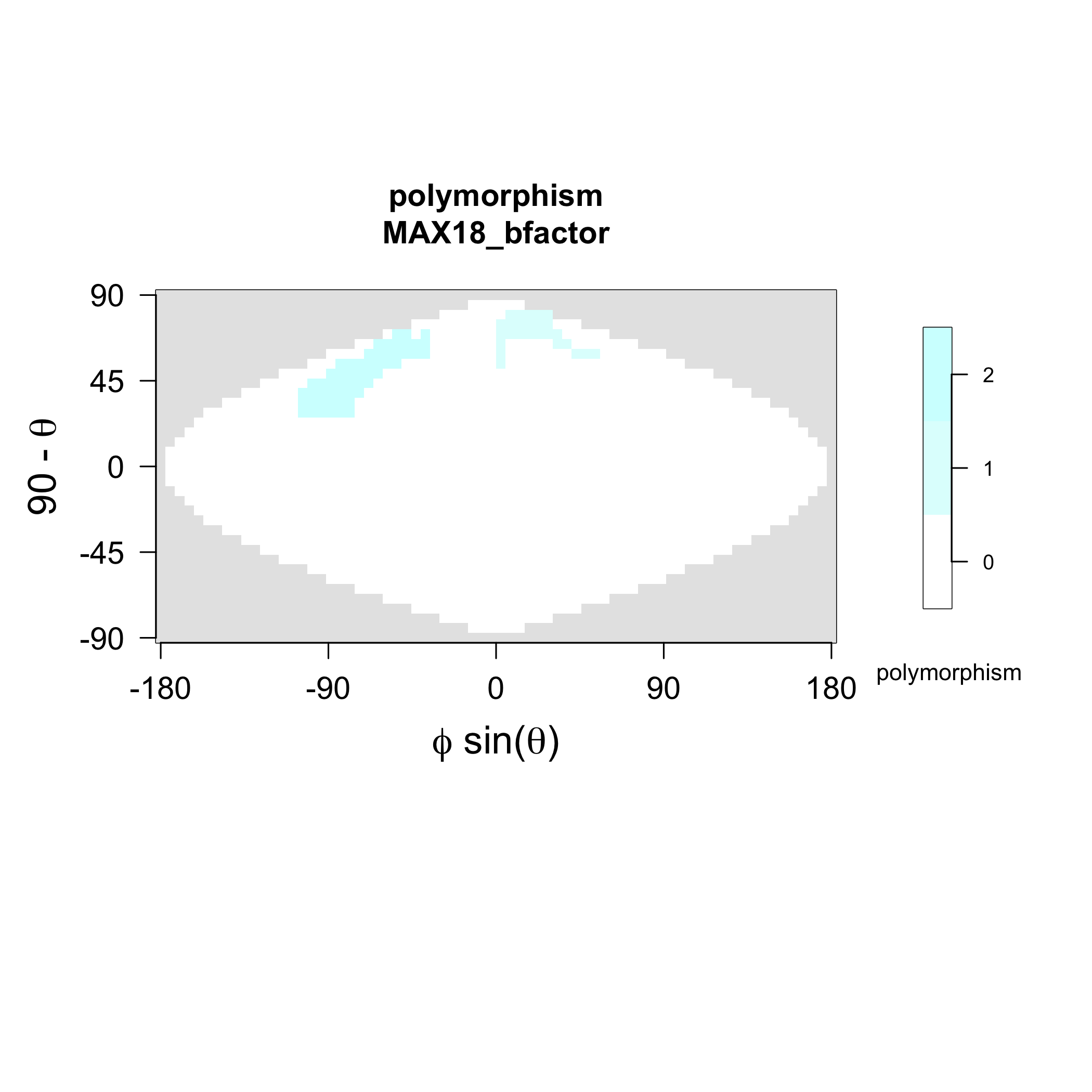

Supplement: S2 File — (ZIP) [file ppat.1012176.s019.zip › S2_File/POLYMORPHISM/MAX18_polymorphism.png]

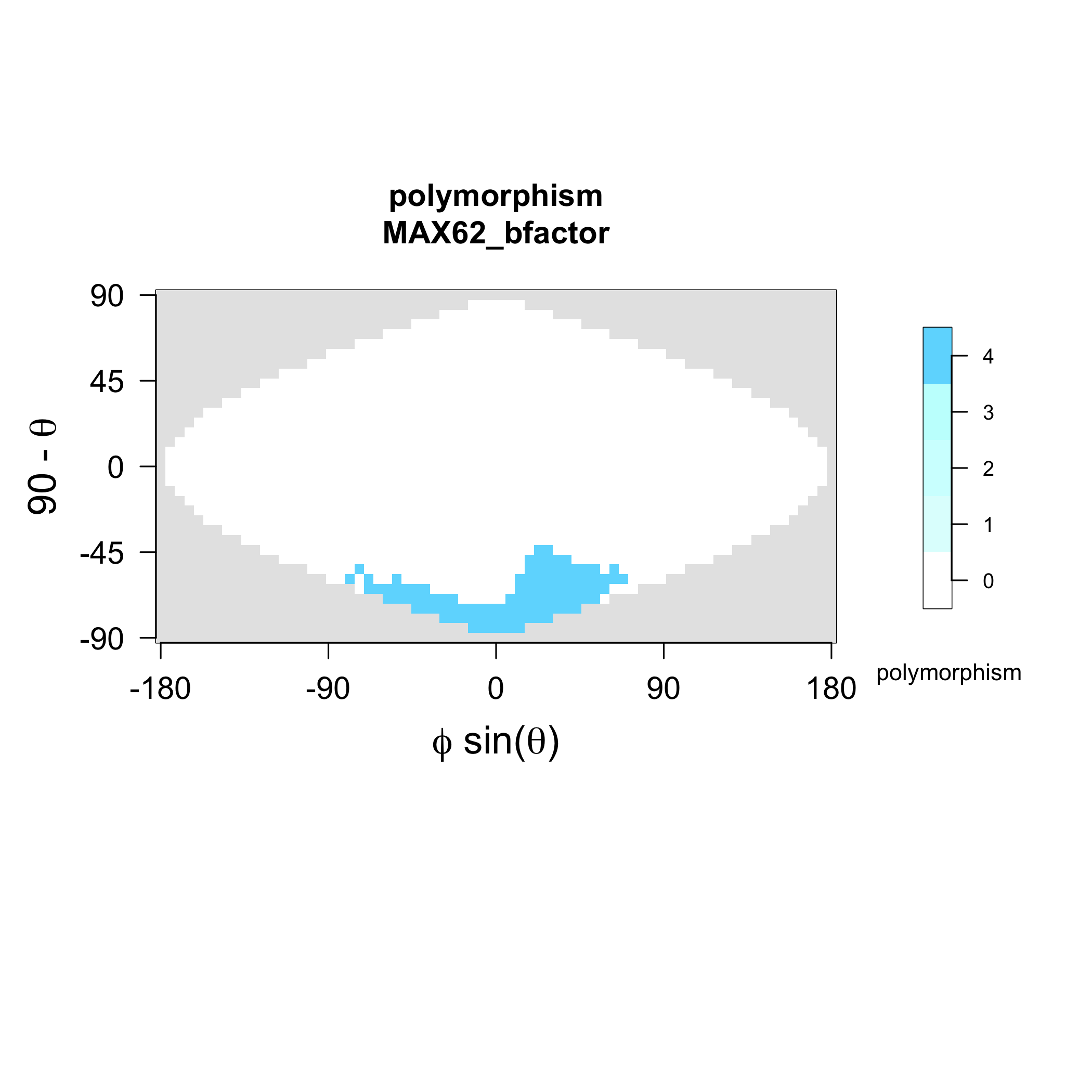

Supplement: S2 File — (ZIP) [file ppat.1012176.s019.zip › S2_File/POLYMORPHISM/MAX62_polymorphism.png]

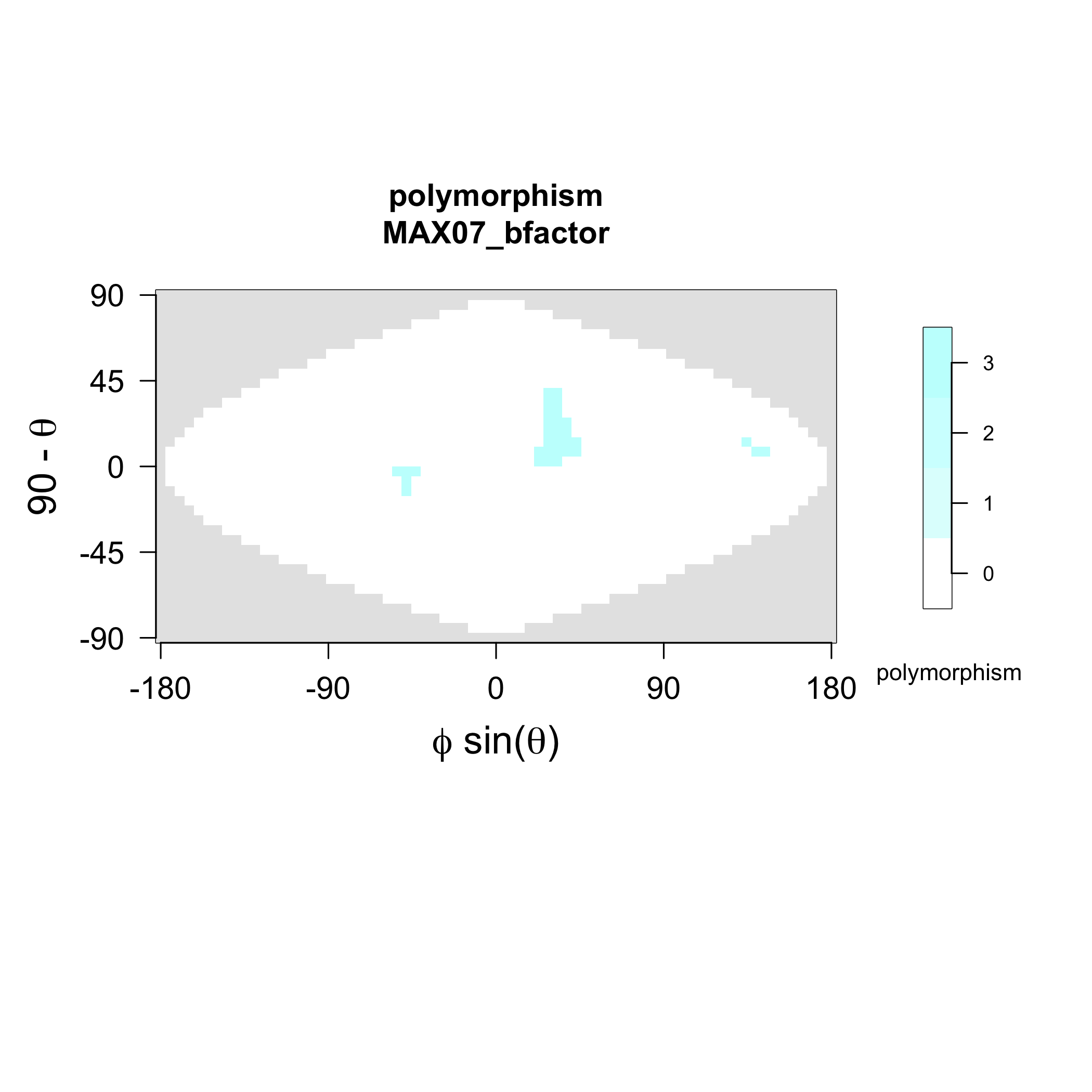

Supplement: S2 File — (ZIP) [file ppat.1012176.s019.zip › S2_File/POLYMORPHISM/MAX07_polymorphism.png]

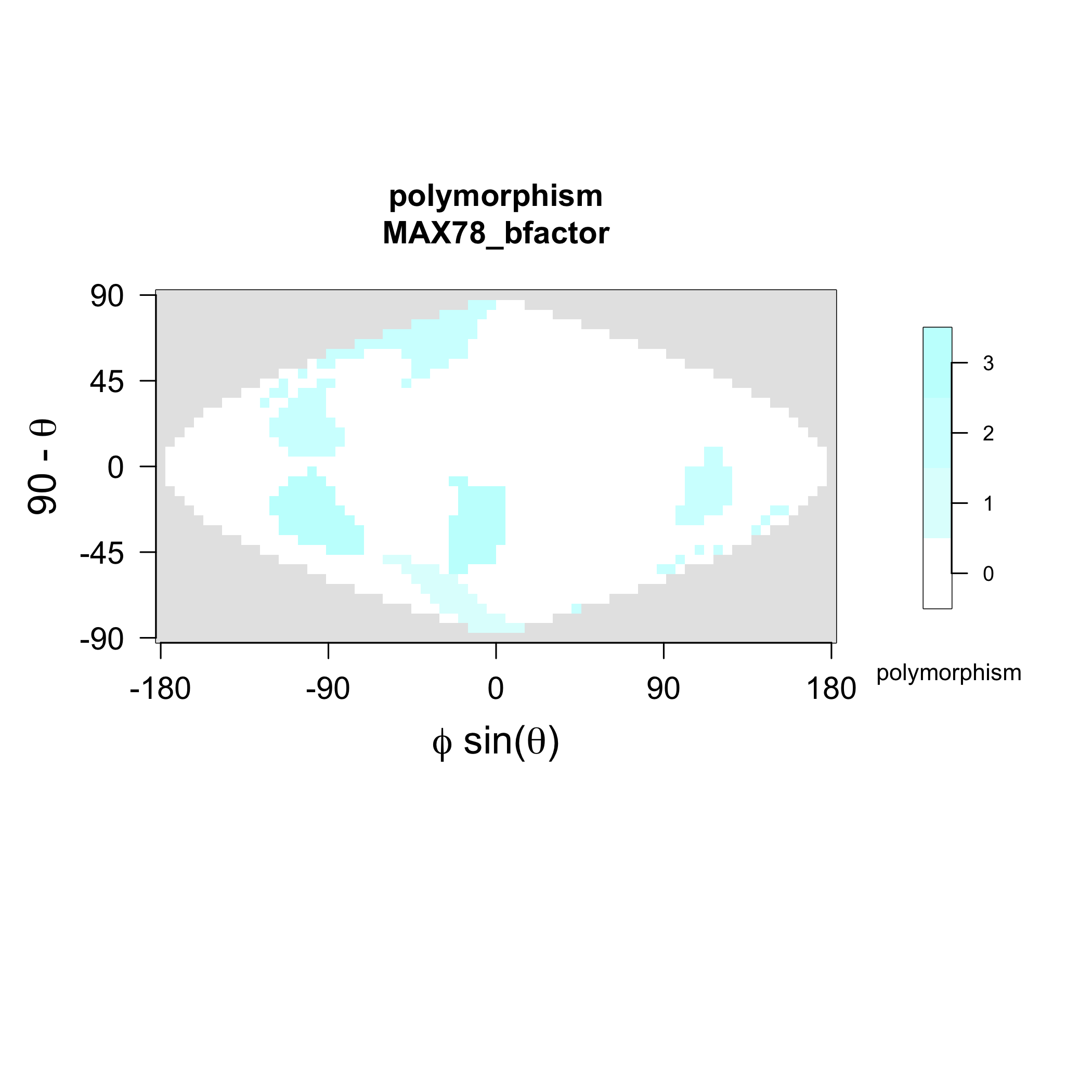

Supplement: S2 File — (ZIP) [file ppat.1012176.s019.zip › S2_File/POLYMORPHISM/MAX78_polymorphism.png]

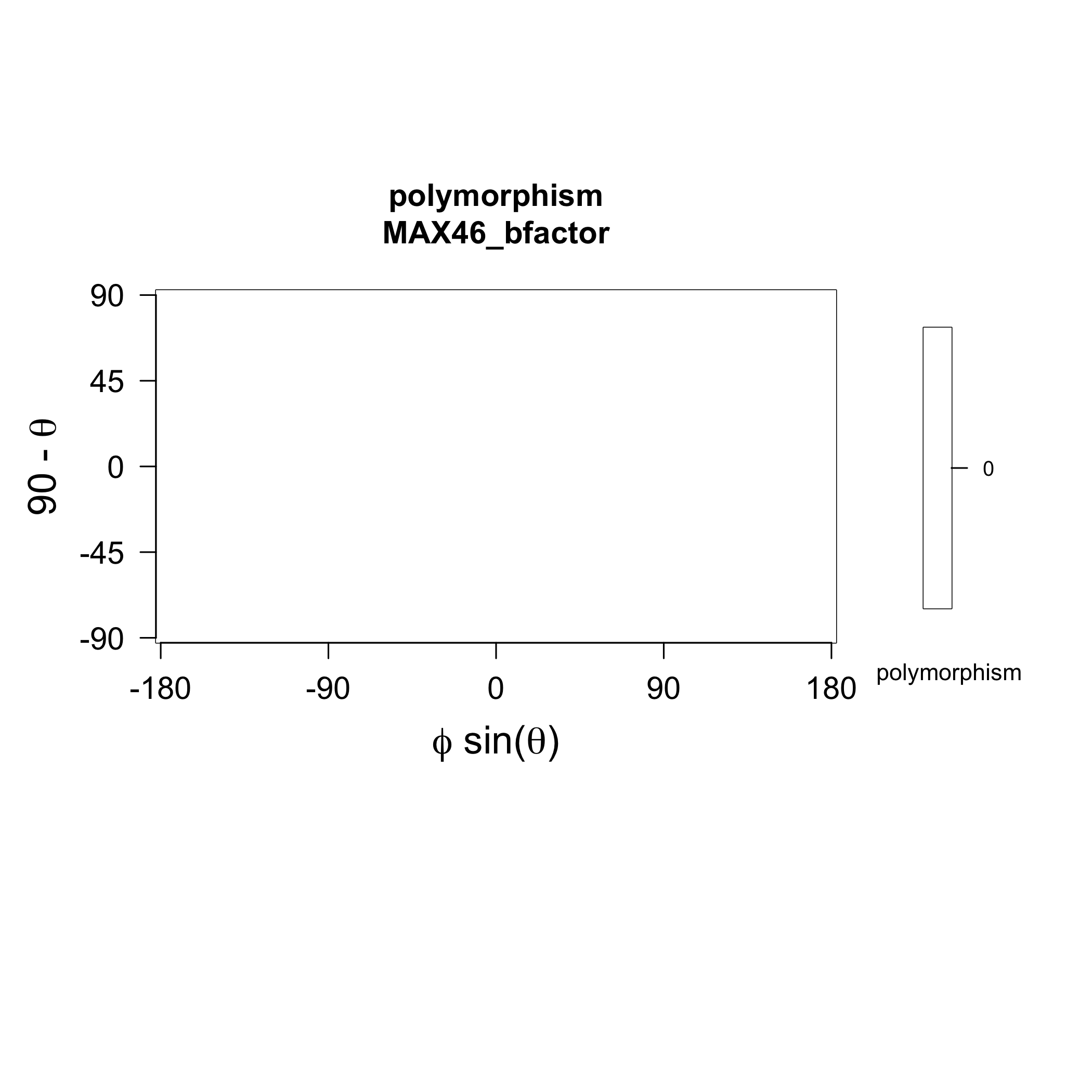

Supplement: S2 File — (ZIP) [file ppat.1012176.s019.zip › S2_File/POLYMORPHISM/MAX46_polymorphism.png]

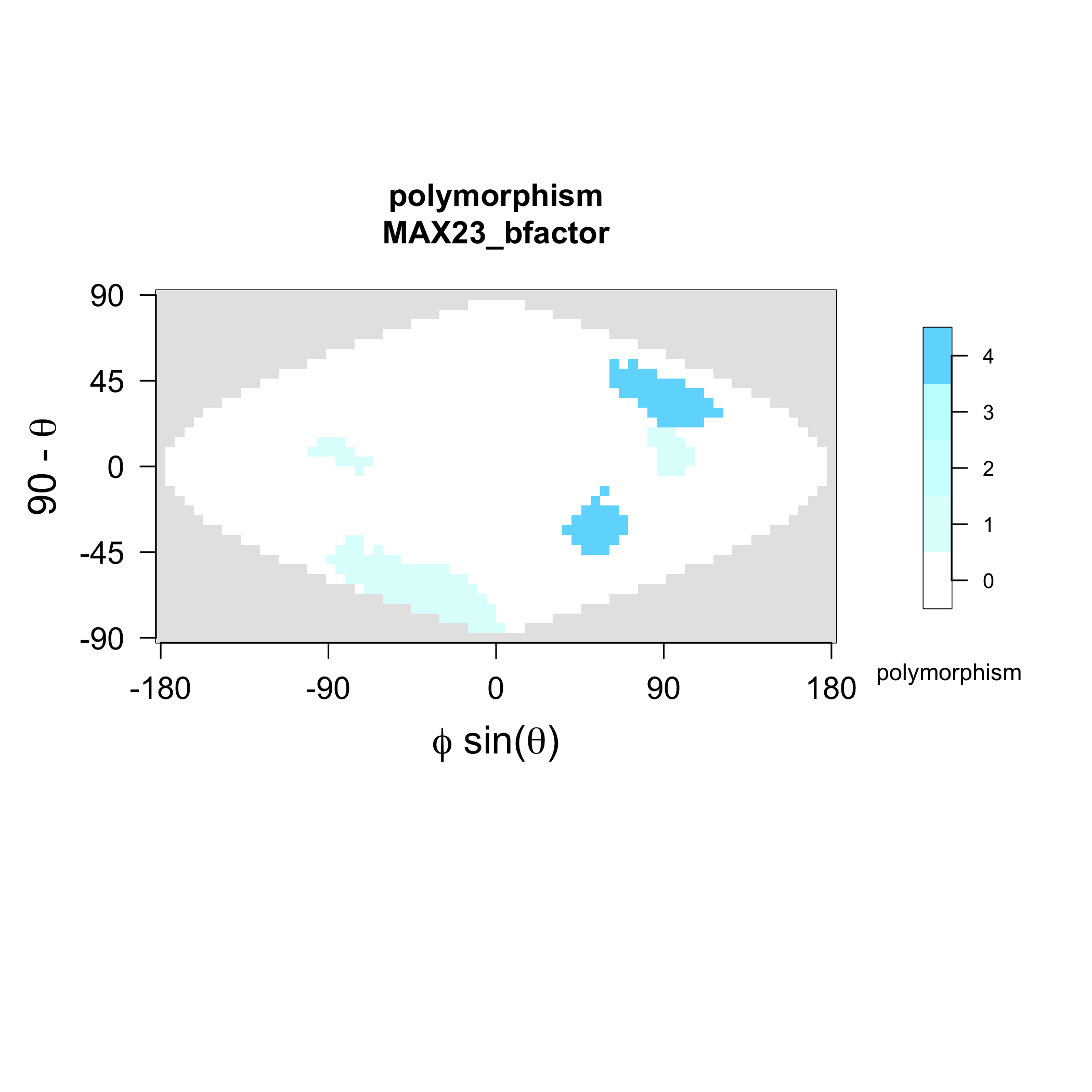

Supplement: S2 File — (ZIP) [file ppat.1012176.s019.zip › S2_File/POLYMORPHISM/MAX23_polymorphism.png]

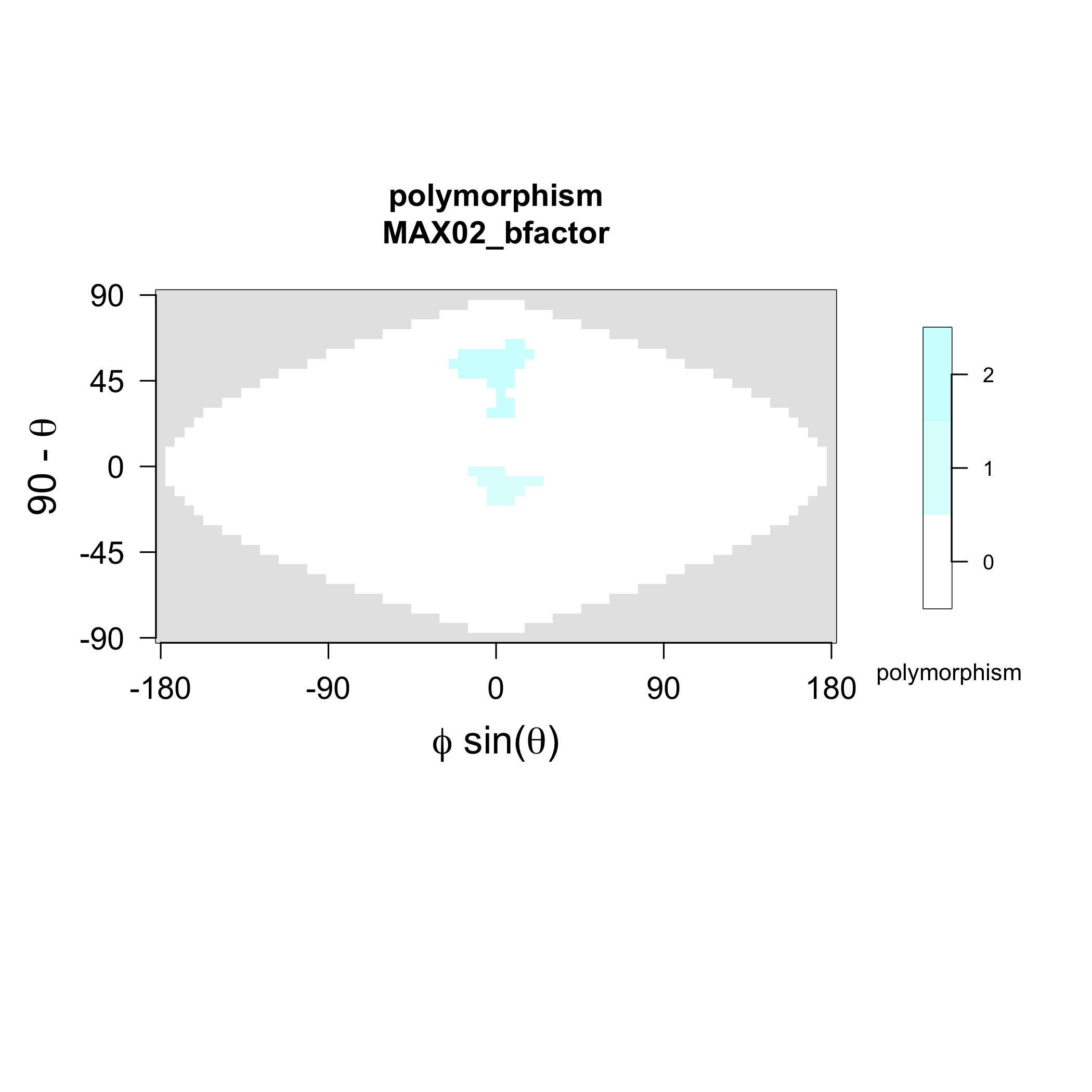

Supplement: S2 File — (ZIP) [file ppat.1012176.s019.zip › S2_File/POLYMORPHISM/MAX02_polymorphism.png]

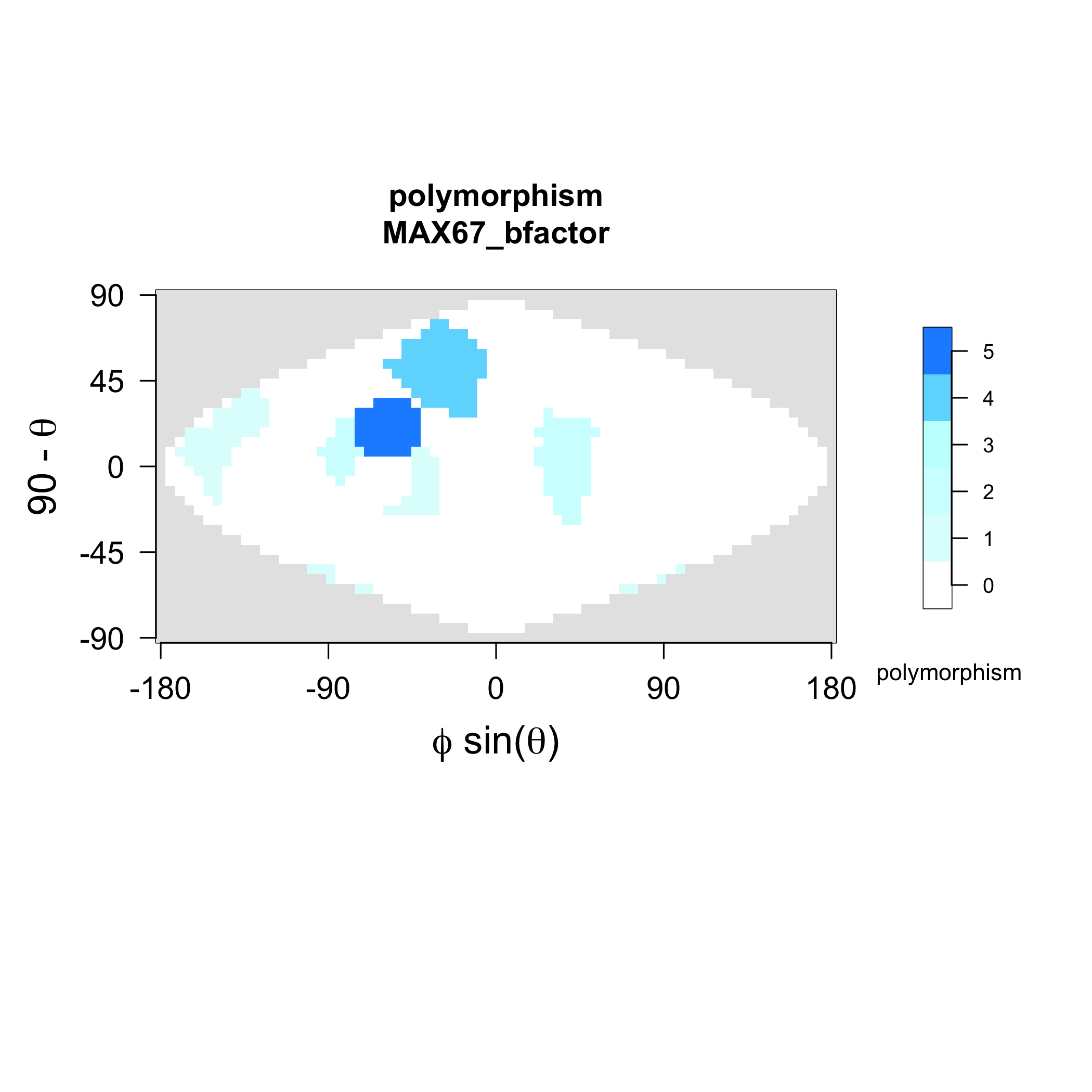

Supplement: S2 File — (ZIP) [file ppat.1012176.s019.zip › S2_File/POLYMORPHISM/MAX67_polymorphism.png]

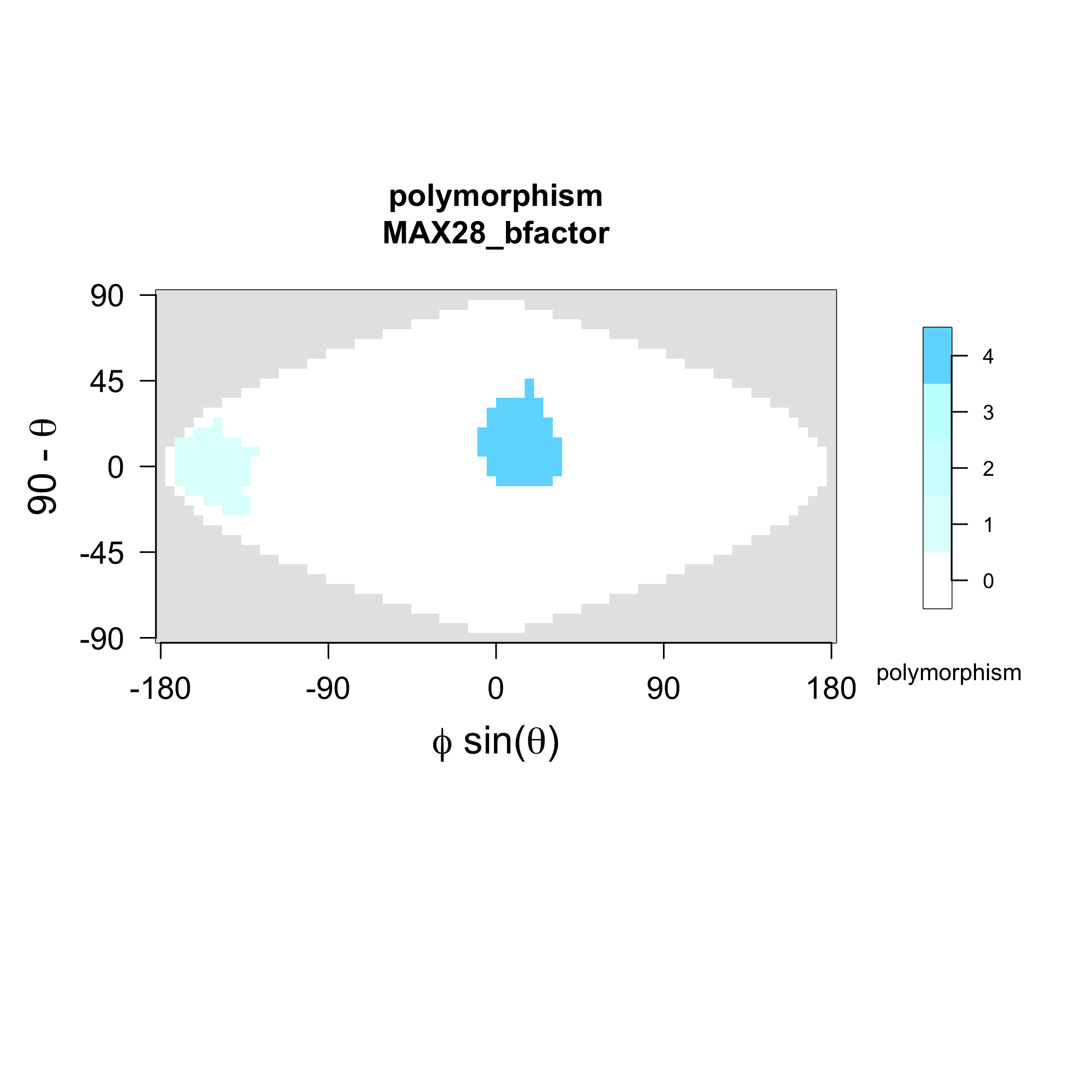

Supplement: S2 File — (ZIP) [file ppat.1012176.s019.zip › S2_File/POLYMORPHISM/MAX28_polymorphism.png]

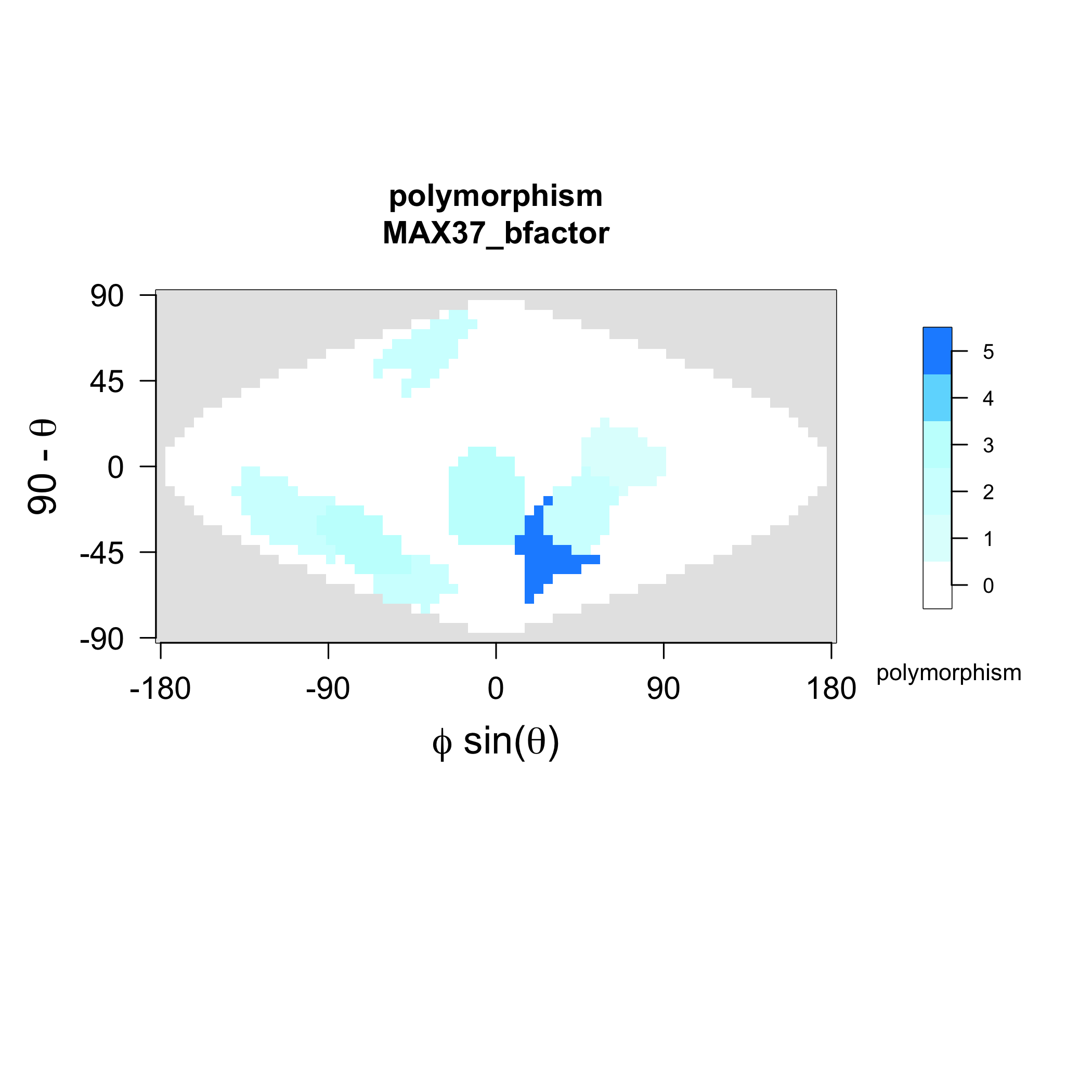

Supplement: S2 File — (ZIP) [file ppat.1012176.s019.zip › S2_File/POLYMORPHISM/MAX37_polymorphism.png]

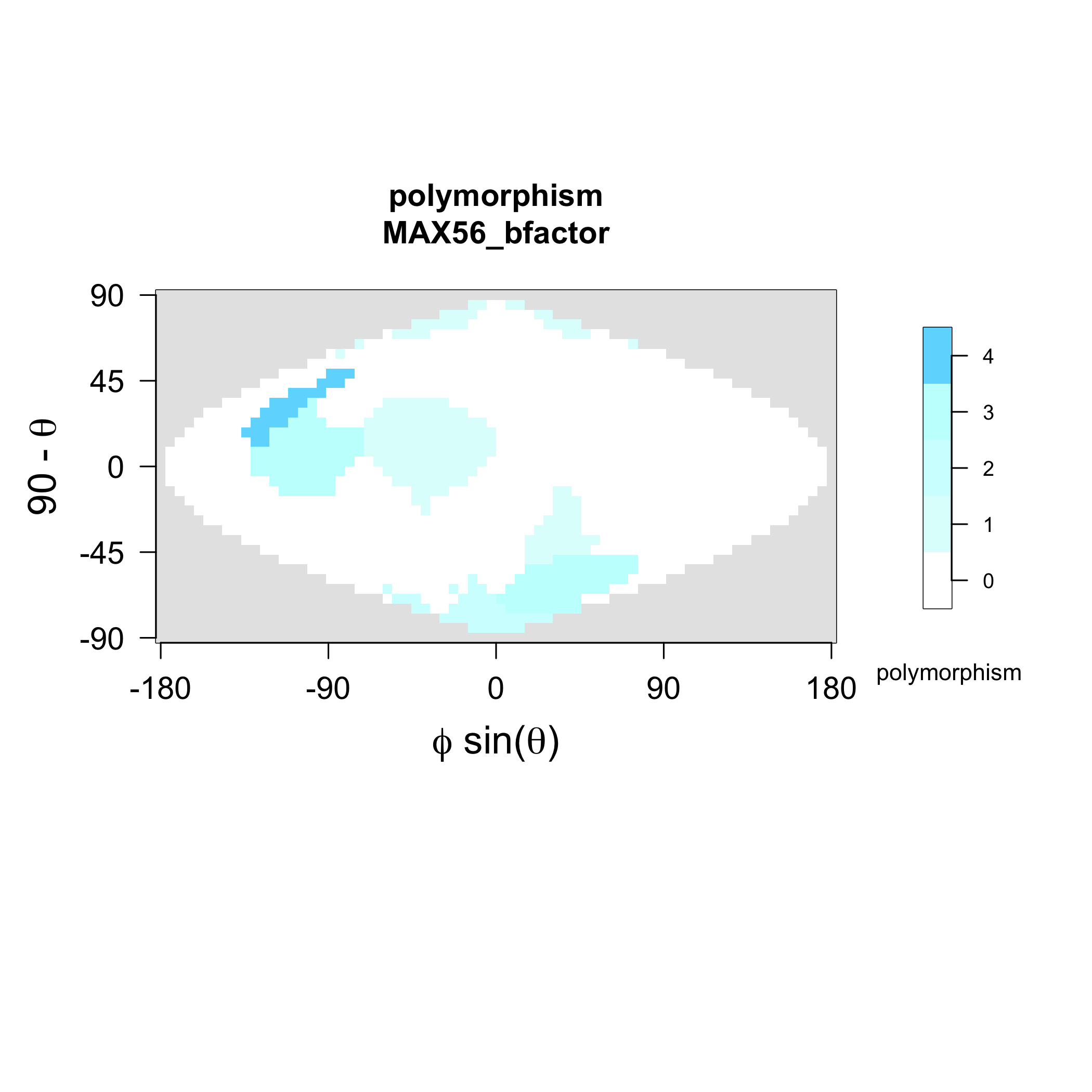

Supplement: S2 File — (ZIP) [file ppat.1012176.s019.zip › S2_File/POLYMORPHISM/MAX56_polymorphism.png]

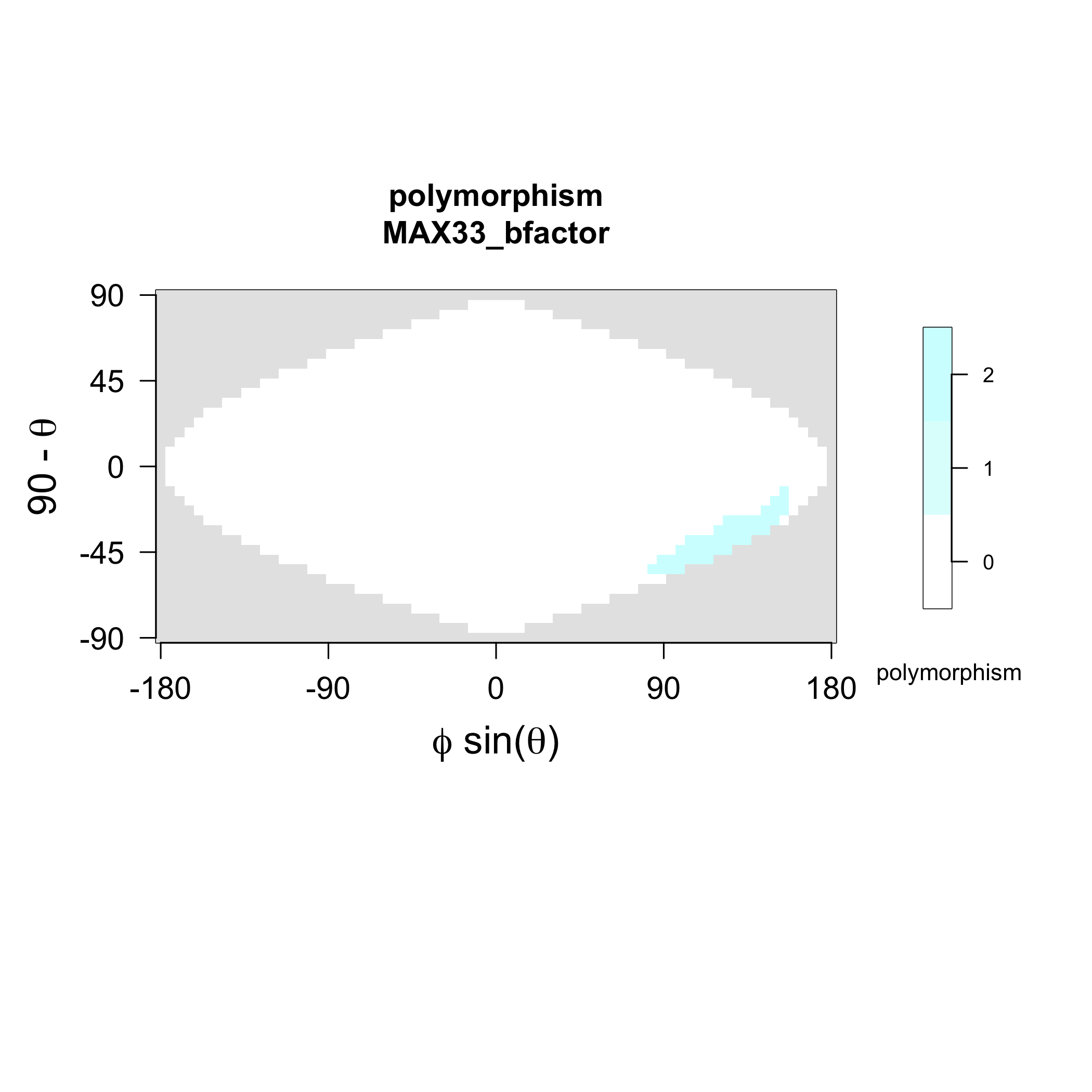

Supplement: S2 File — (ZIP) [file ppat.1012176.s019.zip › S2_File/POLYMORPHISM/MAX33_polymorphism.png]

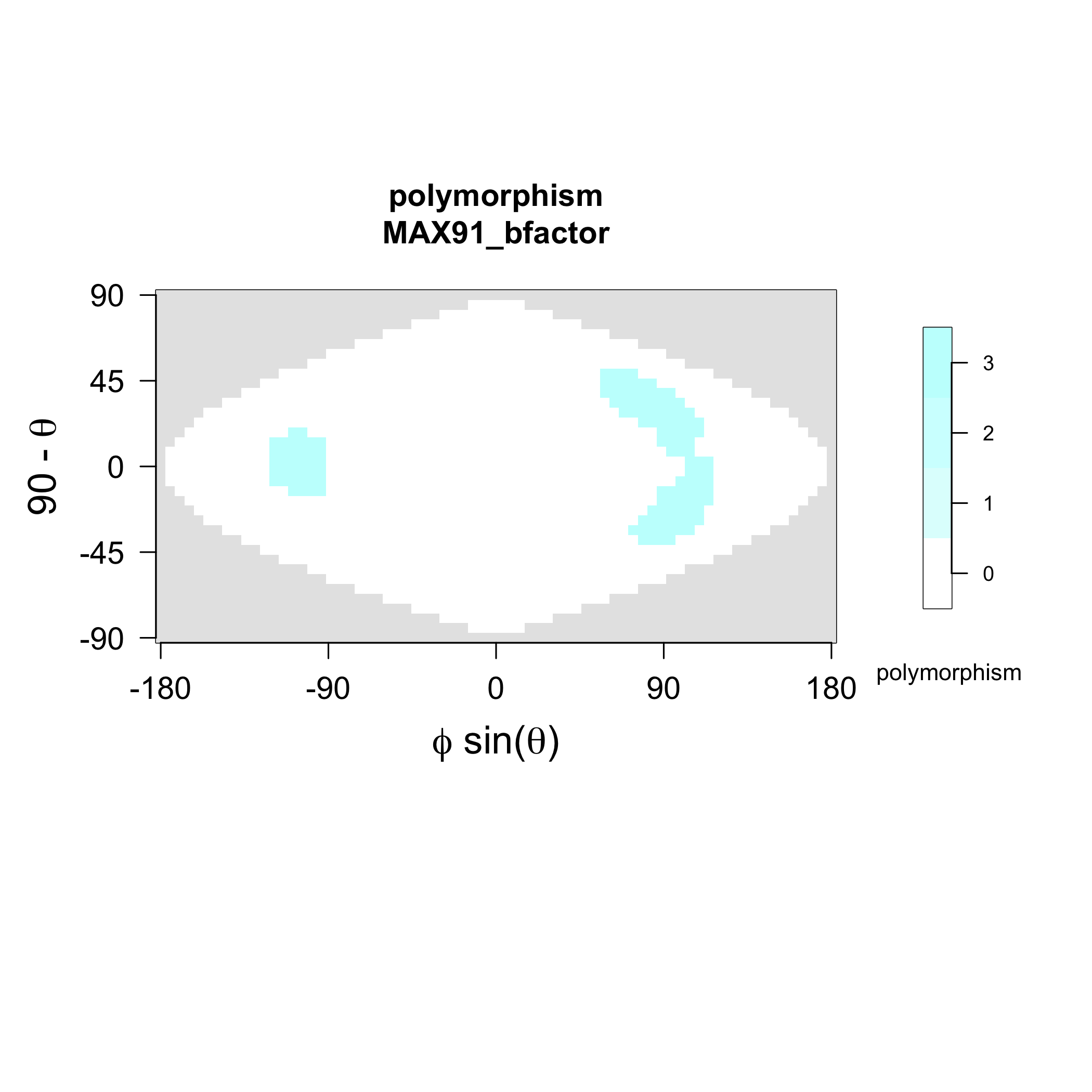

Supplement: S2 File — (ZIP) [file ppat.1012176.s019.zip › S2_File/POLYMORPHISM/MAX91_polymorphism.png]

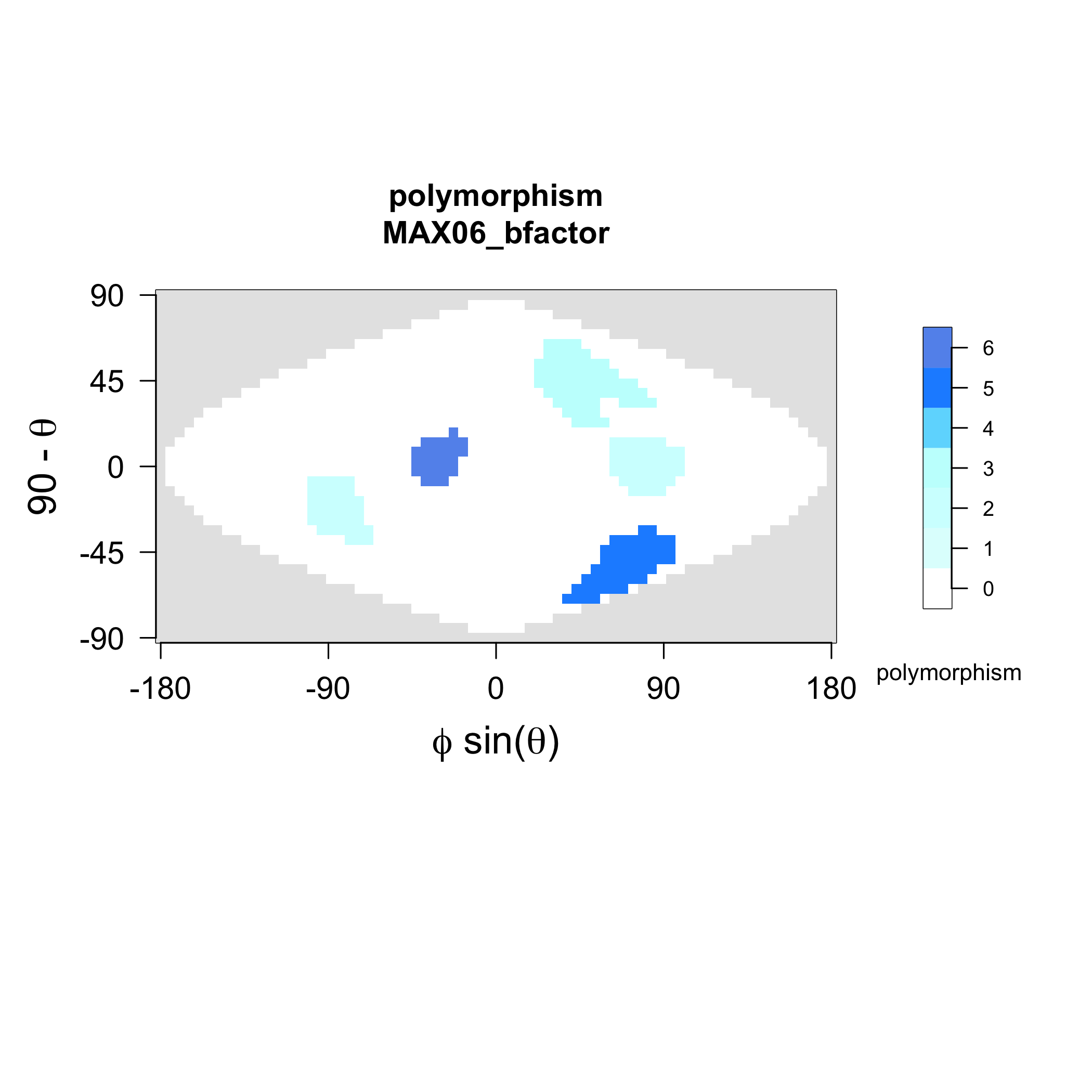

Supplement: S2 File — (ZIP) [file ppat.1012176.s019.zip › S2_File/POLYMORPHISM/MAX06_polymorphism.png]

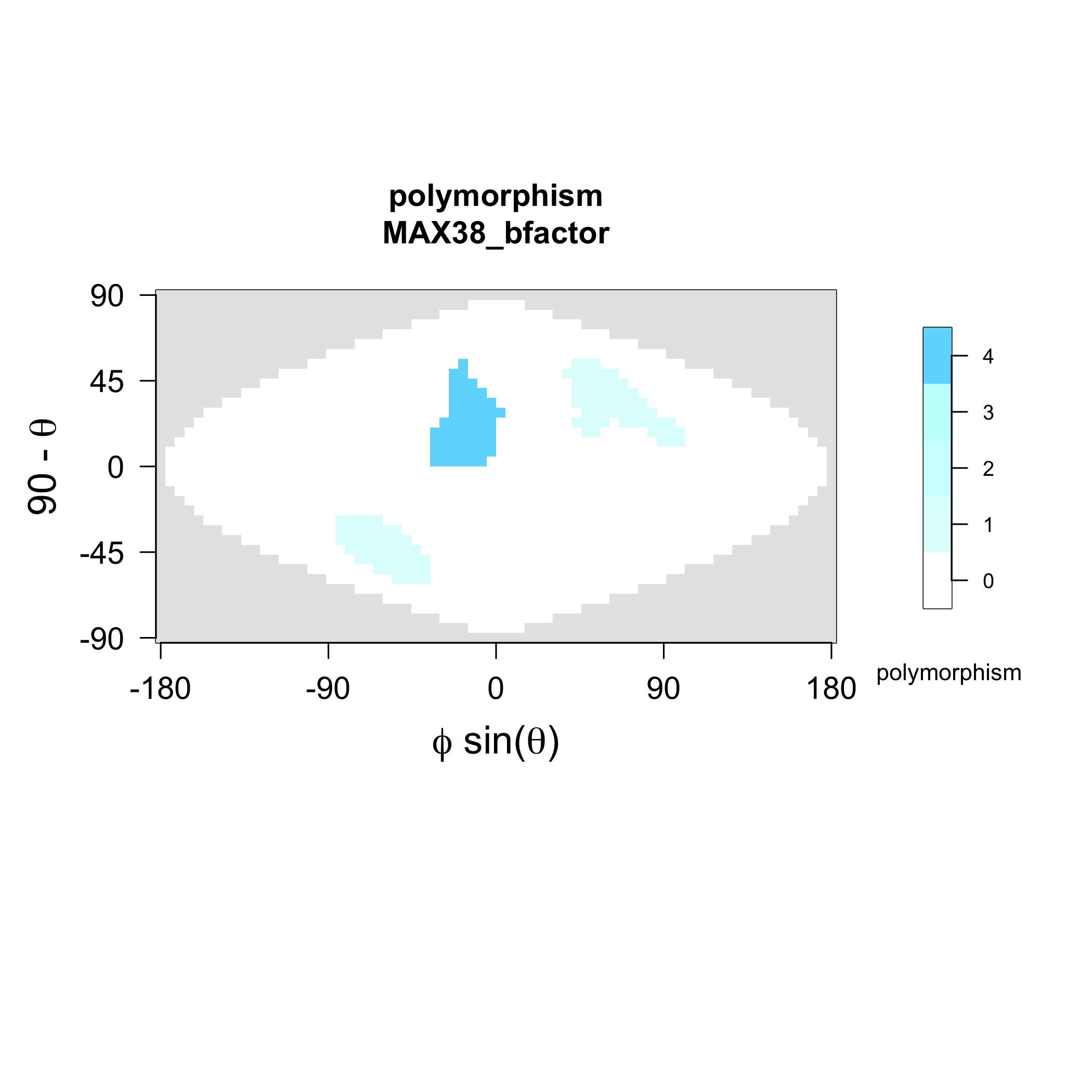

Supplement: S2 File — (ZIP) [file ppat.1012176.s019.zip › S2_File/POLYMORPHISM/MAX38_polymorphism.png]

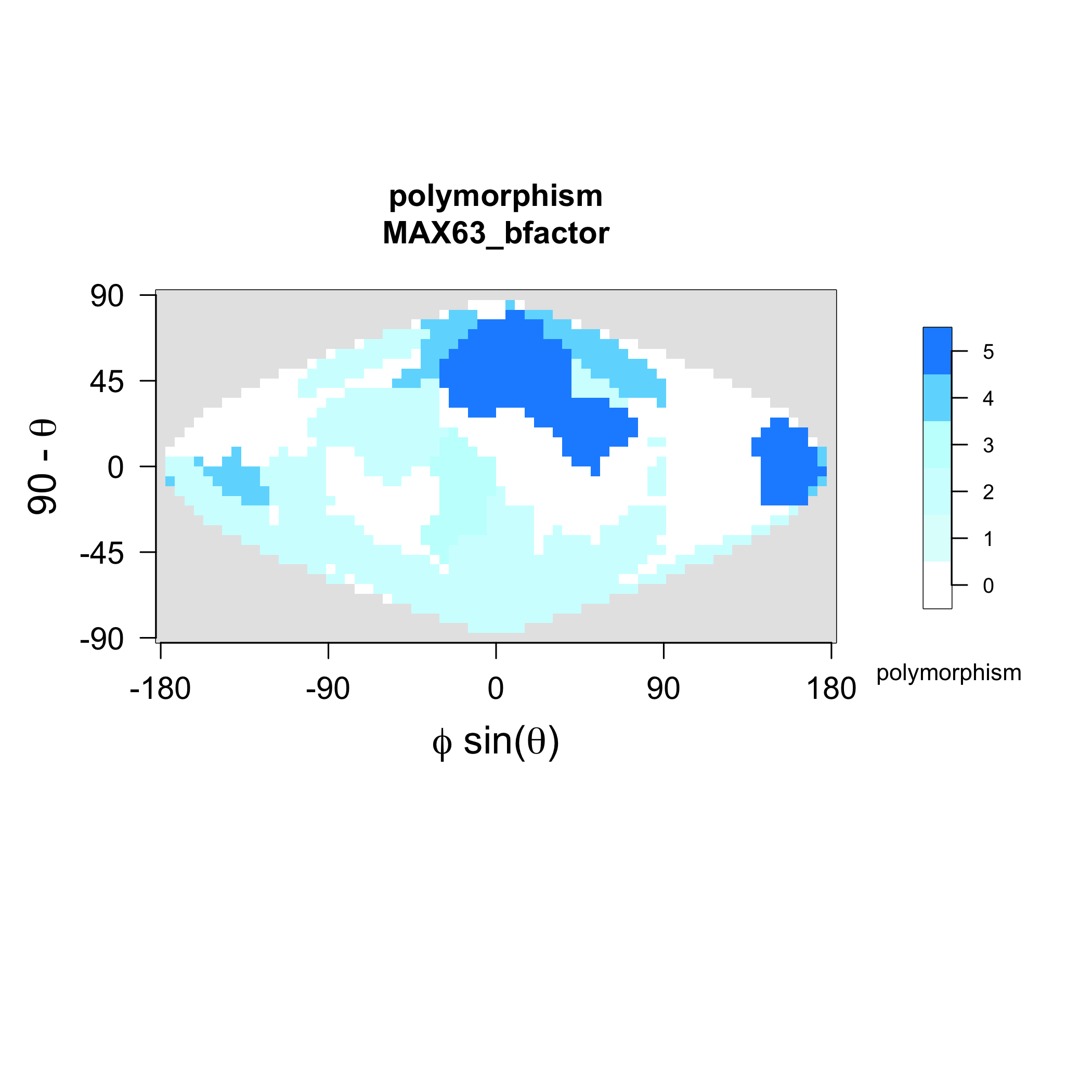

Supplement: S2 File — (ZIP) [file ppat.1012176.s019.zip › S2_File/POLYMORPHISM/MAX63_polymorphism.png]

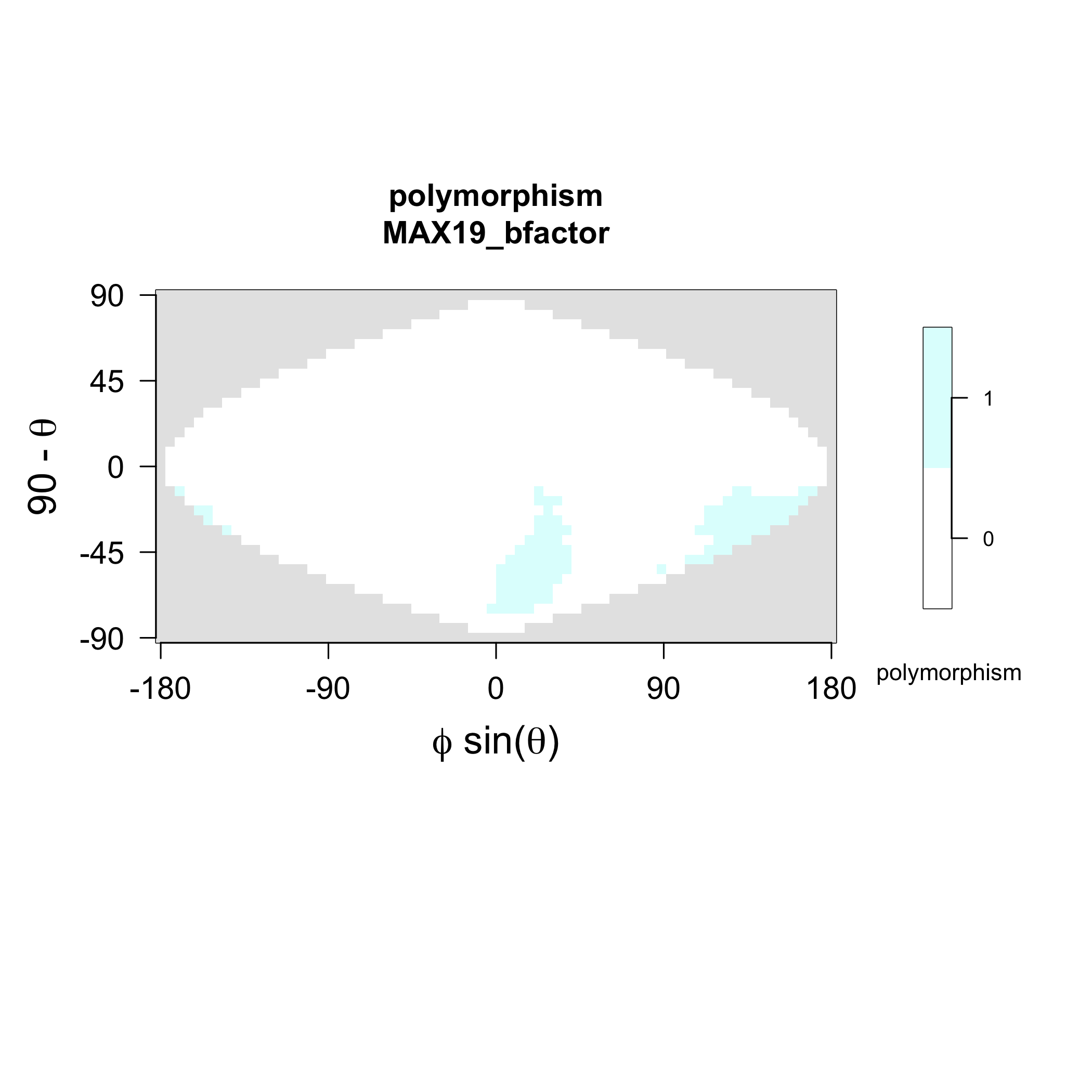

Supplement: S2 File — (ZIP) [file ppat.1012176.s019.zip › S2_File/POLYMORPHISM/MAX19_polymorphism.png]

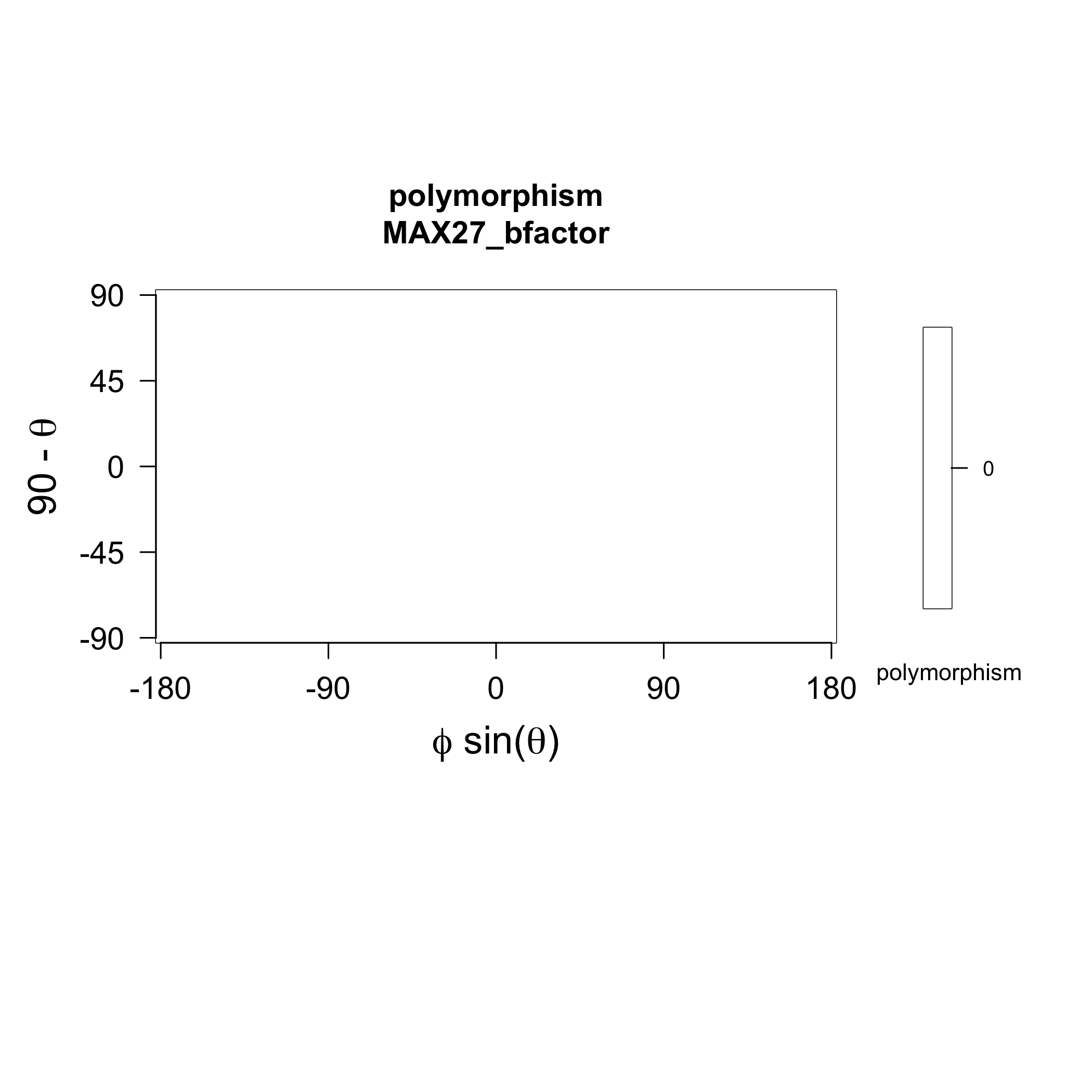

Supplement: S2 File — (ZIP) [file ppat.1012176.s019.zip › S2_File/POLYMORPHISM/MAX27_polymorphism.png]

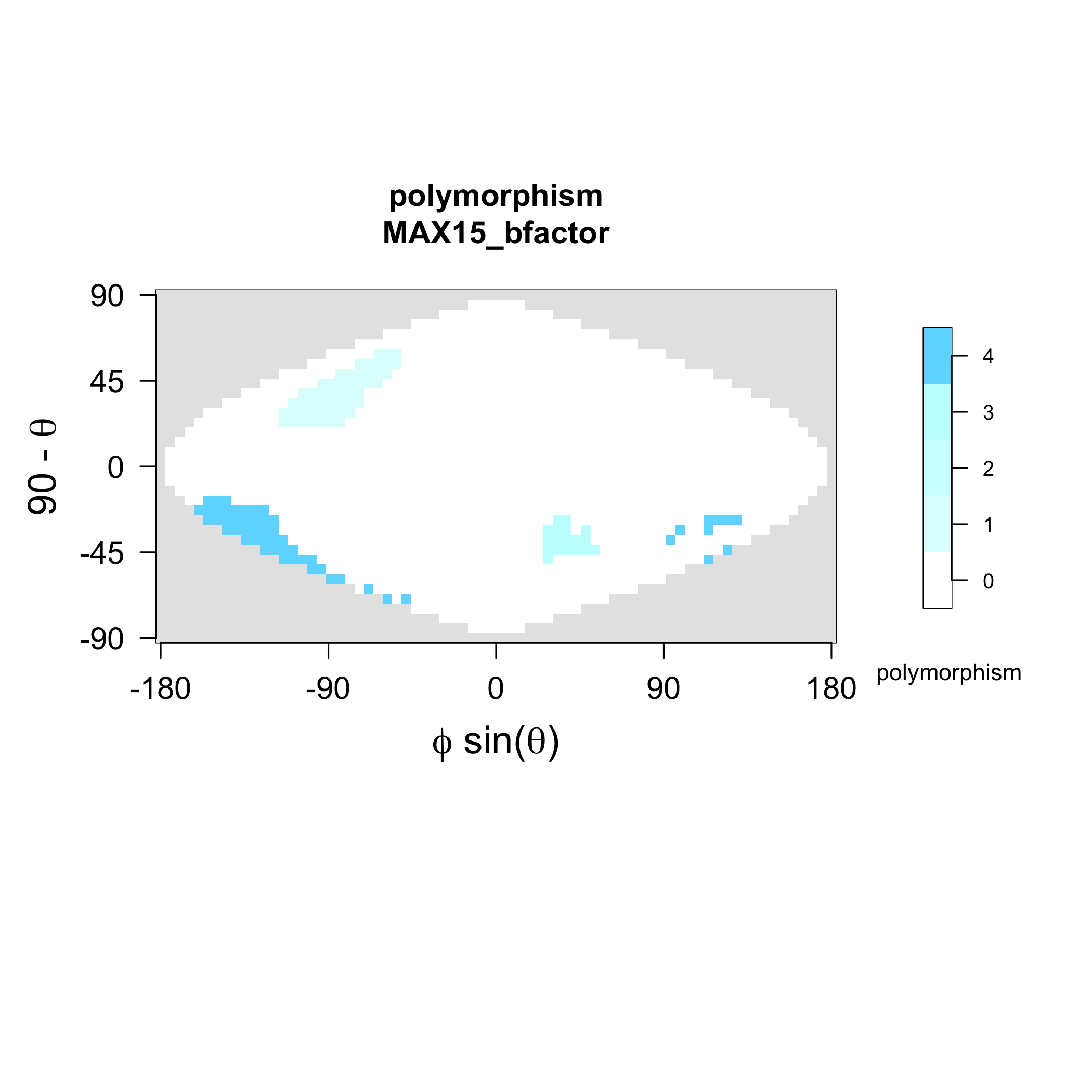

Supplement: S2 File — (ZIP) [file ppat.1012176.s019.zip › S2_File/POLYMORPHISM/MAX15_polymorphism.png]

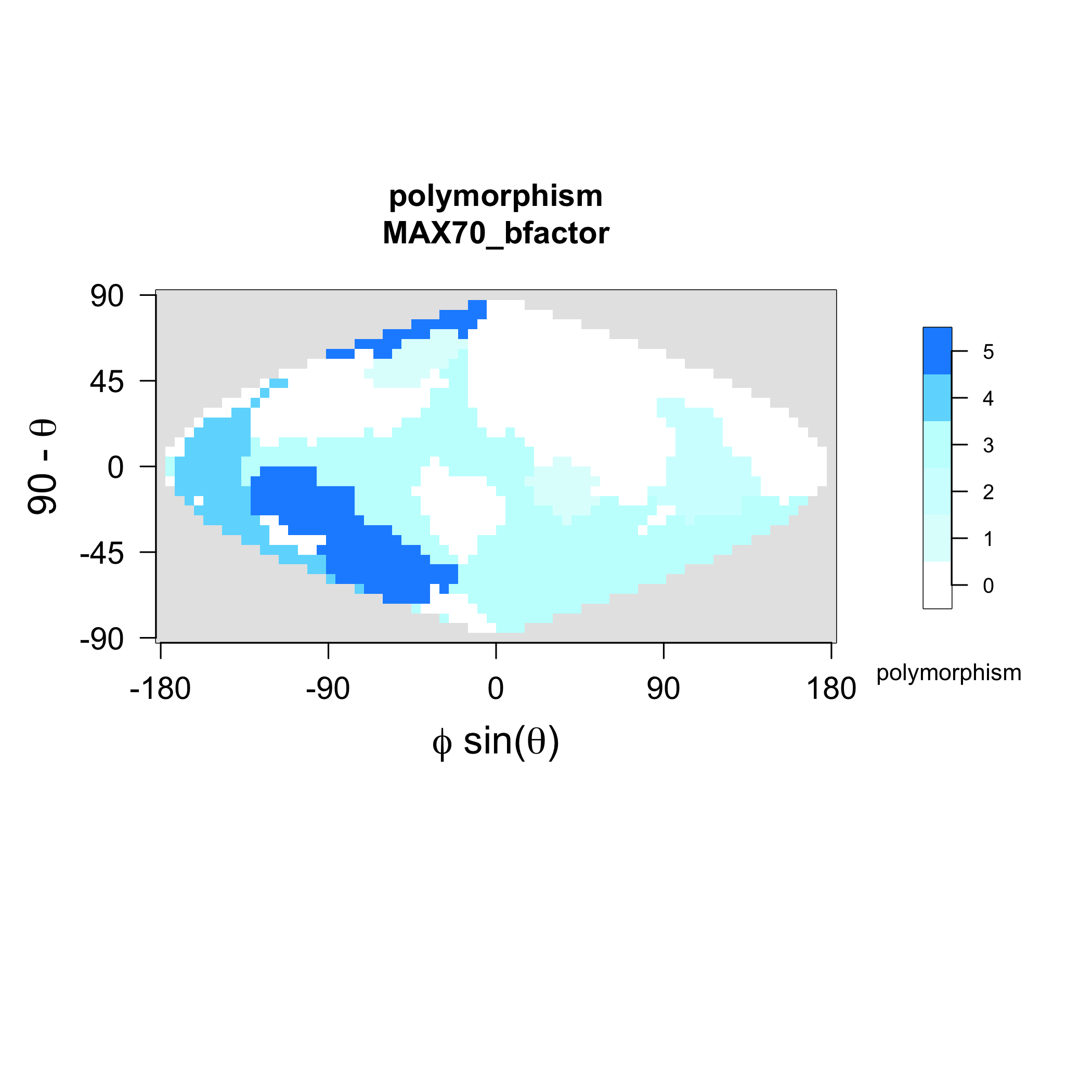

Supplement: S2 File — (ZIP) [file ppat.1012176.s019.zip › S2_File/POLYMORPHISM/MAX70_polymorphism.png]

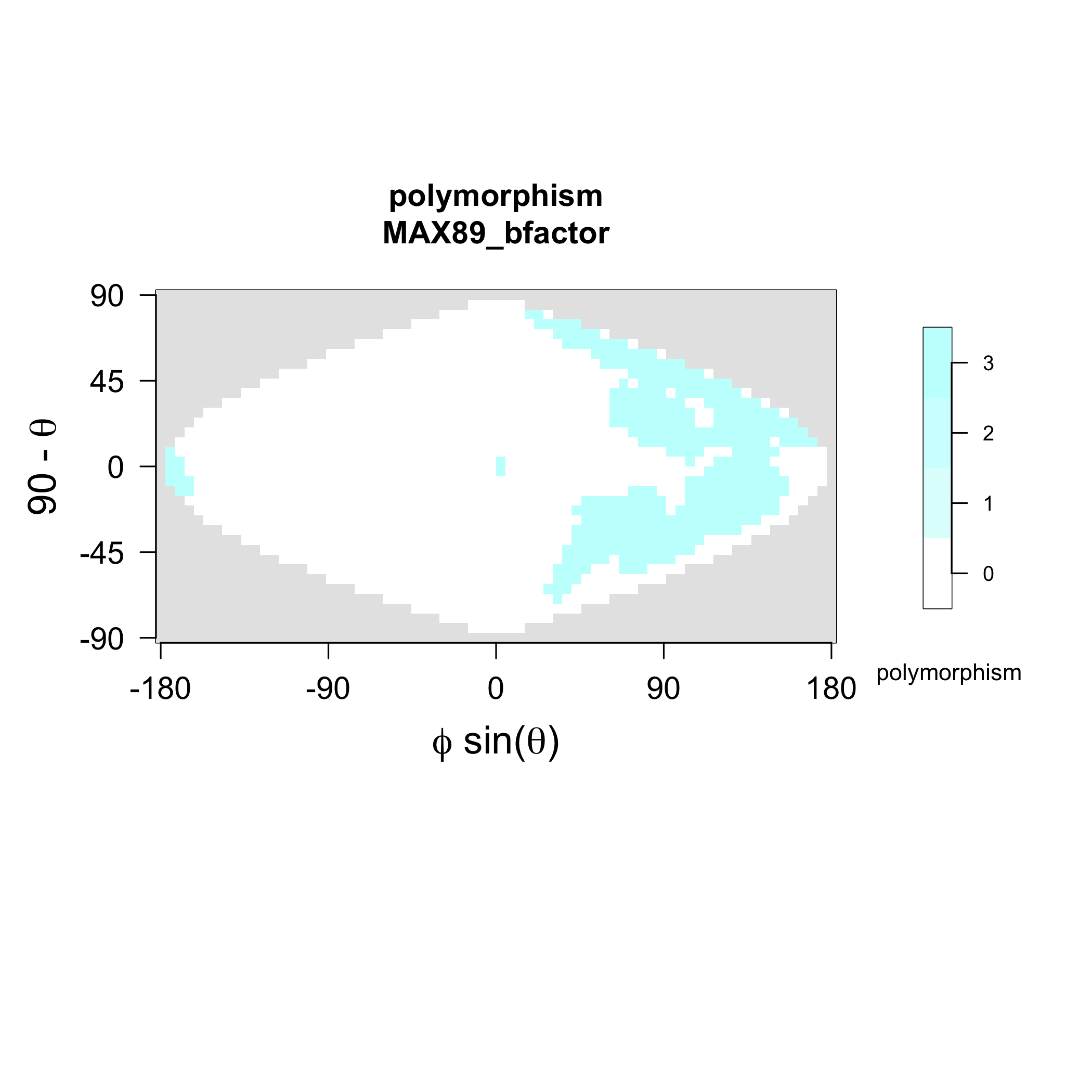

Supplement: S2 File — (ZIP) [file ppat.1012176.s019.zip › S2_File/POLYMORPHISM/MAX89_polymorphism.png]

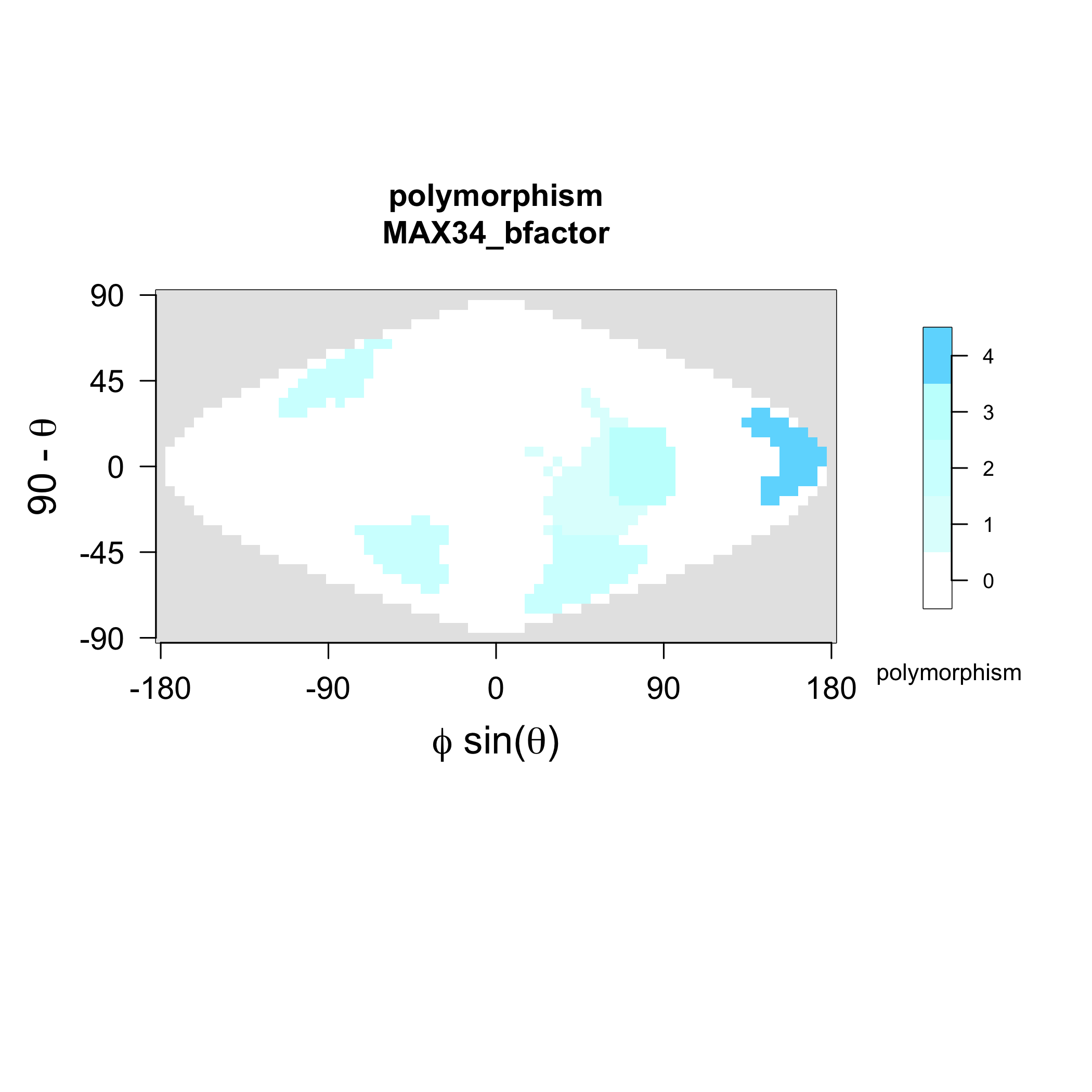

Supplement: S2 File — (ZIP) [file ppat.1012176.s019.zip › S2_File/POLYMORPHISM/MAX34_polymorphism.png]

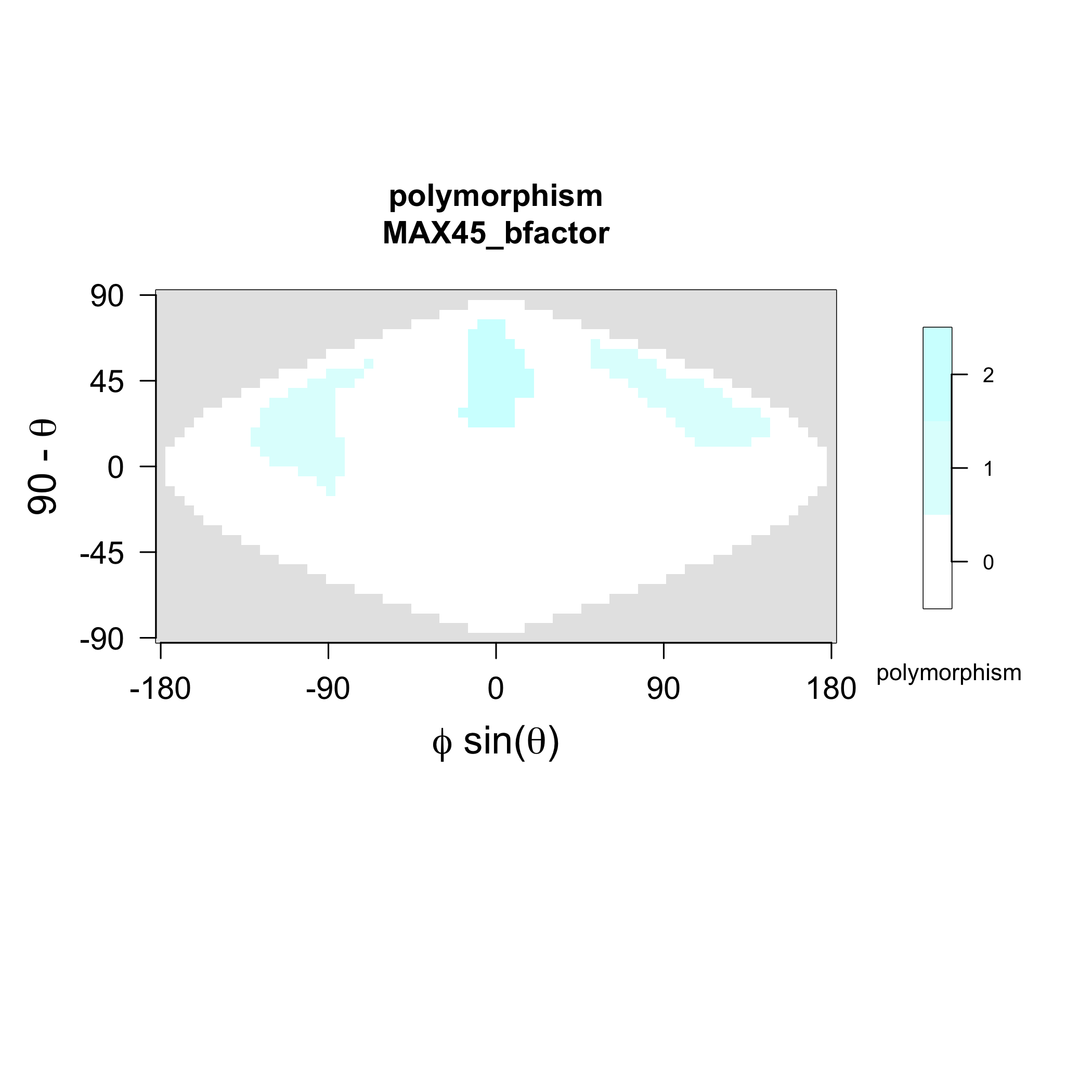

Supplement: S2 File — (ZIP) [file ppat.1012176.s019.zip › S2_File/POLYMORPHISM/MAX45_polymorphism.png]

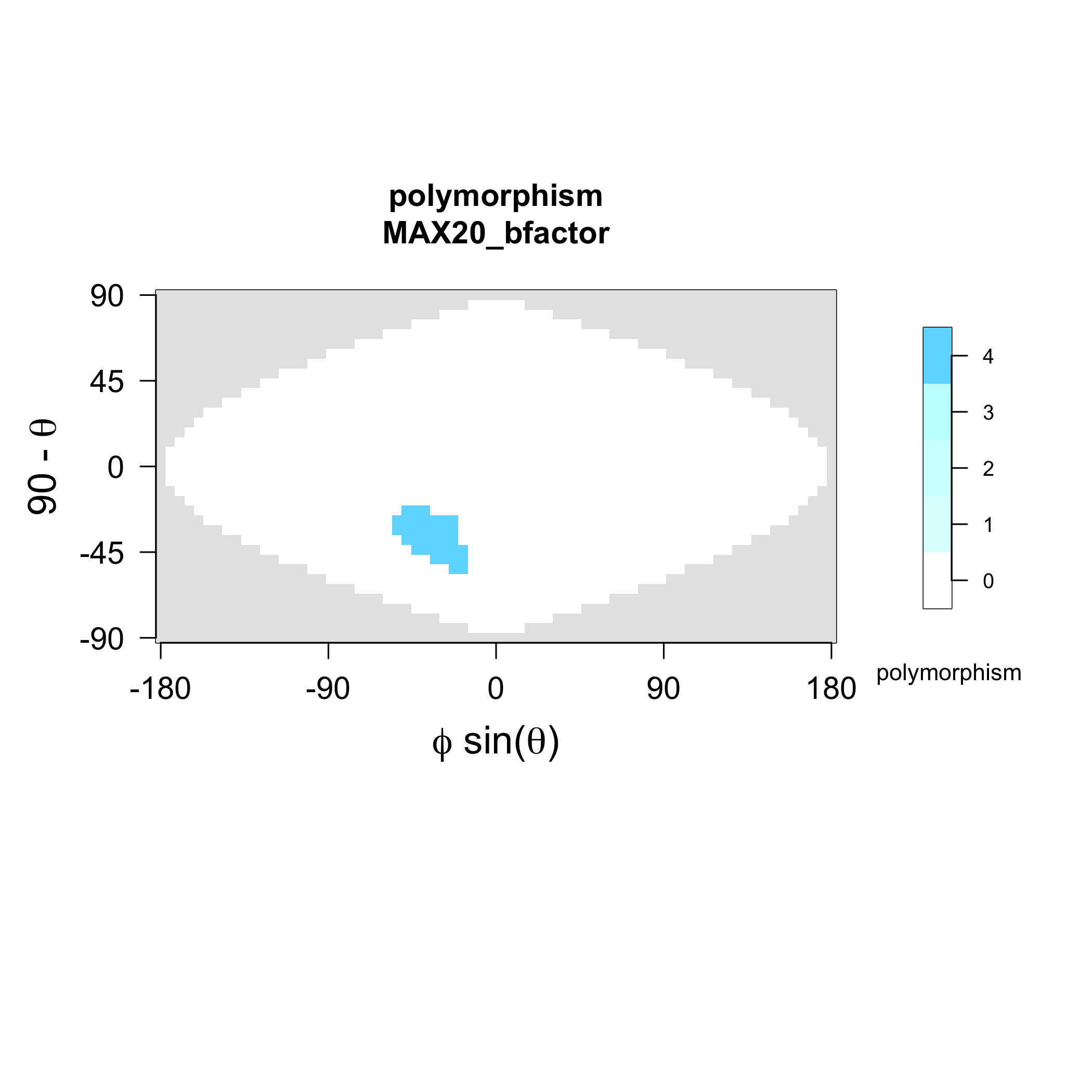

Supplement: S2 File — (ZIP) [file ppat.1012176.s019.zip › S2_File/POLYMORPHISM/MAX20_polymorphism.png]

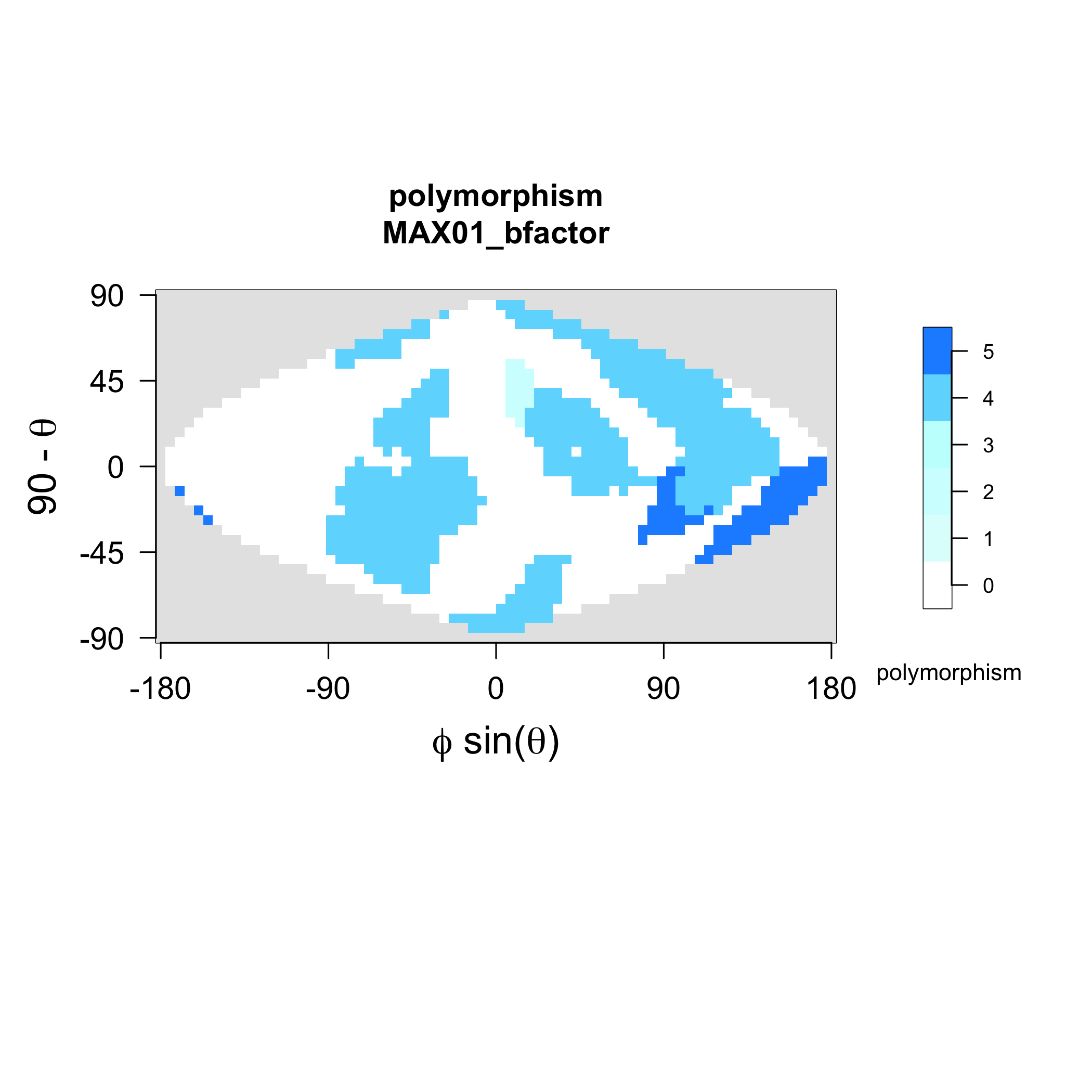

Supplement: S2 File — (ZIP) [file ppat.1012176.s019.zip › S2_File/POLYMORPHISM/MAX01_polymorphism.png]

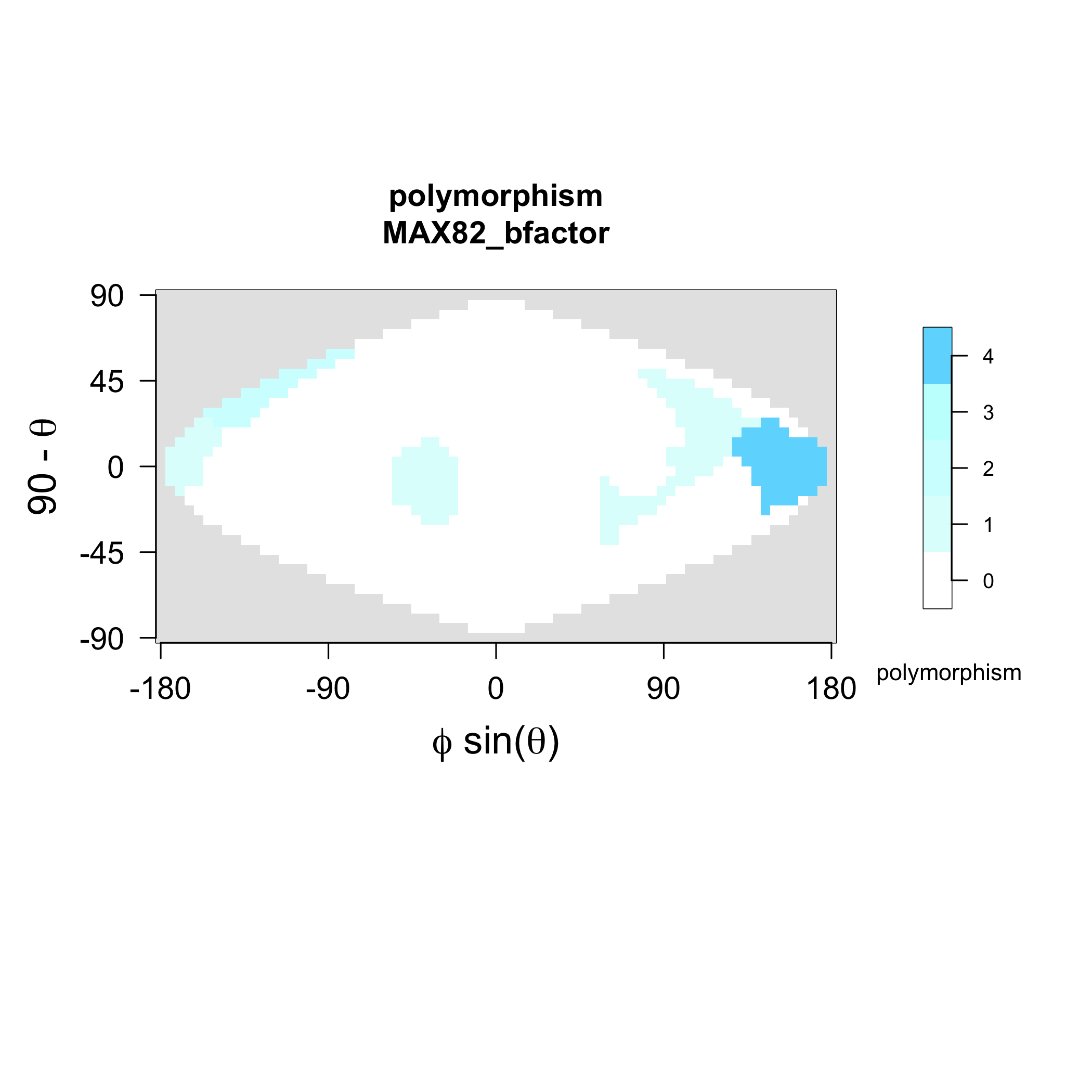

Supplement: S2 File — (ZIP) [file ppat.1012176.s019.zip › S2_File/POLYMORPHISM/MAX82_polymorphism.png]

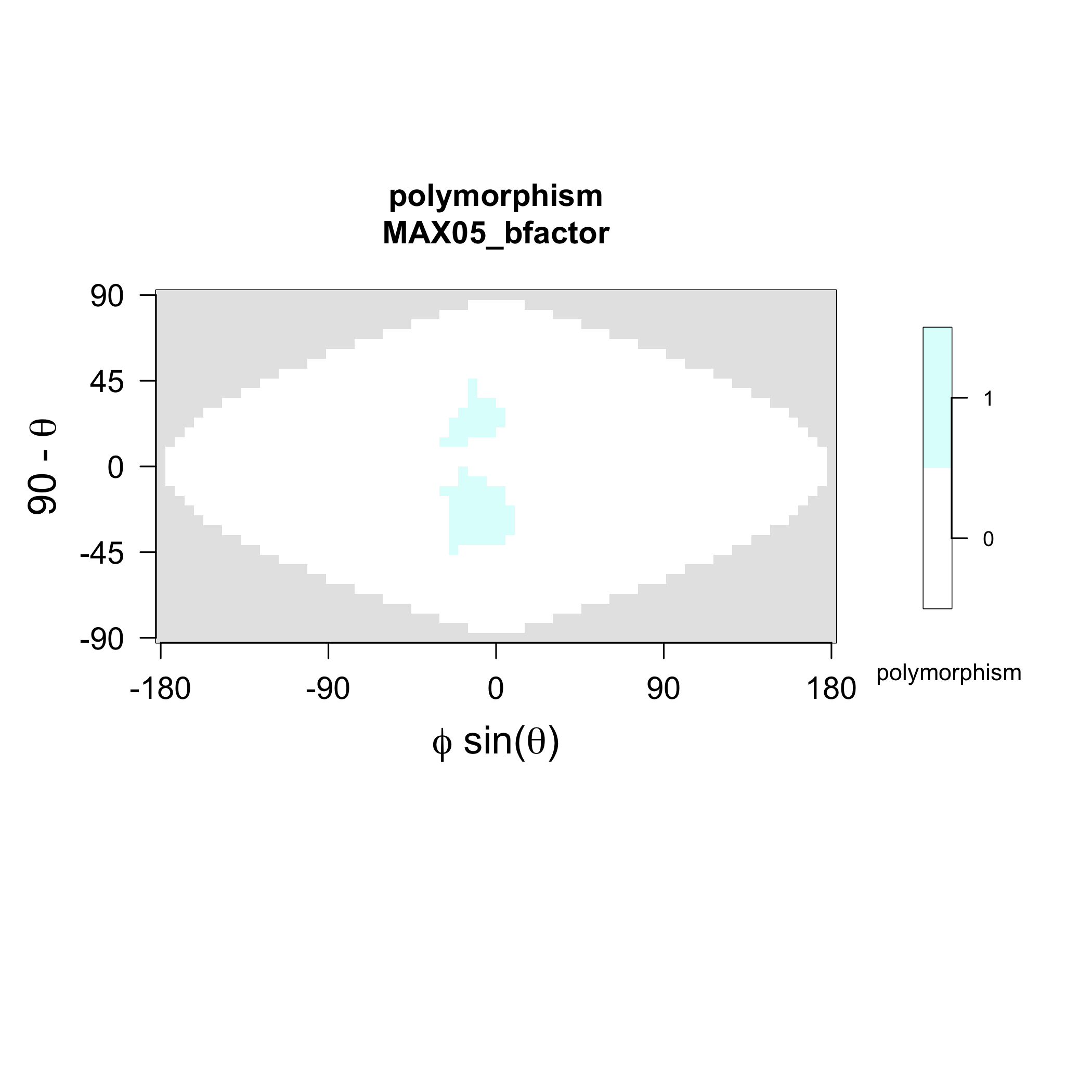

Supplement: S2 File — (ZIP) [file ppat.1012176.s019.zip › S2_File/POLYMORPHISM/MAX05_polymorphism.png]

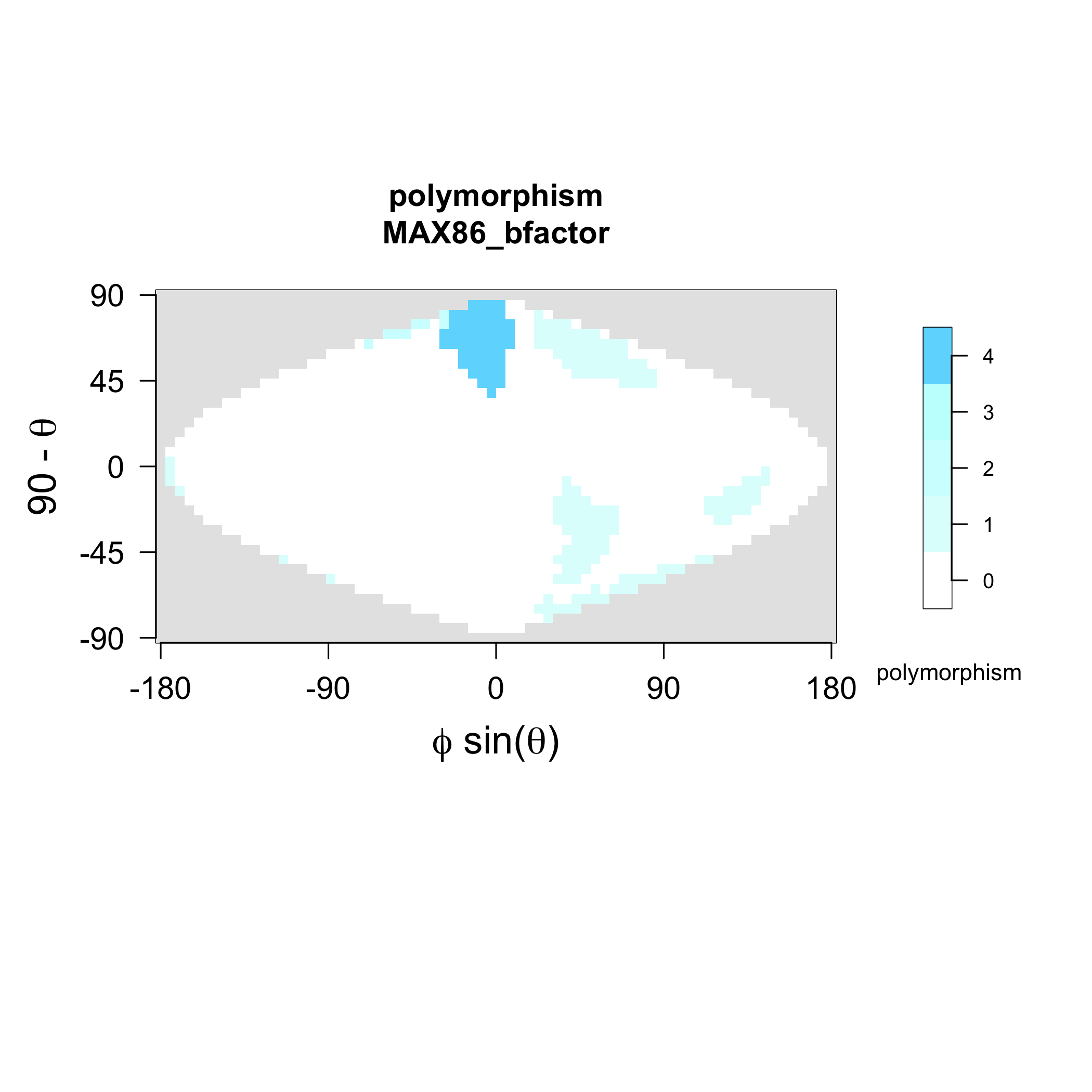

Supplement: S2 File — (ZIP) [file ppat.1012176.s019.zip › S2_File/POLYMORPHISM/MAX86_polymorphism.png]

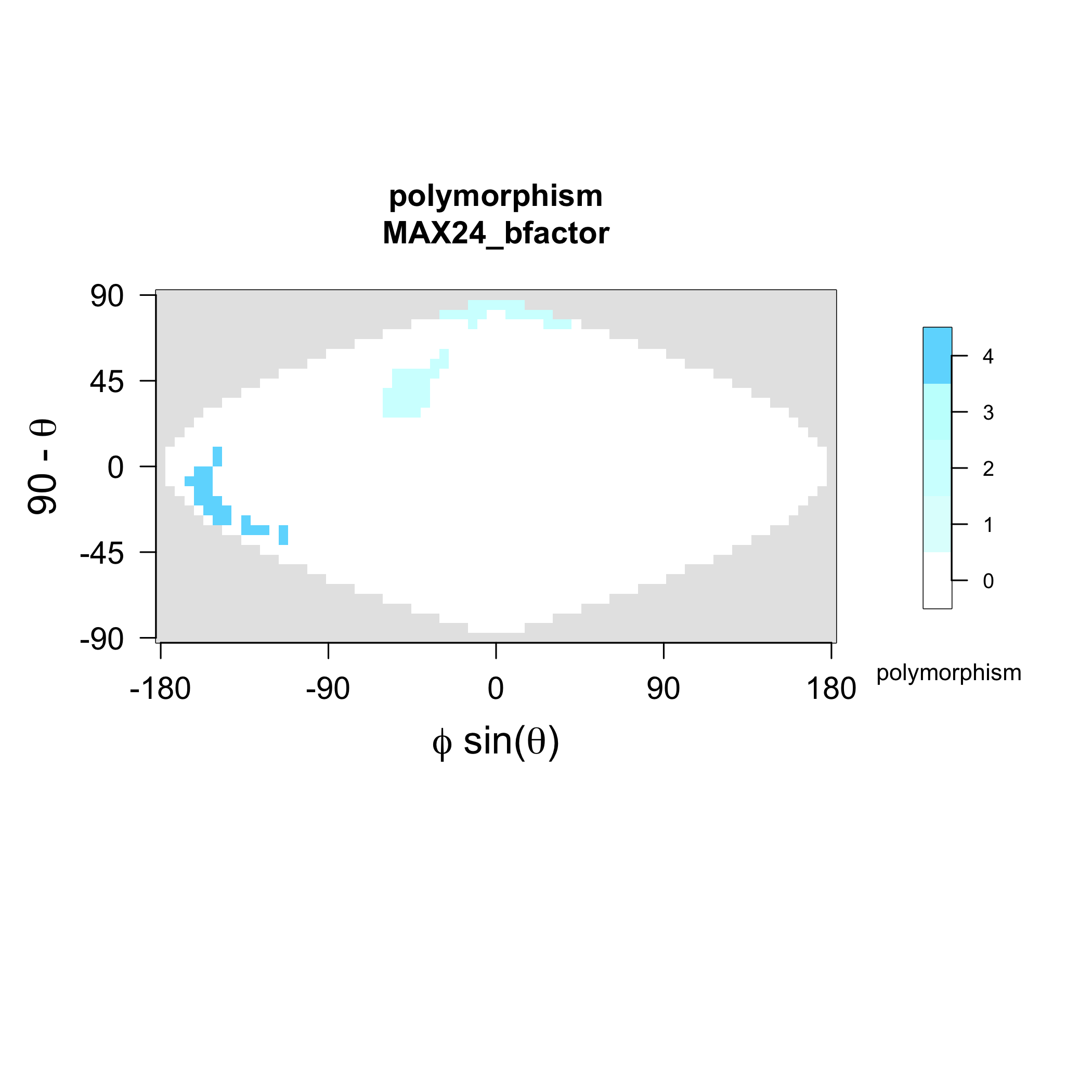

Supplement: S2 File — (ZIP) [file ppat.1012176.s019.zip › S2_File/POLYMORPHISM/MAX24_polymorphism.png]

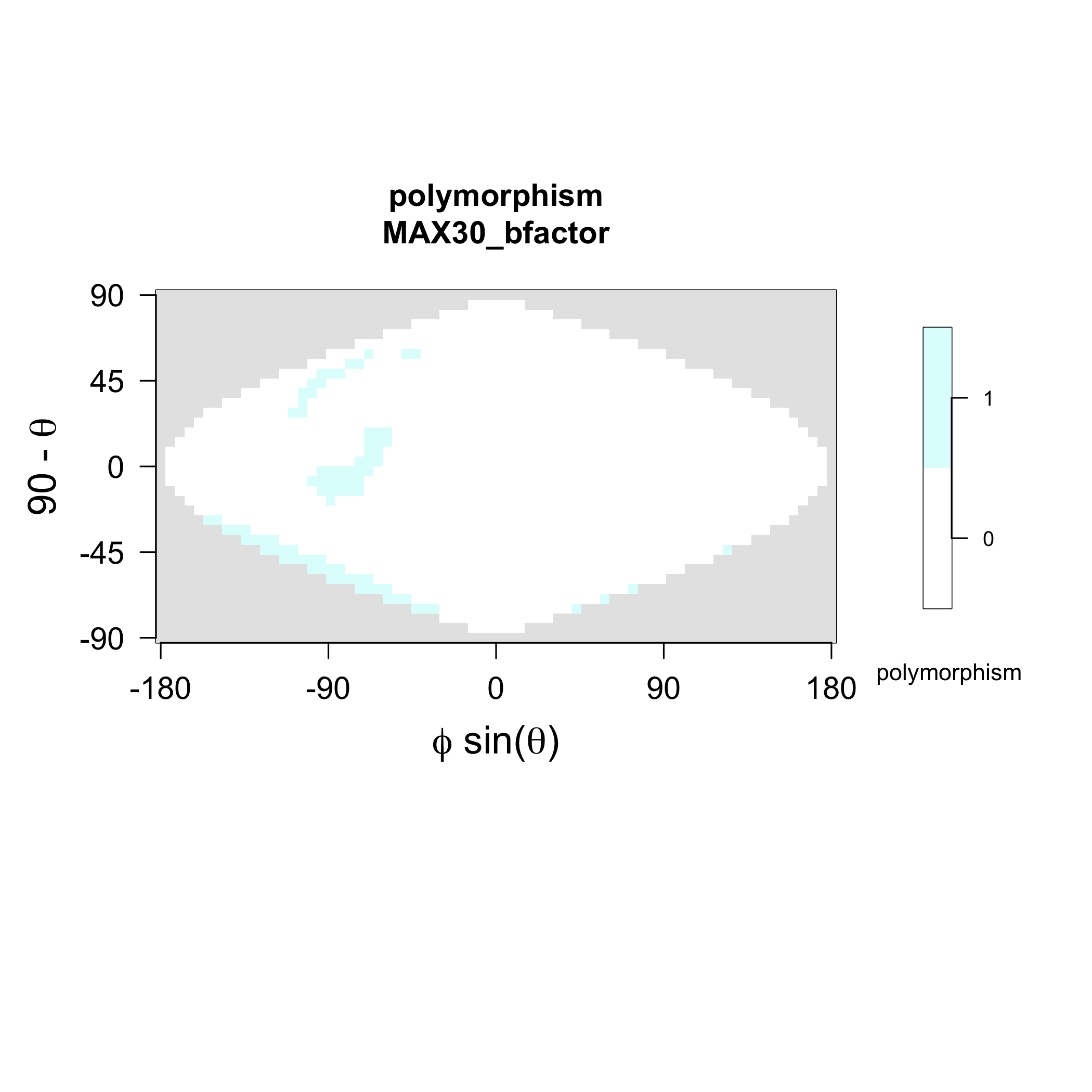

Supplement: S2 File — (ZIP) [file ppat.1012176.s019.zip › S2_File/POLYMORPHISM/MAX30_polymorphism.png]

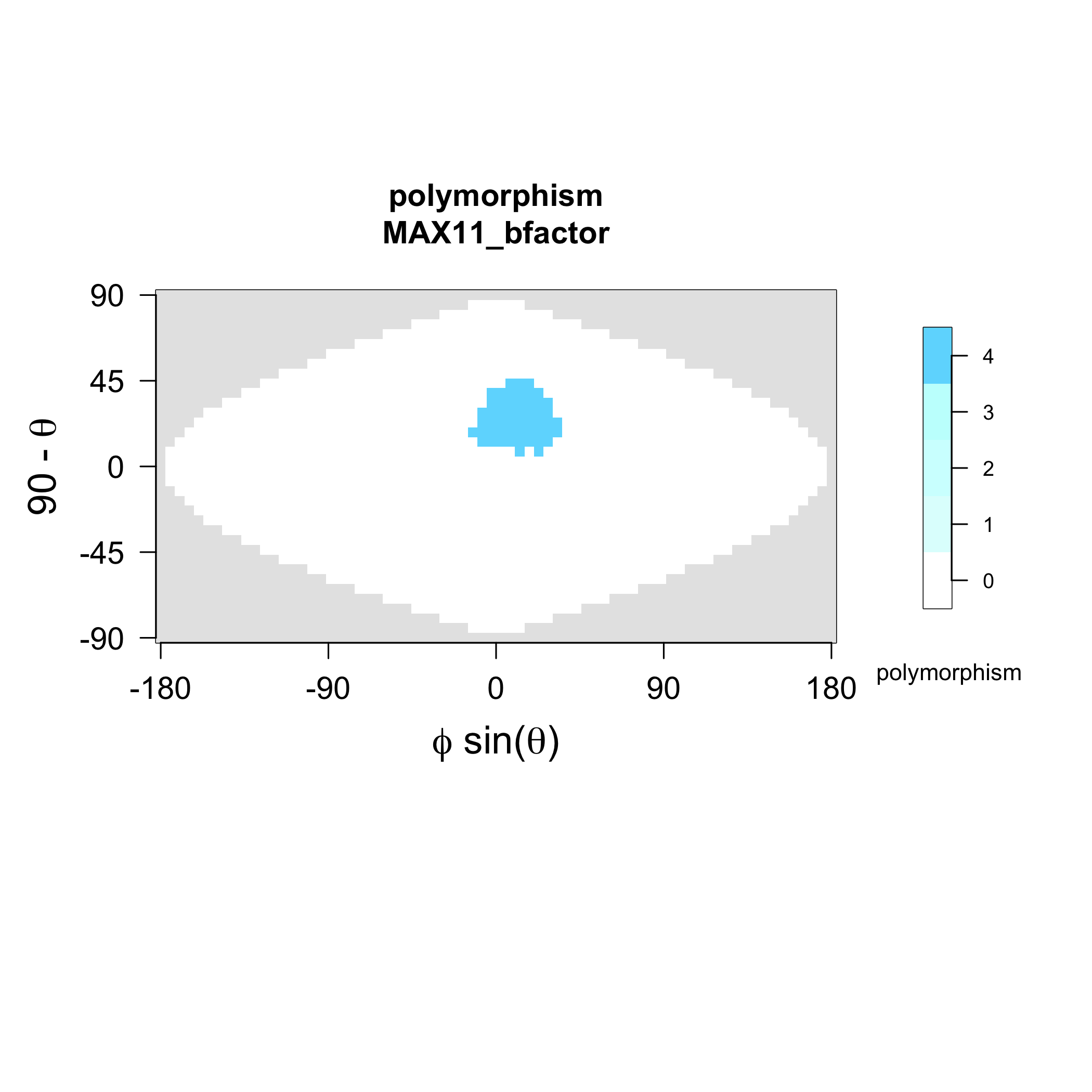

Supplement: S2 File — (ZIP) [file ppat.1012176.s019.zip › S2_File/POLYMORPHISM/MAX11_polymorphism.png]

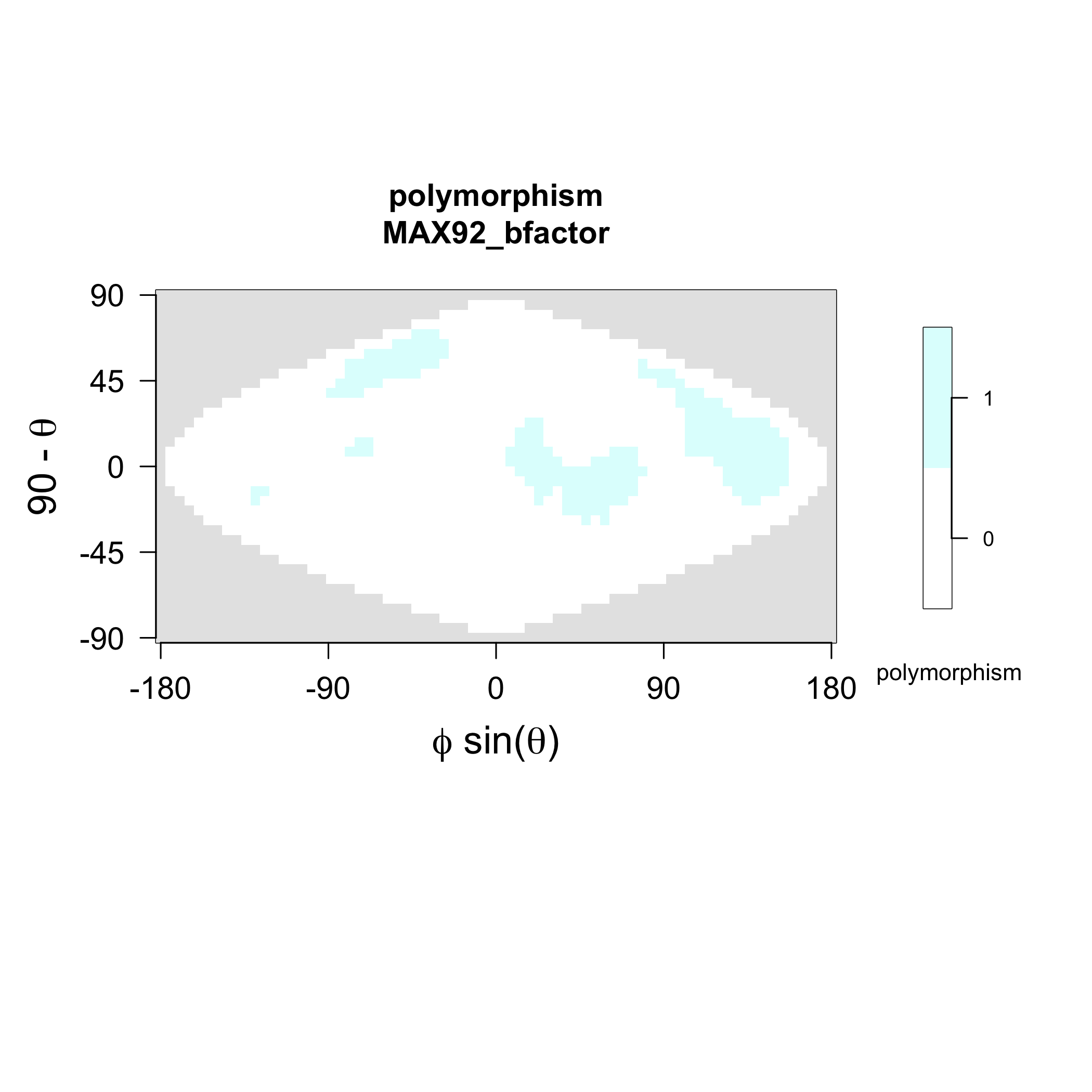

Supplement: S2 File — (ZIP) [file ppat.1012176.s019.zip › S2_File/POLYMORPHISM/MAX92_polymorphism.png]

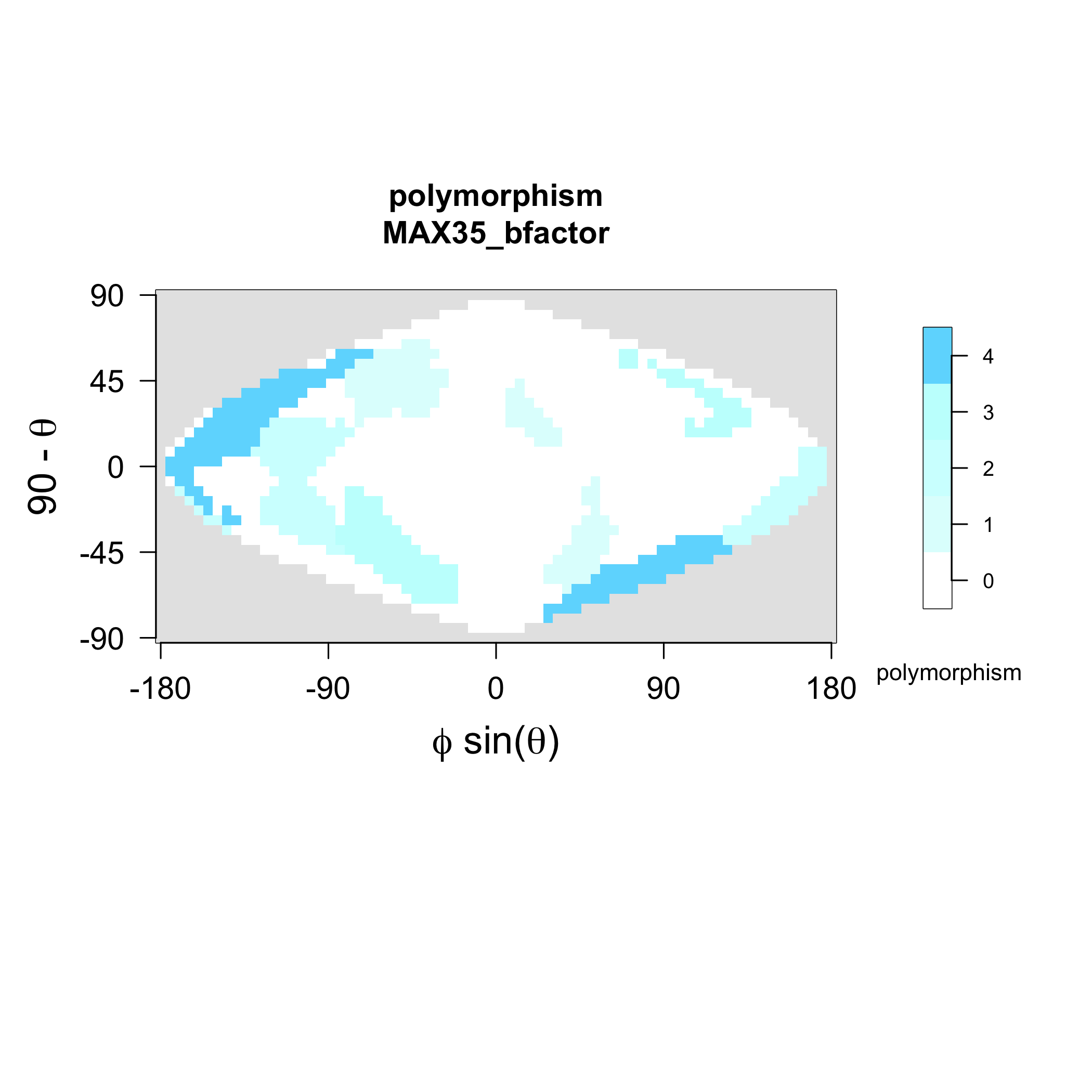

Supplement: S2 File — (ZIP) [file ppat.1012176.s019.zip › S2_File/POLYMORPHISM/MAX35_polymorphism.png]

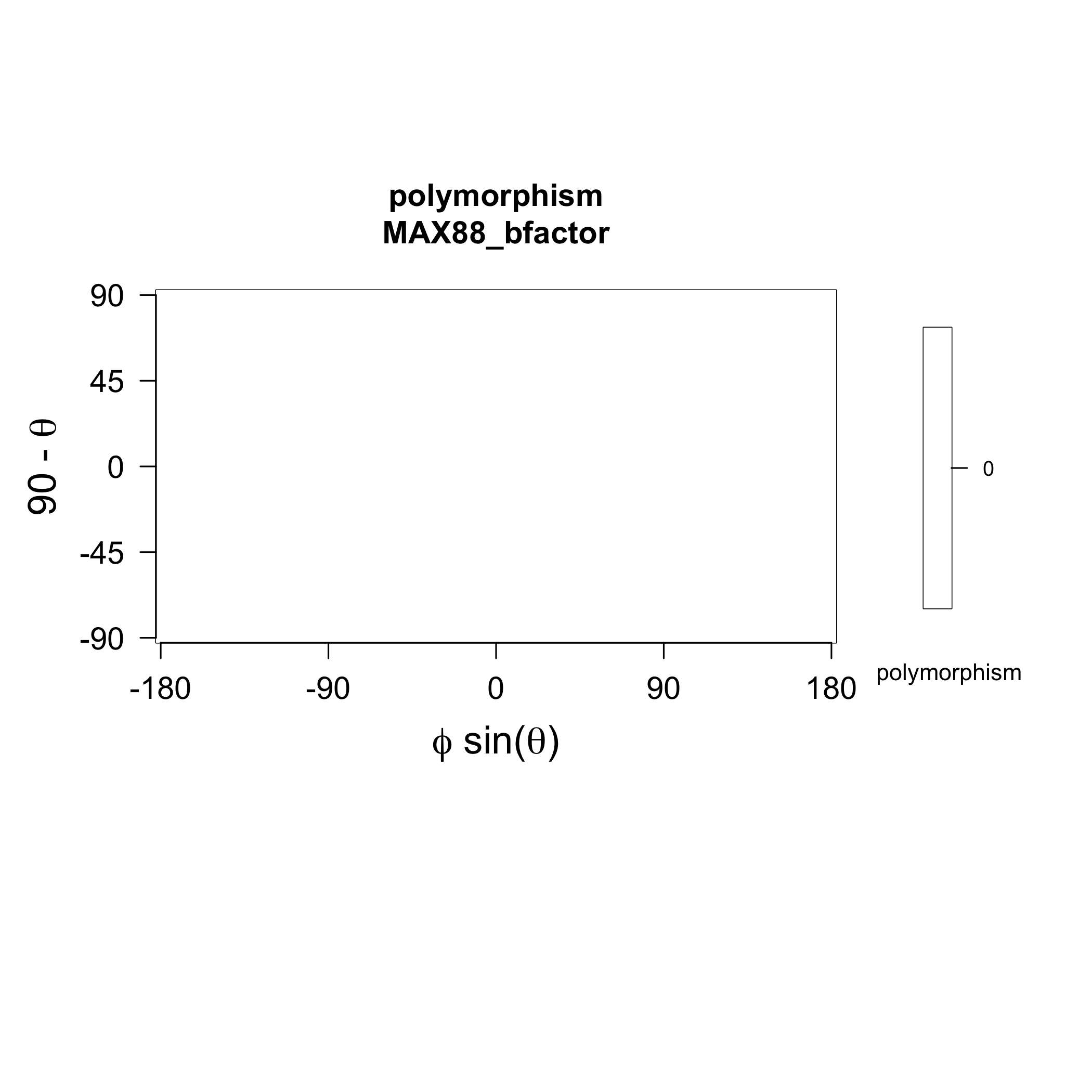

Supplement: S2 File — (ZIP) [file ppat.1012176.s019.zip › S2_File/POLYMORPHISM/MAX88_polymorphism.png]

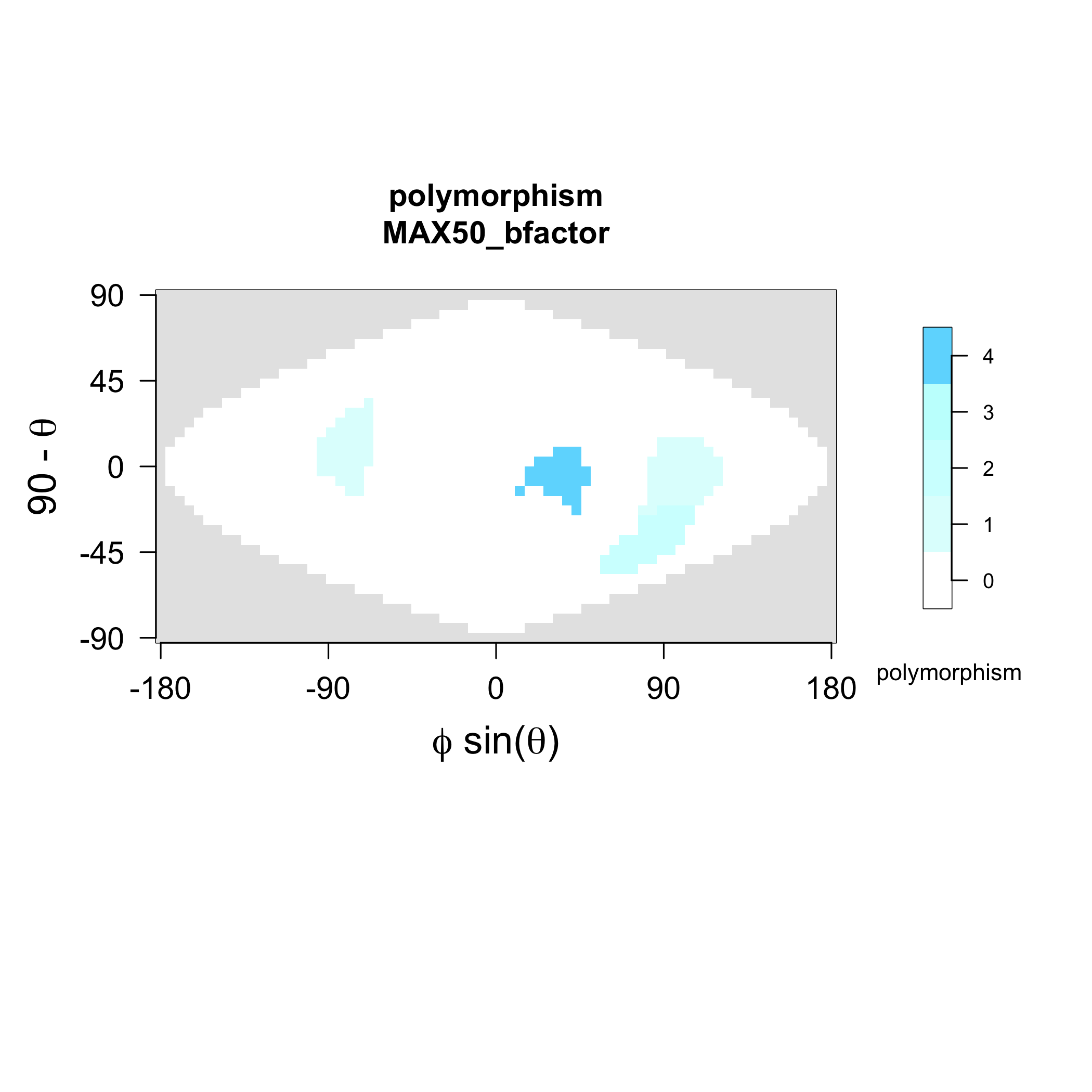

Supplement: S2 File — (ZIP) [file ppat.1012176.s019.zip › S2_File/POLYMORPHISM/MAX50_polymorphism.png]

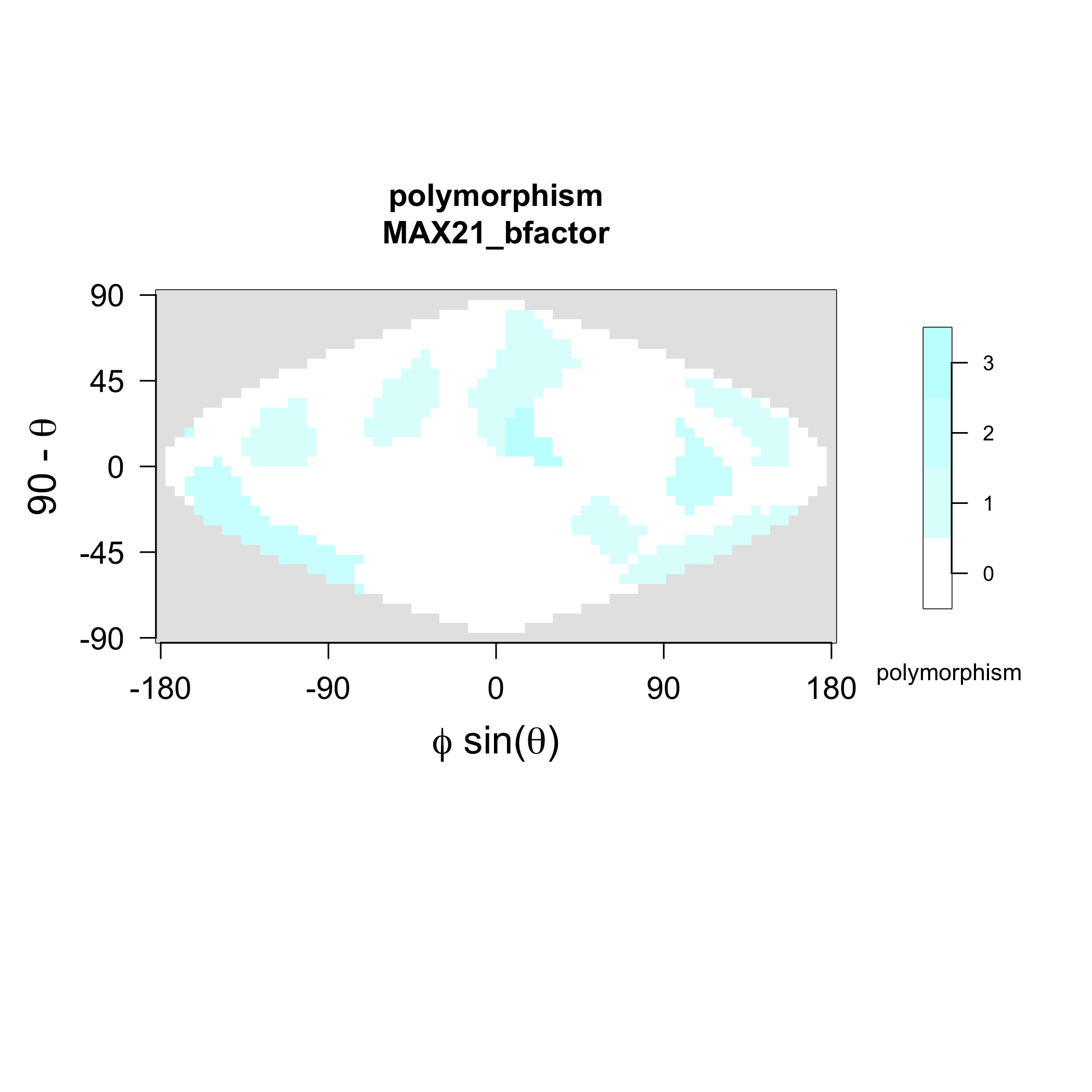

Supplement: S2 File — (ZIP) [file ppat.1012176.s019.zip › S2_File/POLYMORPHISM/MAX21_polymorphism.png]

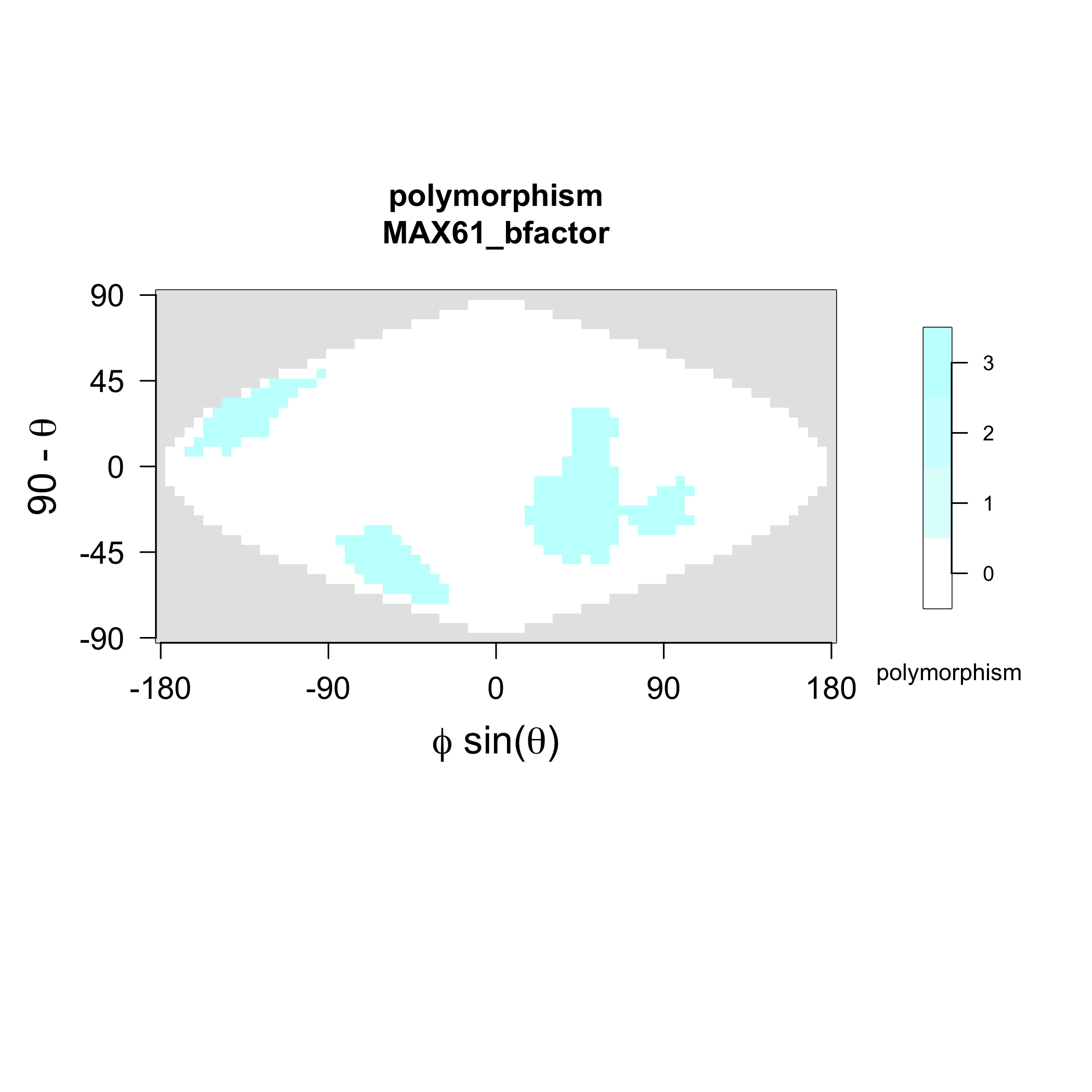

Supplement: S2 File — (ZIP) [file ppat.1012176.s019.zip › S2_File/POLYMORPHISM/MAX61_polymorphism.png]

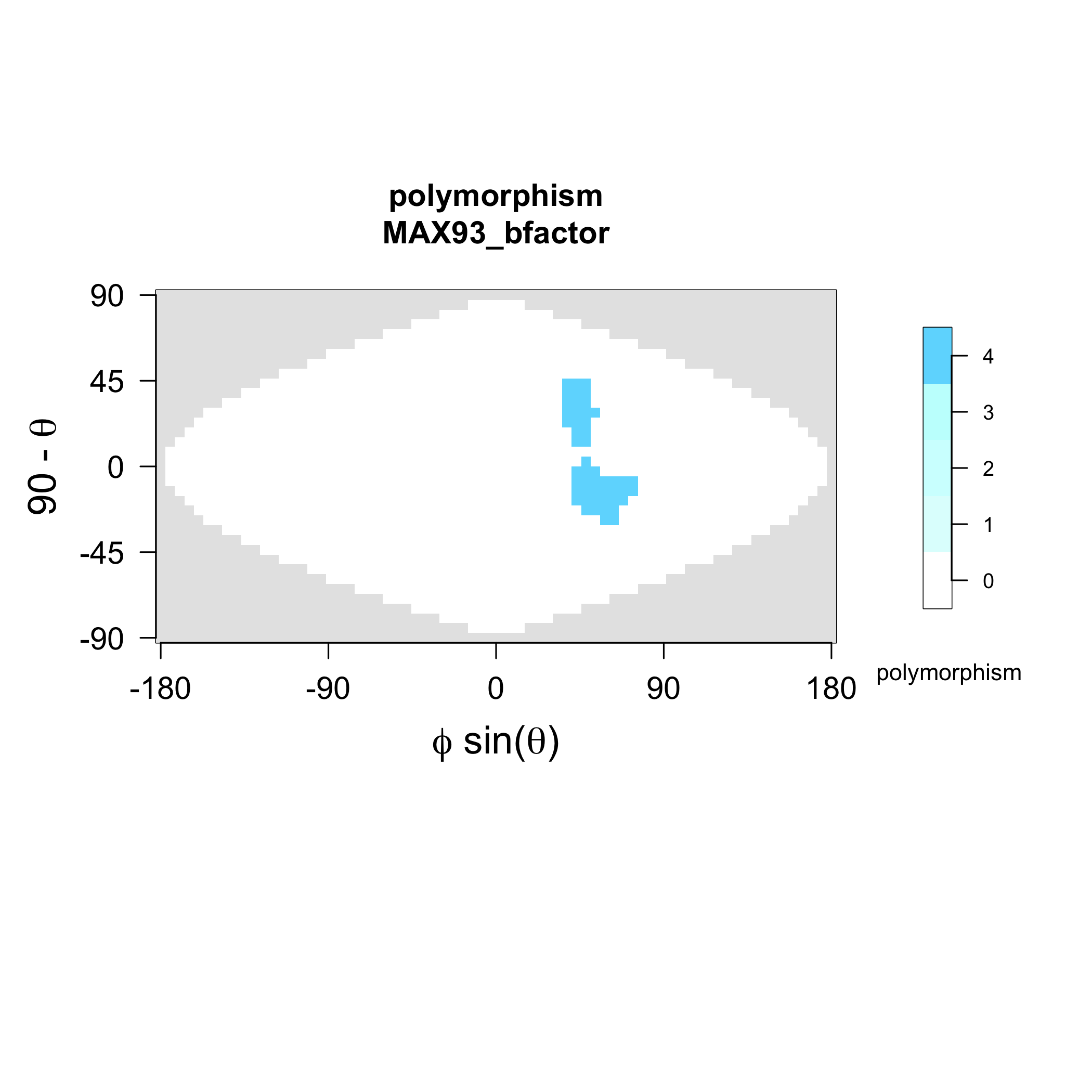

Supplement: S2 File — (ZIP) [file ppat.1012176.s019.zip › S2_File/POLYMORPHISM/MAX93_polymorphism.png]

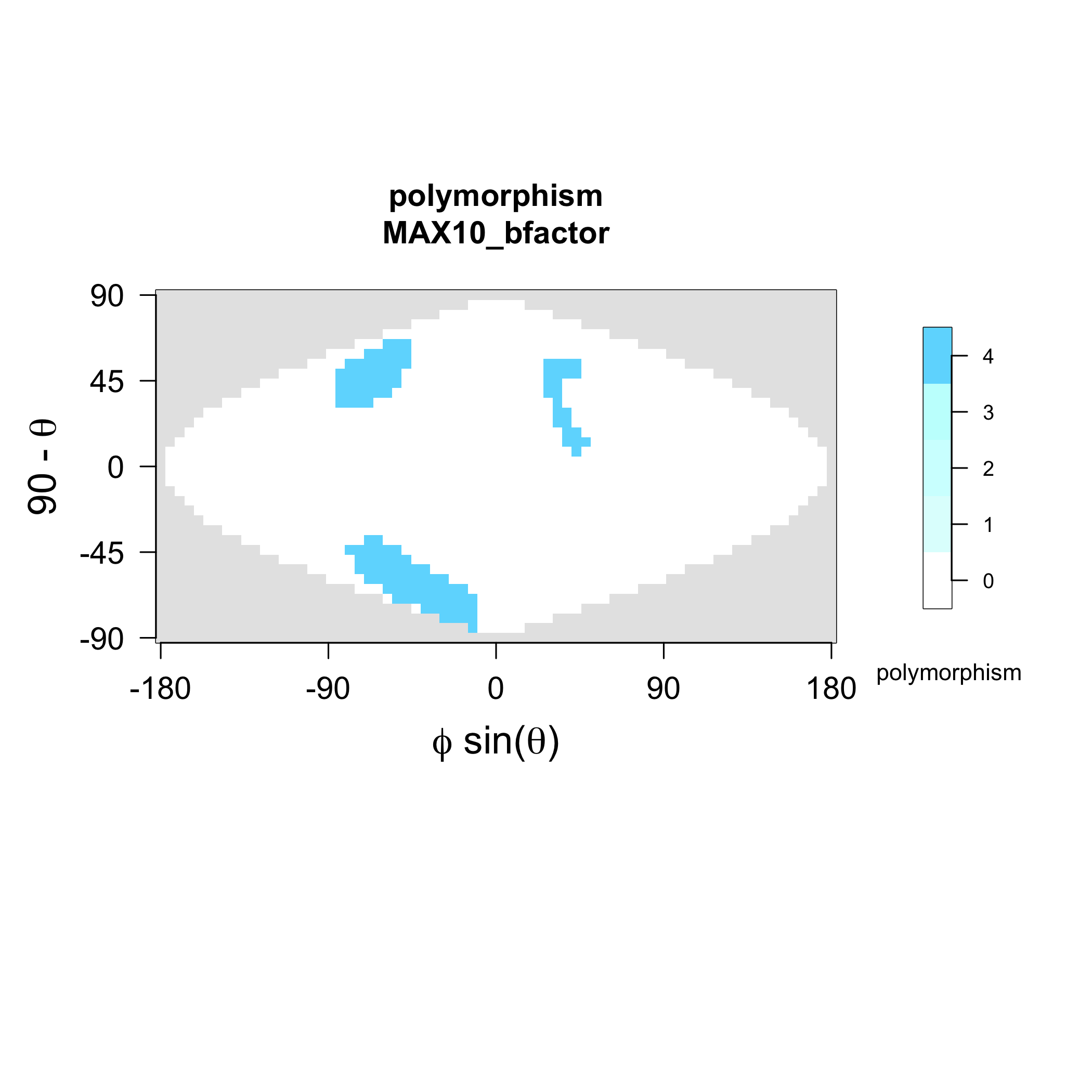

Supplement: S2 File — (ZIP) [file ppat.1012176.s019.zip › S2_File/POLYMORPHISM/MAX10_polymorphism.png]

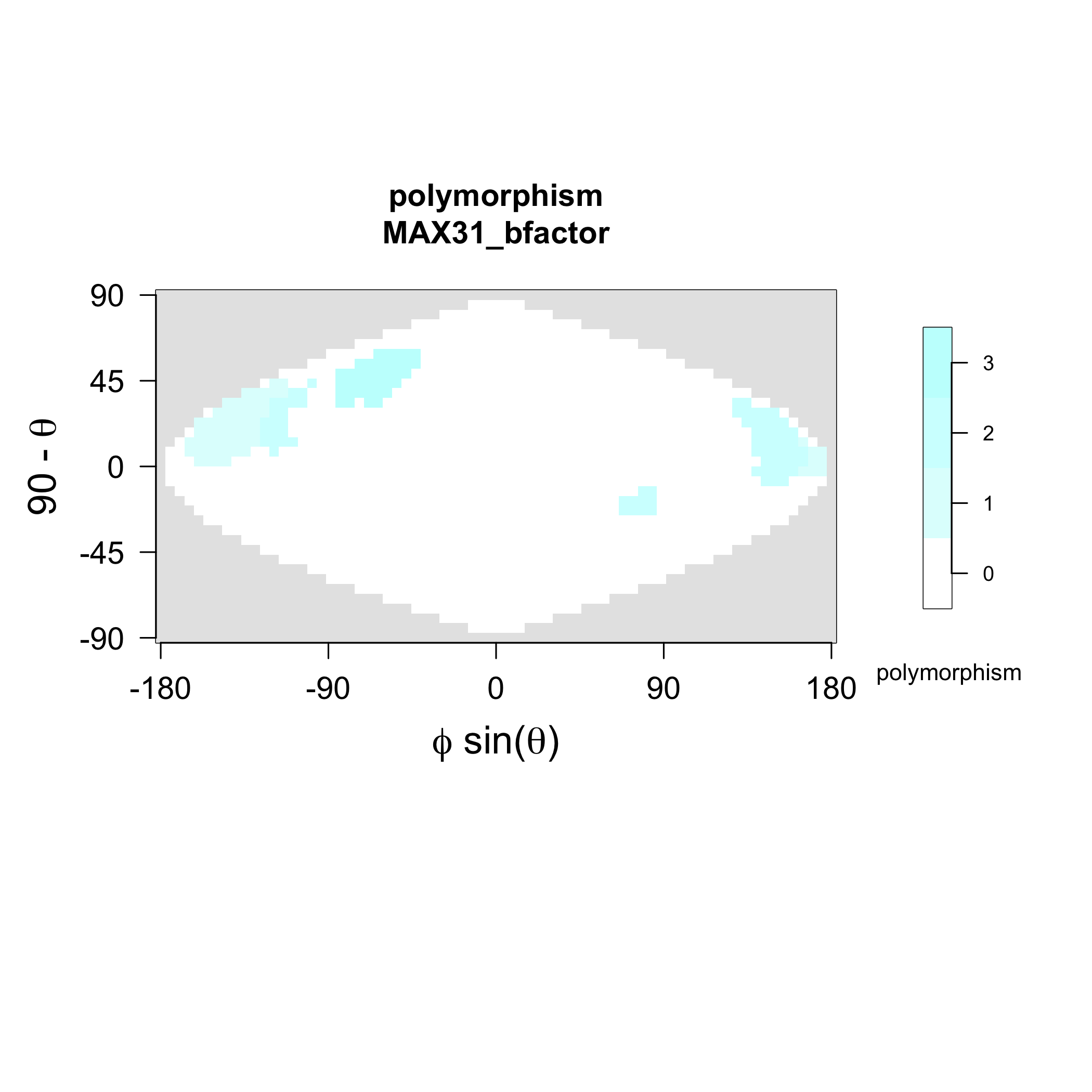

Supplement: S2 File — (ZIP) [file ppat.1012176.s019.zip › S2_File/POLYMORPHISM/MAX31_polymorphism.png]

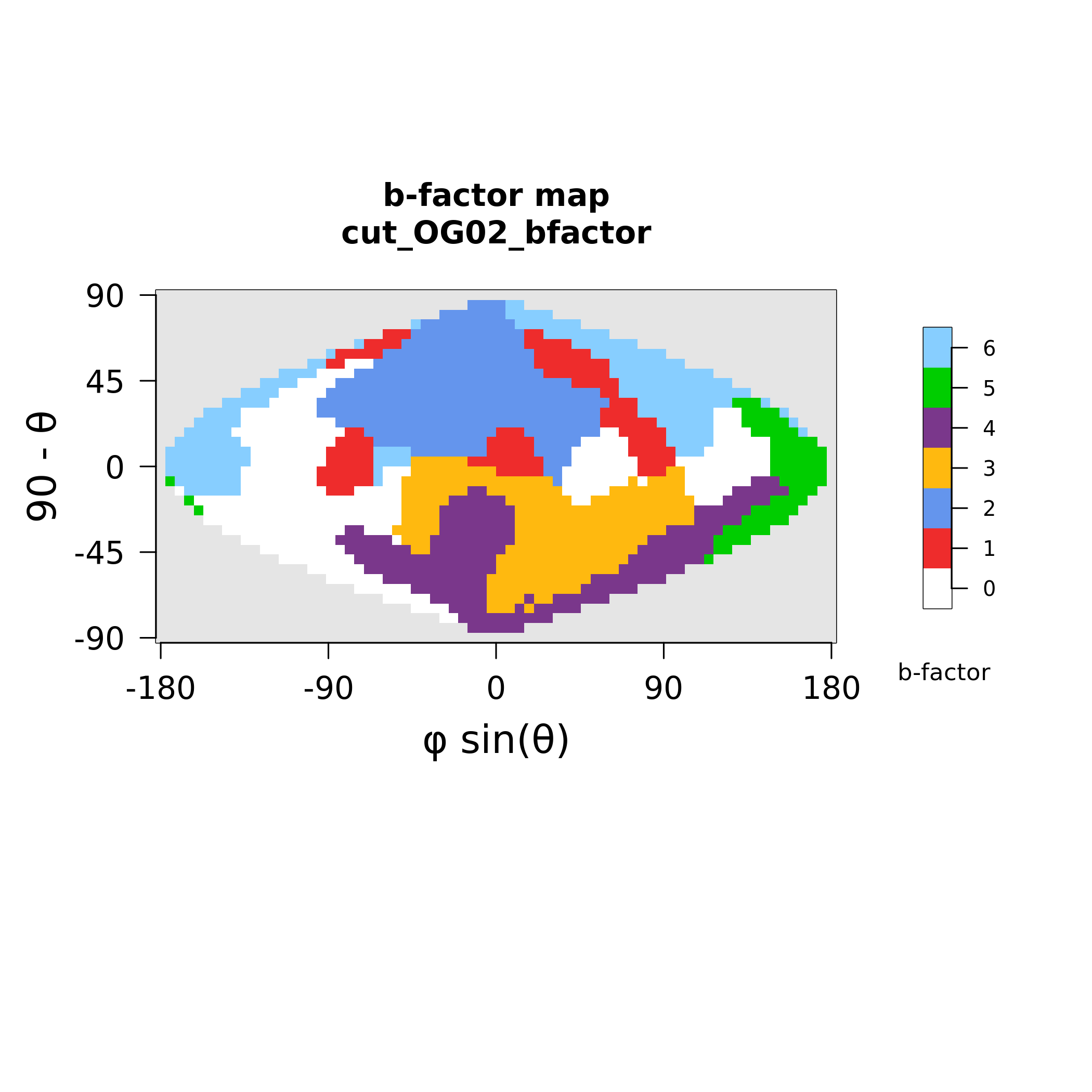

Supplement: S2 File — (ZIP) [file ppat.1012176.s019.zip › S2_File/STRANDS/MAX02_strands.png]

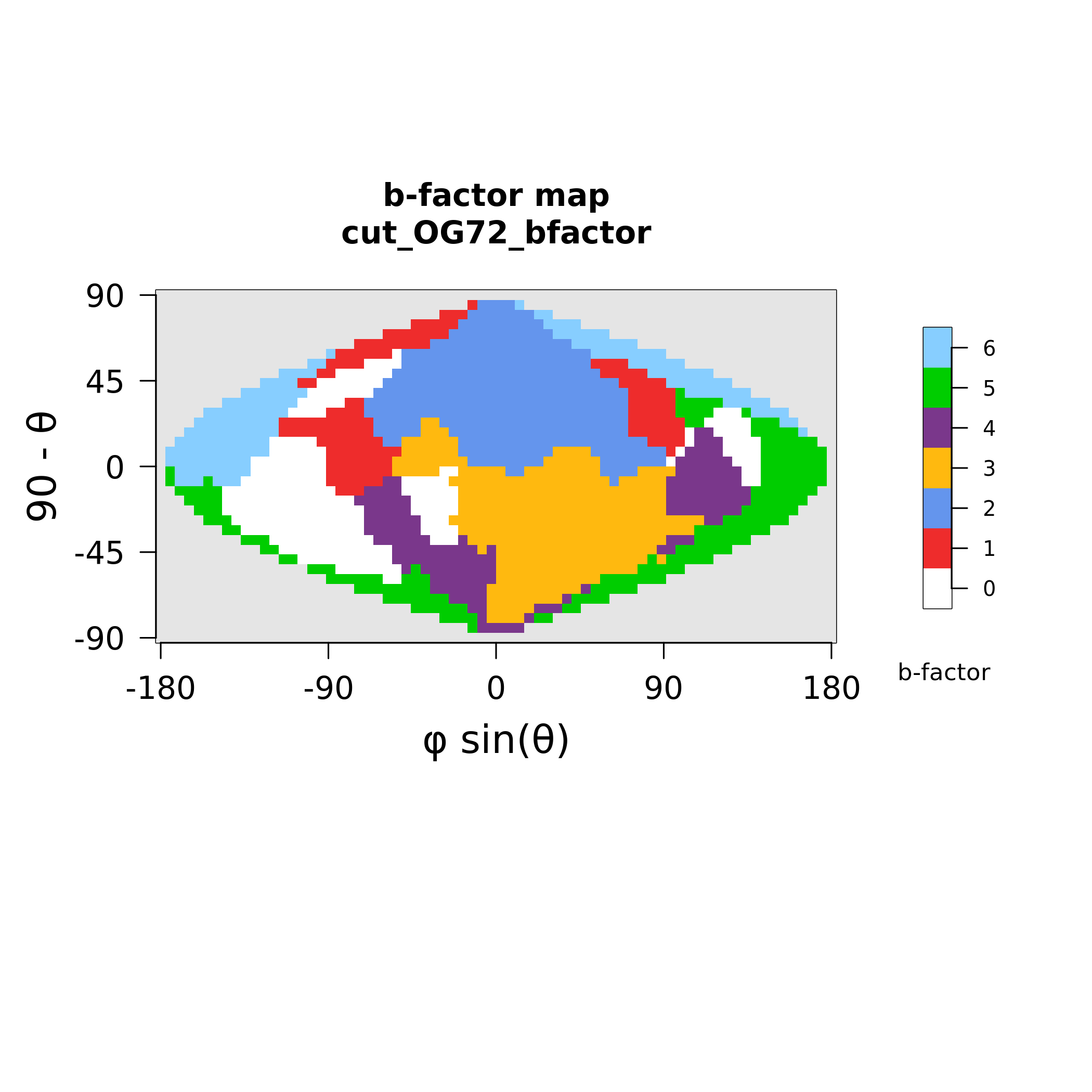

Supplement: S2 File — (ZIP) [file ppat.1012176.s019.zip › S2_File/STRANDS/MAX72_strands.png]

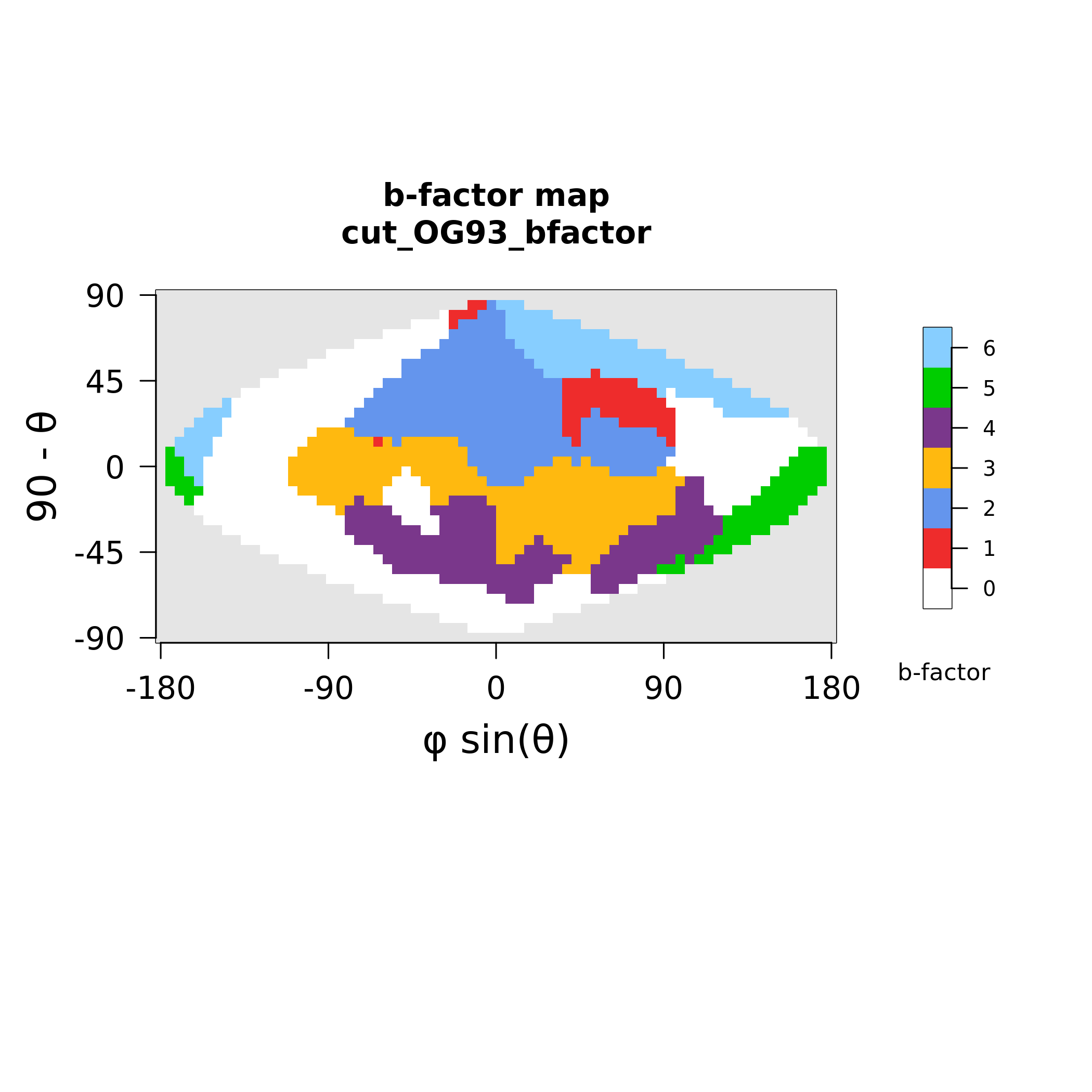

Supplement: S2 File — (ZIP) [file ppat.1012176.s019.zip › S2_File/STRANDS/MAX93_strands.png]

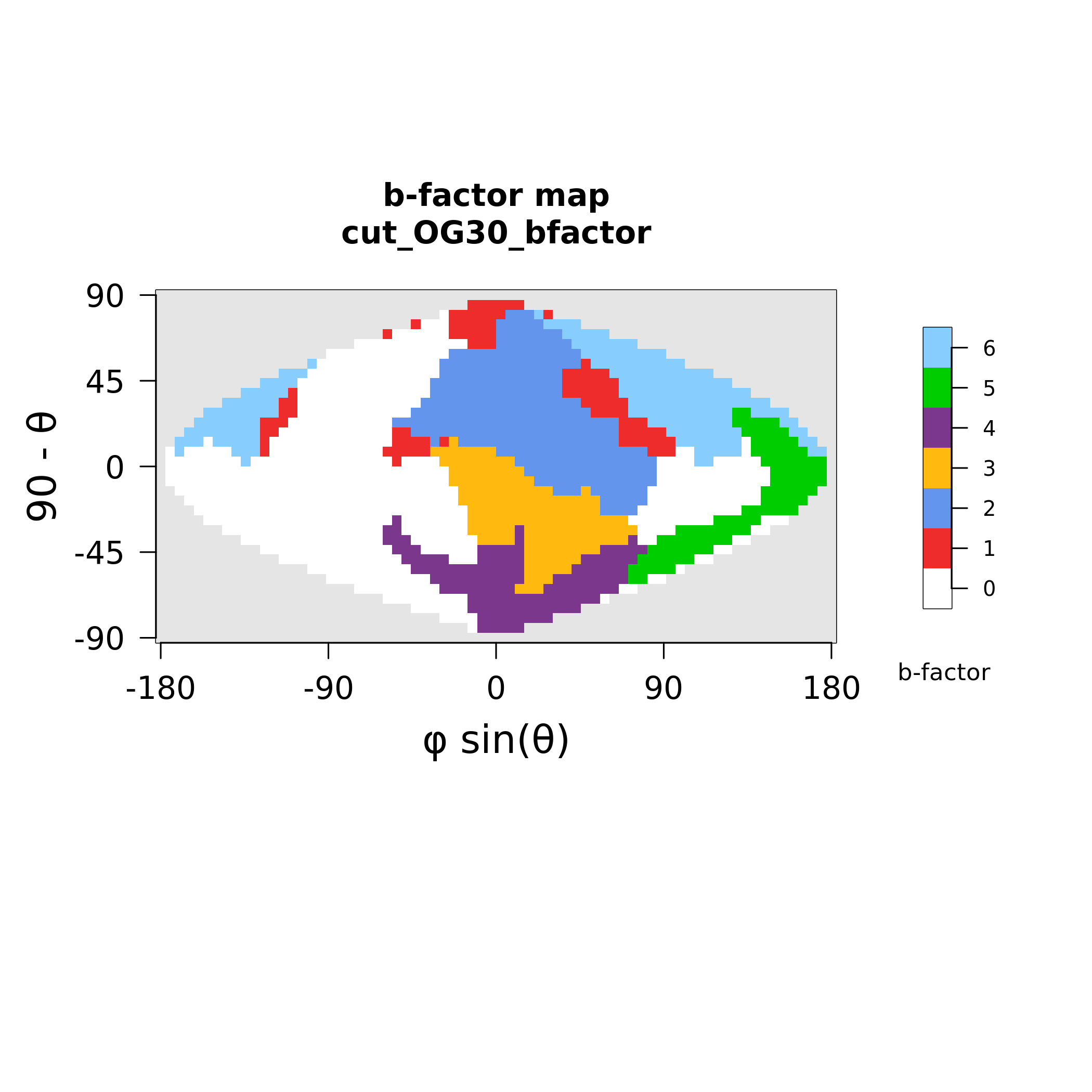

Supplement: S2 File — (ZIP) [file ppat.1012176.s019.zip › S2_File/STRANDS/MAX30_strands.png]

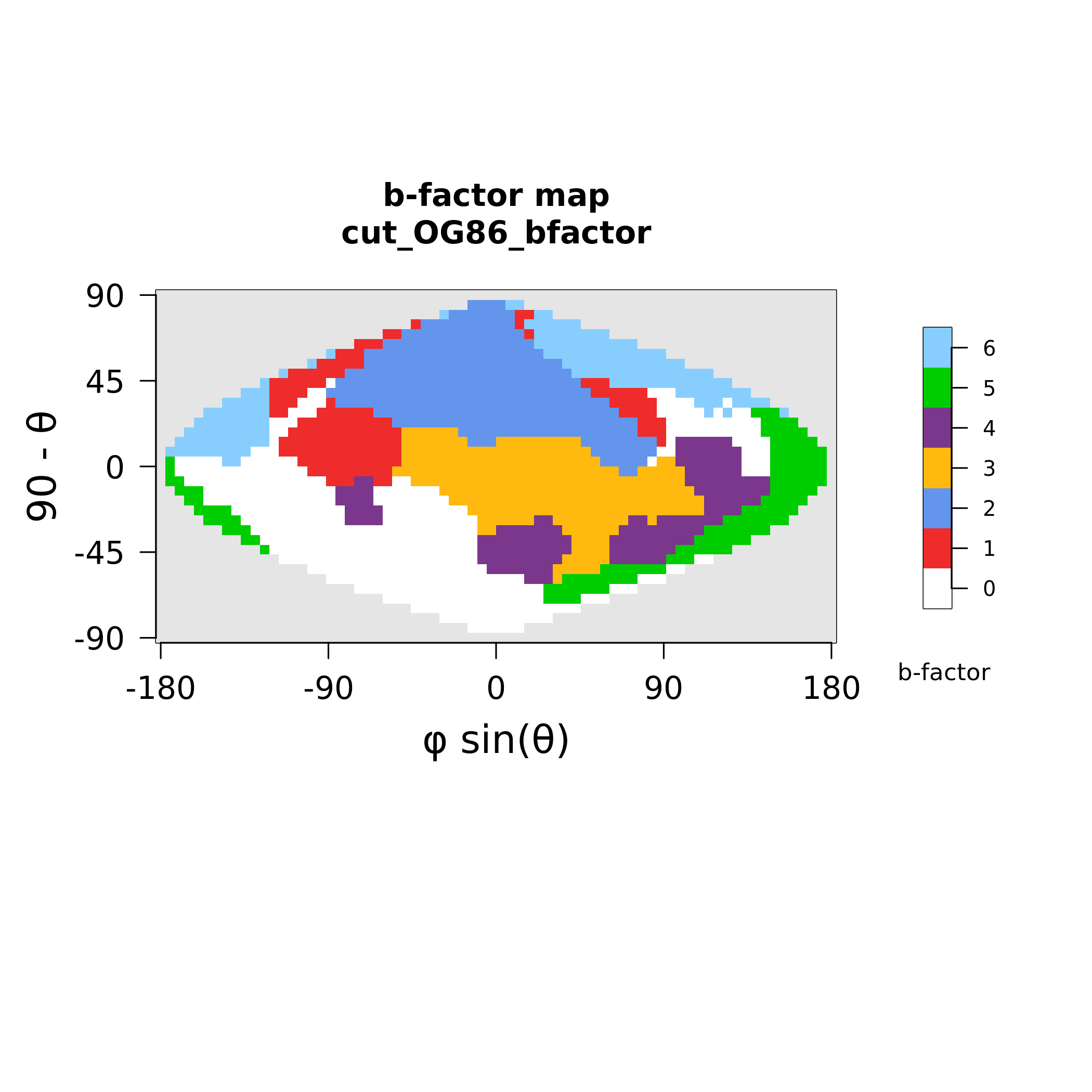

Supplement: S2 File — (ZIP) [file ppat.1012176.s019.zip › S2_File/STRANDS/MAX86_strands.png]

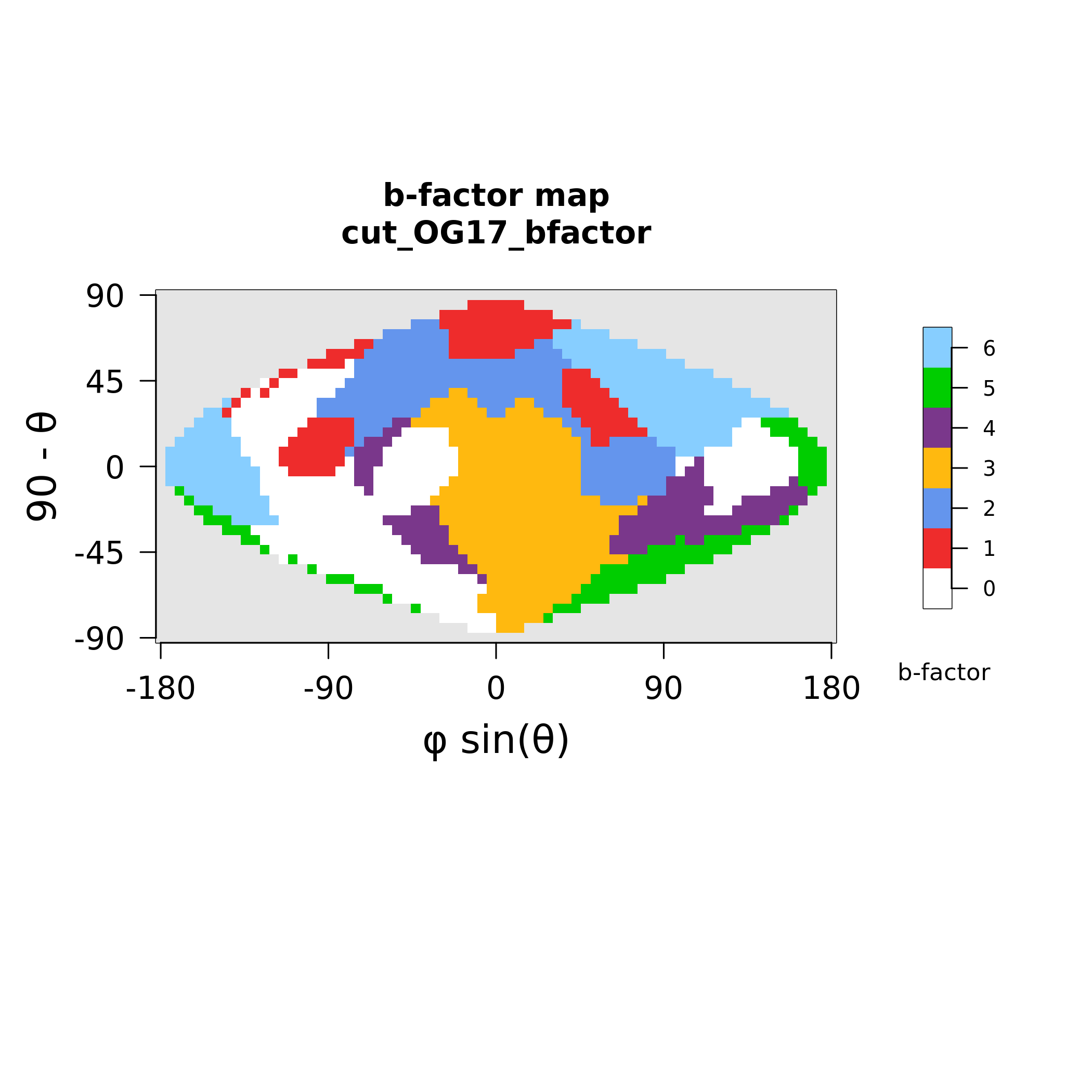

Supplement: S2 File — (ZIP) [file ppat.1012176.s019.zip › S2_File/STRANDS/MAX17_strands.png]

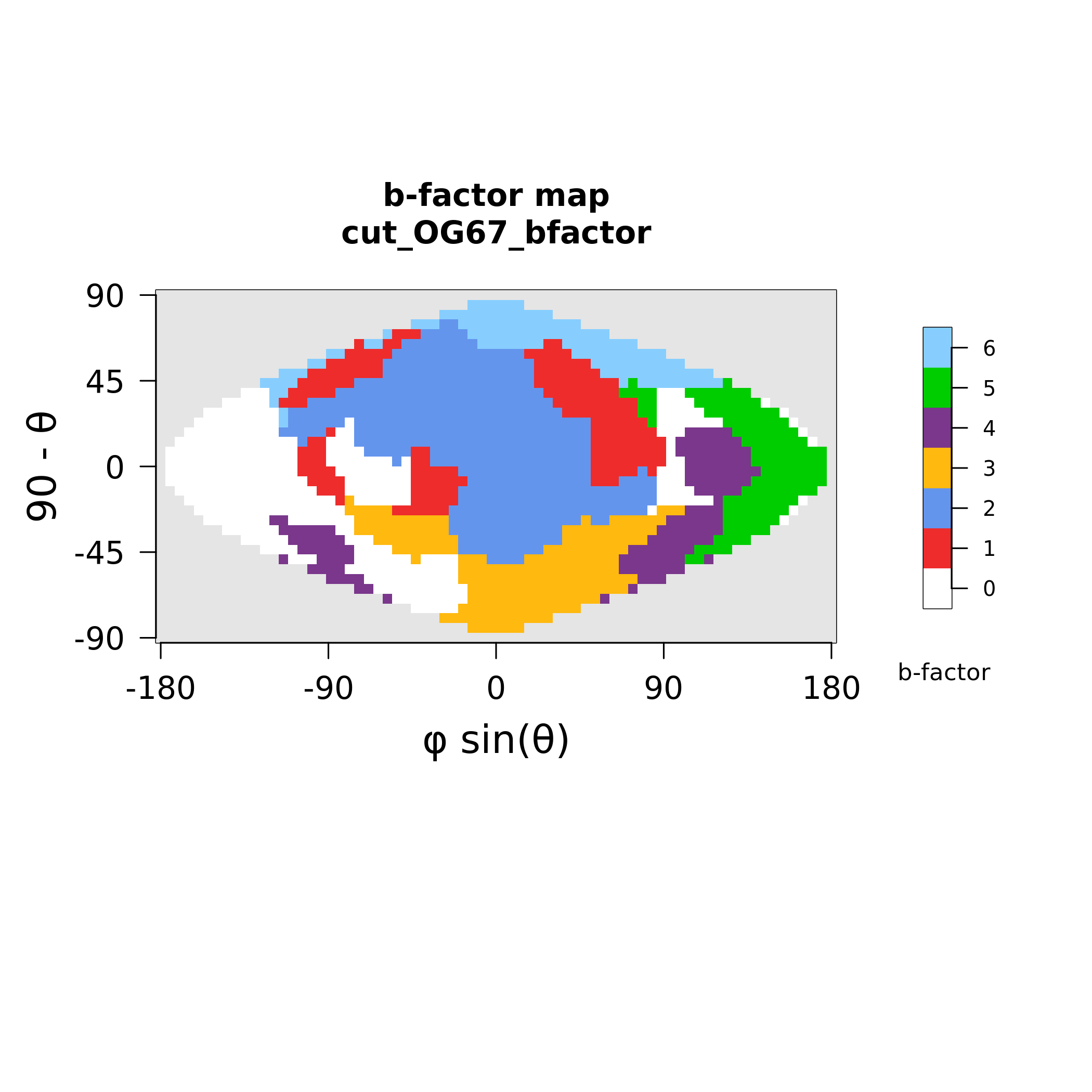

Supplement: S2 File — (ZIP) [file ppat.1012176.s019.zip › S2_File/STRANDS/MAX67_strands.png]

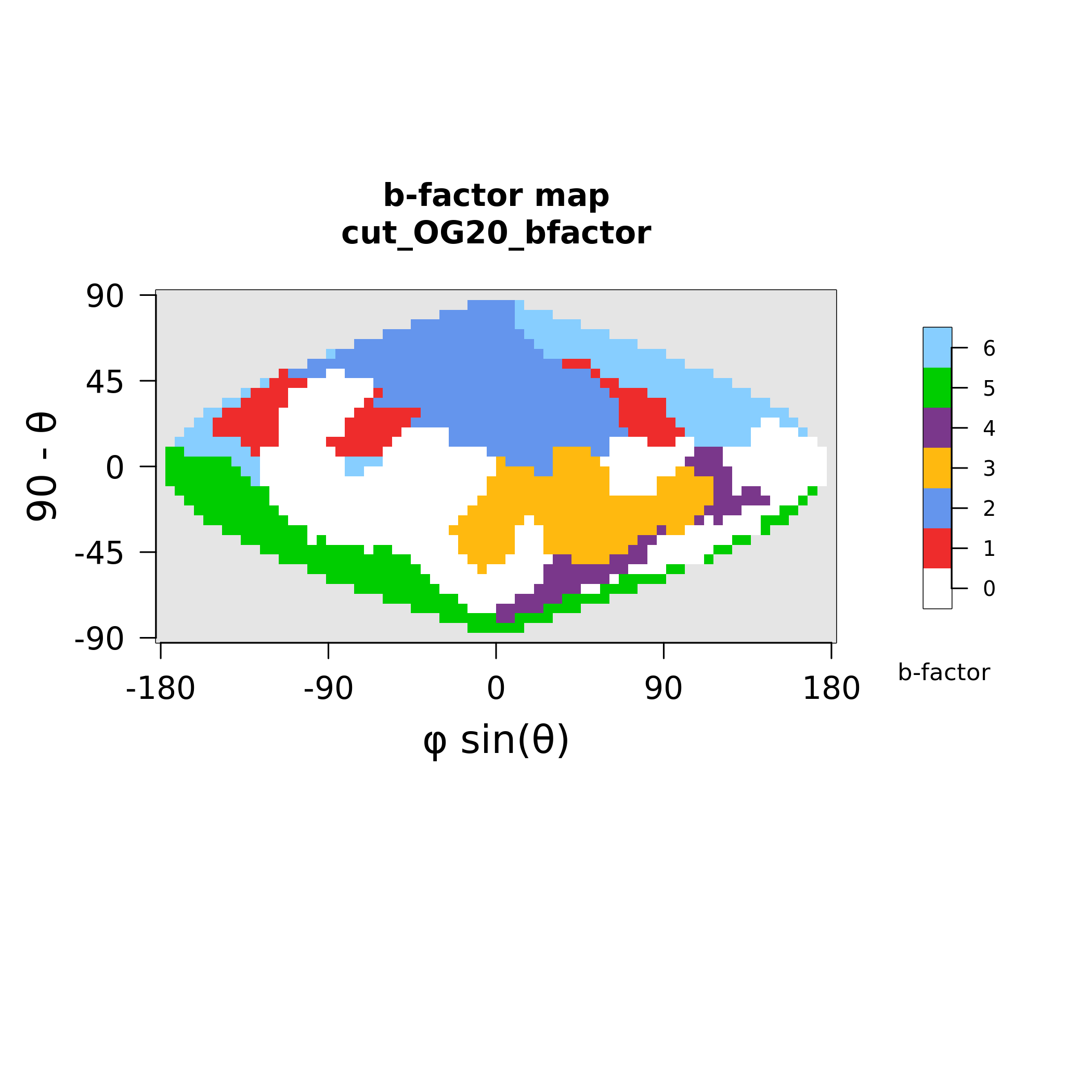

Supplement: S2 File — (ZIP) [file ppat.1012176.s019.zip › S2_File/STRANDS/MAX20_strands.png]

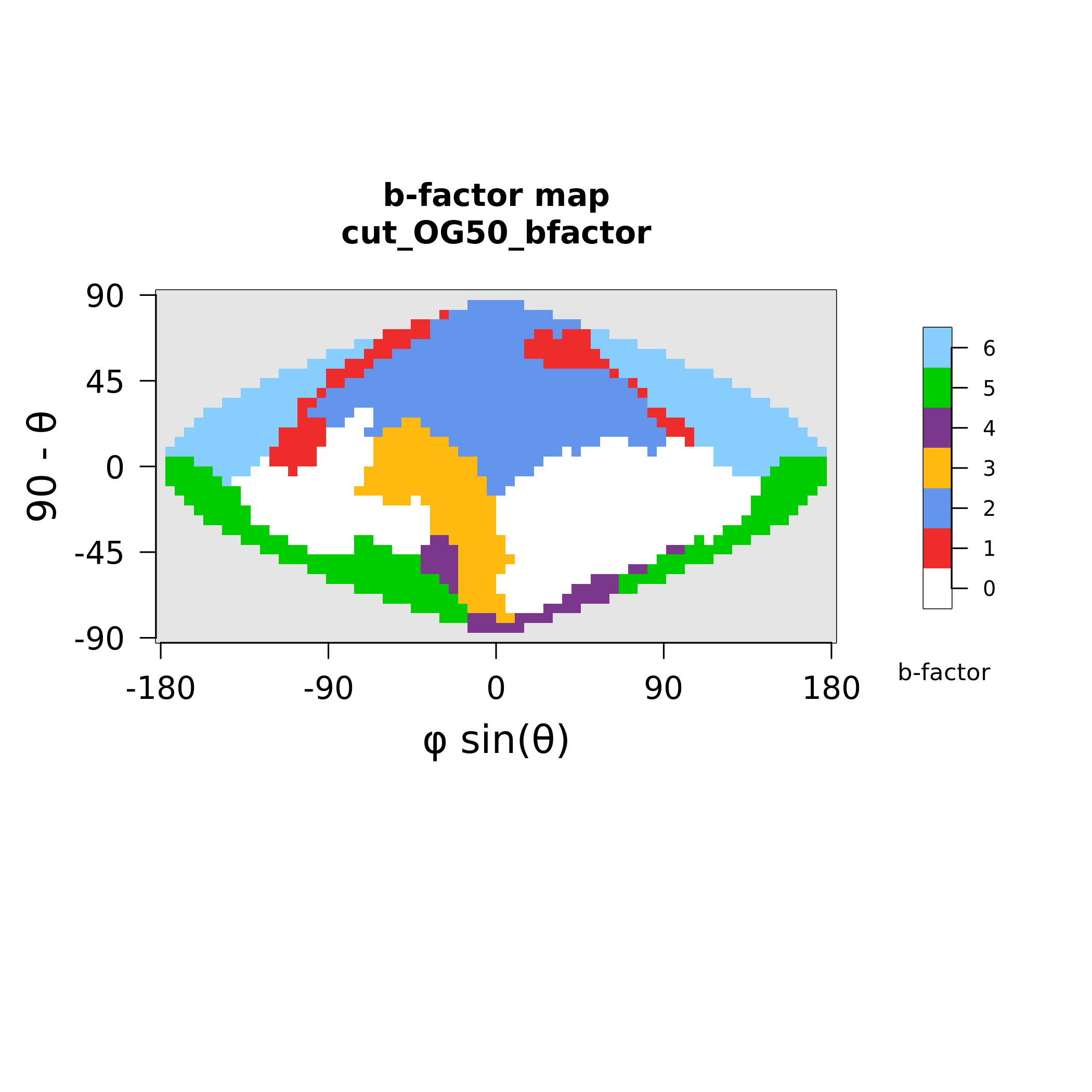

Supplement: S2 File — (ZIP) [file ppat.1012176.s019.zip › S2_File/STRANDS/MAX50_strands.png]

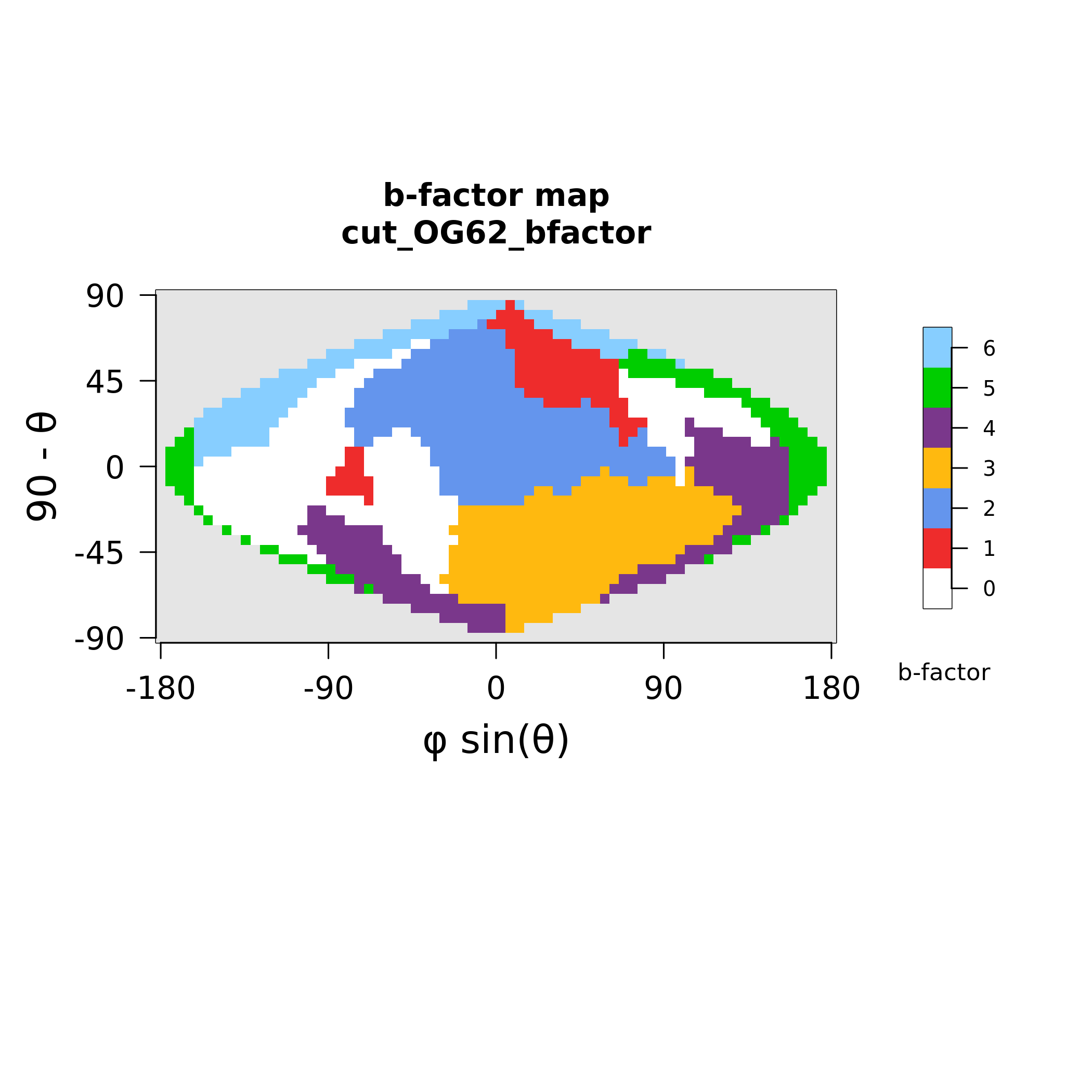

Supplement: S2 File — (ZIP) [file ppat.1012176.s019.zip › S2_File/STRANDS/MAX62_strands.png]

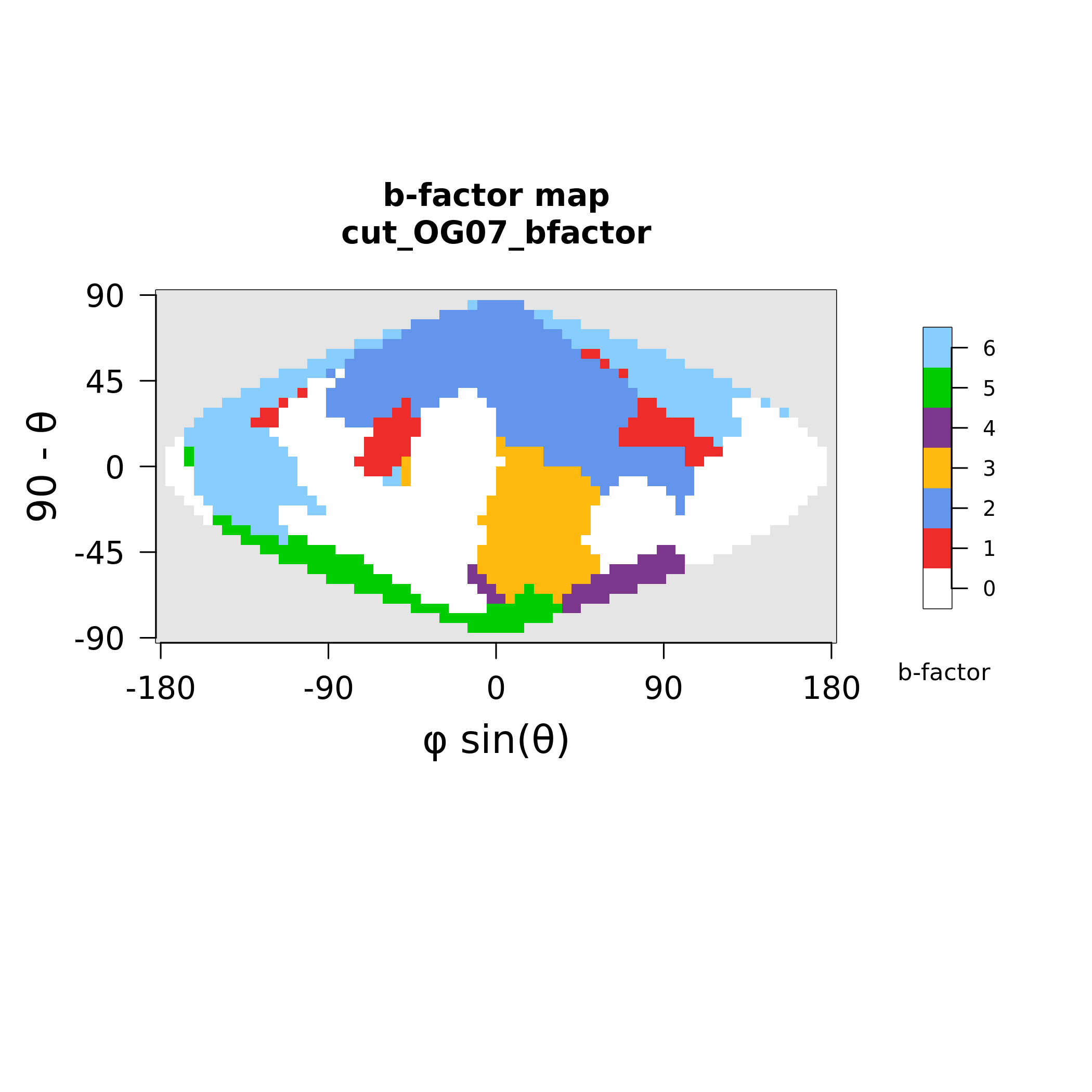

Supplement: S2 File — (ZIP) [file ppat.1012176.s019.zip › S2_File/STRANDS/MAX07_strands.png]

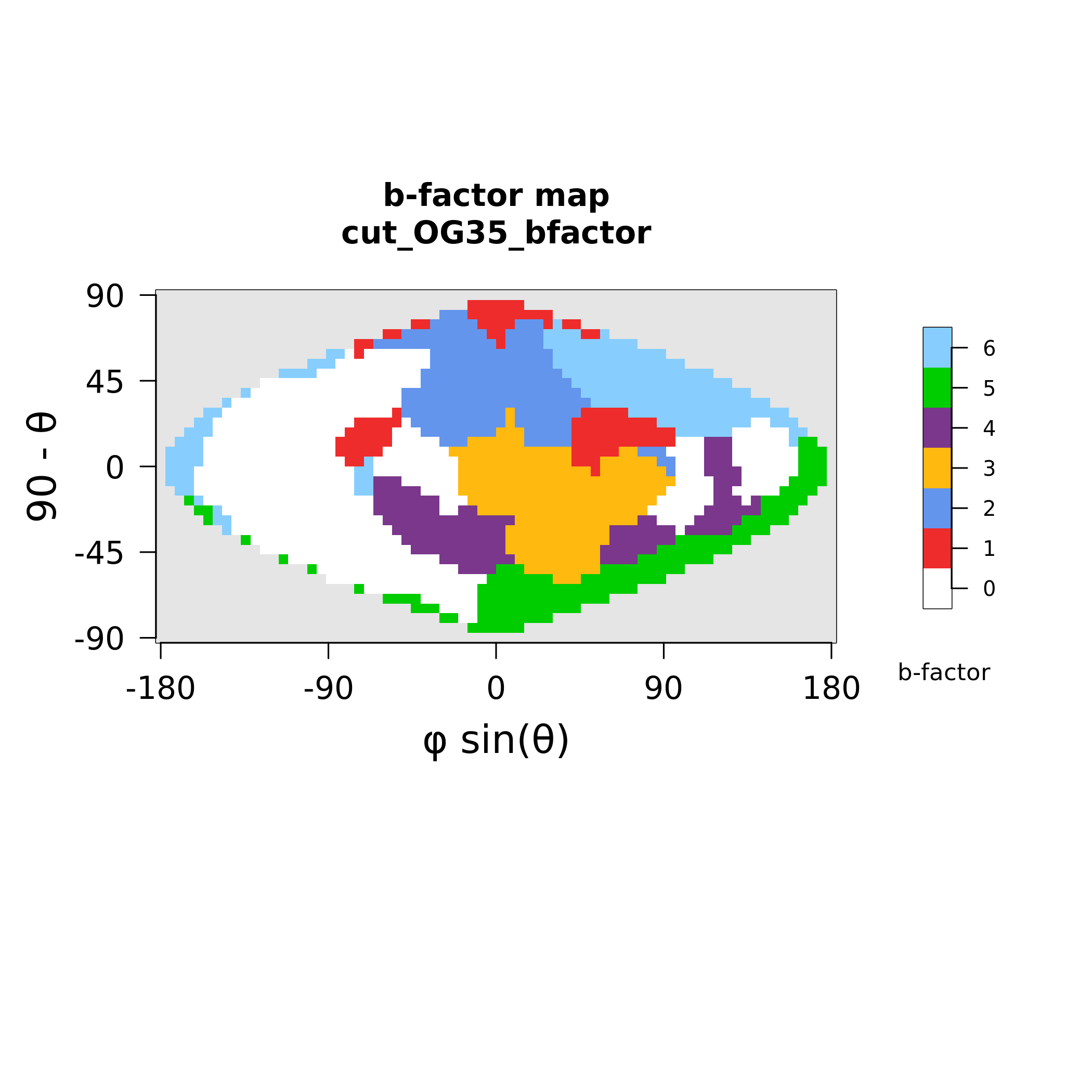

Supplement: S2 File — (ZIP) [file ppat.1012176.s019.zip › S2_File/STRANDS/MAX35_strands.png]

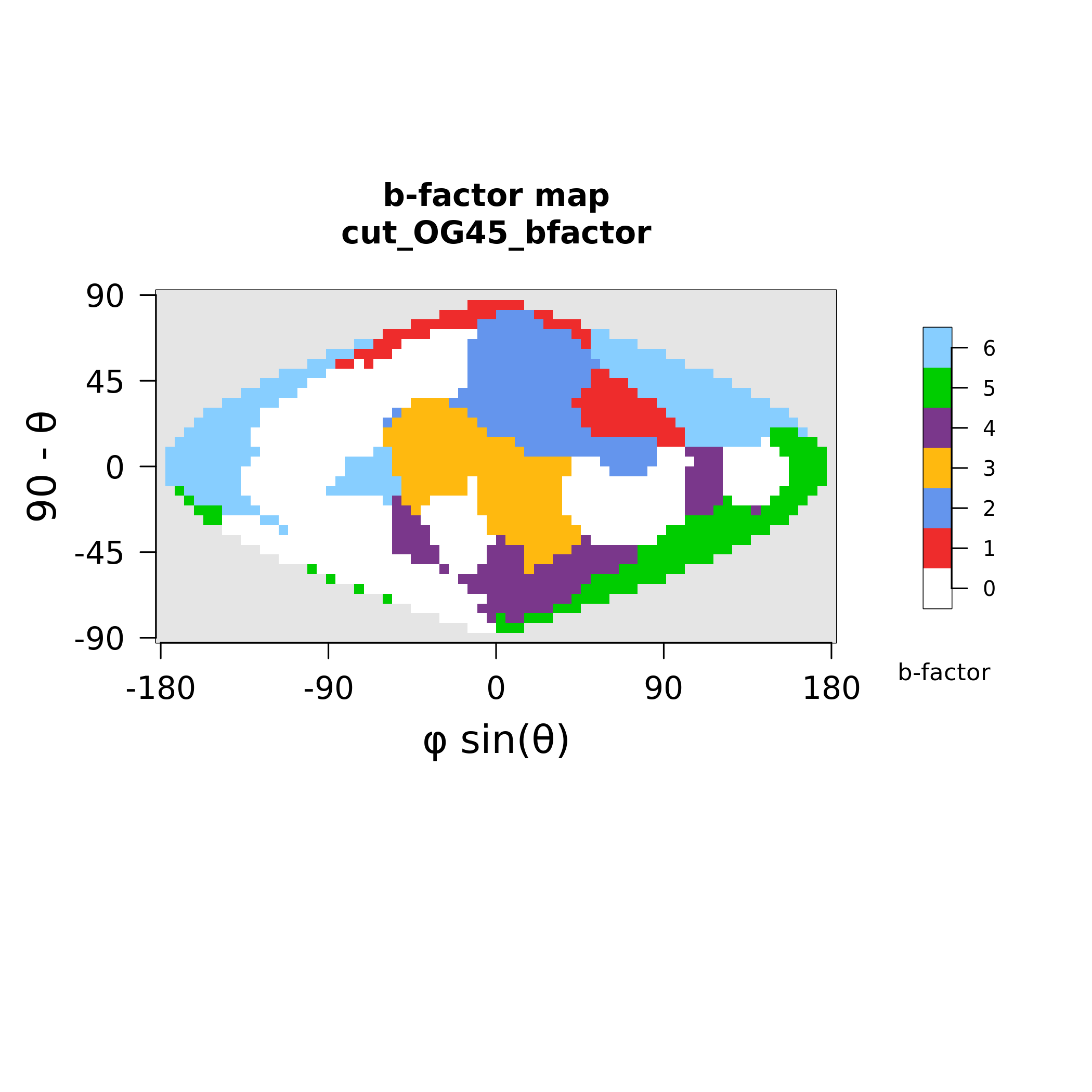

Supplement: S2 File — (ZIP) [file ppat.1012176.s019.zip › S2_File/STRANDS/MAX45_strands.png]

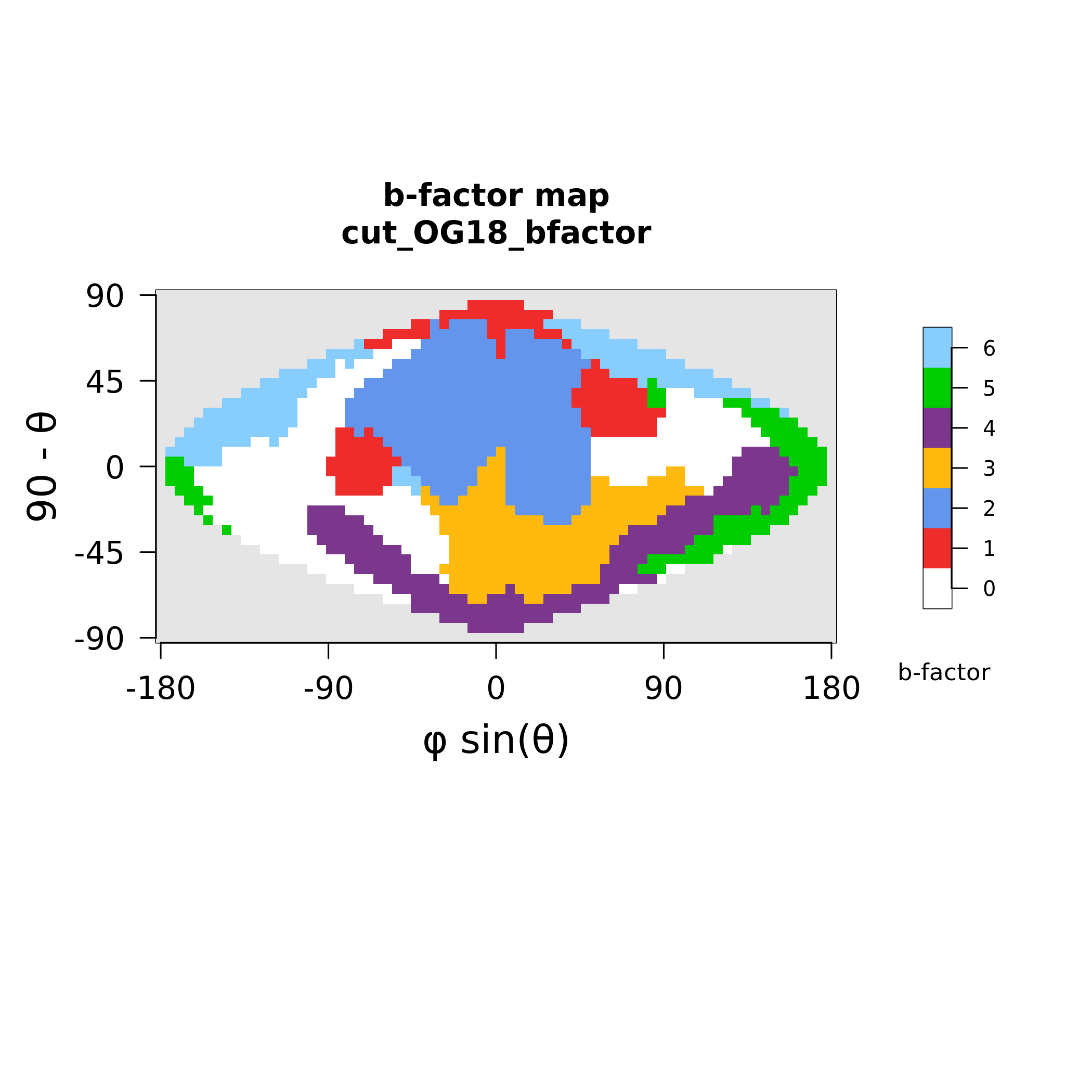

Supplement: S2 File — (ZIP) [file ppat.1012176.s019.zip › S2_File/STRANDS/MAX18_strands.png]

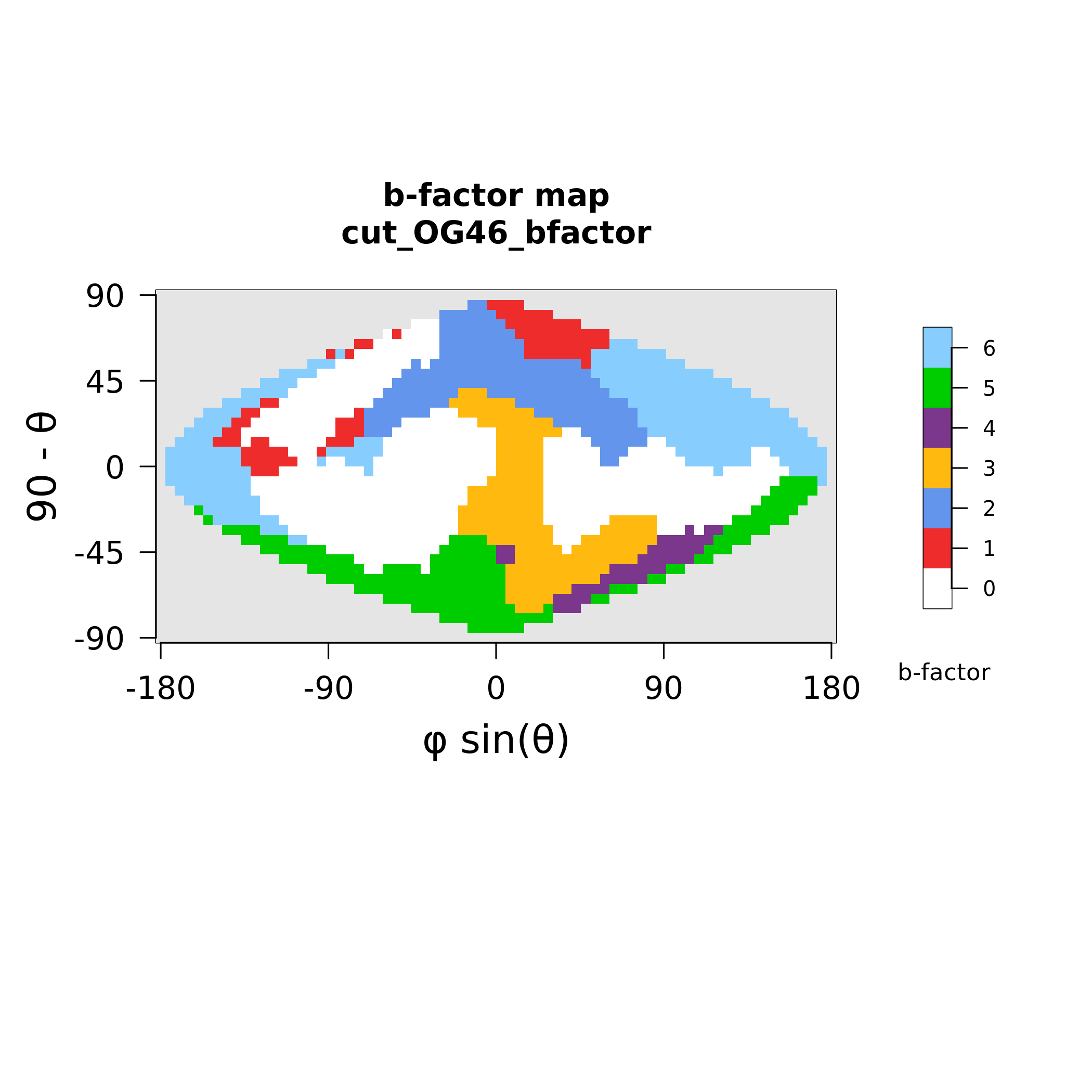

Supplement: S2 File — (ZIP) [file ppat.1012176.s019.zip › S2_File/STRANDS/MAX46_strands.png]

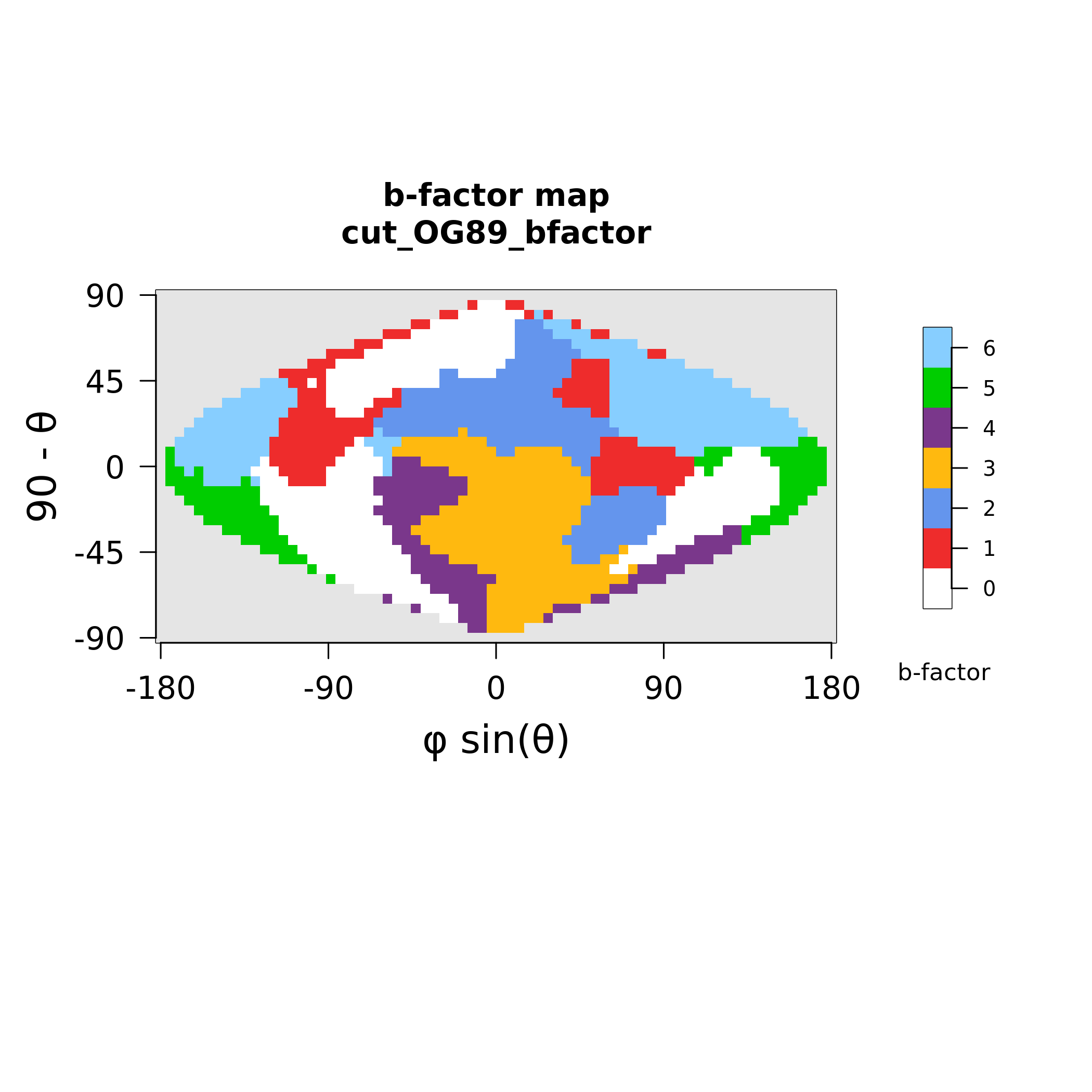

Supplement: S2 File — (ZIP) [file ppat.1012176.s019.zip › S2_File/STRANDS/MAX89_strands.png]

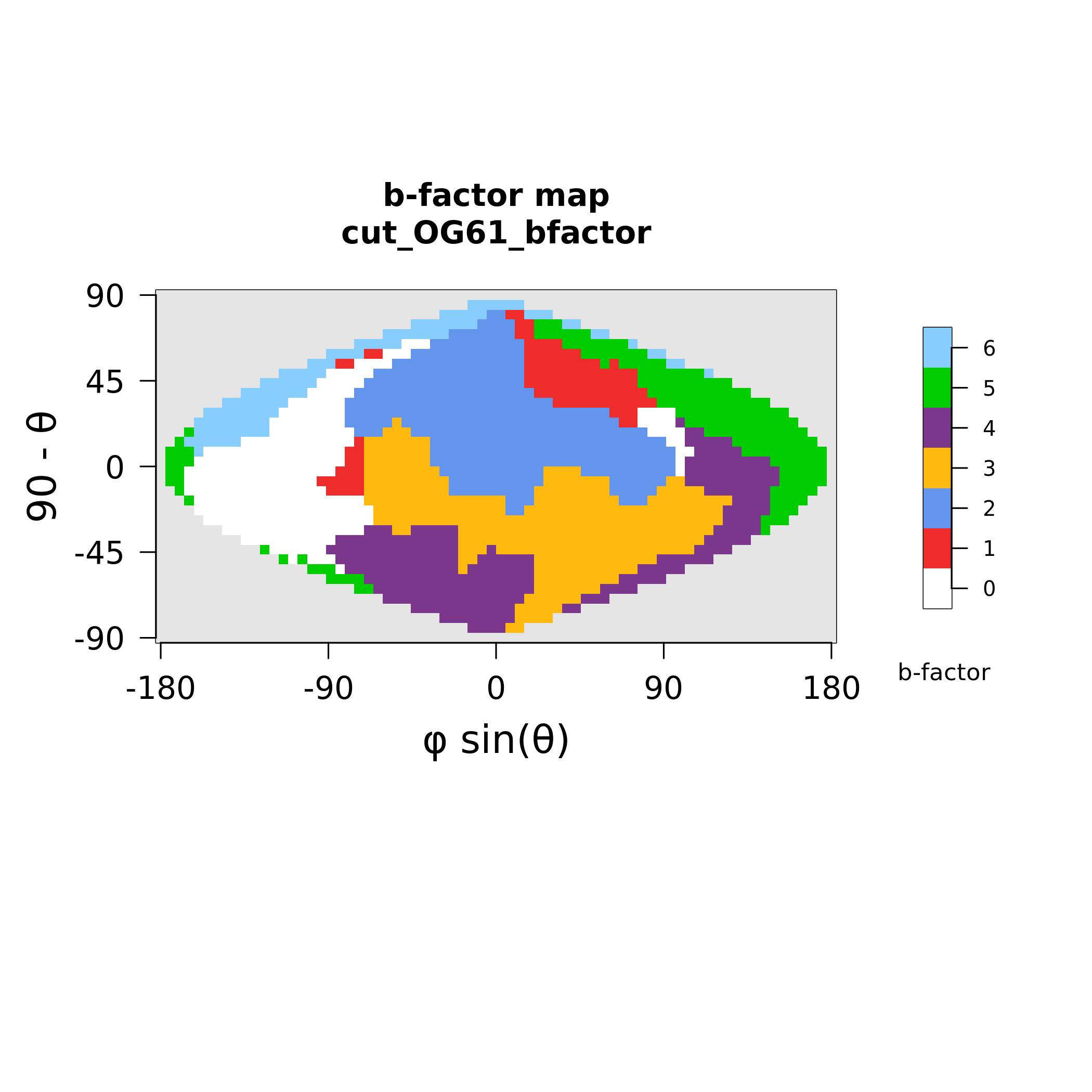

Supplement: S2 File — (ZIP) [file ppat.1012176.s019.zip › S2_File/STRANDS/MAX61_strands.png]

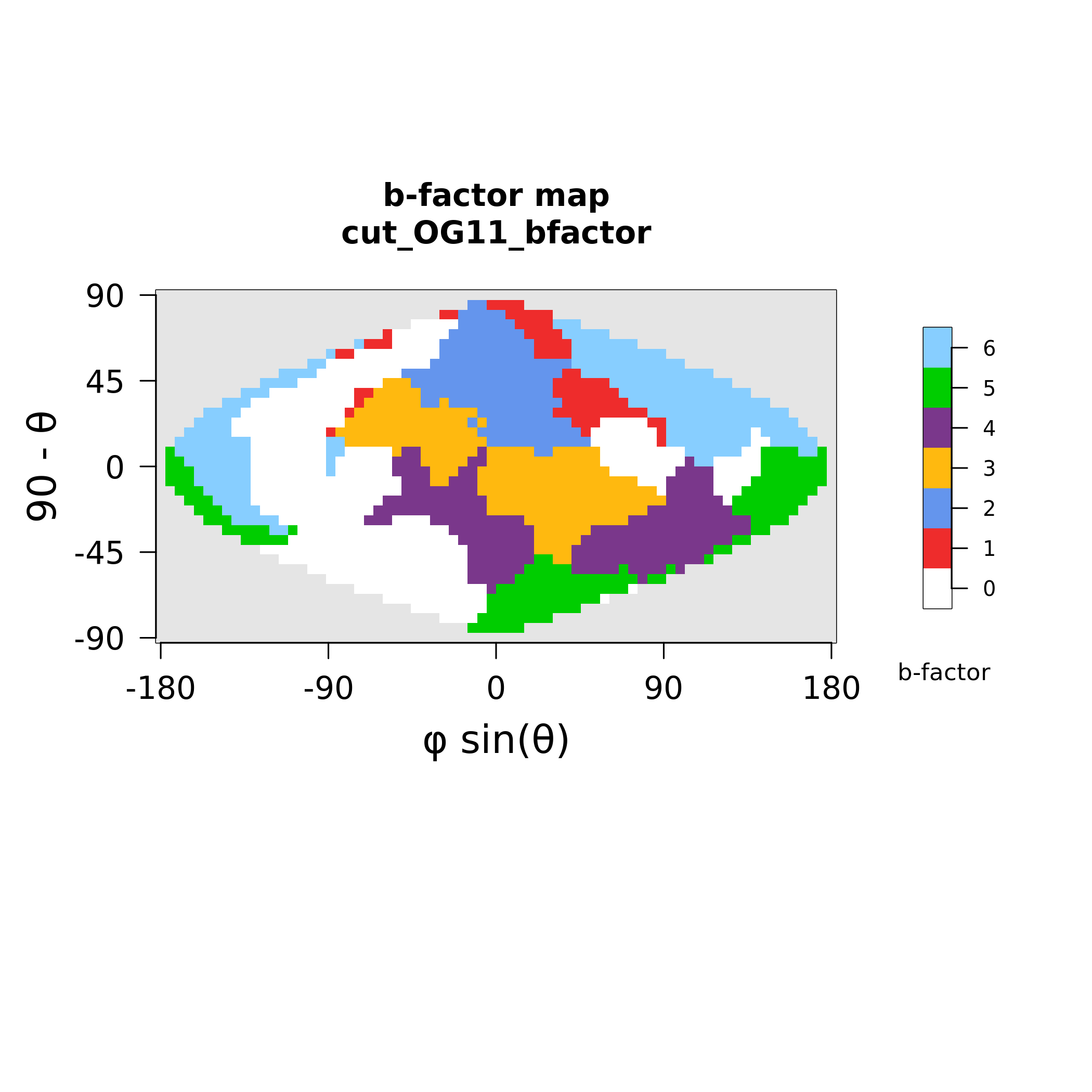

Supplement: S2 File — (ZIP) [file ppat.1012176.s019.zip › S2_File/STRANDS/MAX11_strands.png]

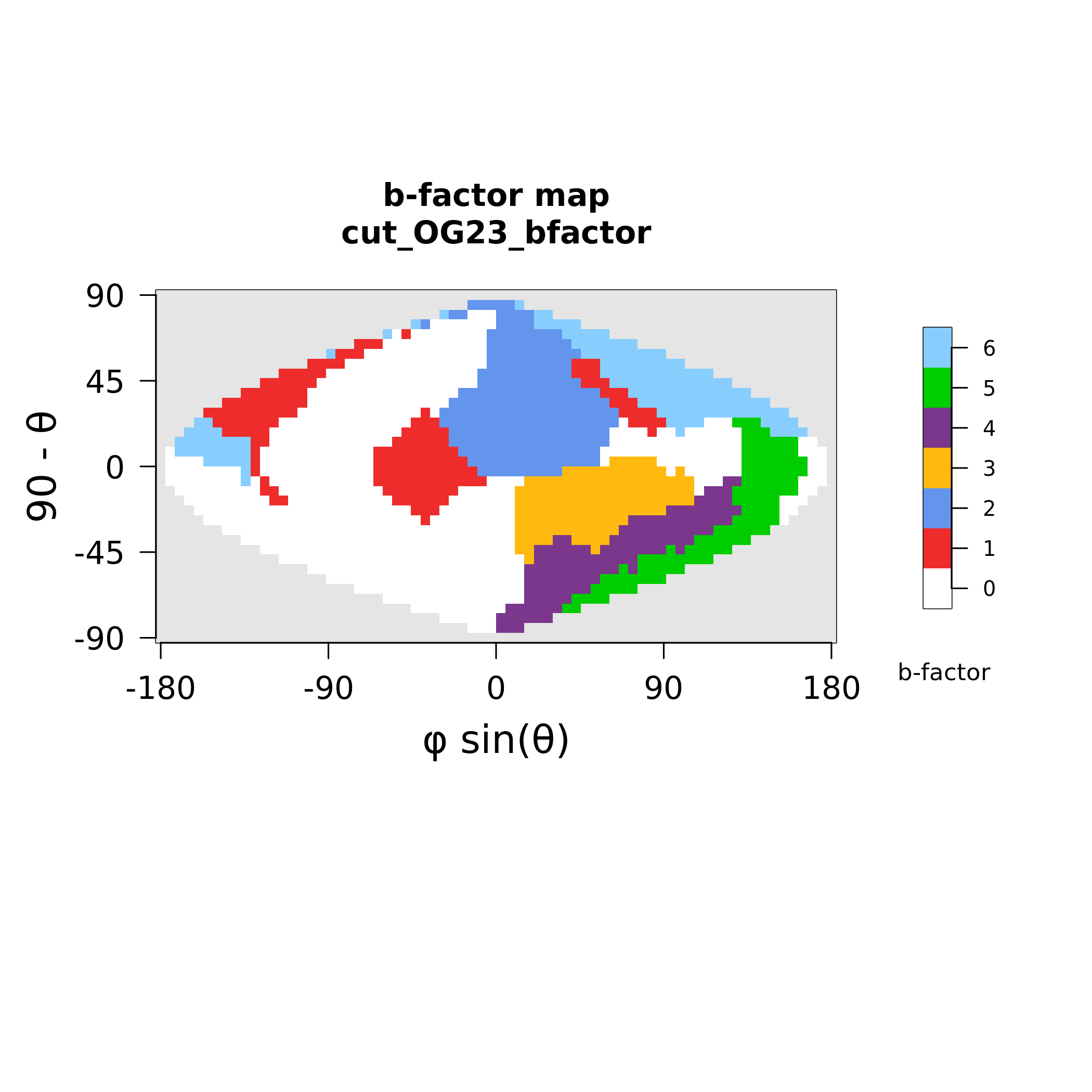

Supplement: S2 File — (ZIP) [file ppat.1012176.s019.zip › S2_File/STRANDS/MAX23_strands.png]

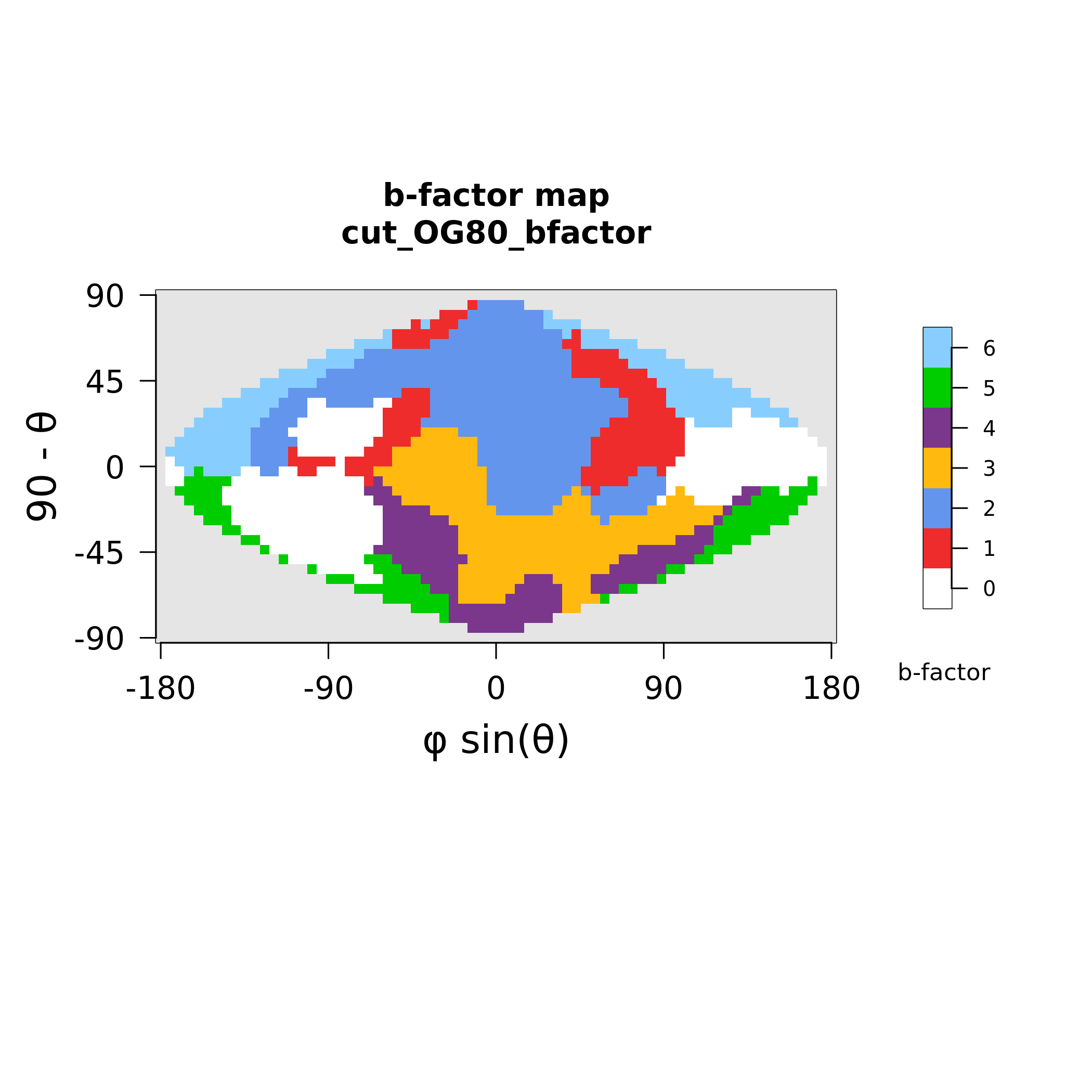

Supplement: S2 File — (ZIP) [file ppat.1012176.s019.zip › S2_File/STRANDS/MAX80_strands.png]

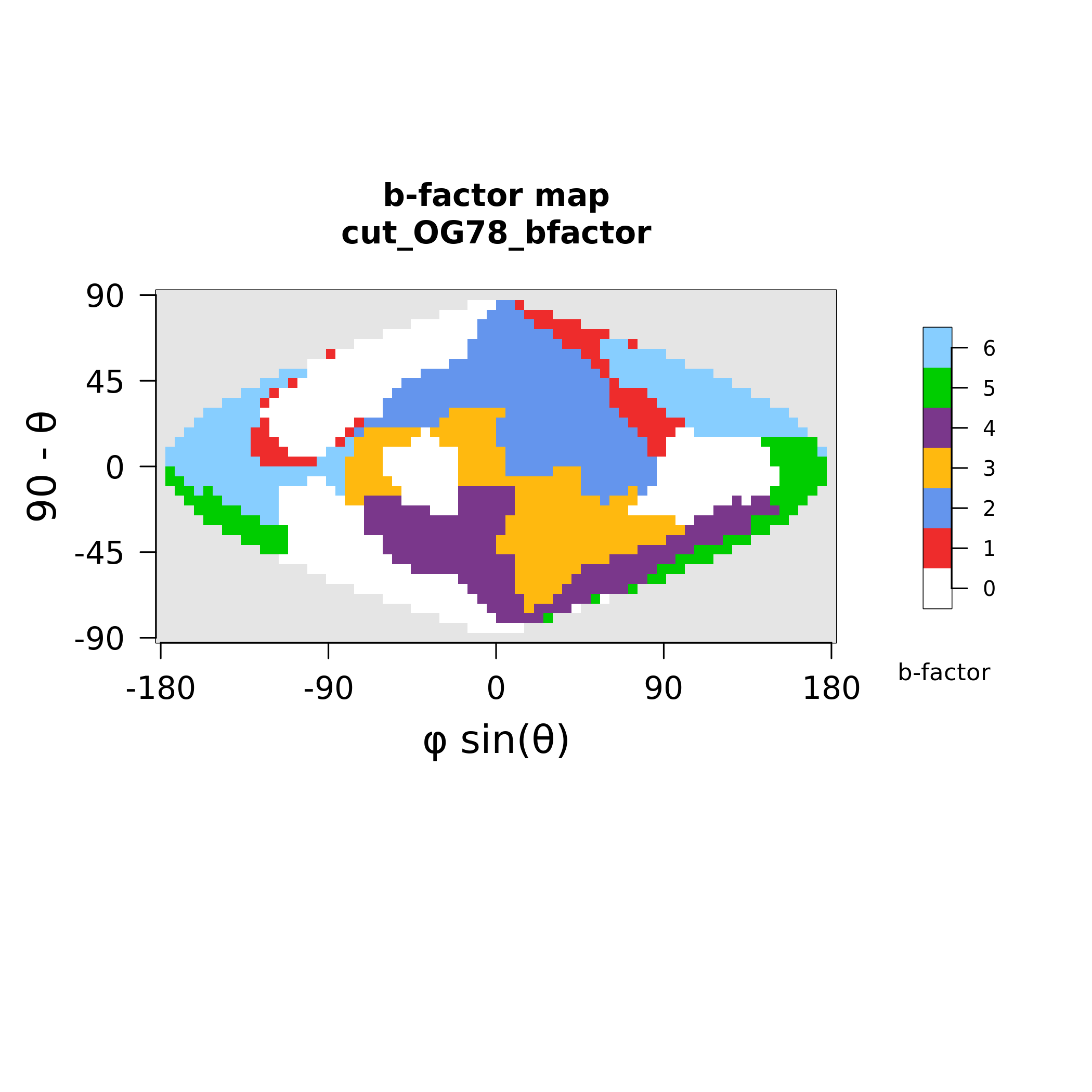

Supplement: S2 File — (ZIP) [file ppat.1012176.s019.zip › S2_File/STRANDS/MAX78_strands.png]

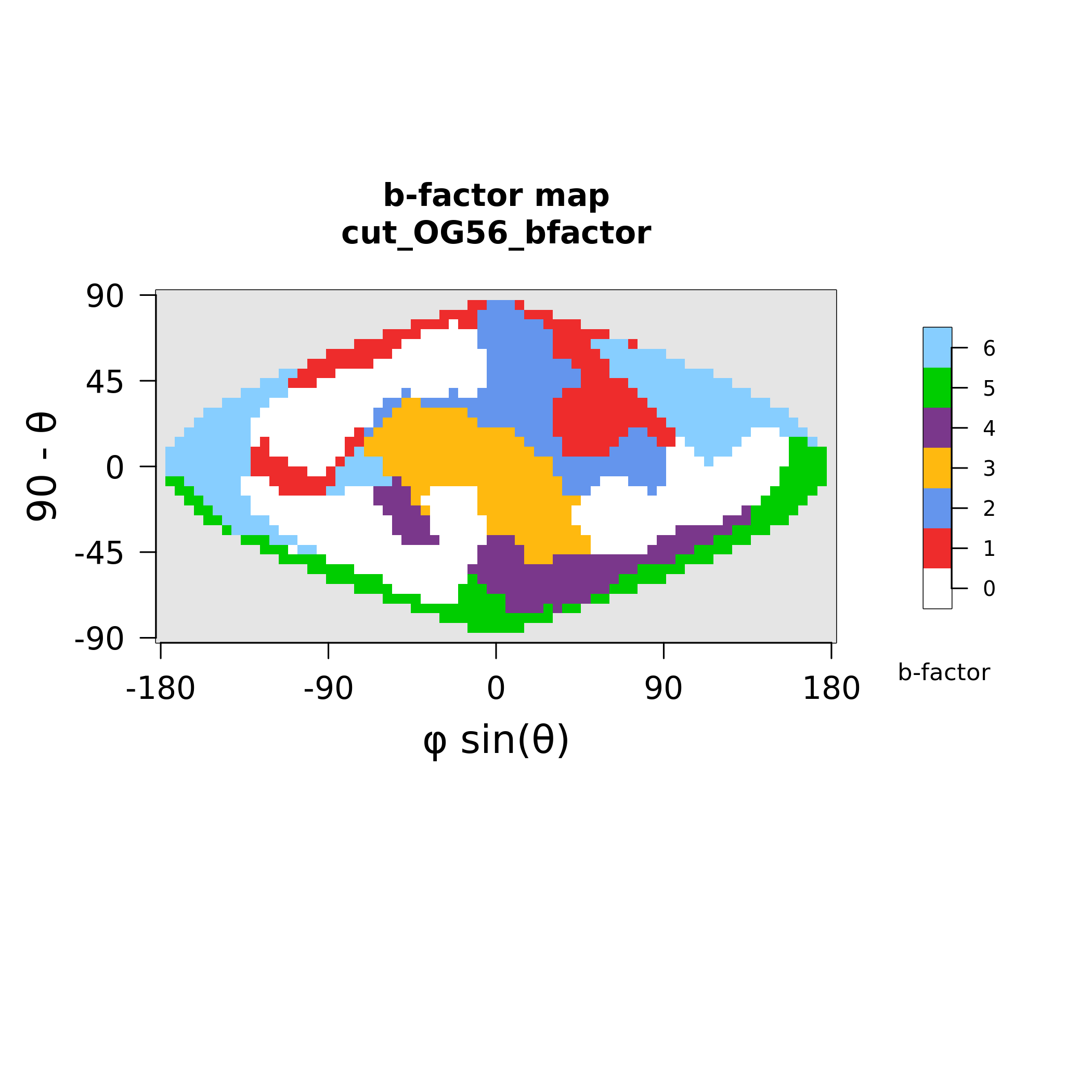

Supplement: S2 File — (ZIP) [file ppat.1012176.s019.zip › S2_File/STRANDS/MAX56_strands.png]

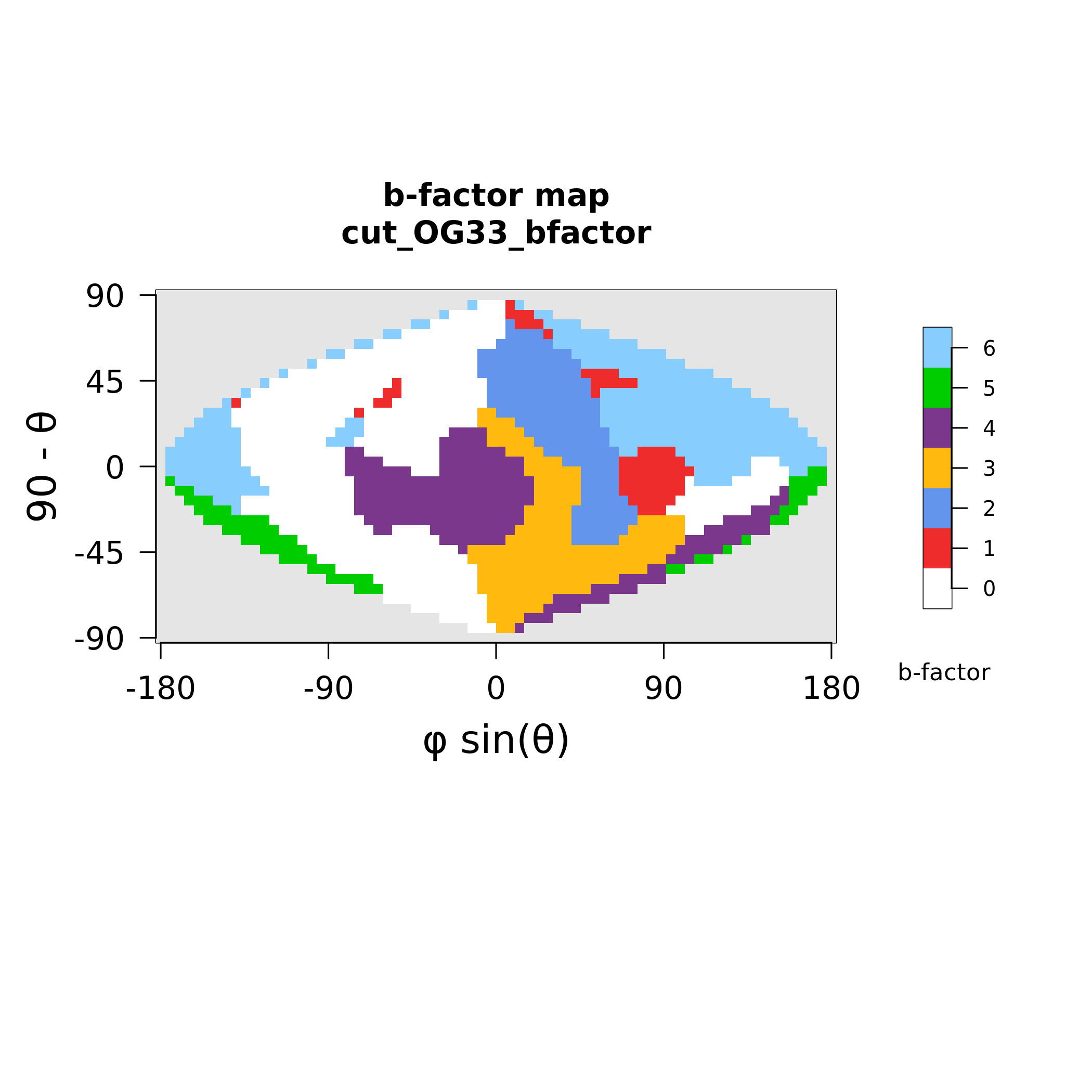

Supplement: S2 File — (ZIP) [file ppat.1012176.s019.zip › S2_File/STRANDS/MAX33_strands.png]

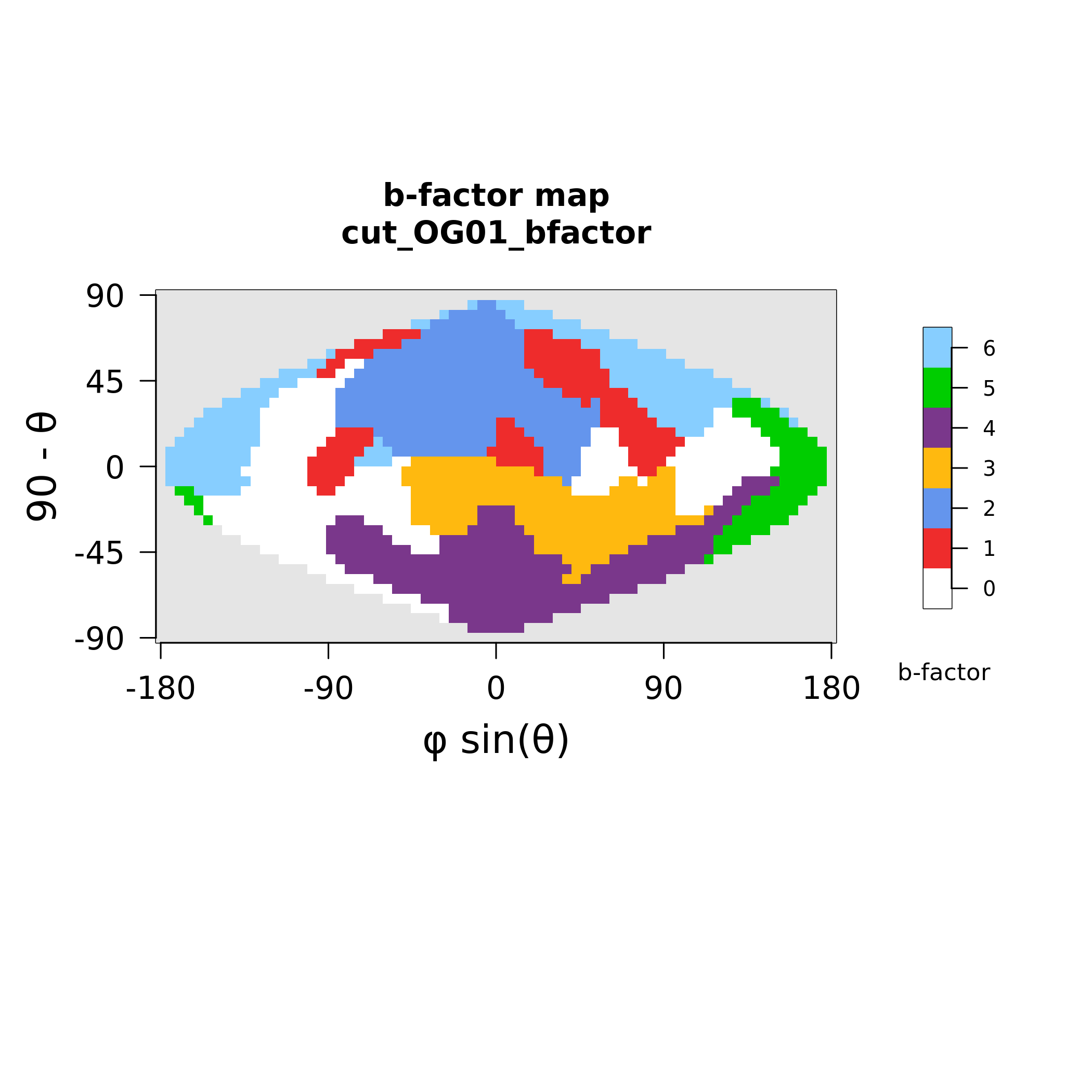

Supplement: S2 File — (ZIP) [file ppat.1012176.s019.zip › S2_File/STRANDS/MAX01_strands.png]

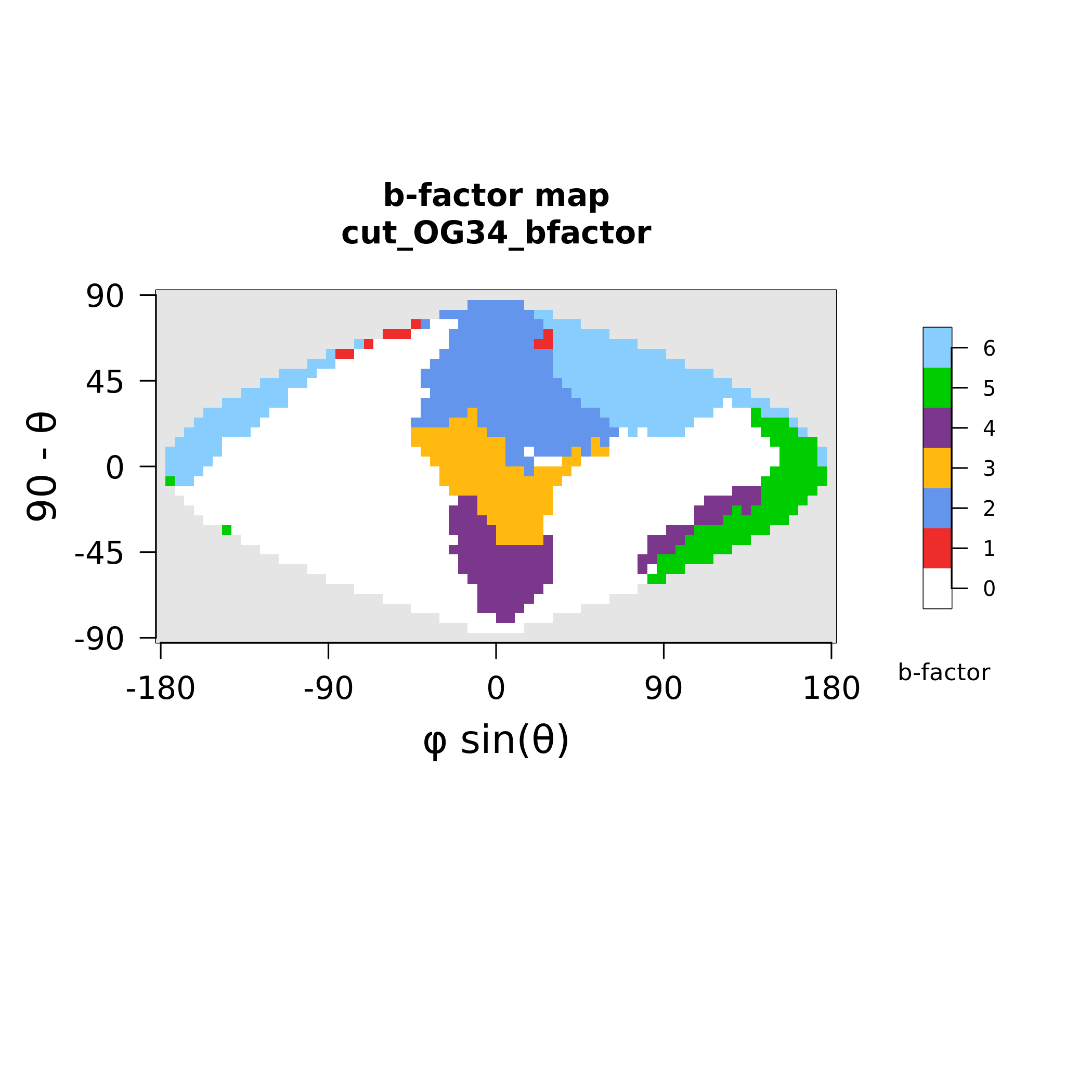

Supplement: S2 File — (ZIP) [file ppat.1012176.s019.zip › S2_File/STRANDS/MAX34_strands.png]

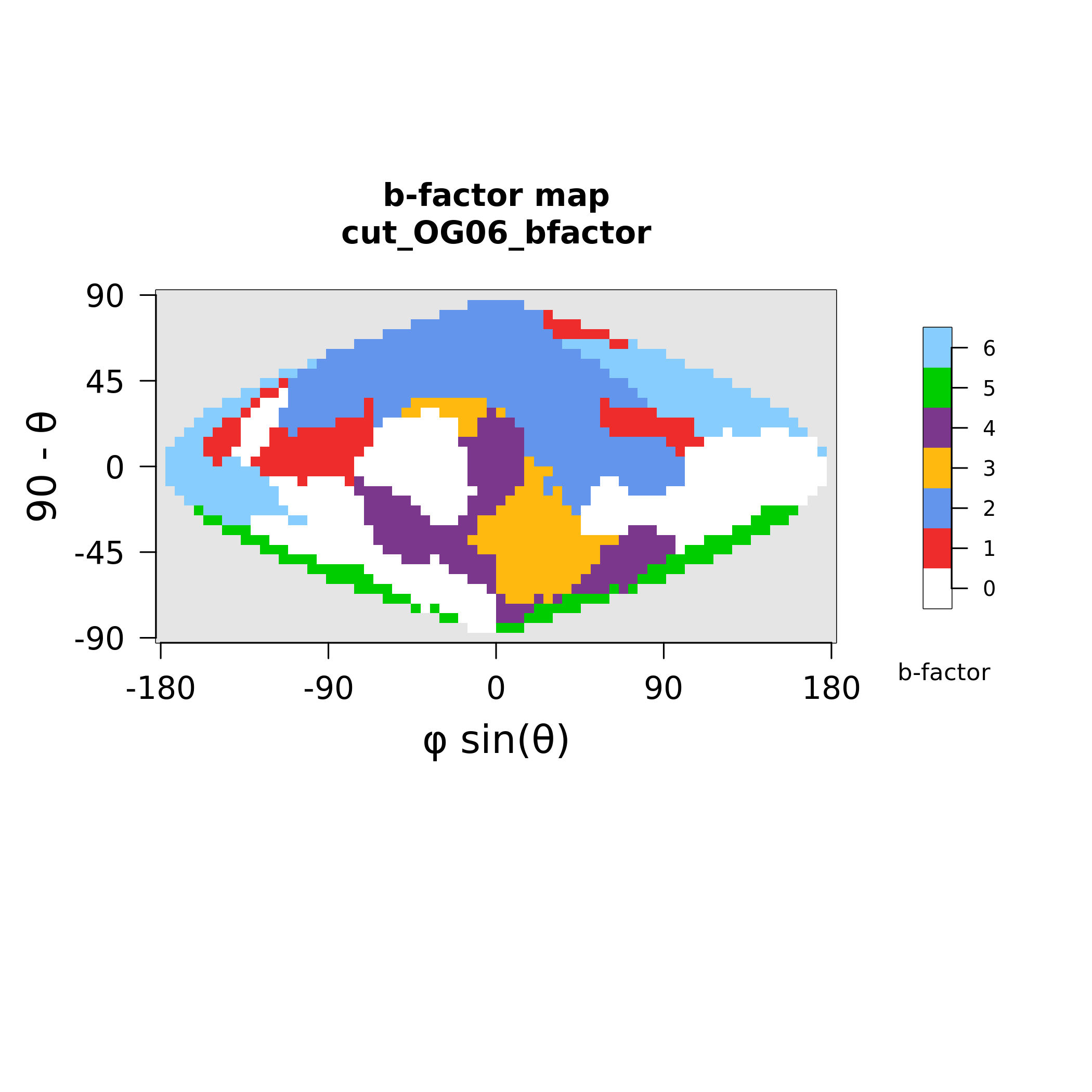

Supplement: S2 File — (ZIP) [file ppat.1012176.s019.zip › S2_File/STRANDS/MAX06_strands.png]

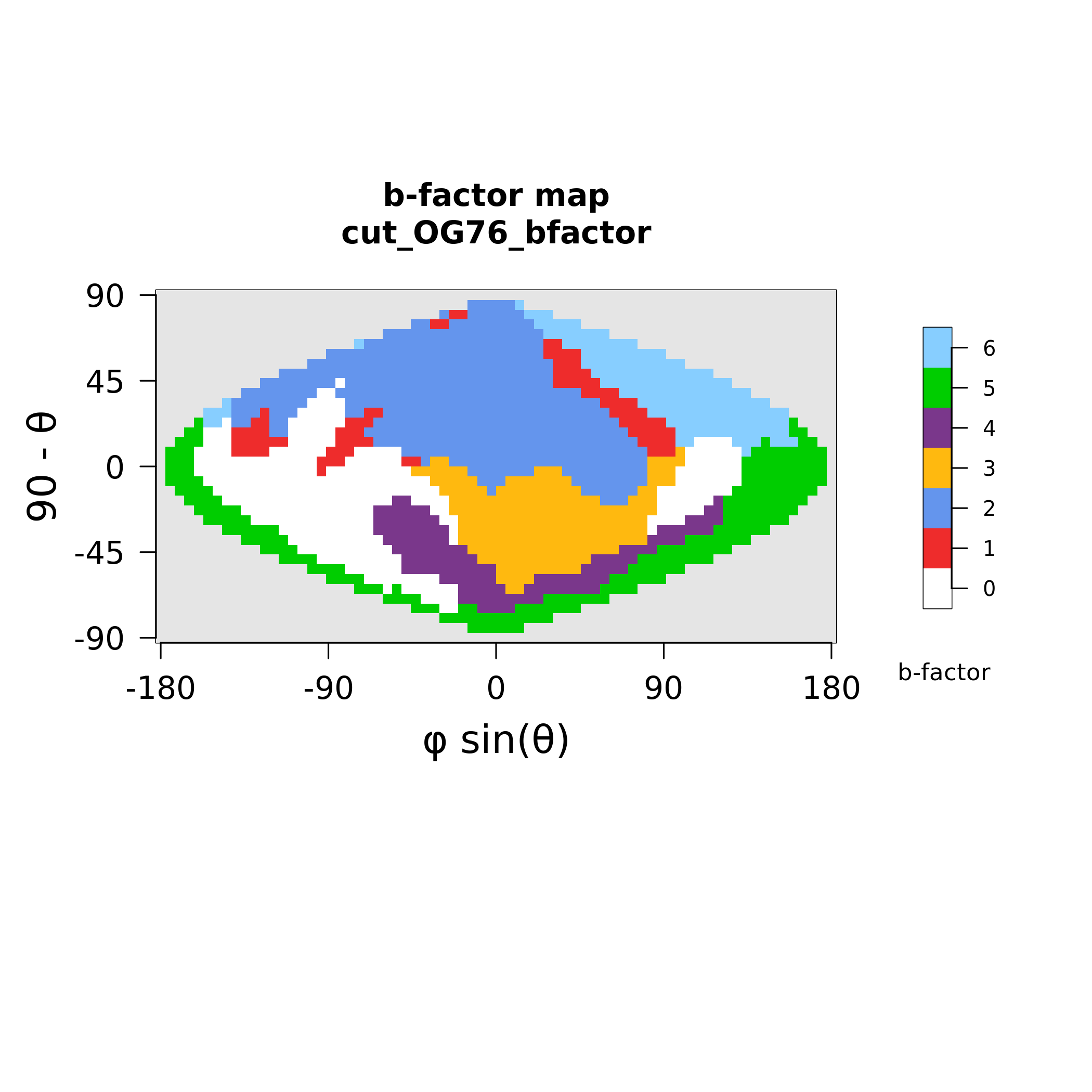

Supplement: S2 File — (ZIP) [file ppat.1012176.s019.zip › S2_File/STRANDS/MAX76_strands.png]

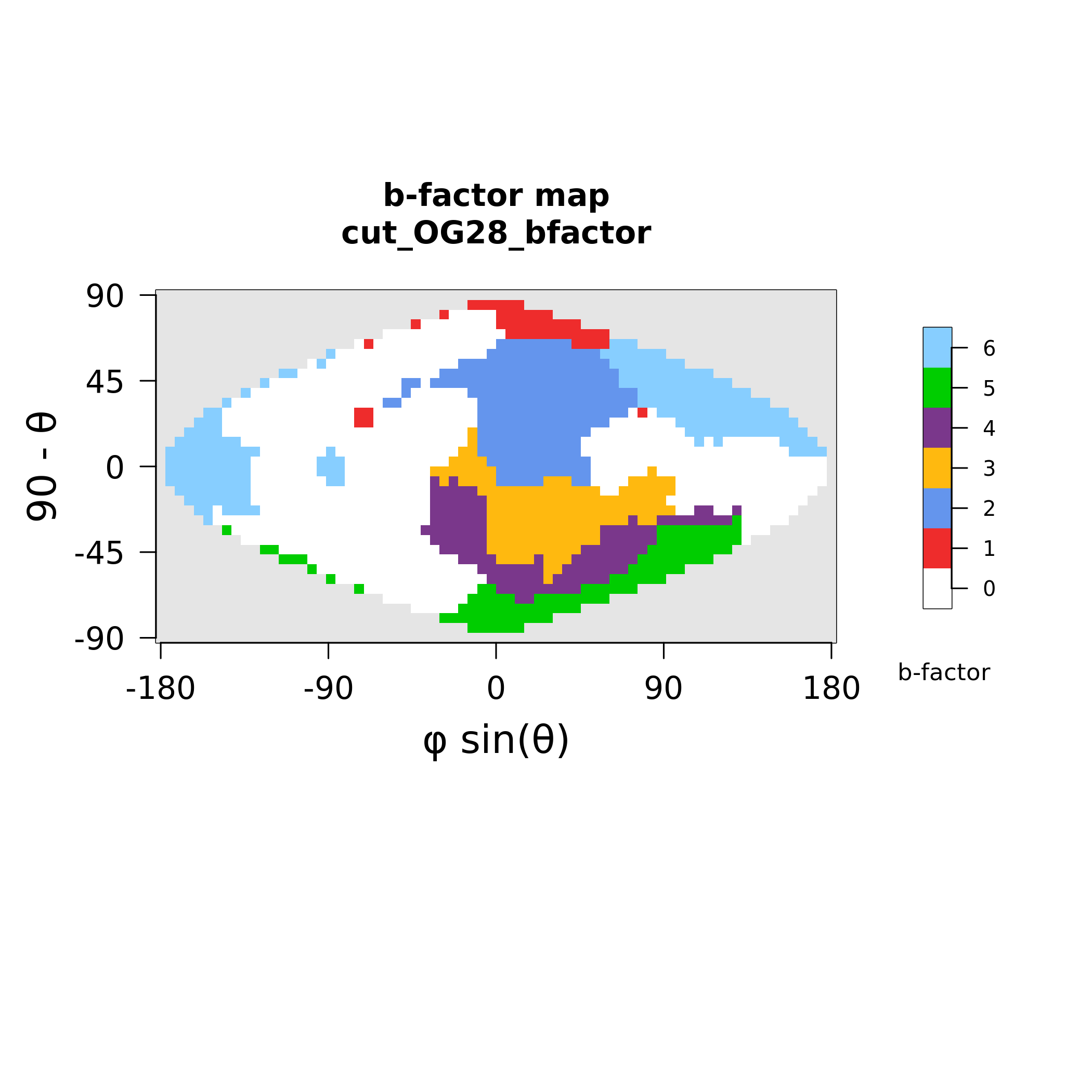

Supplement: S2 File — (ZIP) [file ppat.1012176.s019.zip › S2_File/STRANDS/MAX28_strands.png]

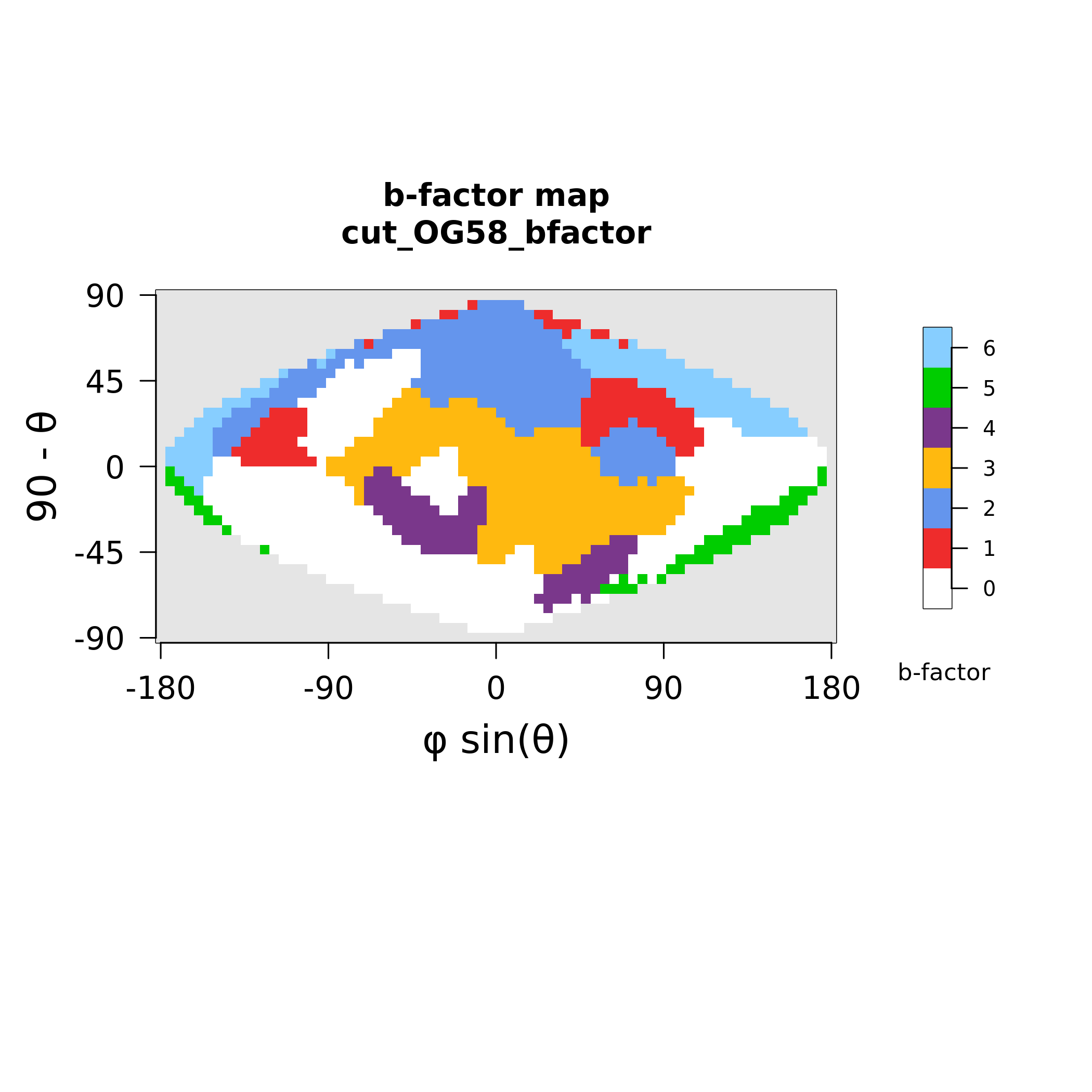

Supplement: S2 File — (ZIP) [file ppat.1012176.s019.zip › S2_File/STRANDS/MAX58_strands.png]

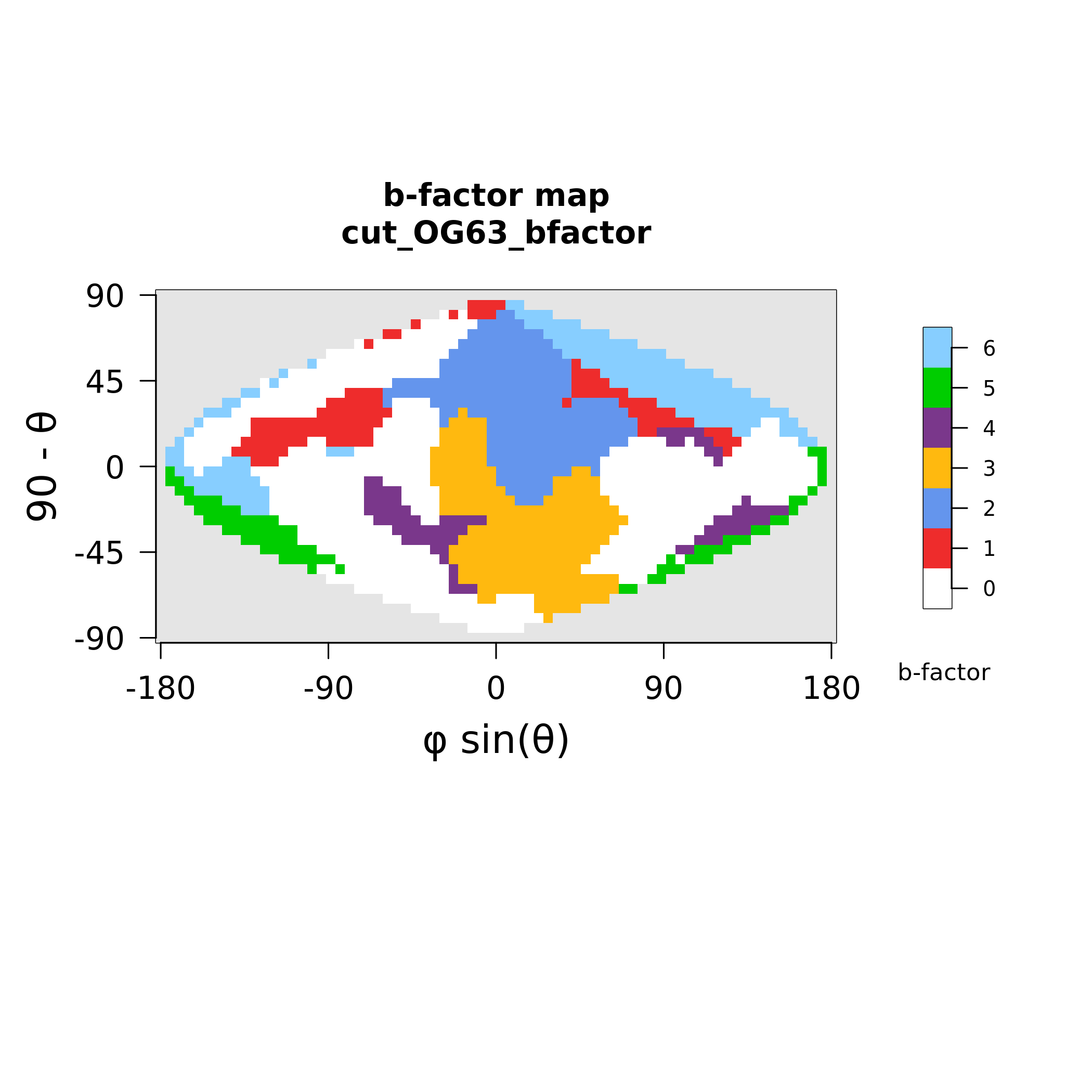

Supplement: S2 File — (ZIP) [file ppat.1012176.s019.zip › S2_File/STRANDS/MAX63_strands.png]

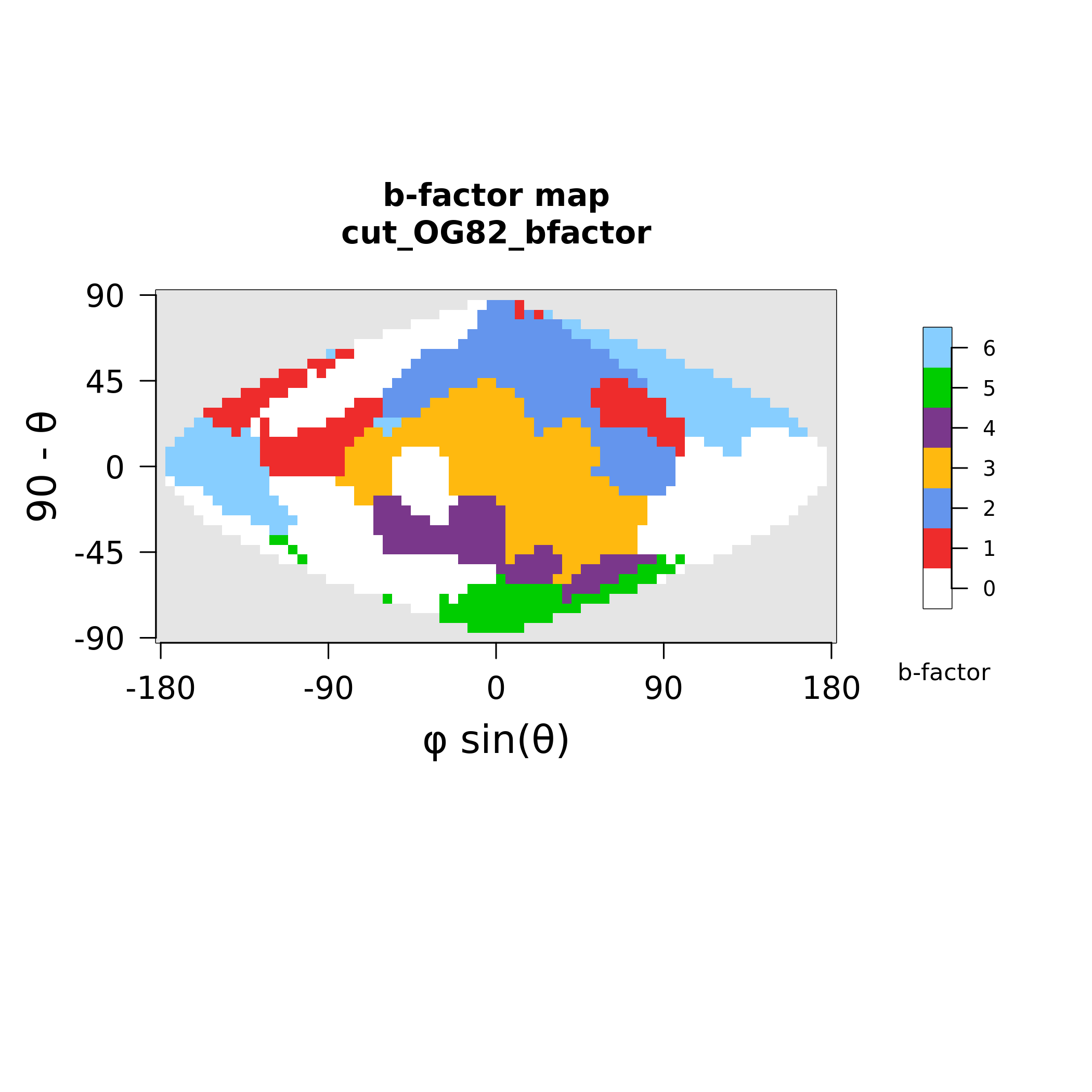

Supplement: S2 File — (ZIP) [file ppat.1012176.s019.zip › S2_File/STRANDS/MAX82_strands.png]

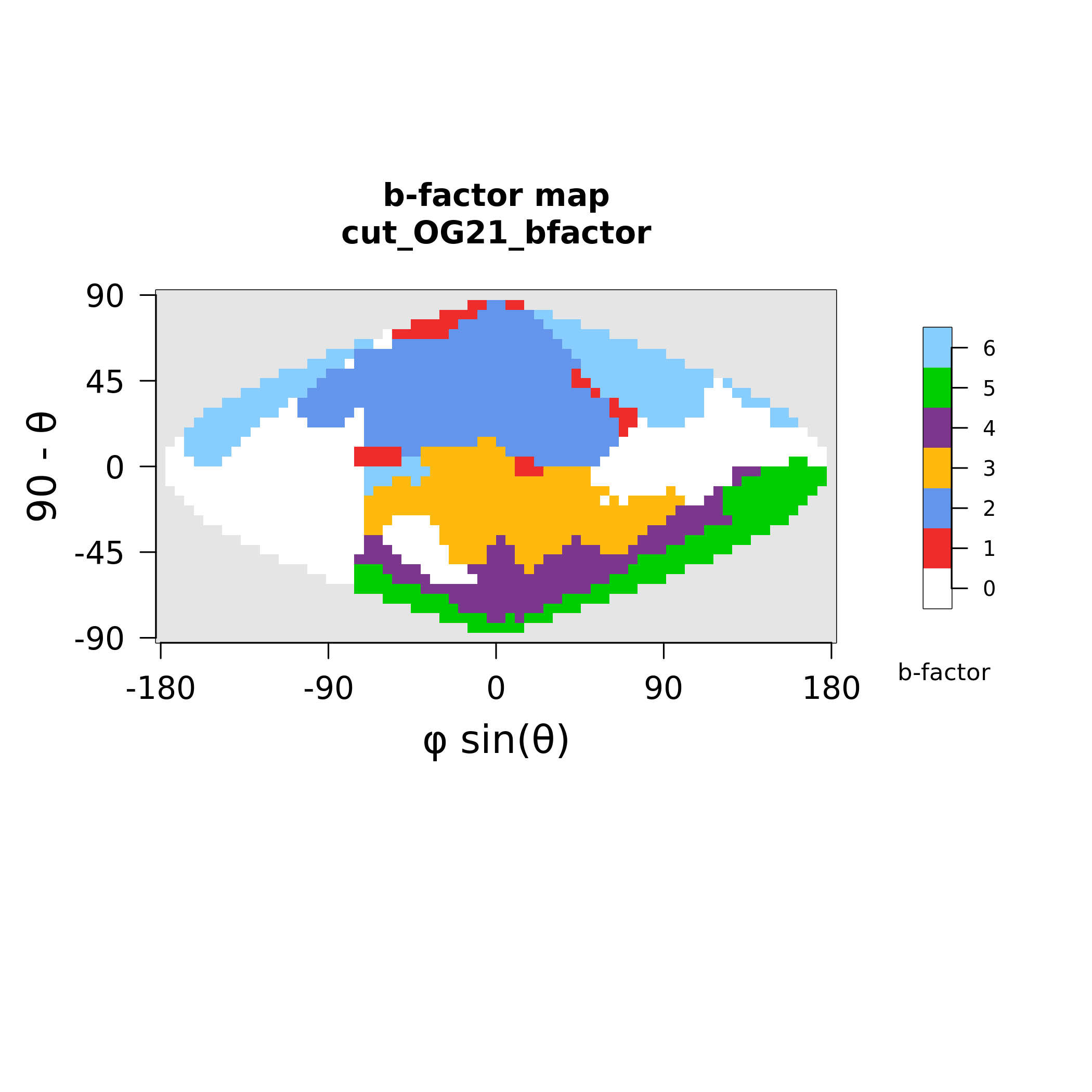

Supplement: S2 File — (ZIP) [file ppat.1012176.s019.zip › S2_File/STRANDS/MAX21_strands.png]

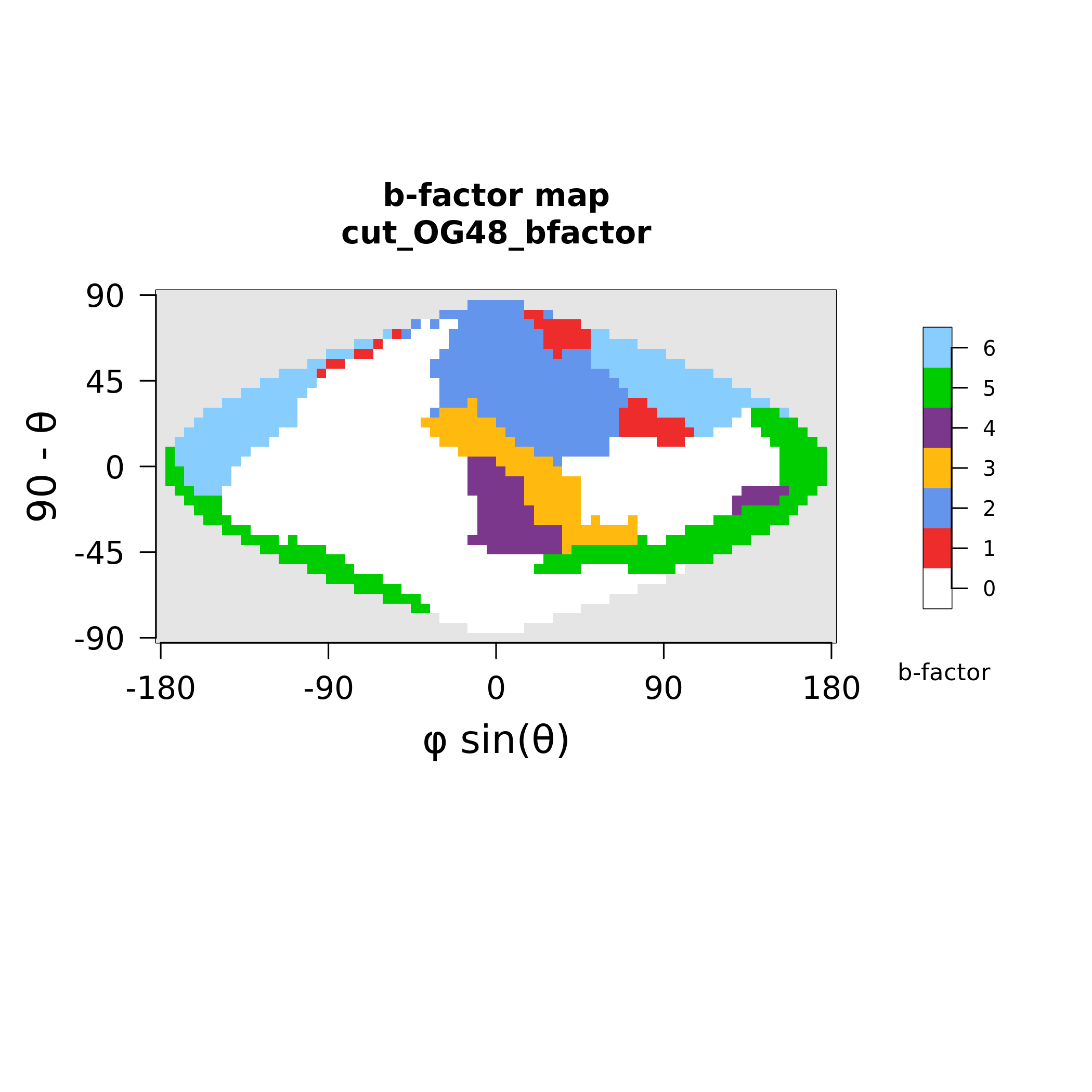

Supplement: S2 File — (ZIP) [file ppat.1012176.s019.zip › S2_File/STRANDS/MAX48_strands.png]

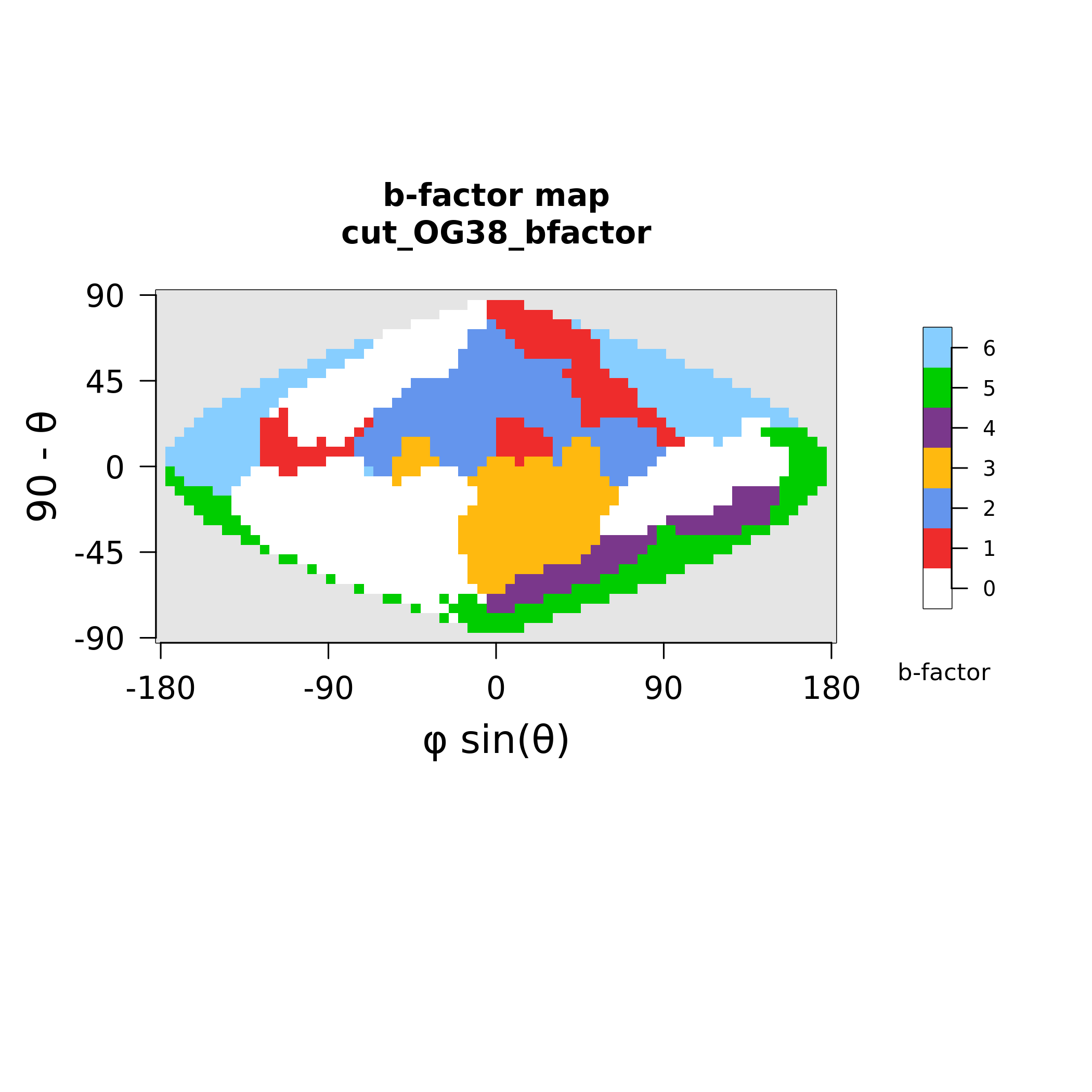

Supplement: S2 File — (ZIP) [file ppat.1012176.s019.zip › S2_File/STRANDS/MAX38_strands.png]

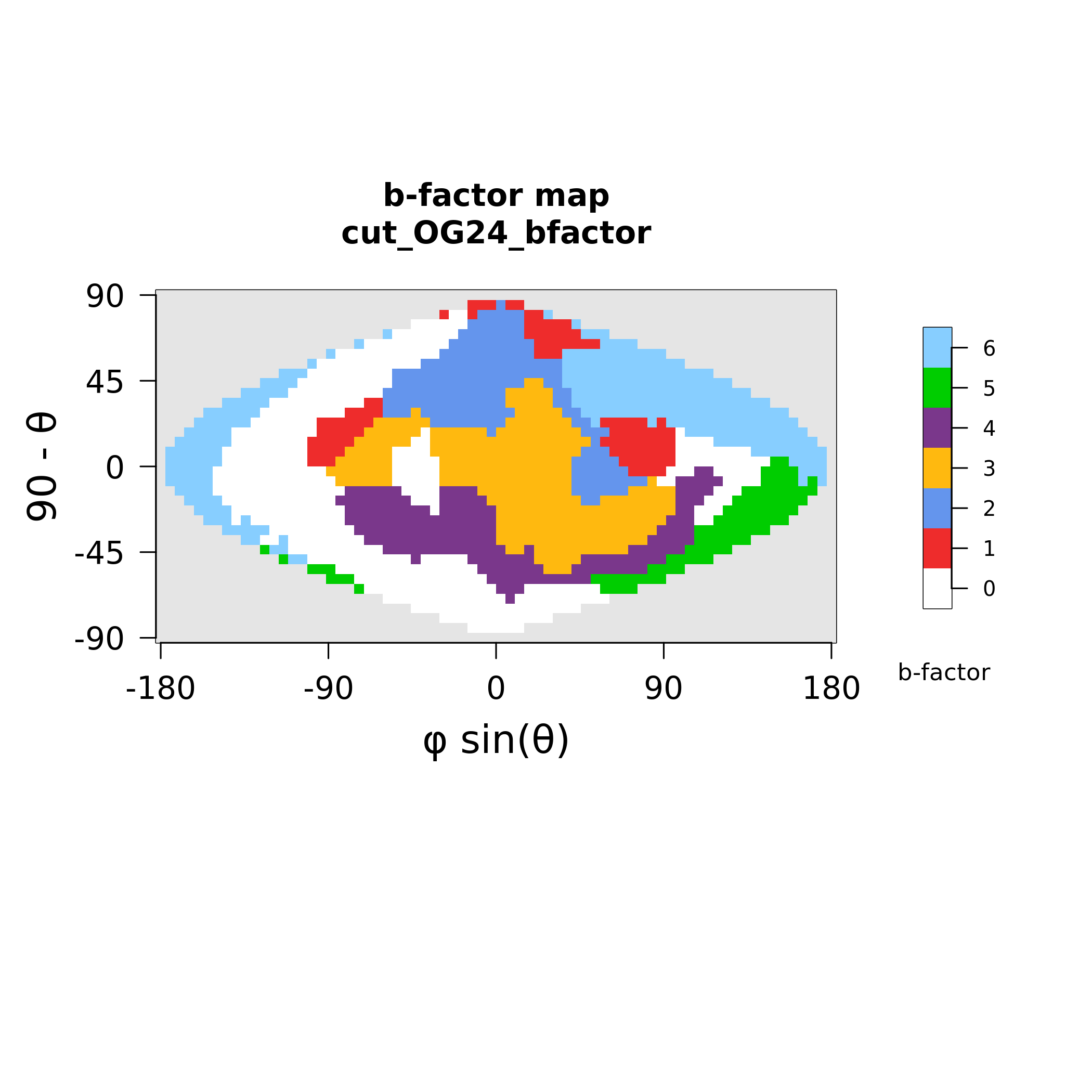

Supplement: S2 File — (ZIP) [file ppat.1012176.s019.zip › S2_File/STRANDS/MAX24_strands.png]

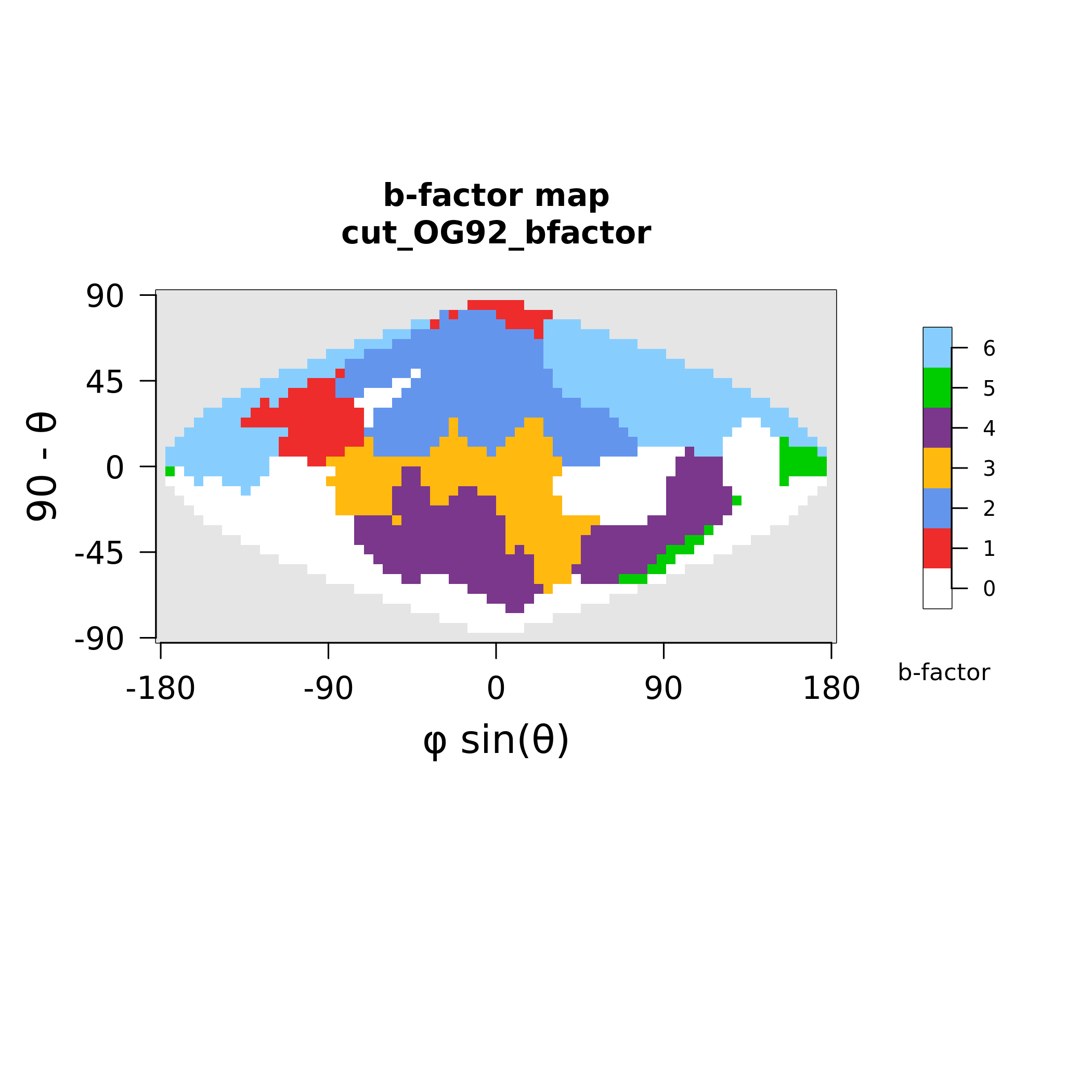

Supplement: S2 File — (ZIP) [file ppat.1012176.s019.zip › S2_File/STRANDS/MAX92_strands.png]

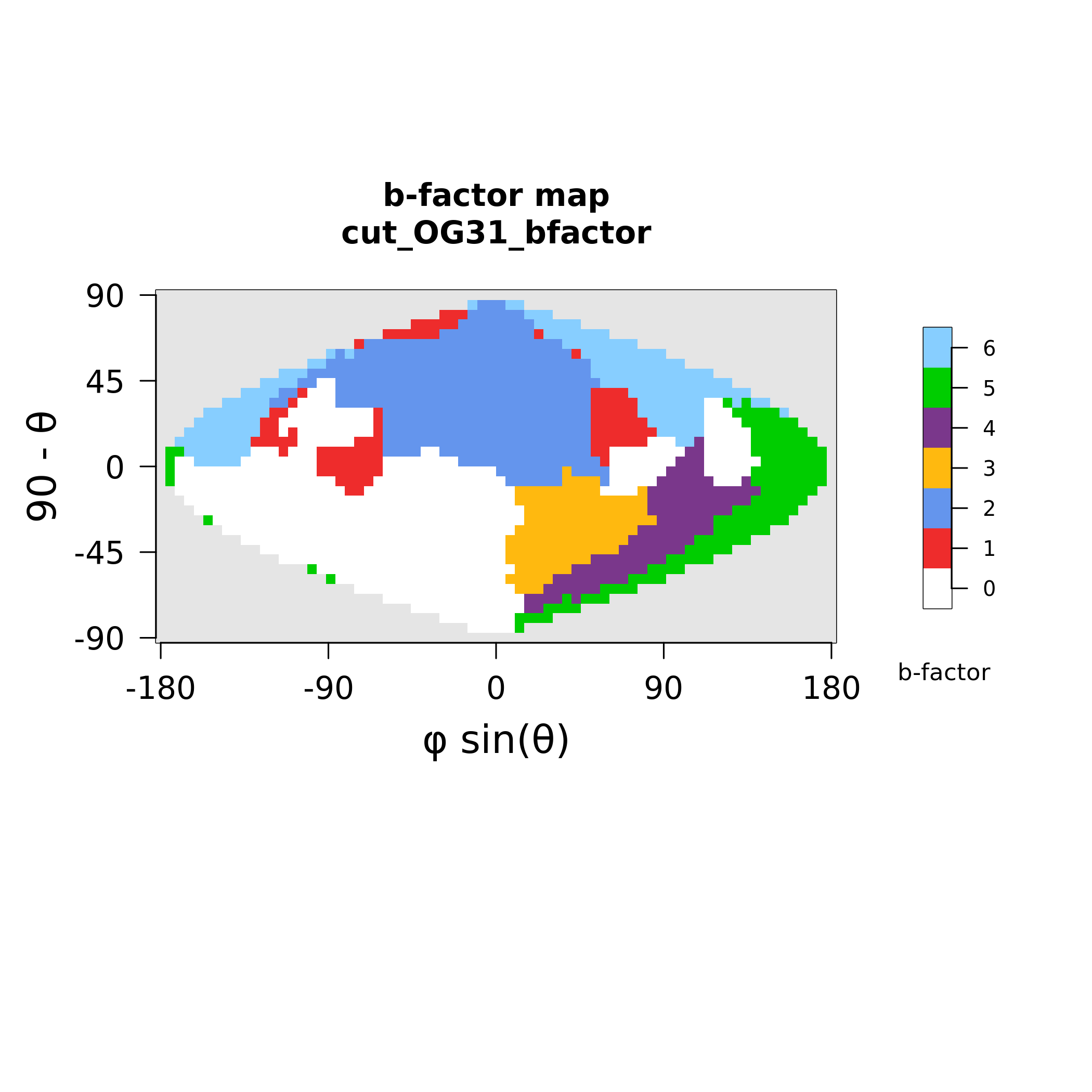

Supplement: S2 File — (ZIP) [file ppat.1012176.s019.zip › S2_File/STRANDS/MAX31_strands.png]

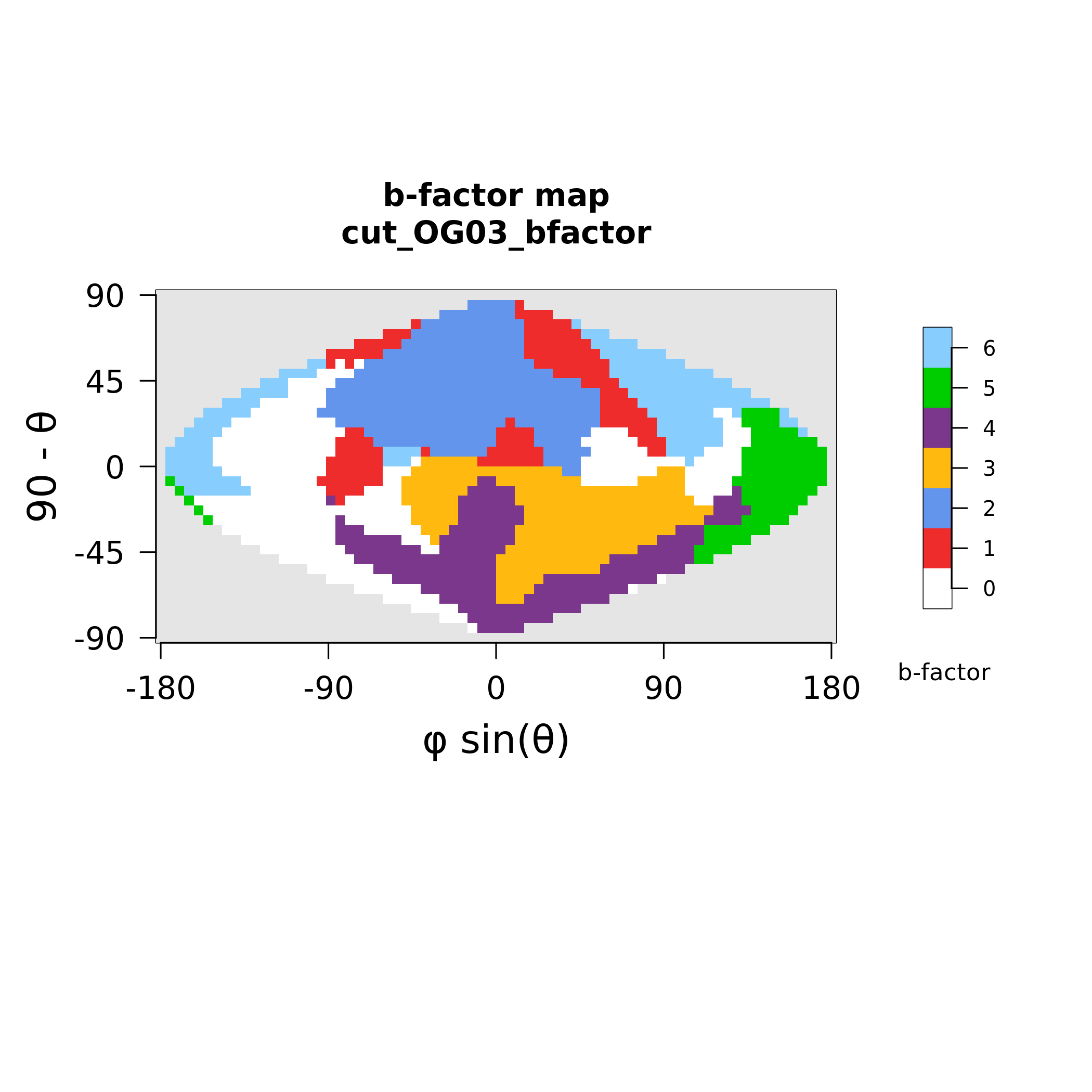

Supplement: S2 File — (ZIP) [file ppat.1012176.s019.zip › S2_File/STRANDS/MAX03_strands.png]

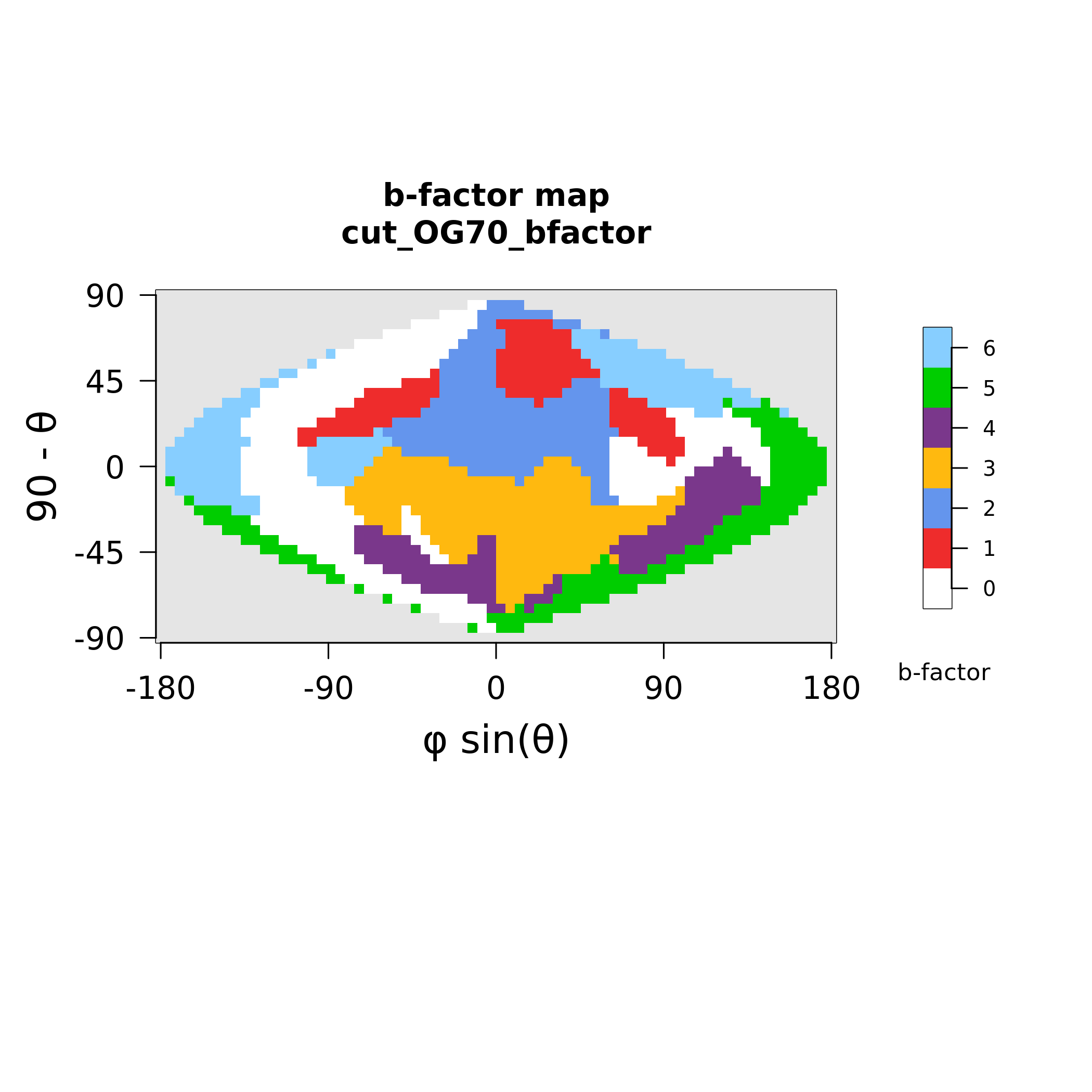

Supplement: S2 File — (ZIP) [file ppat.1012176.s019.zip › S2_File/STRANDS/MAX70_strands.png]

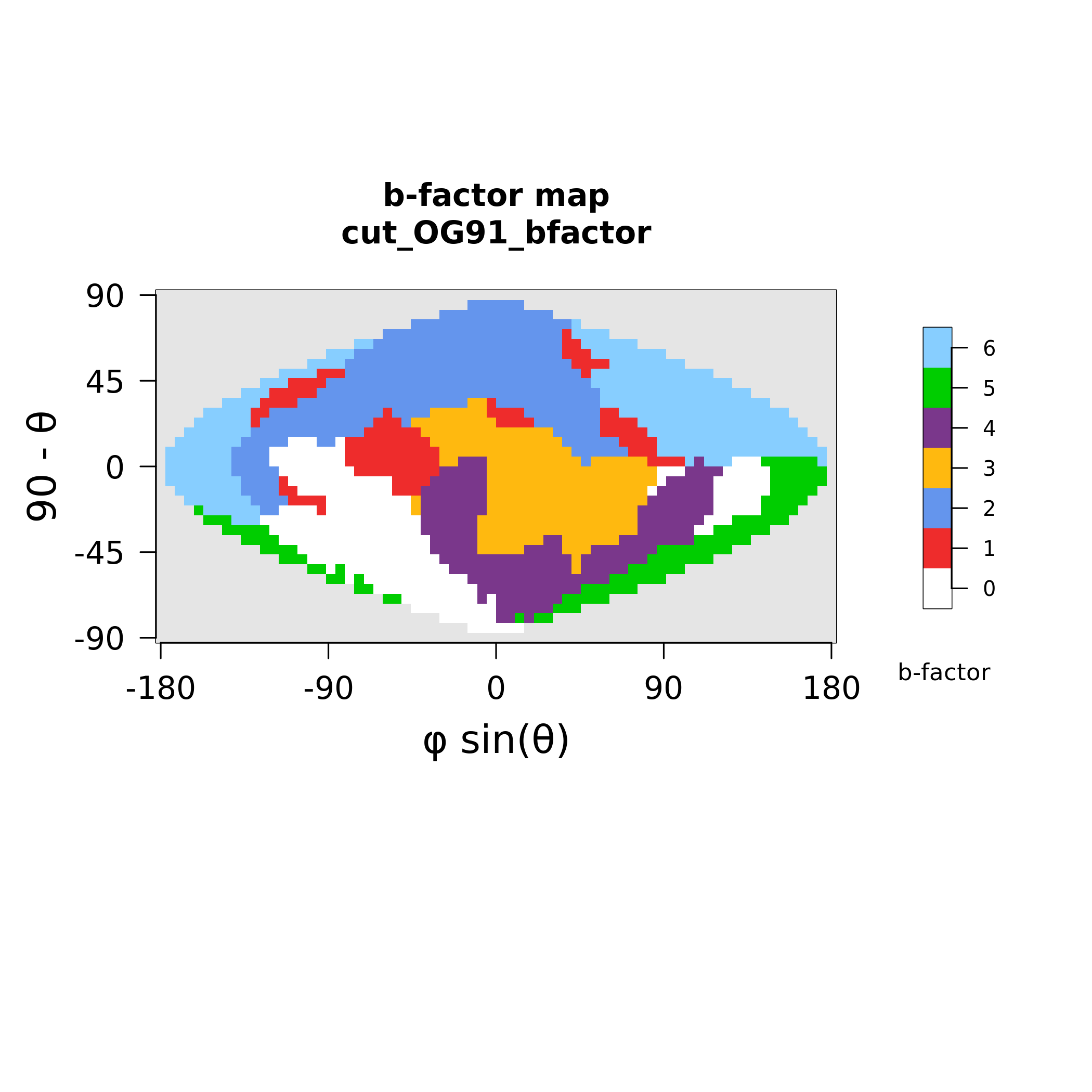

Supplement: S2 File — (ZIP) [file ppat.1012176.s019.zip › S2_File/STRANDS/MAX91_strands.png]

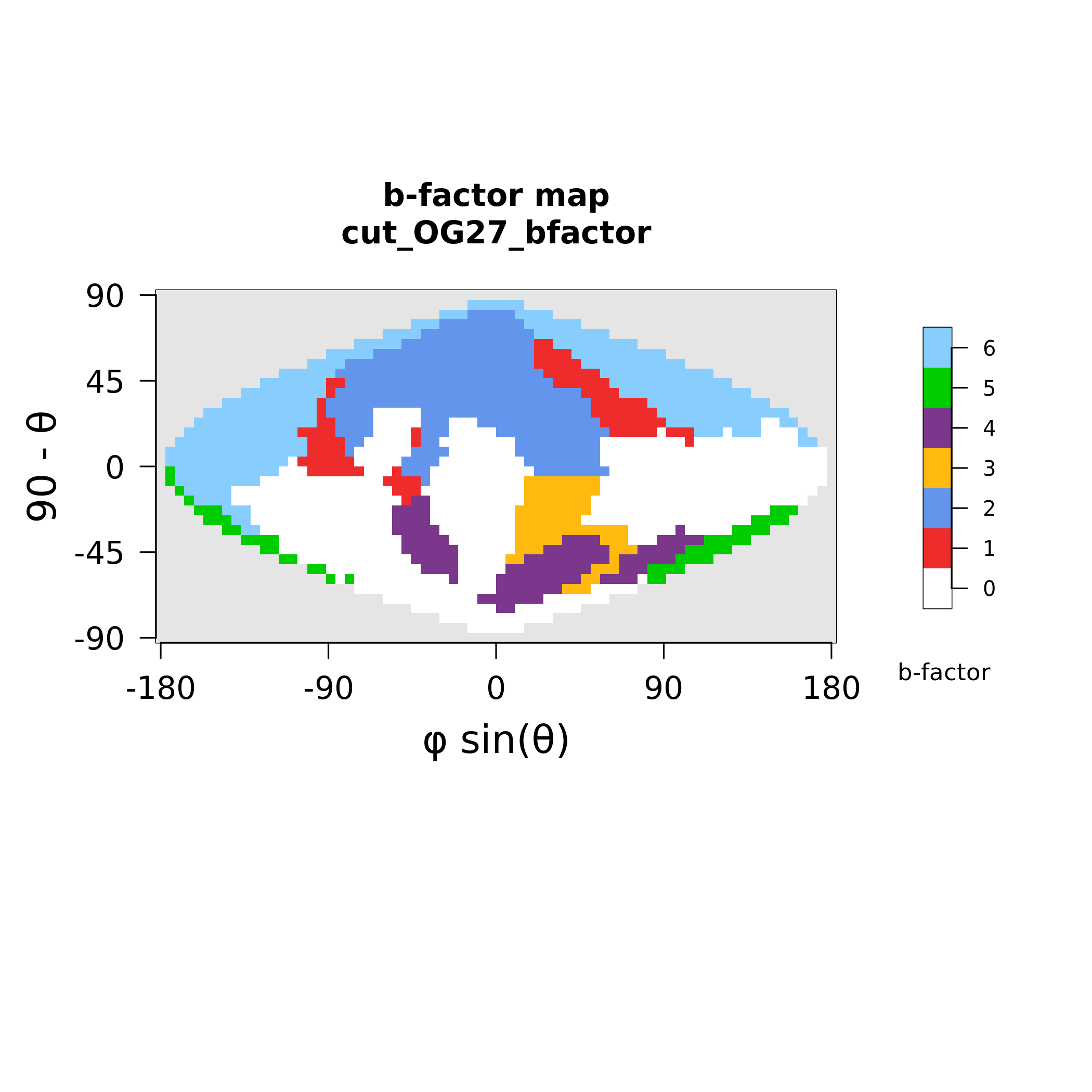

Supplement: S2 File — (ZIP) [file ppat.1012176.s019.zip › S2_File/STRANDS/MAX27_strands.png]

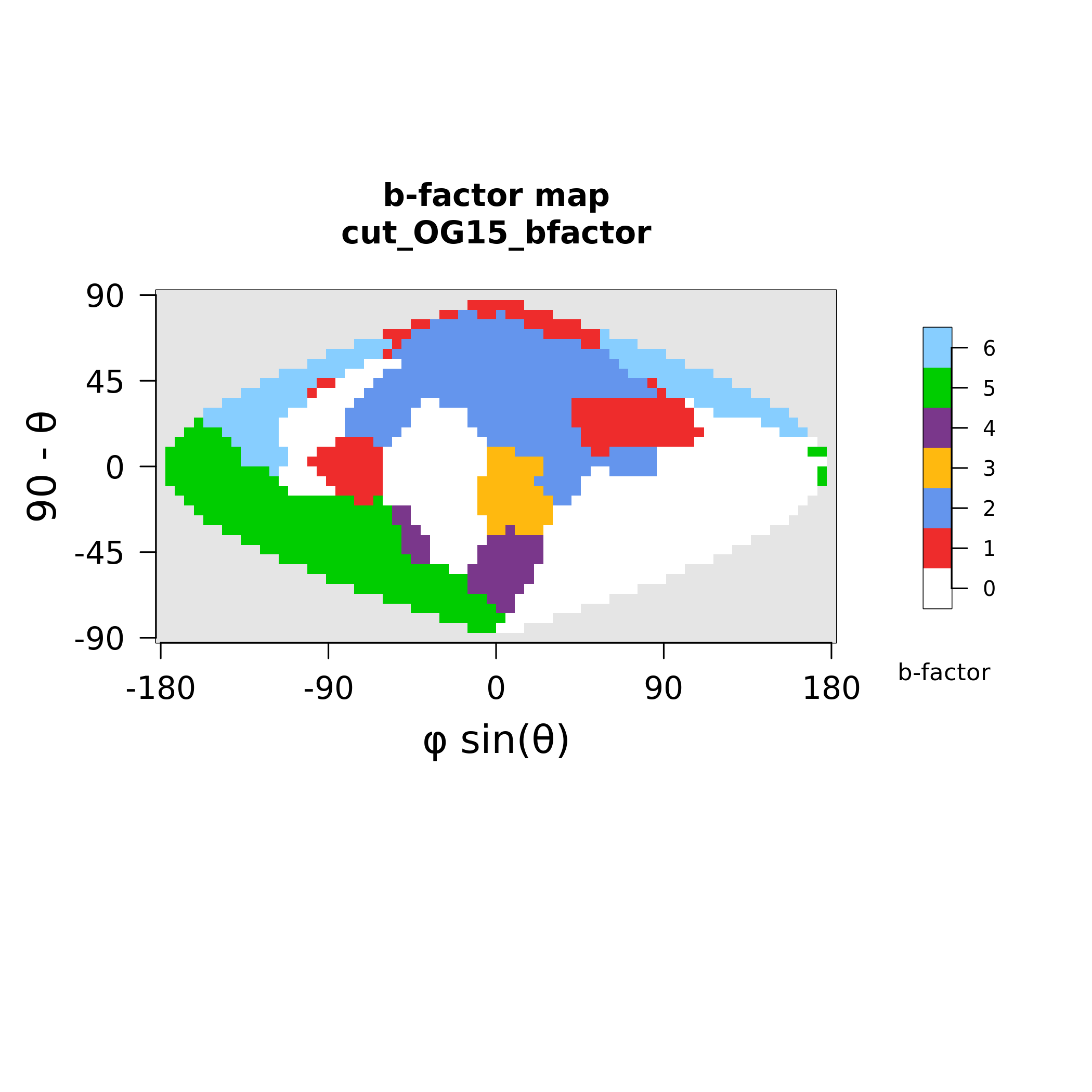

Supplement: S2 File — (ZIP) [file ppat.1012176.s019.zip › S2_File/STRANDS/MAX15_strands.png]

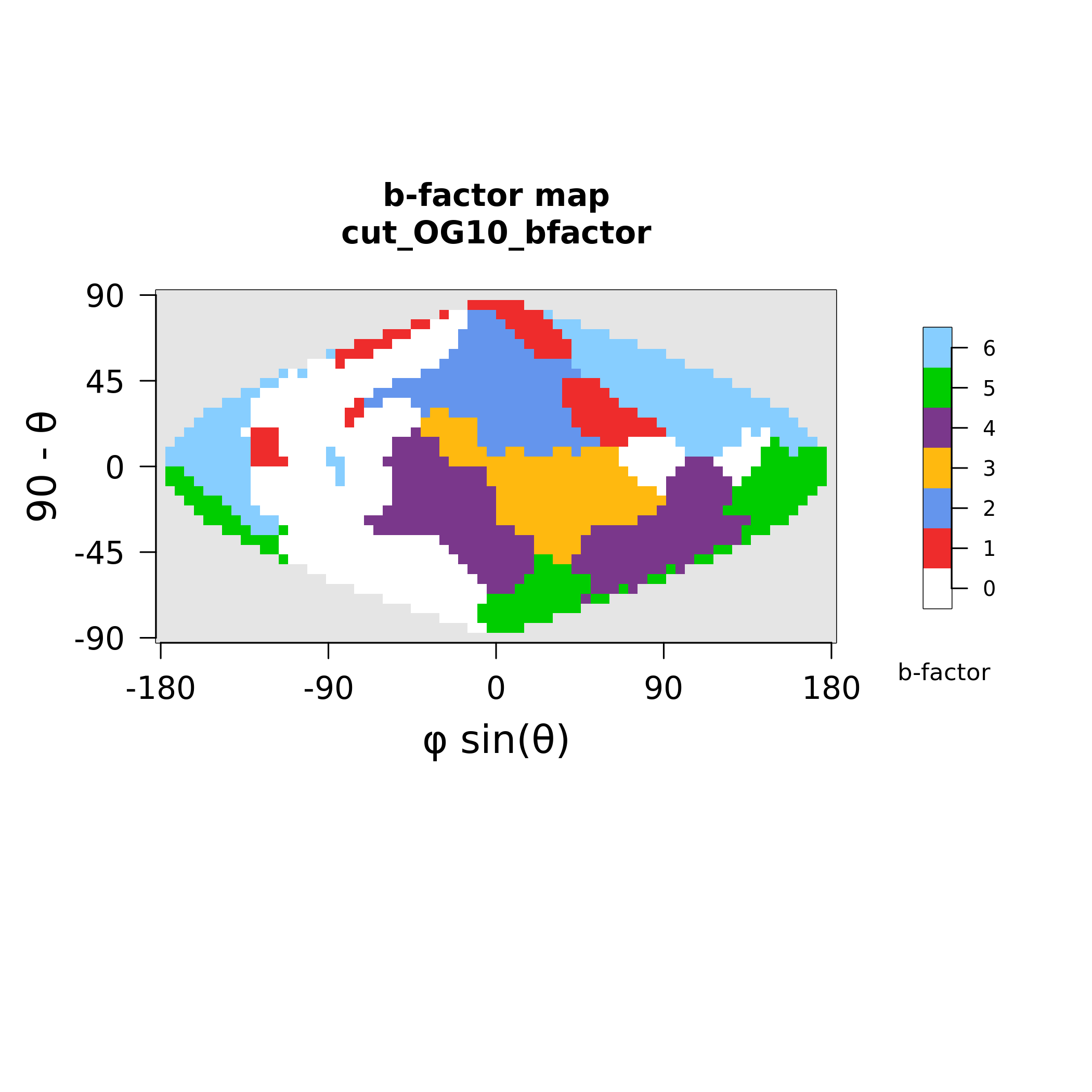

Supplement: S2 File — (ZIP) [file ppat.1012176.s019.zip › S2_File/STRANDS/MAX10_strands.png]

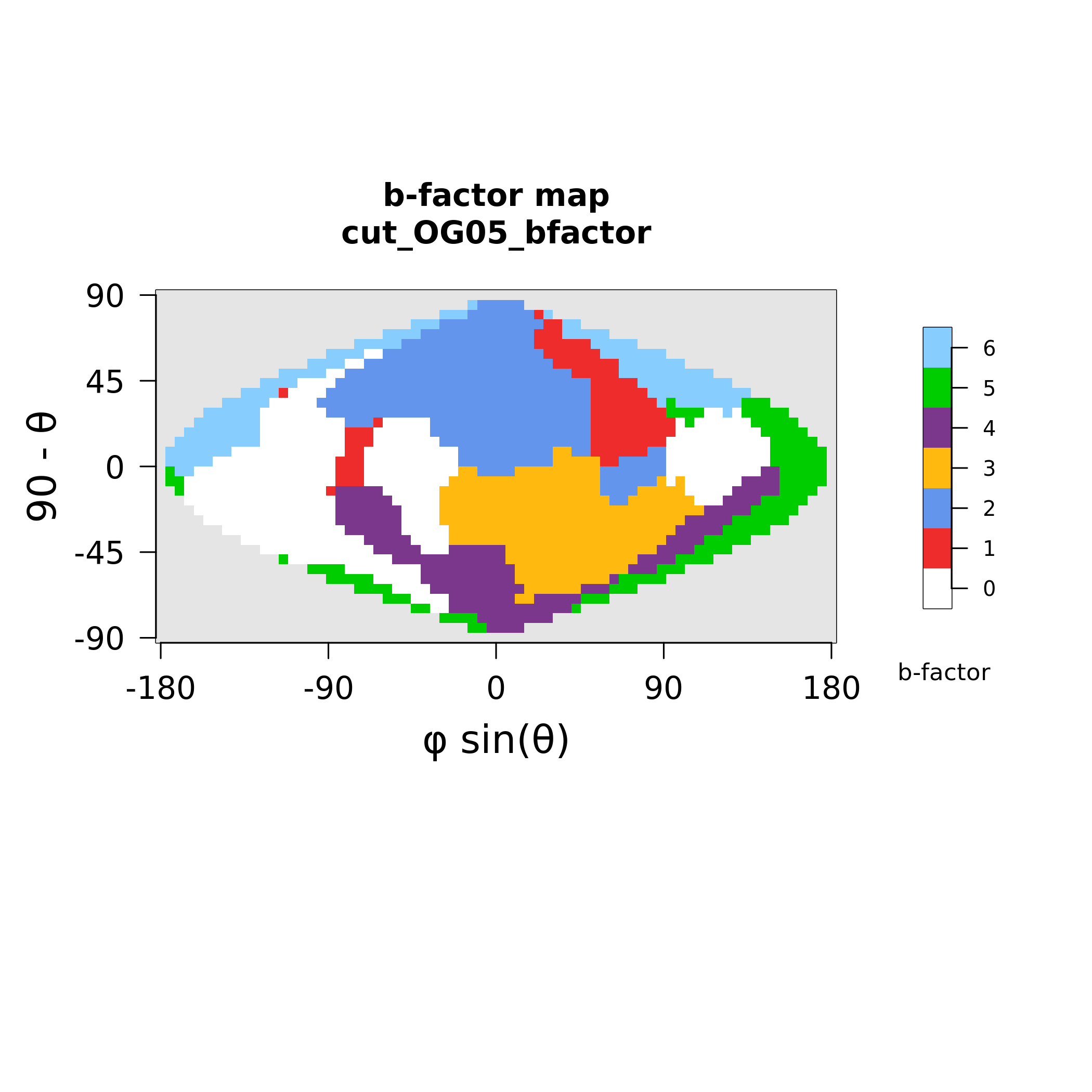

Supplement: S2 File — (ZIP) [file ppat.1012176.s019.zip › S2_File/STRANDS/MAX05_strands.png]

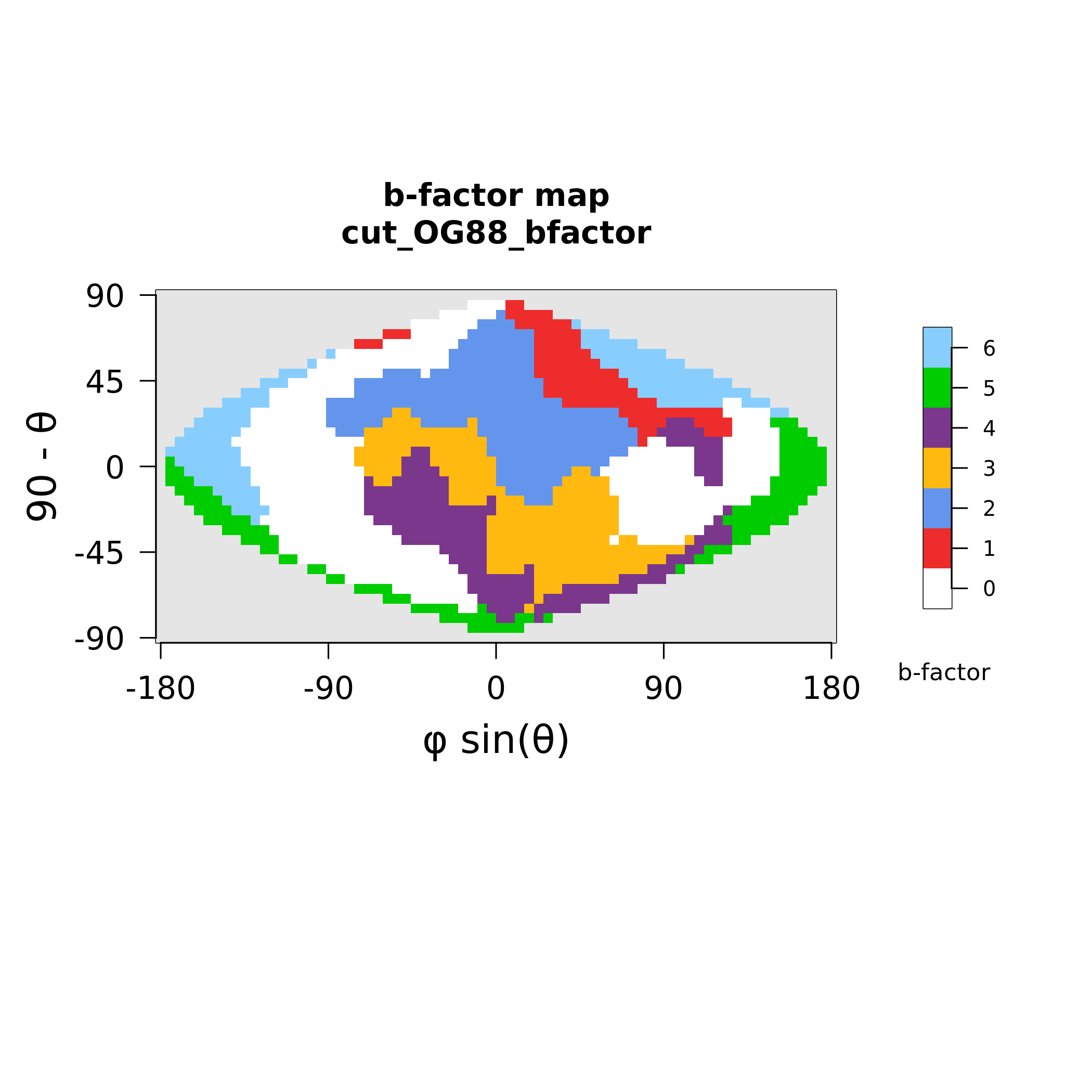

Supplement: S2 File — (ZIP) [file ppat.1012176.s019.zip › S2_File/STRANDS/MAX88_strands.png]

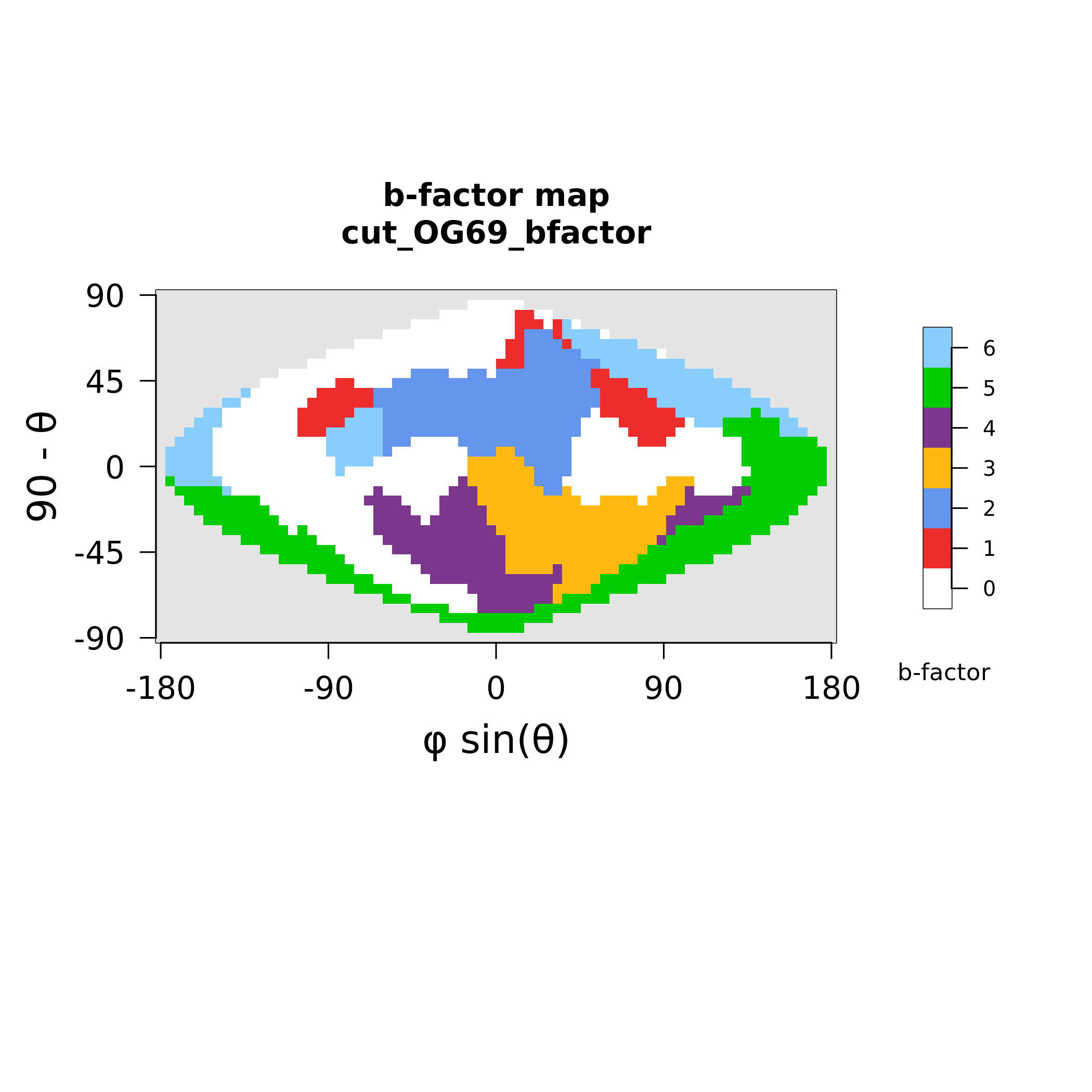

Supplement: S2 File — (ZIP) [file ppat.1012176.s019.zip › S2_File/STRANDS/MAX69_strands.png]

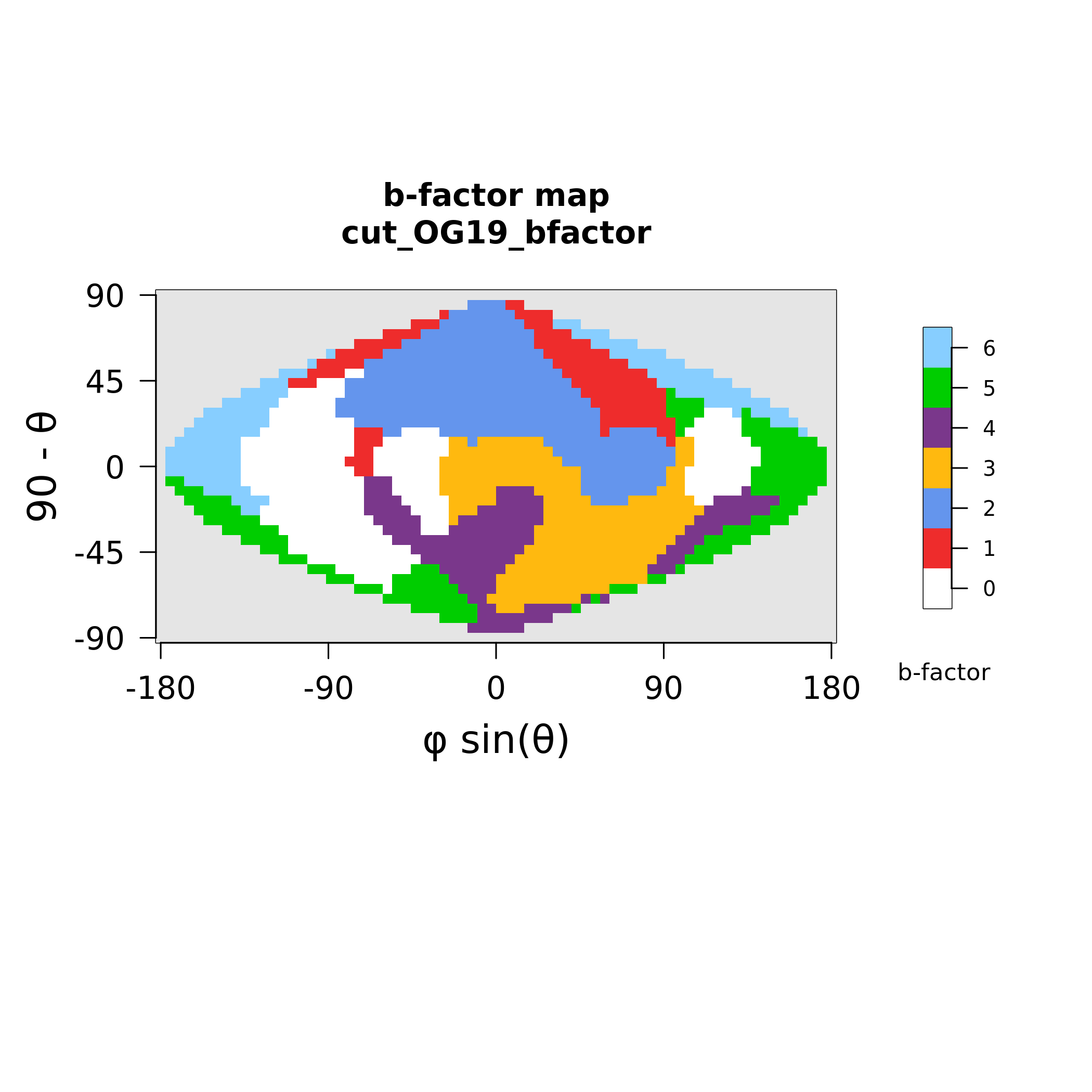

Supplement: S2 File — (ZIP) [file ppat.1012176.s019.zip › S2_File/STRANDS/MAX19_strands.png]

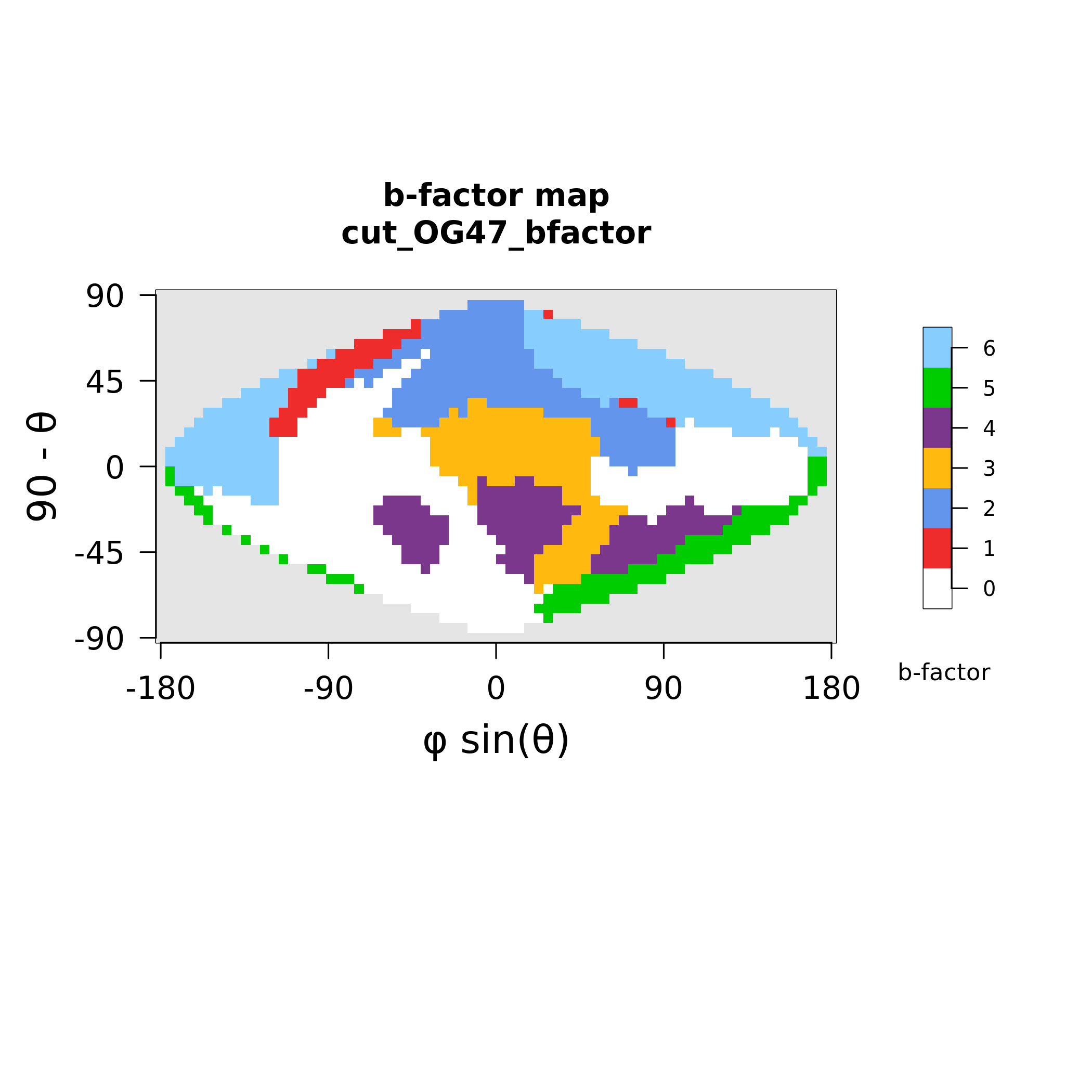

Supplement: S2 File — (ZIP) [file ppat.1012176.s019.zip › S2_File/STRANDS/MAX47_strands.png]

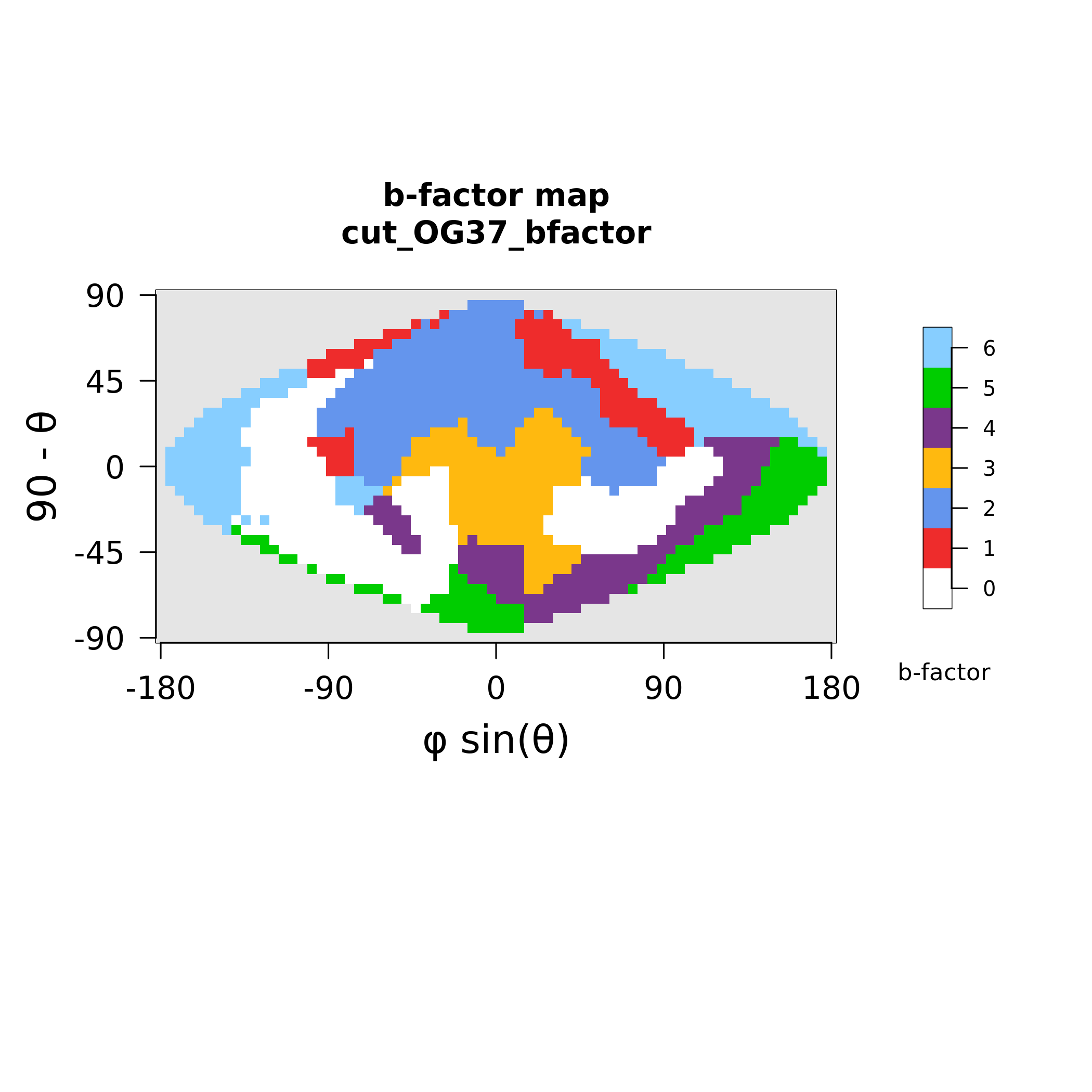

Supplement: S2 File — (ZIP) [file ppat.1012176.s019.zip › S2_File/STRANDS/MAX37_strands.png]
