# Supplementary figures and images for: The structural landscape and diversity of Pyricularia oryzae MAX effectors revisited (part 1 of 2)
Source: PLoS Pathog. 2024 May 6;20(5):e1012176. doi: 10.1371/journal.ppat.1012176 (PMC11132498; doi:10.1371/journal.ppat.1012176)

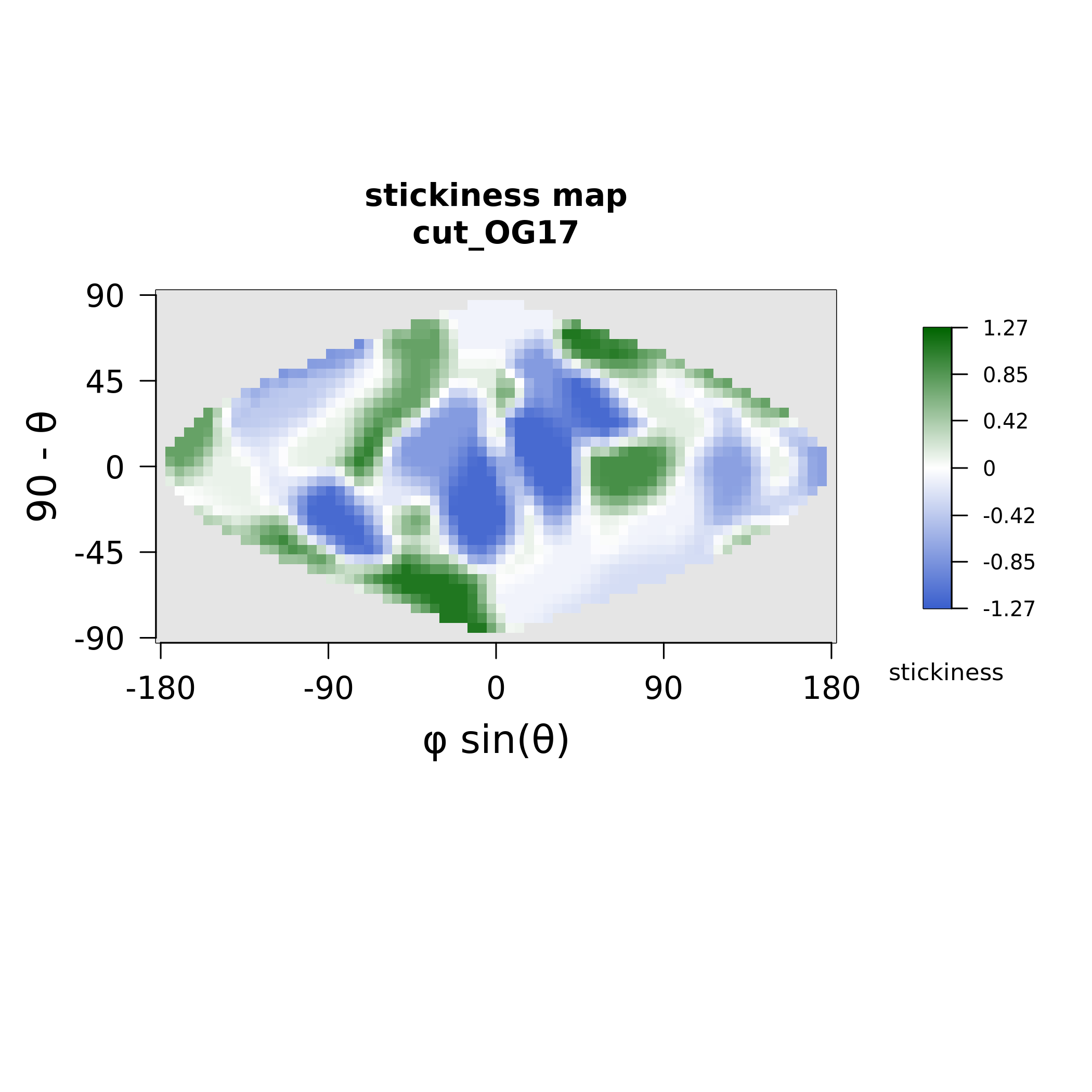

Supplement: S2 File — (ZIP) [file ppat.1012176.s019.zip › S2_File/STICKINESS/MAX17_stickiness.png]

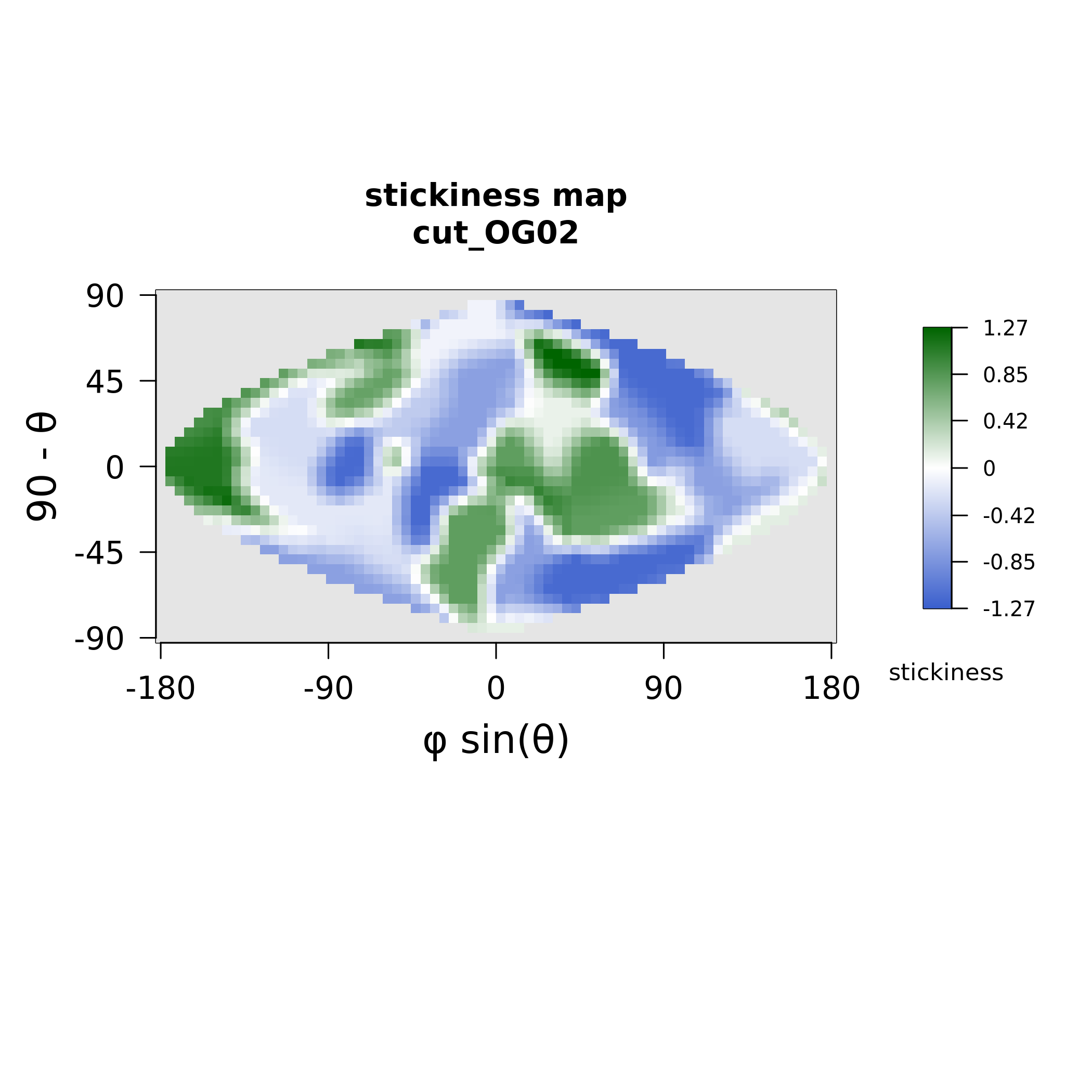

Supplement: S2 File — (ZIP) [file ppat.1012176.s019.zip › S2_File/STICKINESS/MAX02_stickiness.png]

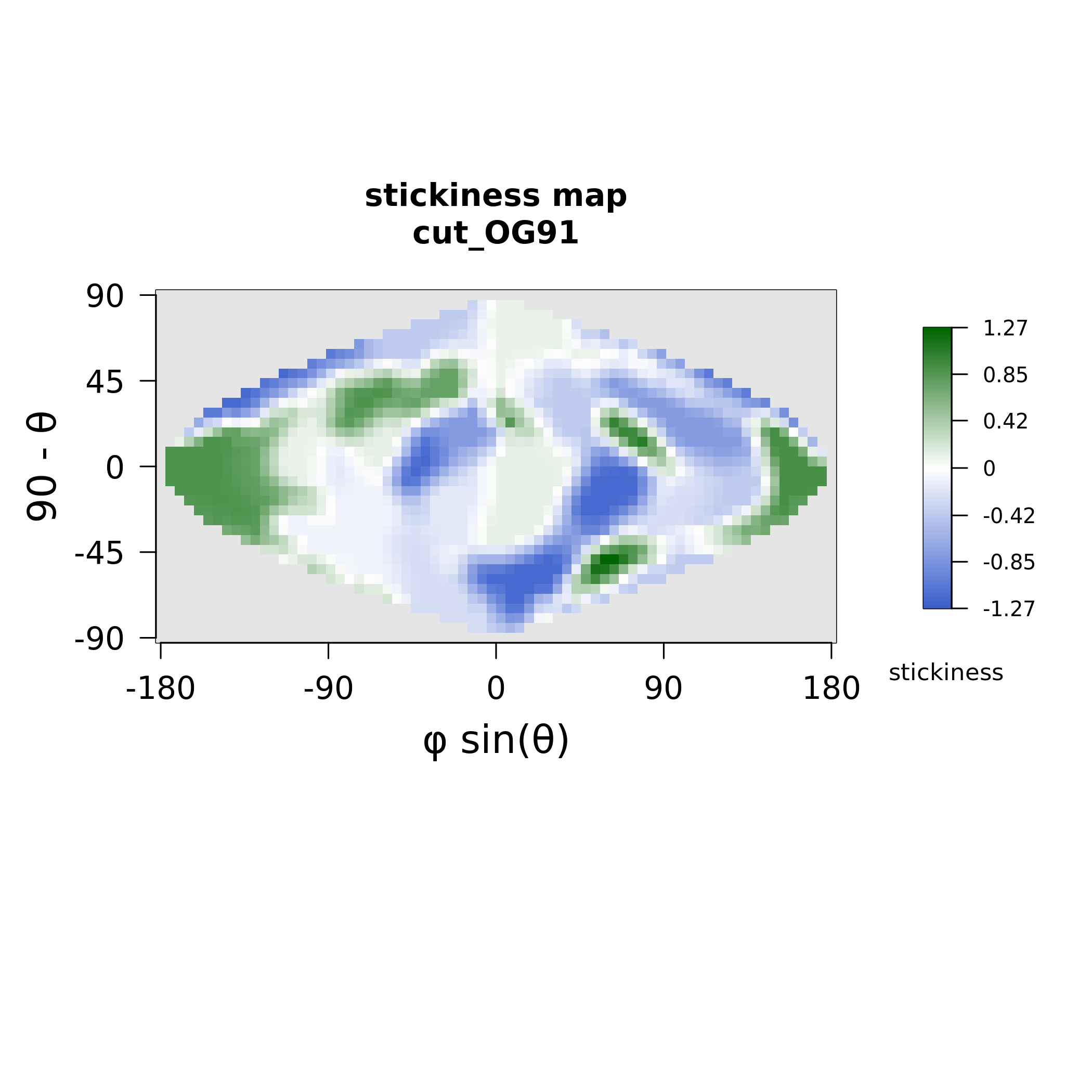

Supplement: S2 File — (ZIP) [file ppat.1012176.s019.zip › S2_File/STICKINESS/MAX91_stickiness.png]

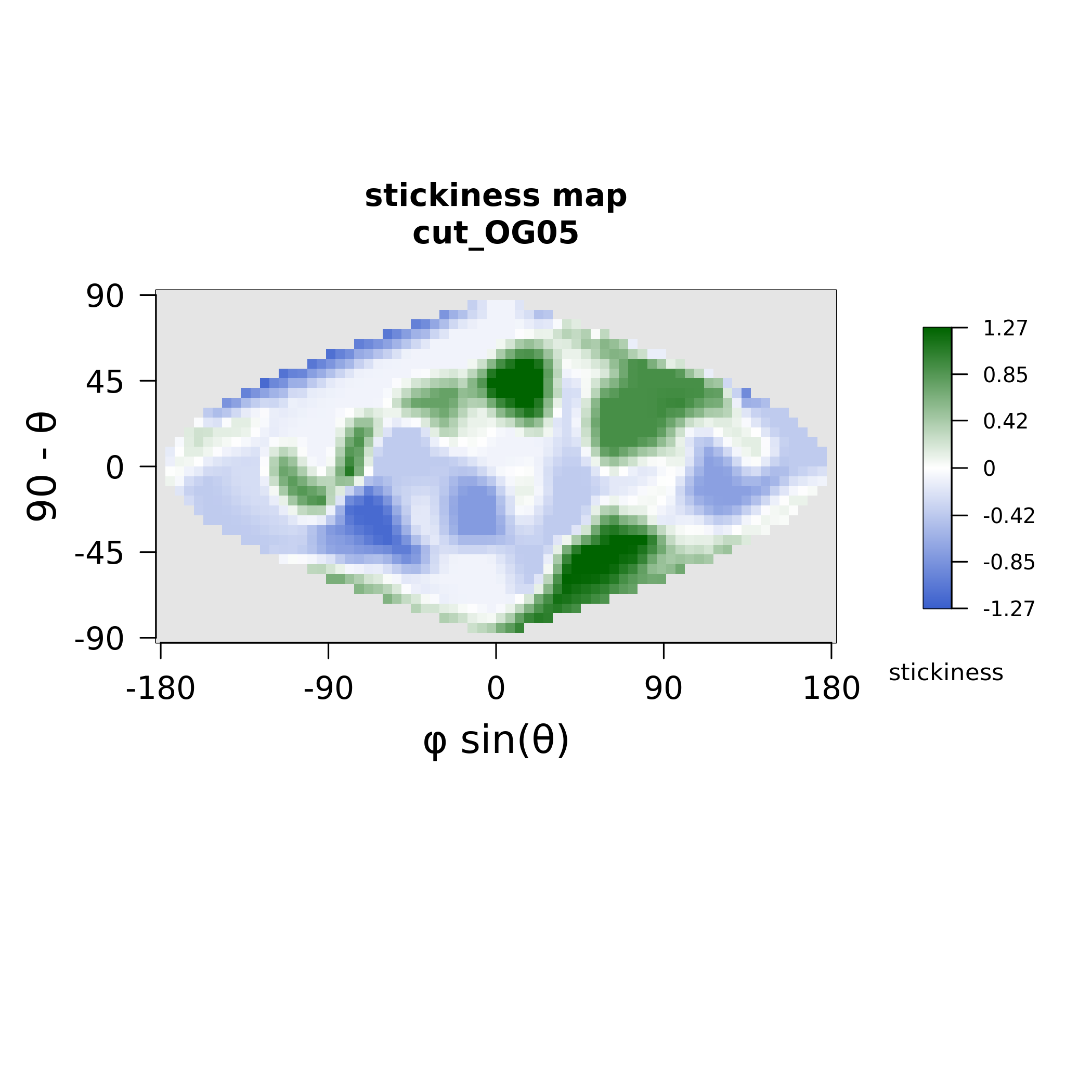

Supplement: S2 File — (ZIP) [file ppat.1012176.s019.zip › S2_File/STICKINESS/MAX05_stickiness.png]

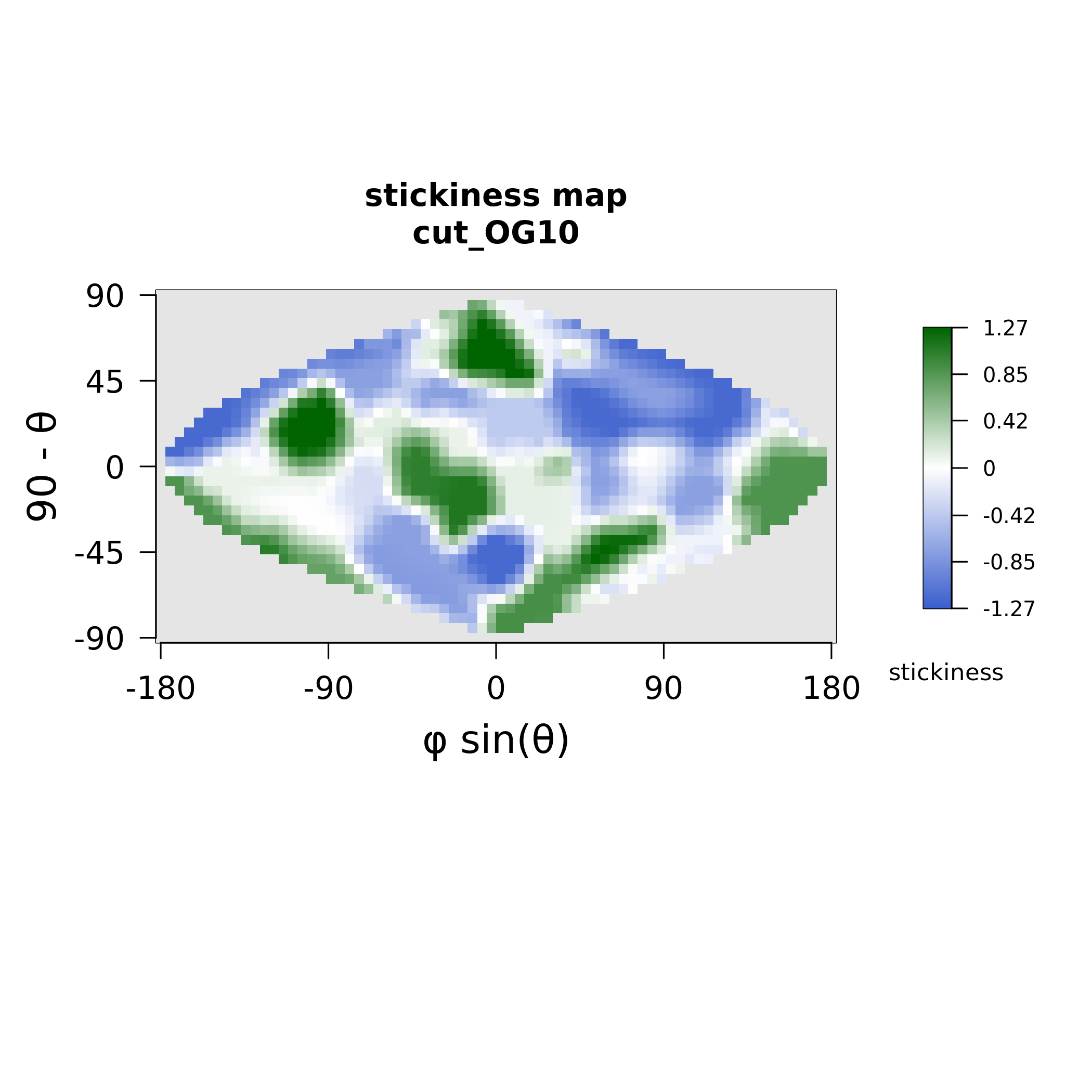

Supplement: S2 File — (ZIP) [file ppat.1012176.s019.zip › S2_File/STICKINESS/MAX10_stickiness.png]

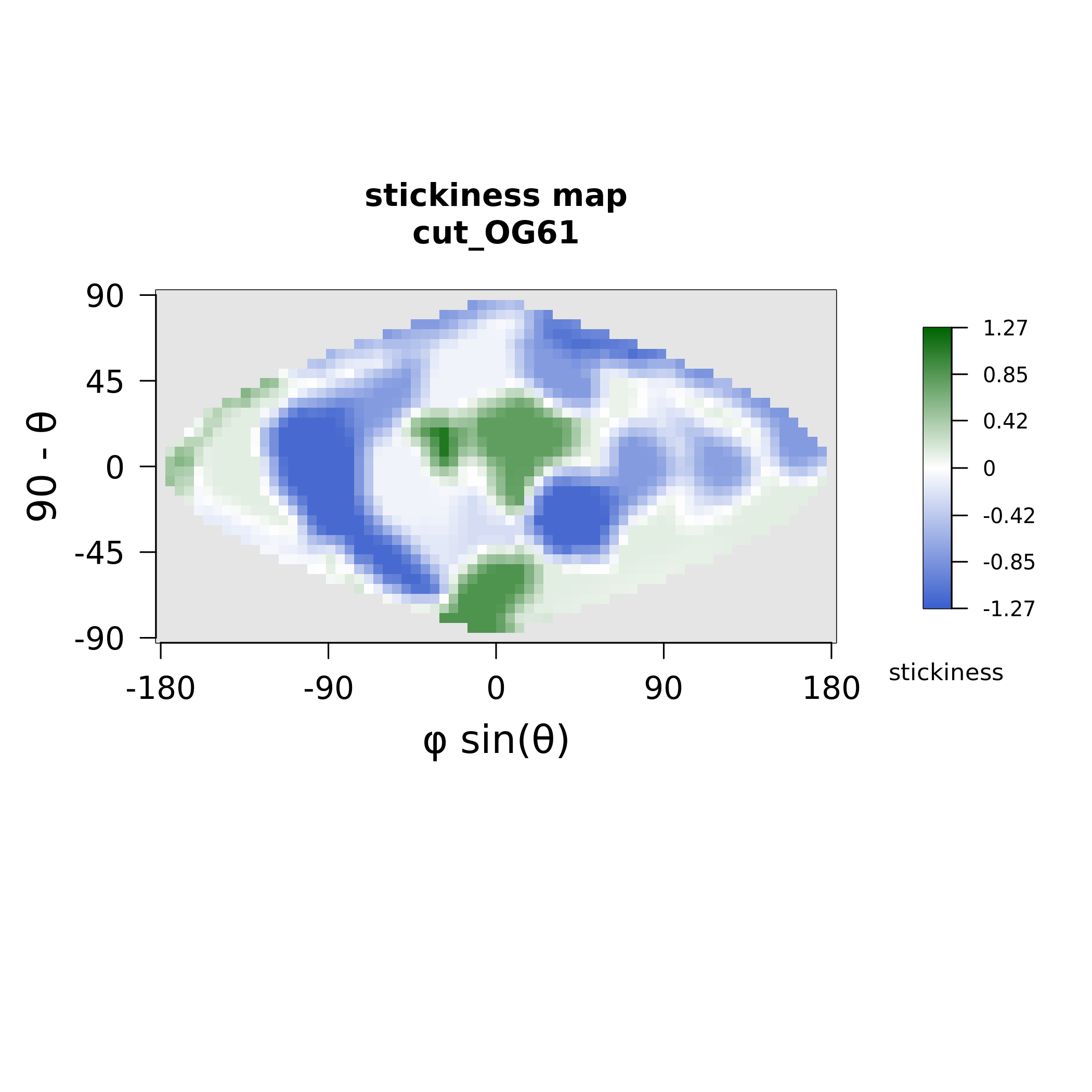

Supplement: S2 File — (ZIP) [file ppat.1012176.s019.zip › S2_File/STICKINESS/MAX61_stickiness.png]

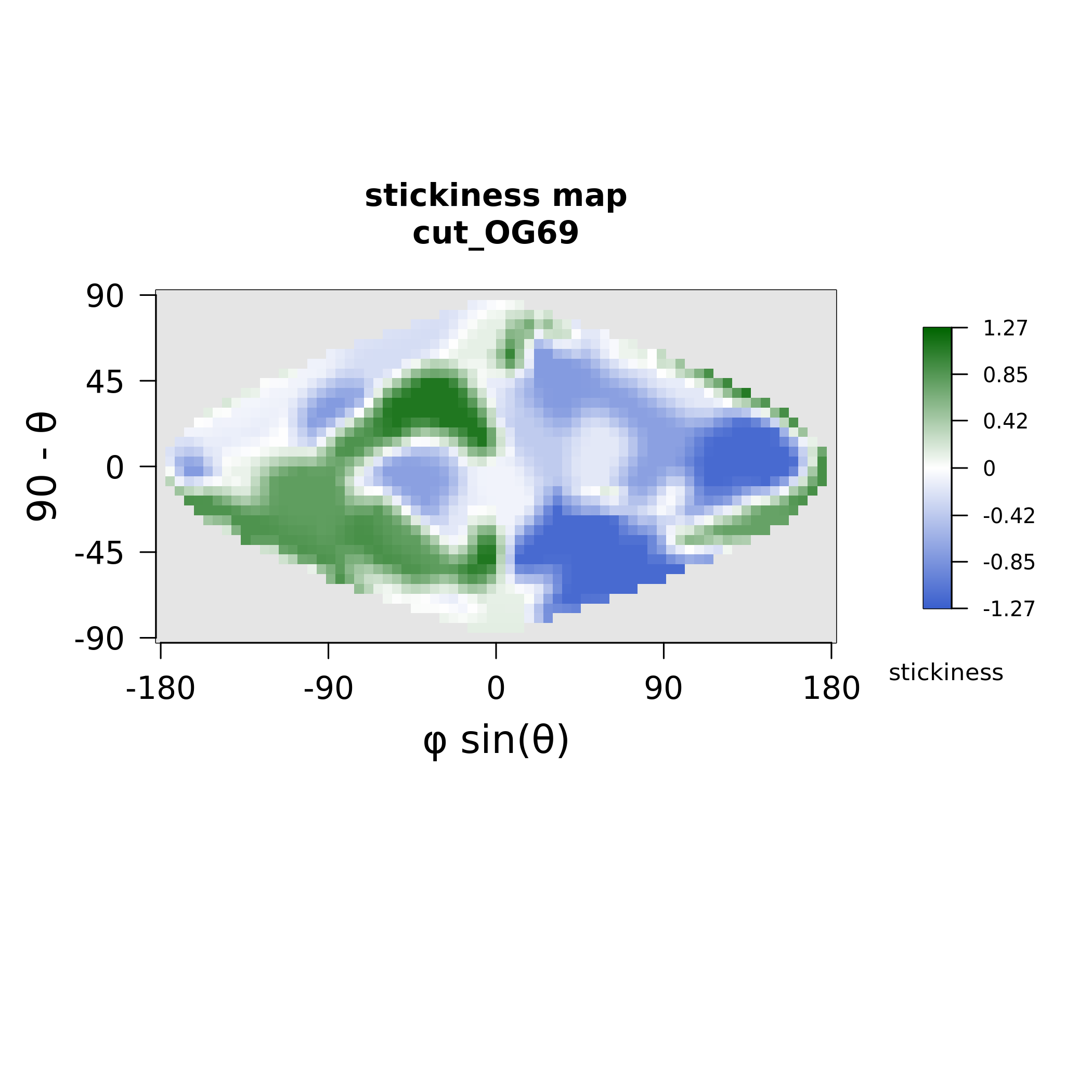

Supplement: S2 File — (ZIP) [file ppat.1012176.s019.zip › S2_File/STICKINESS/MAX69_stickiness.png]

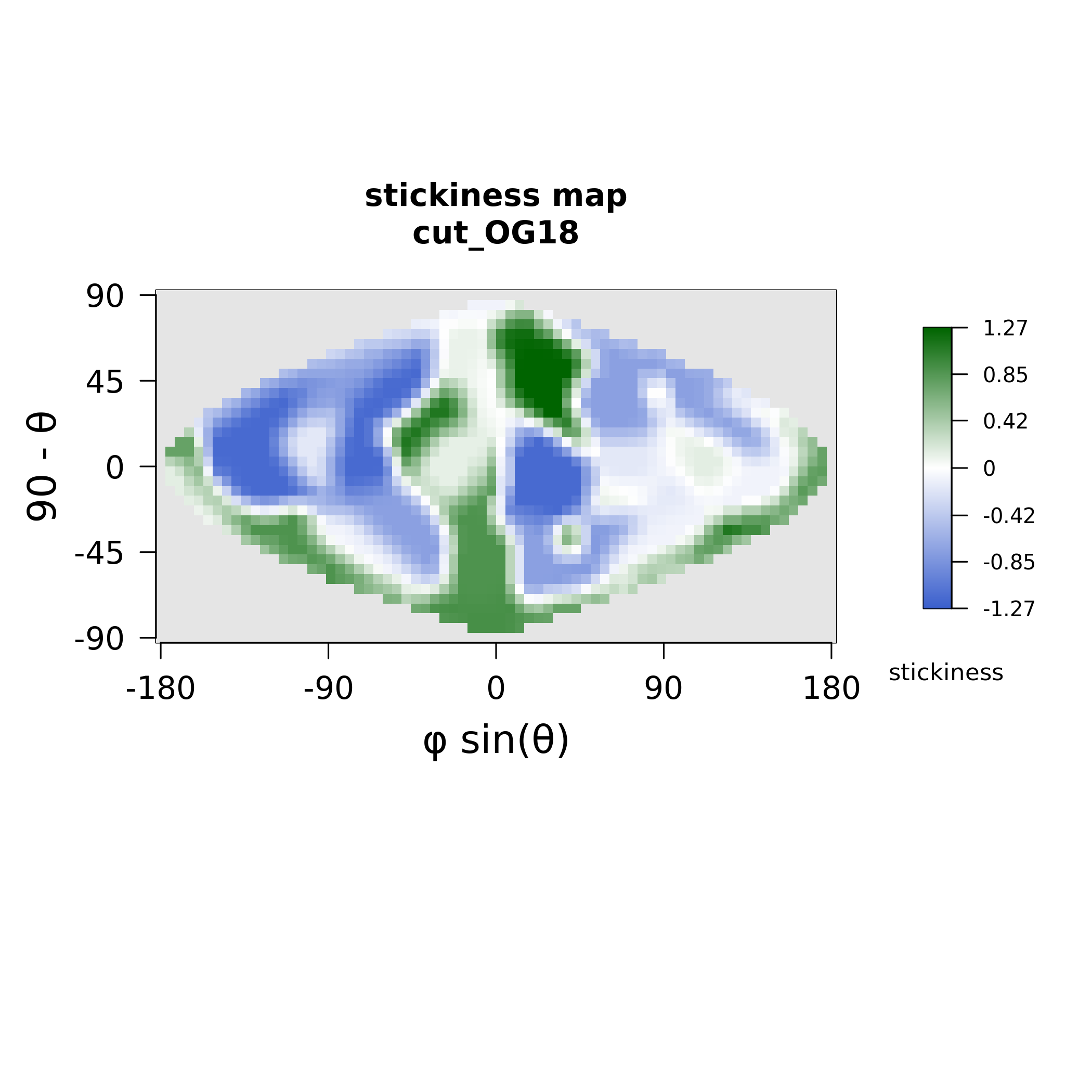

Supplement: S2 File — (ZIP) [file ppat.1012176.s019.zip › S2_File/STICKINESS/MAX18_stickiness.png]

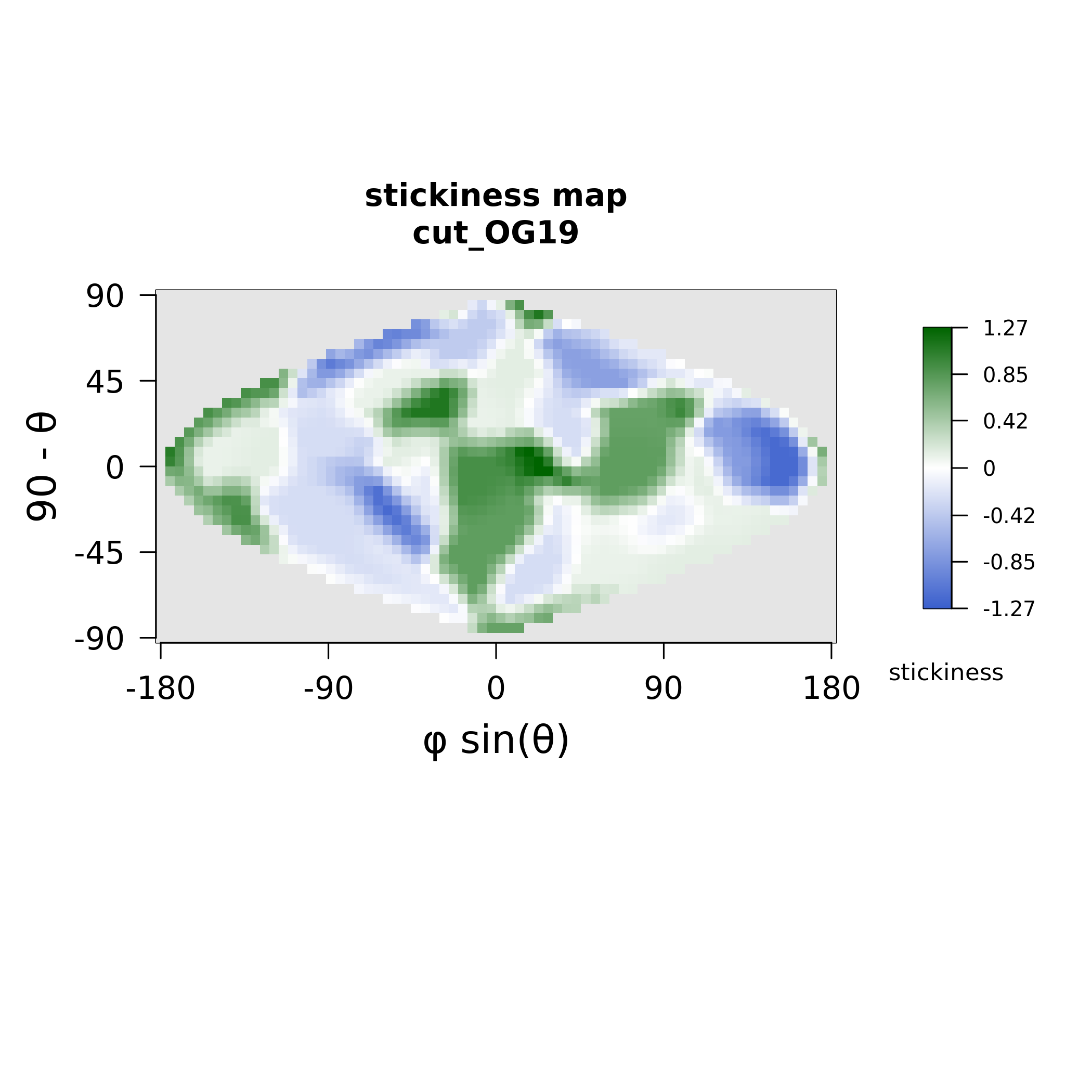

Supplement: S2 File — (ZIP) [file ppat.1012176.s019.zip › S2_File/STICKINESS/MAX19_stickiness.png]

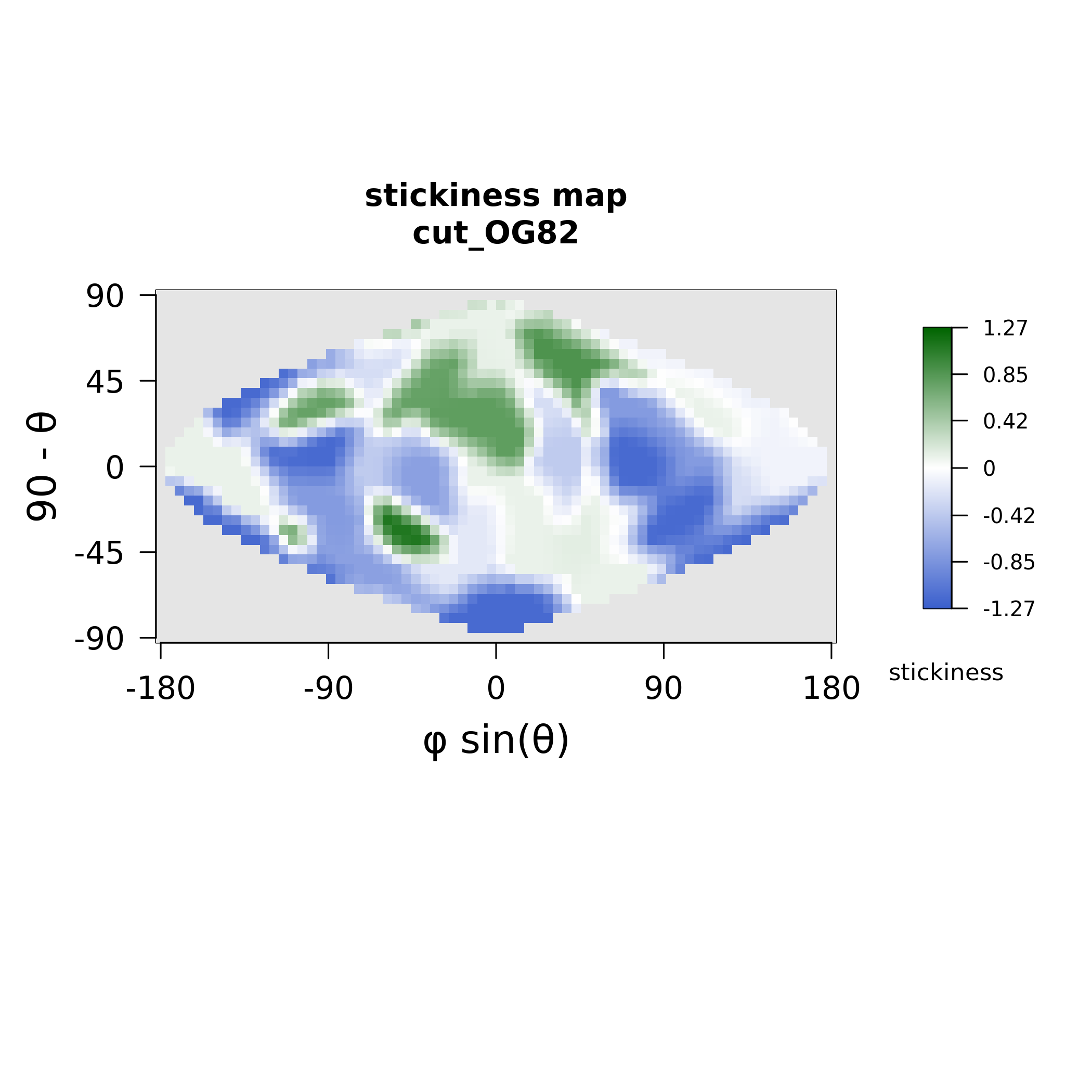

Supplement: S2 File — (ZIP) [file ppat.1012176.s019.zip › S2_File/STICKINESS/MAX82_stickiness.png]

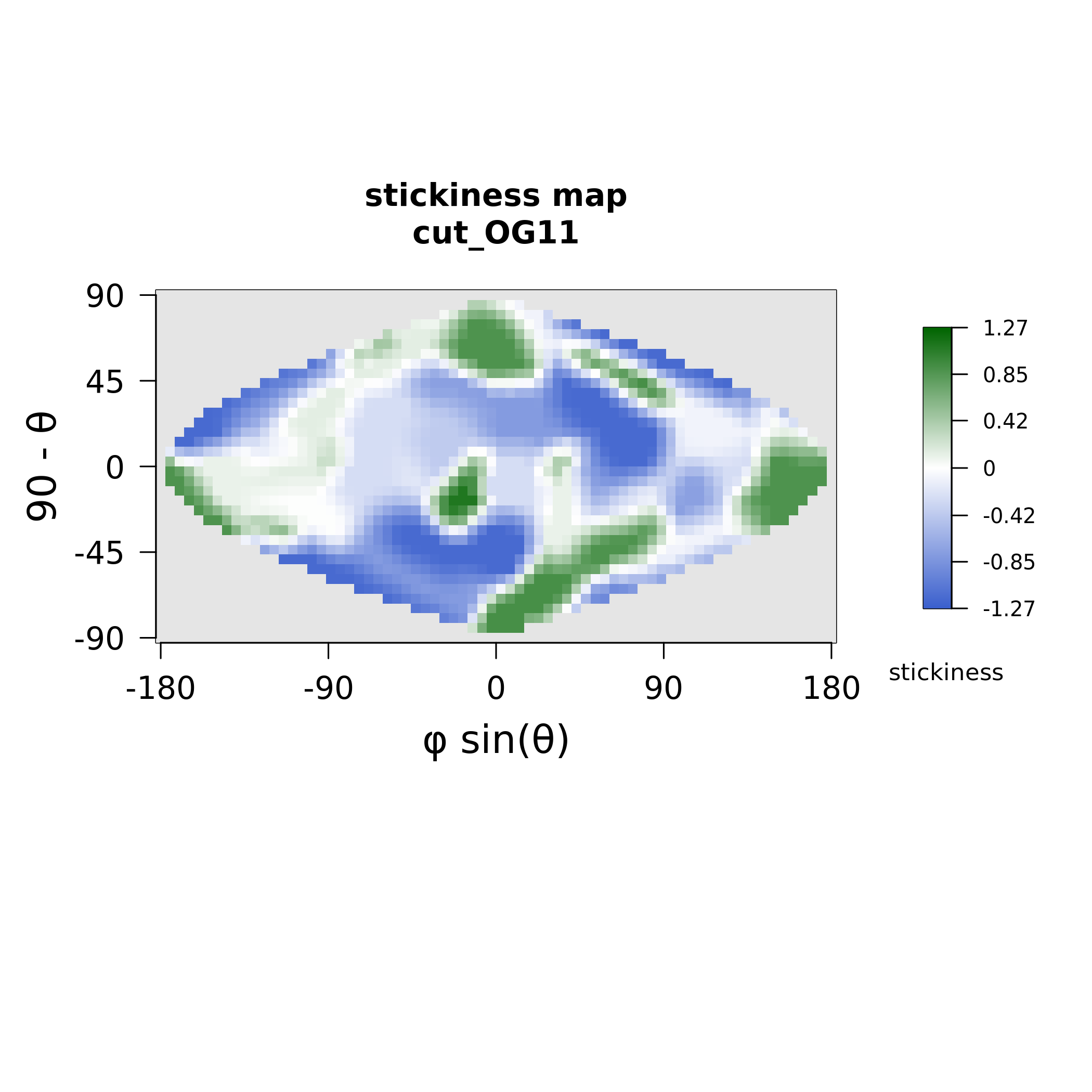

Supplement: S2 File — (ZIP) [file ppat.1012176.s019.zip › S2_File/STICKINESS/MAX11_stickiness.png]

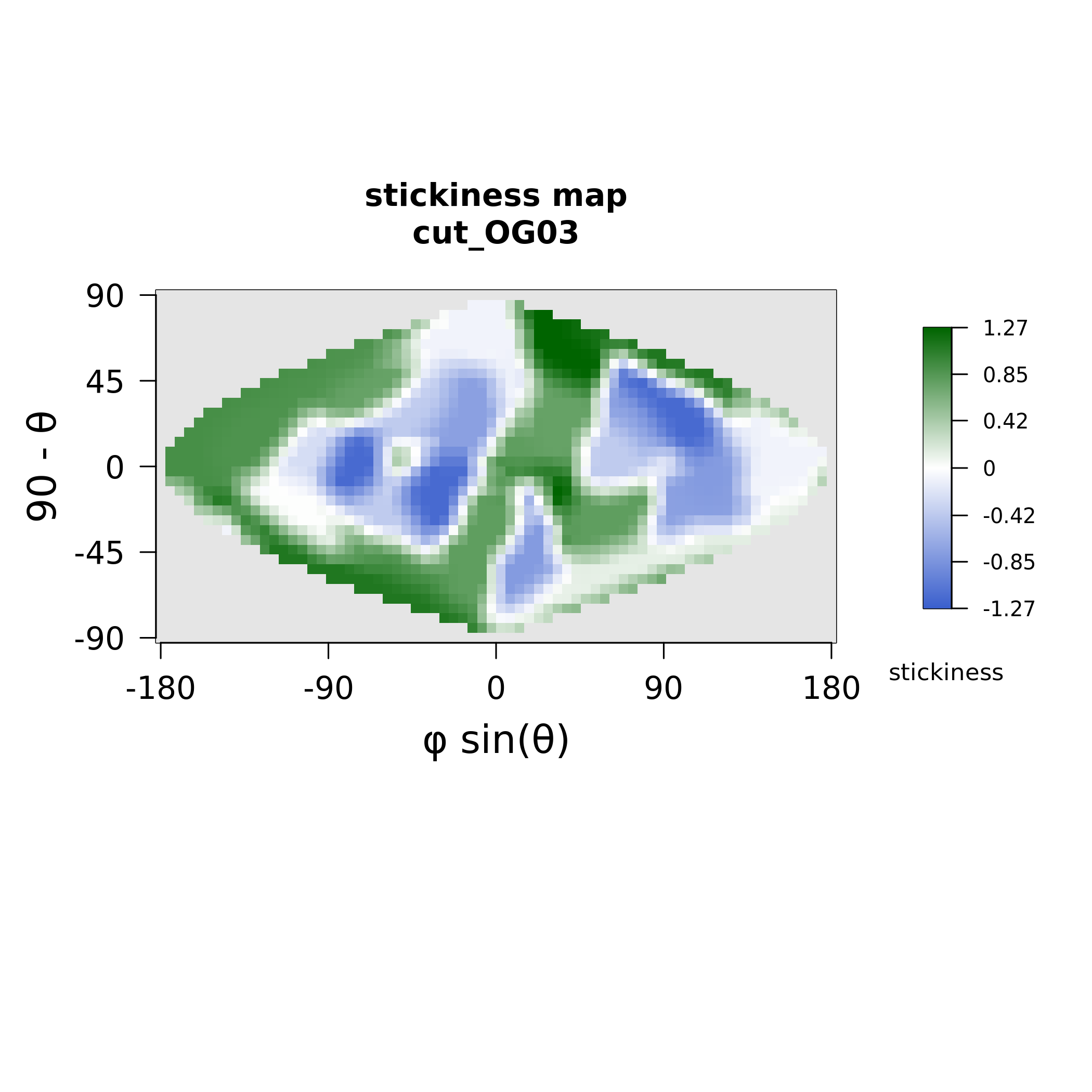

Supplement: S2 File — (ZIP) [file ppat.1012176.s019.zip › S2_File/STICKINESS/MAX03_stickiness.png]

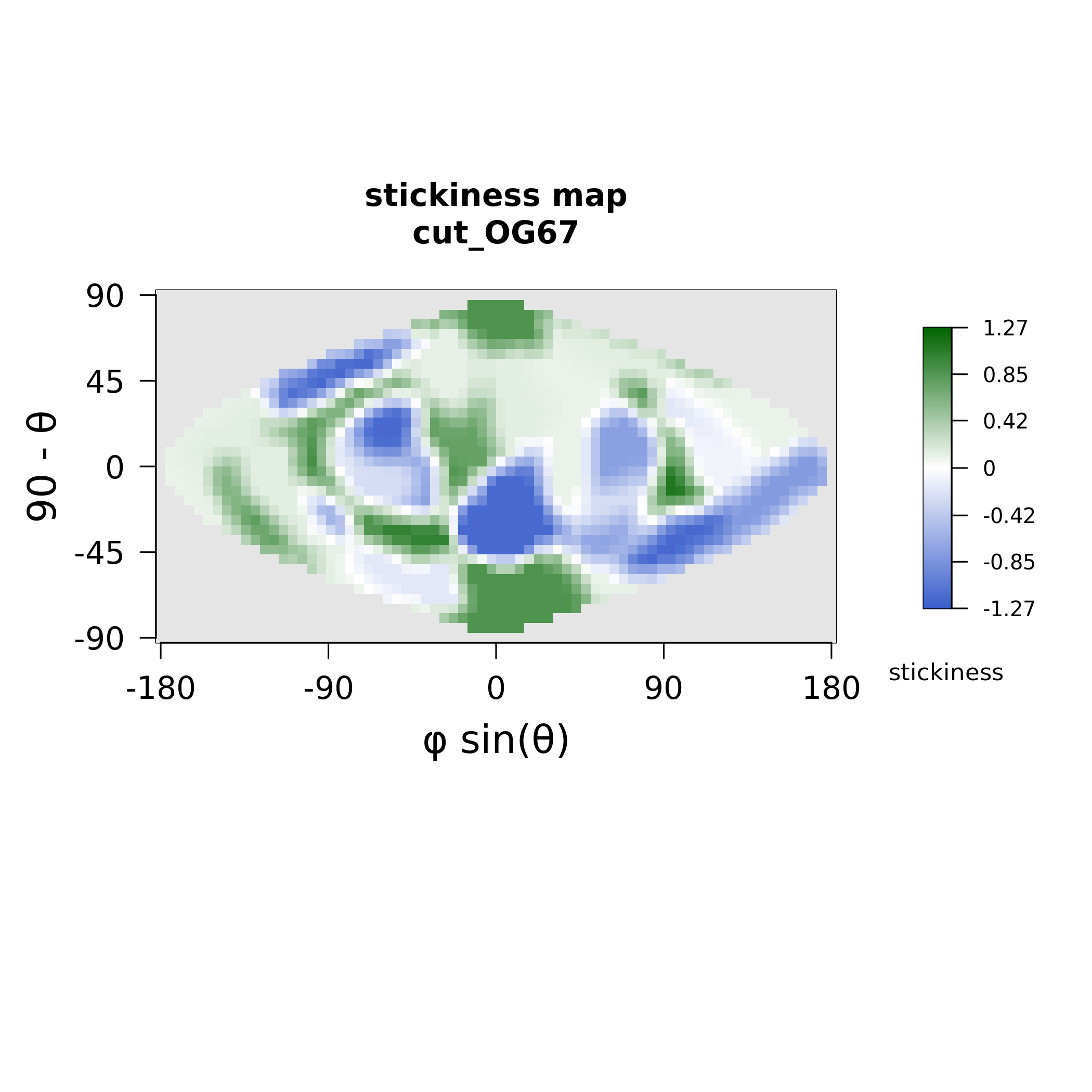

Supplement: S2 File — (ZIP) [file ppat.1012176.s019.zip › S2_File/STICKINESS/MAX67_stickiness.png]

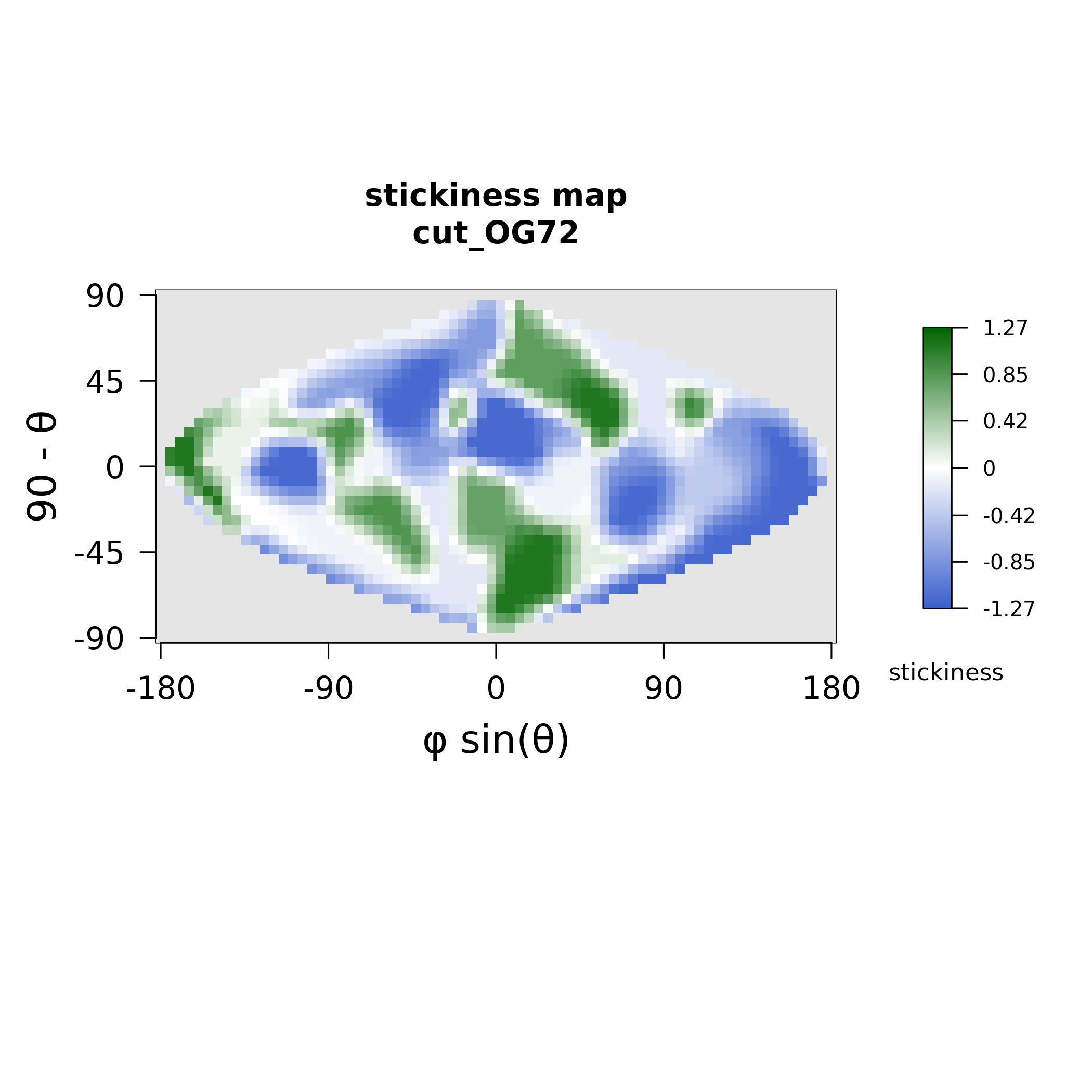

Supplement: S2 File — (ZIP) [file ppat.1012176.s019.zip › S2_File/STICKINESS/MAX72_stickiness.png]

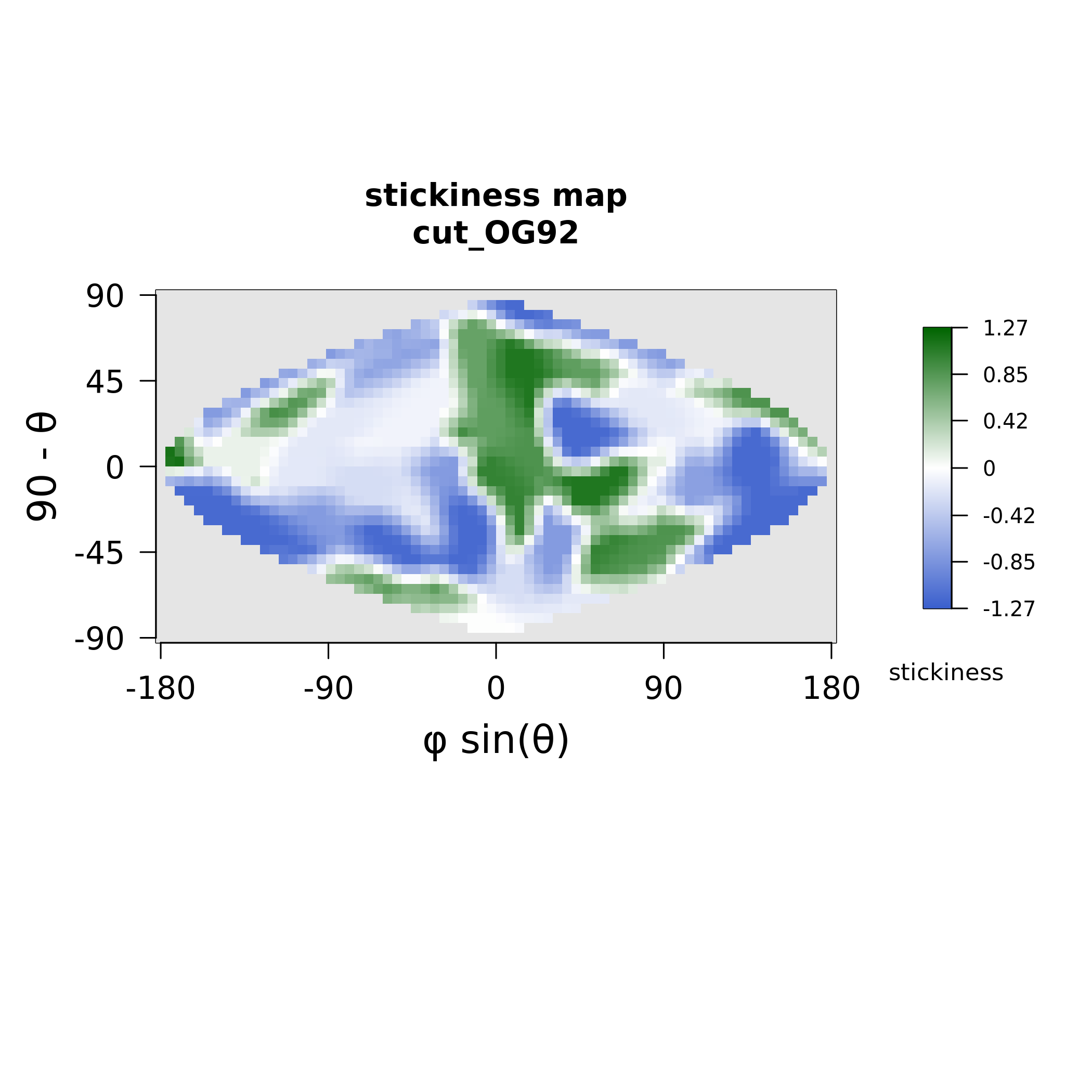

Supplement: S2 File — (ZIP) [file ppat.1012176.s019.zip › S2_File/STICKINESS/MAX92_stickiness.png]

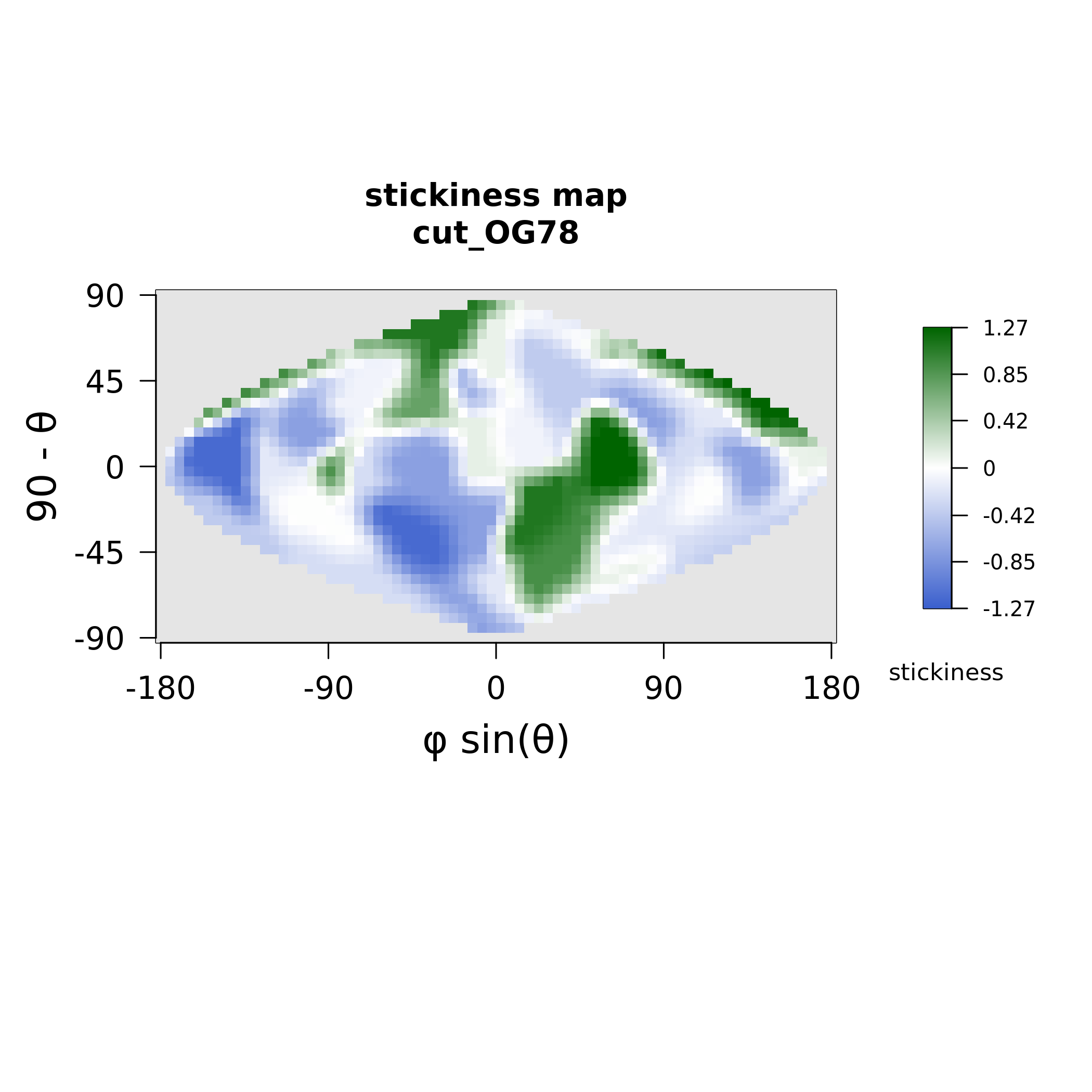

Supplement: S2 File — (ZIP) [file ppat.1012176.s019.zip › S2_File/STICKINESS/MAX78_stickiness.png]

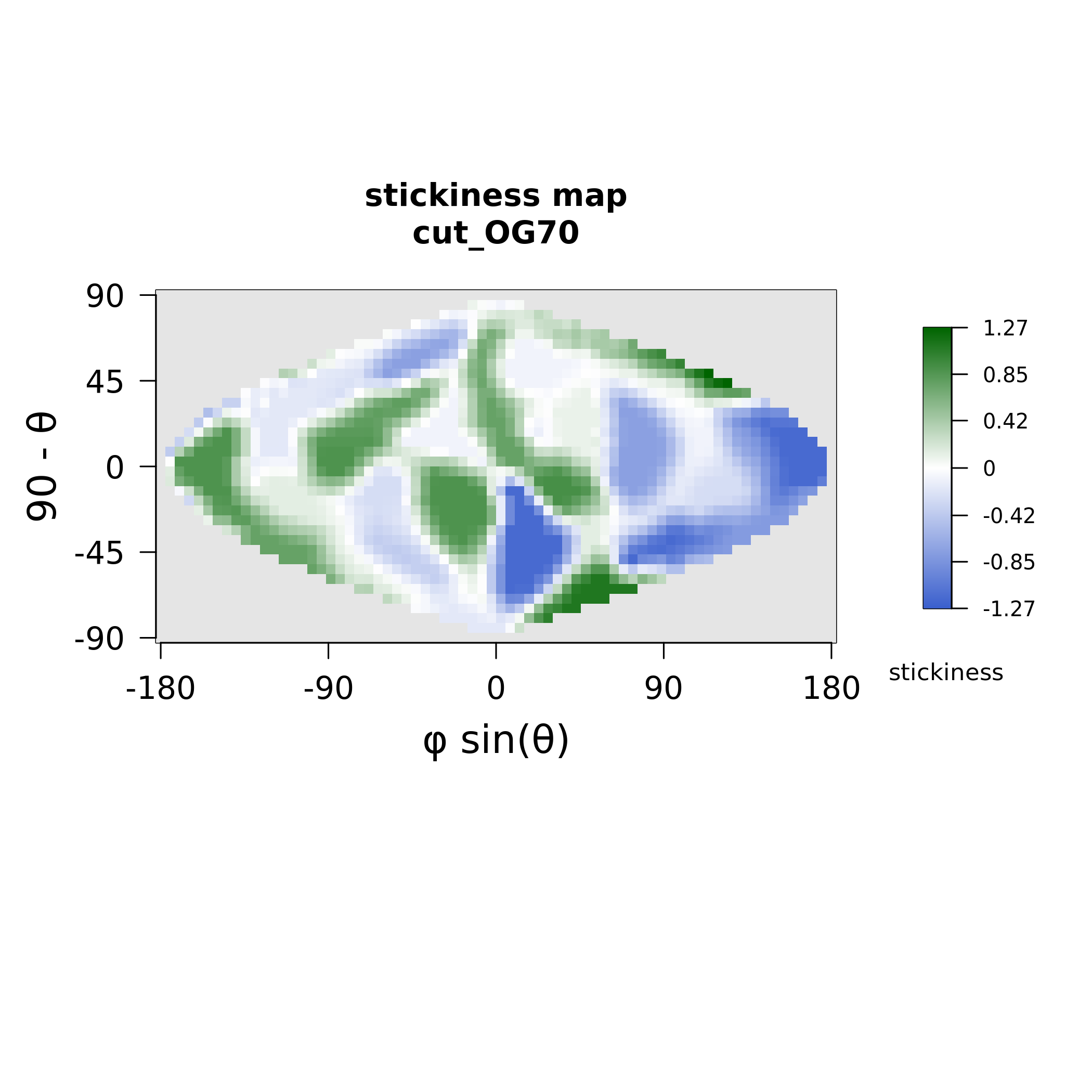

Supplement: S2 File — (ZIP) [file ppat.1012176.s019.zip › S2_File/STICKINESS/MAX70_stickiness.png]

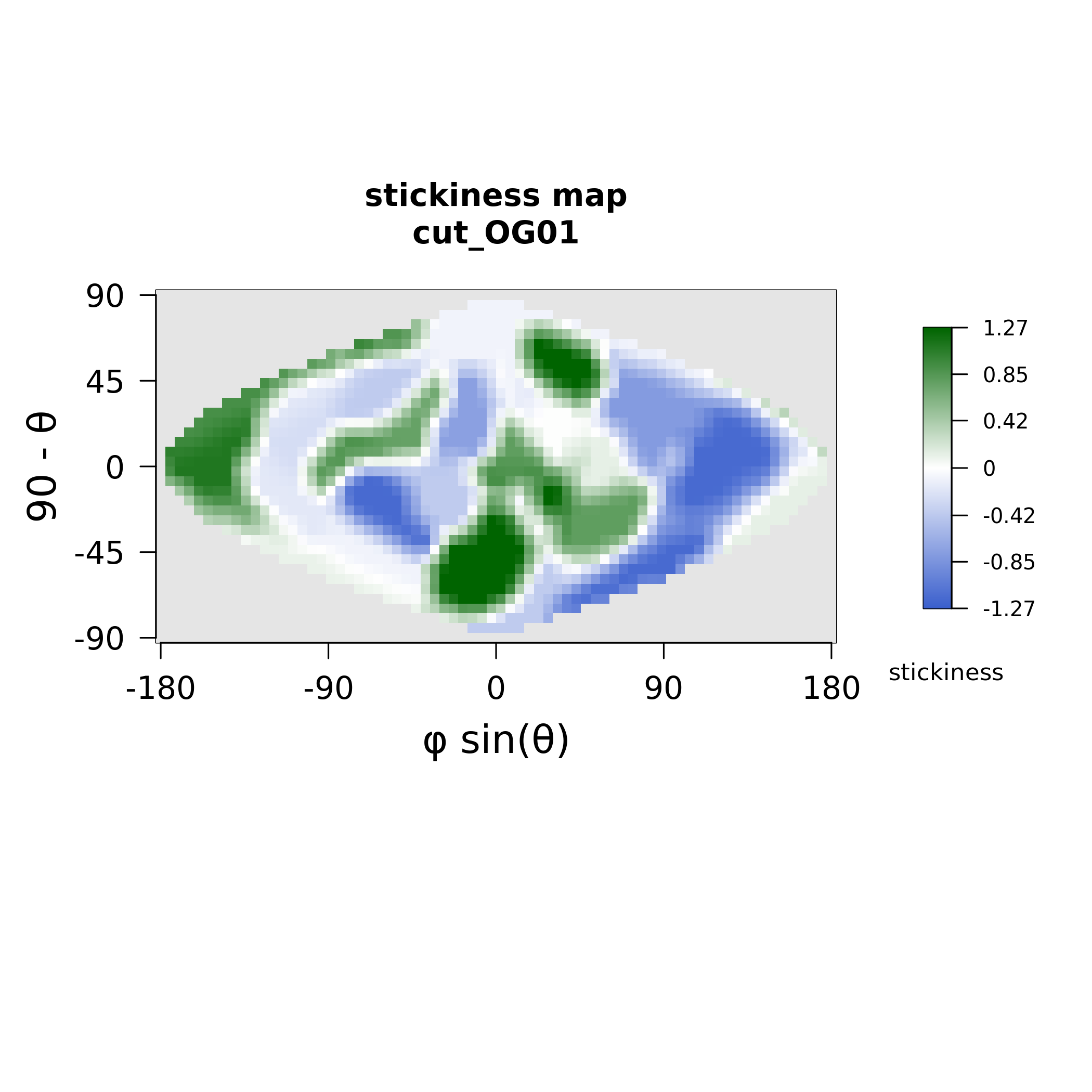

Supplement: S2 File — (ZIP) [file ppat.1012176.s019.zip › S2_File/STICKINESS/MAX01_stickiness.png]

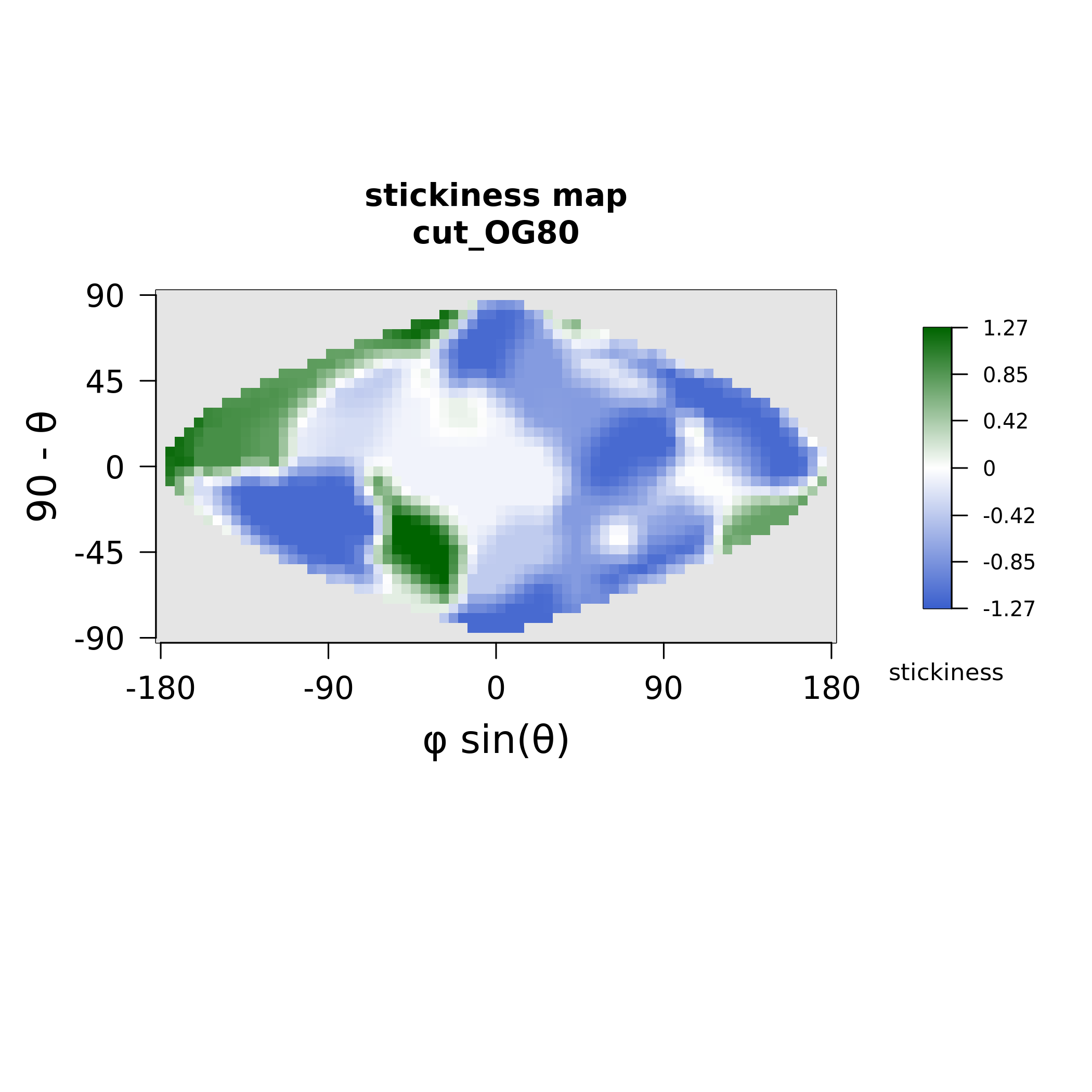

Supplement: S2 File — (ZIP) [file ppat.1012176.s019.zip › S2_File/STICKINESS/MAX80_stickiness.png]

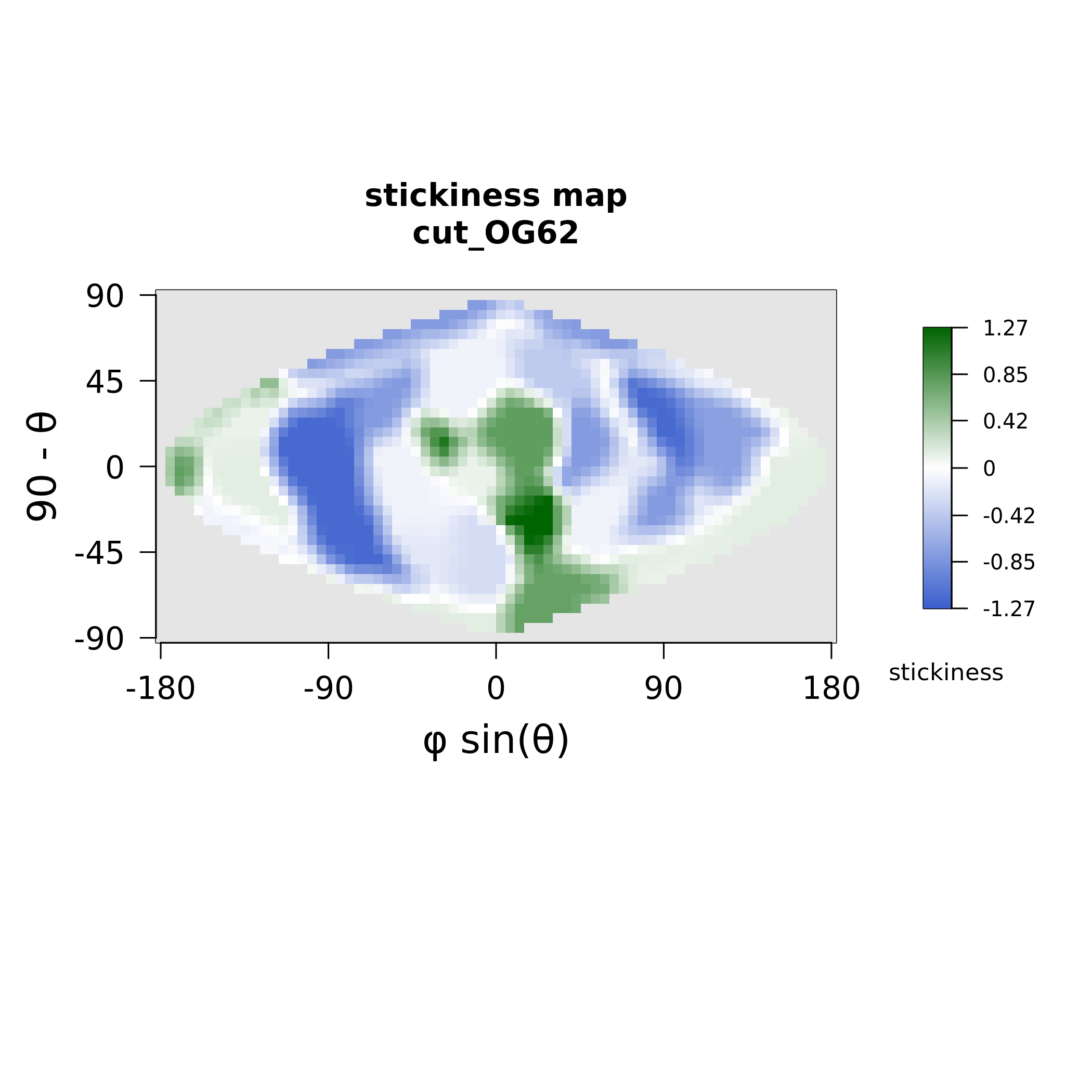

Supplement: S2 File — (ZIP) [file ppat.1012176.s019.zip › S2_File/STICKINESS/MAX62_stickiness.png]

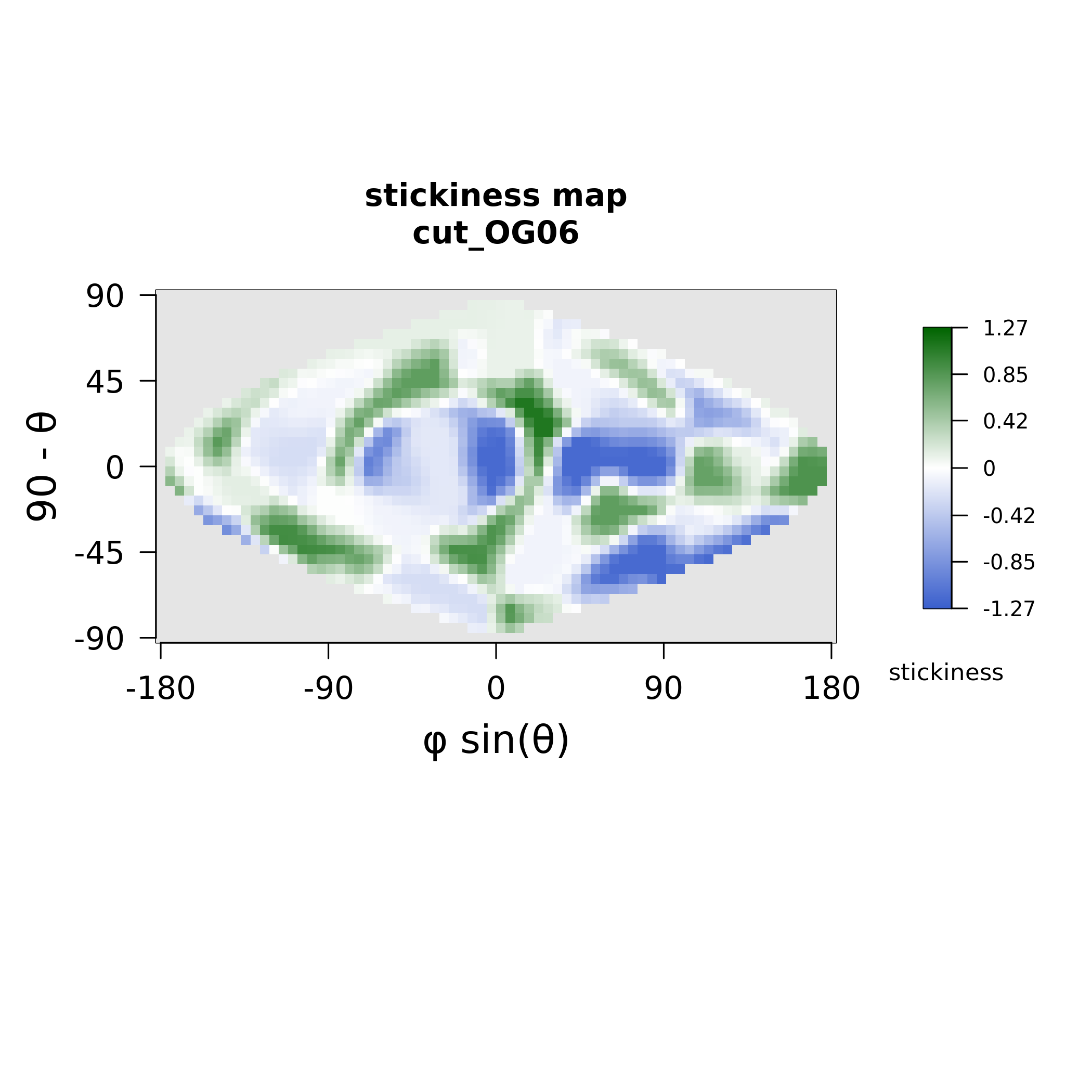

Supplement: S2 File — (ZIP) [file ppat.1012176.s019.zip › S2_File/STICKINESS/MAX06_stickiness.png]

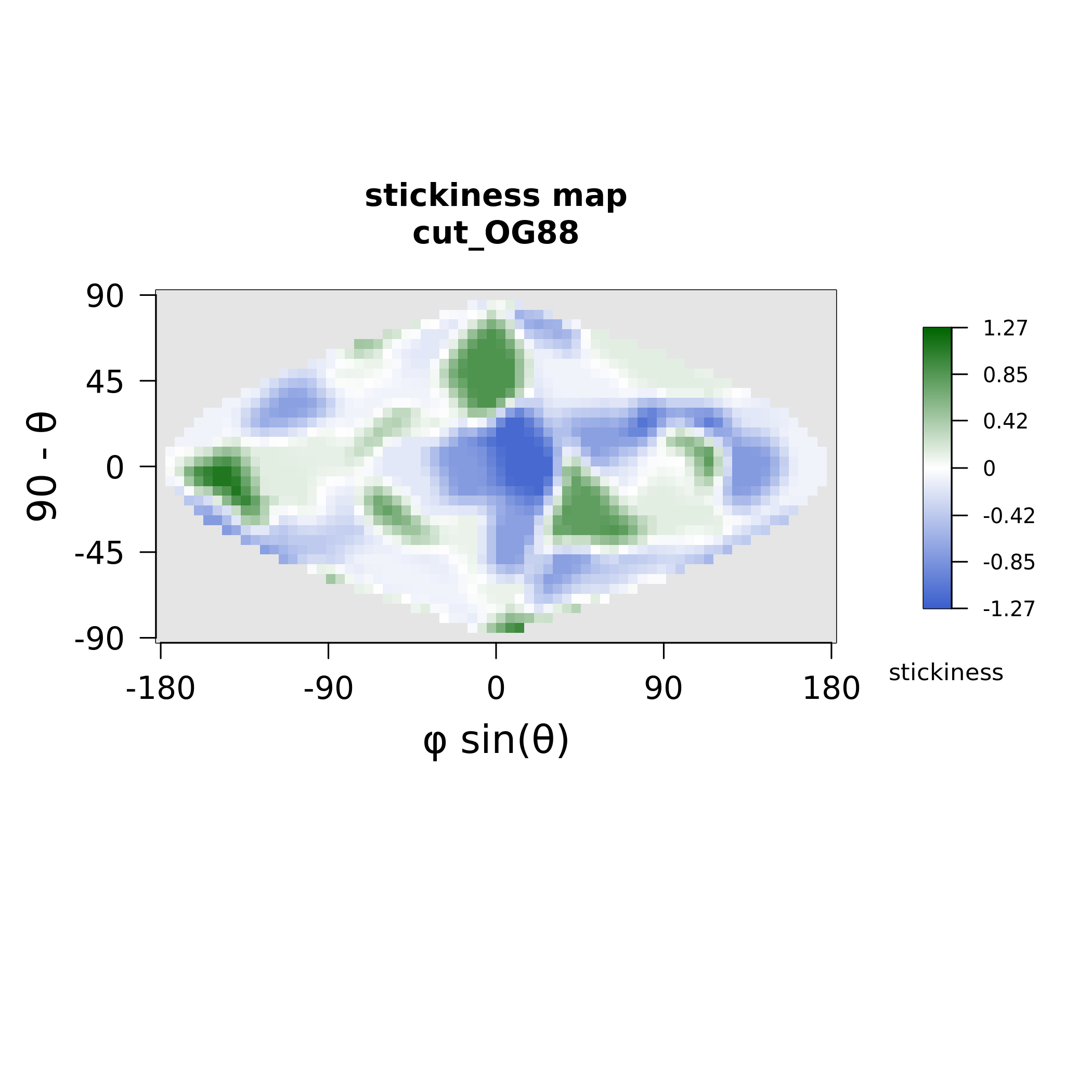

Supplement: S2 File — (ZIP) [file ppat.1012176.s019.zip › S2_File/STICKINESS/MAX88_stickiness.png]

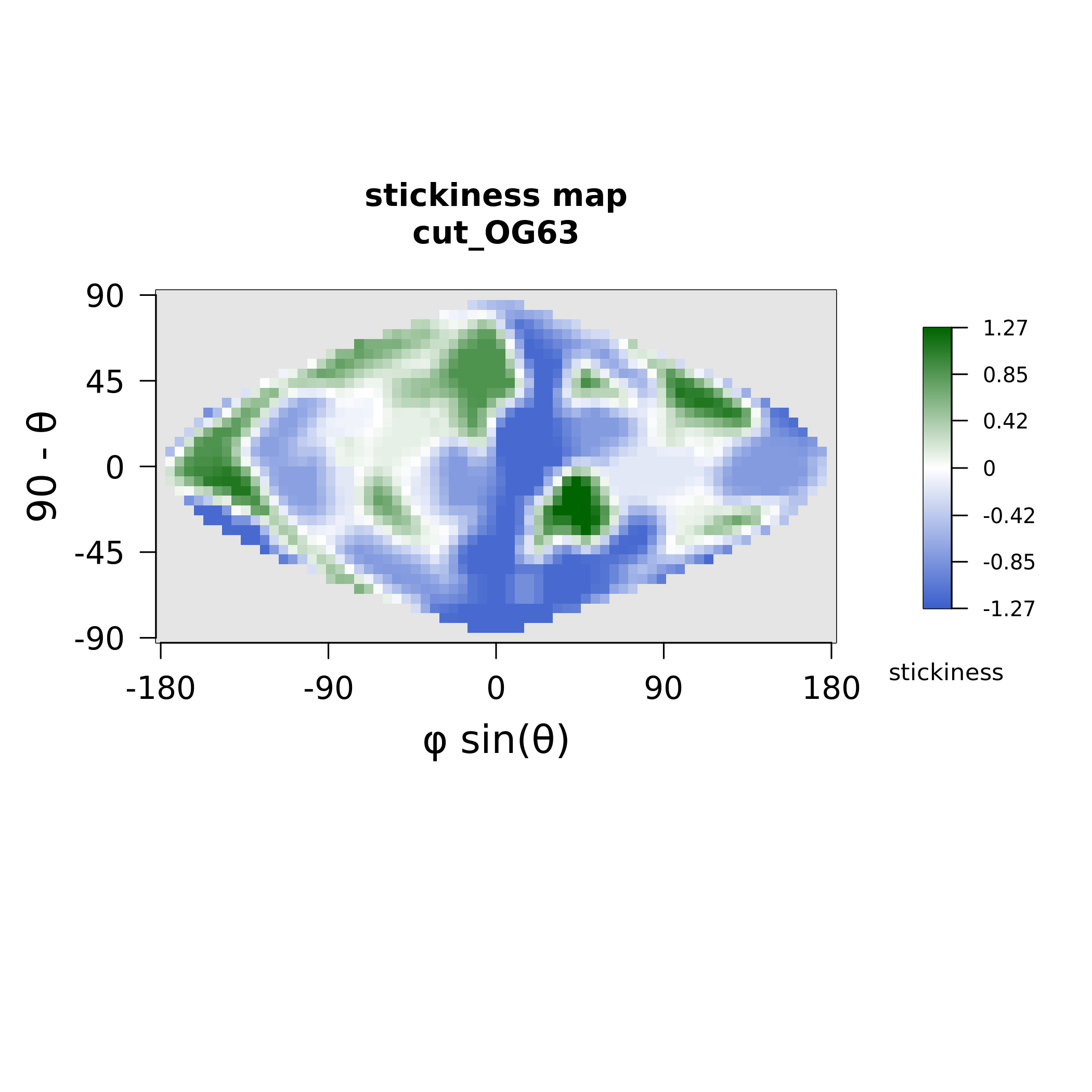

Supplement: S2 File — (ZIP) [file ppat.1012176.s019.zip › S2_File/STICKINESS/MAX63_stickiness.png]

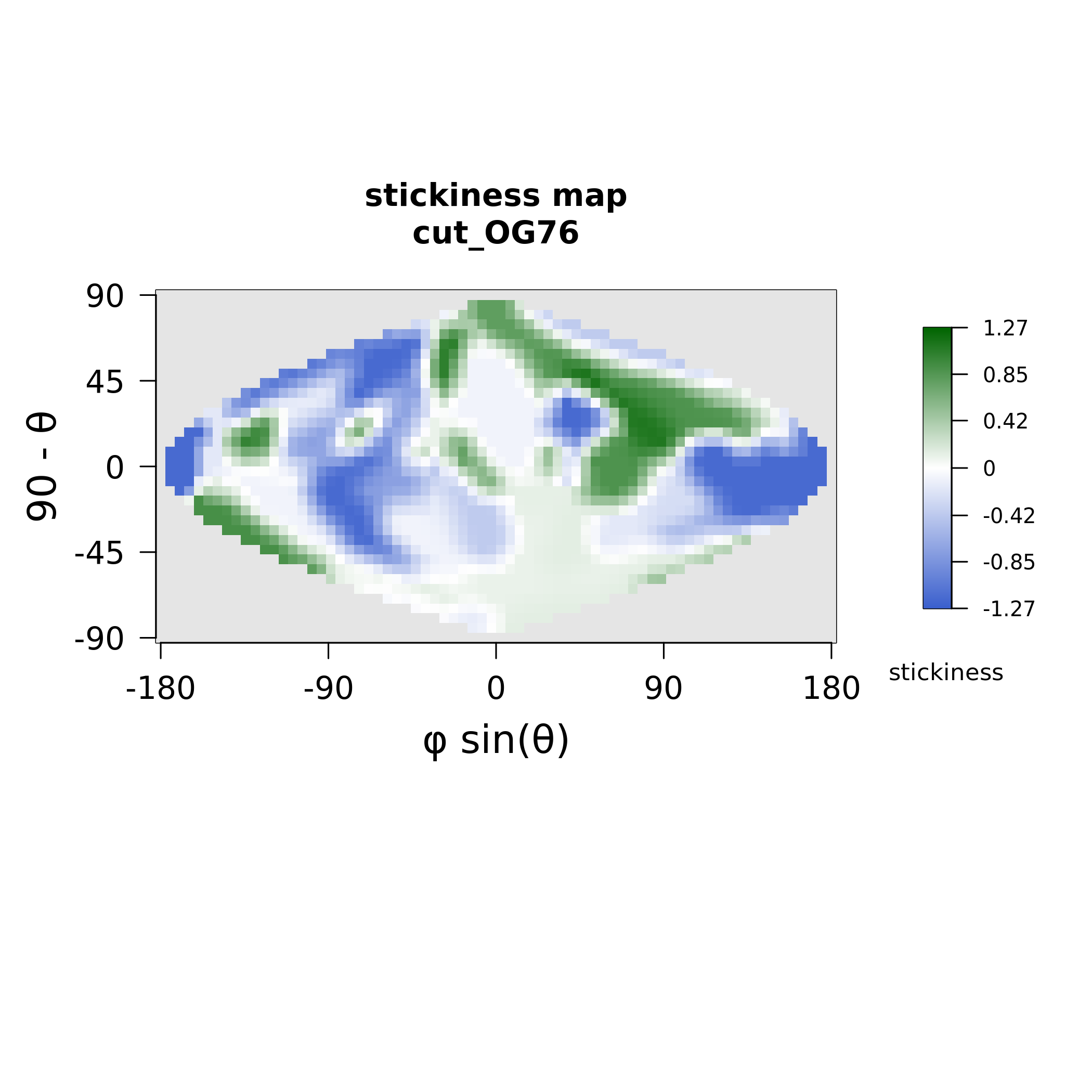

Supplement: S2 File — (ZIP) [file ppat.1012176.s019.zip › S2_File/STICKINESS/MAX76_stickiness.png]

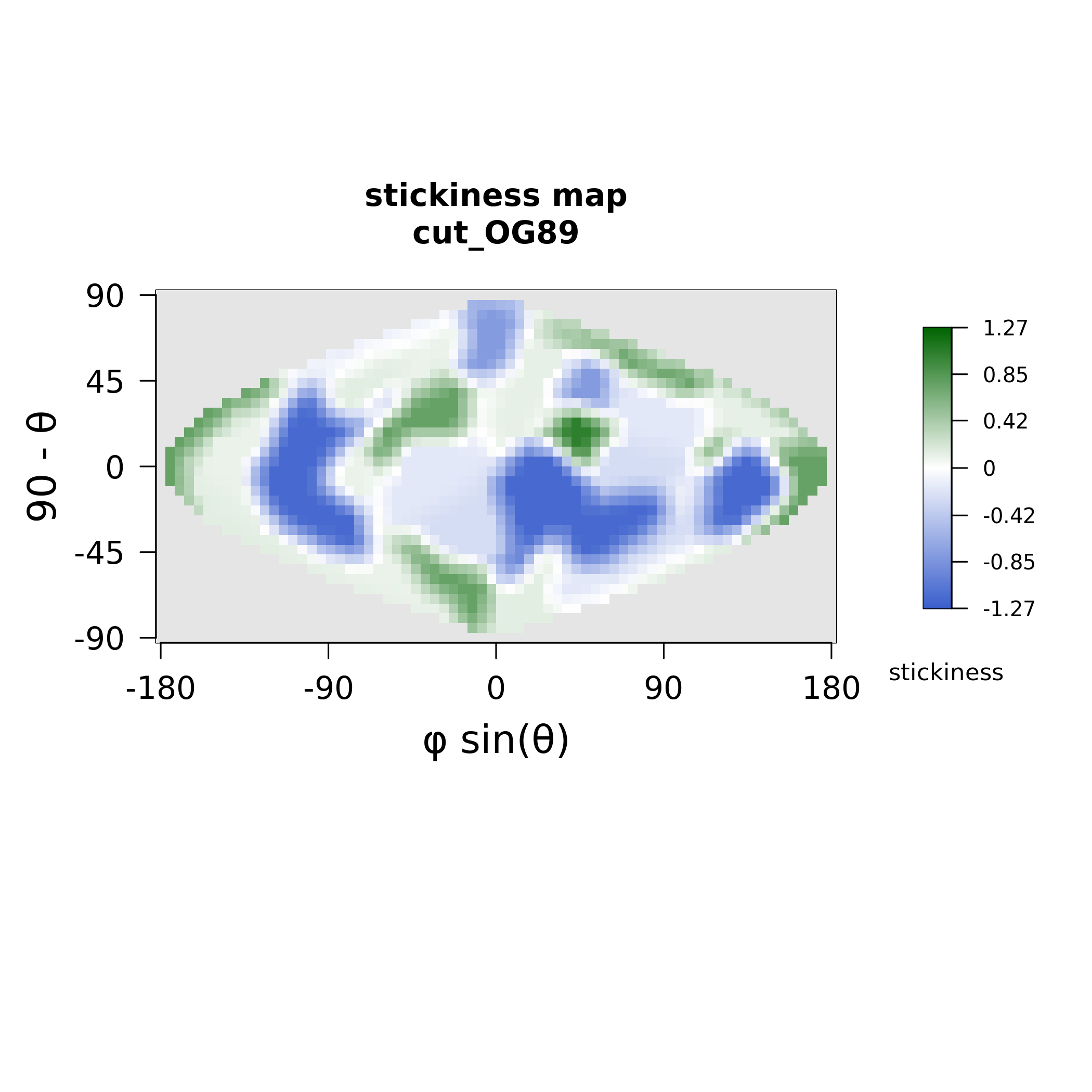

Supplement: S2 File — (ZIP) [file ppat.1012176.s019.zip › S2_File/STICKINESS/MAX89_stickiness.png]

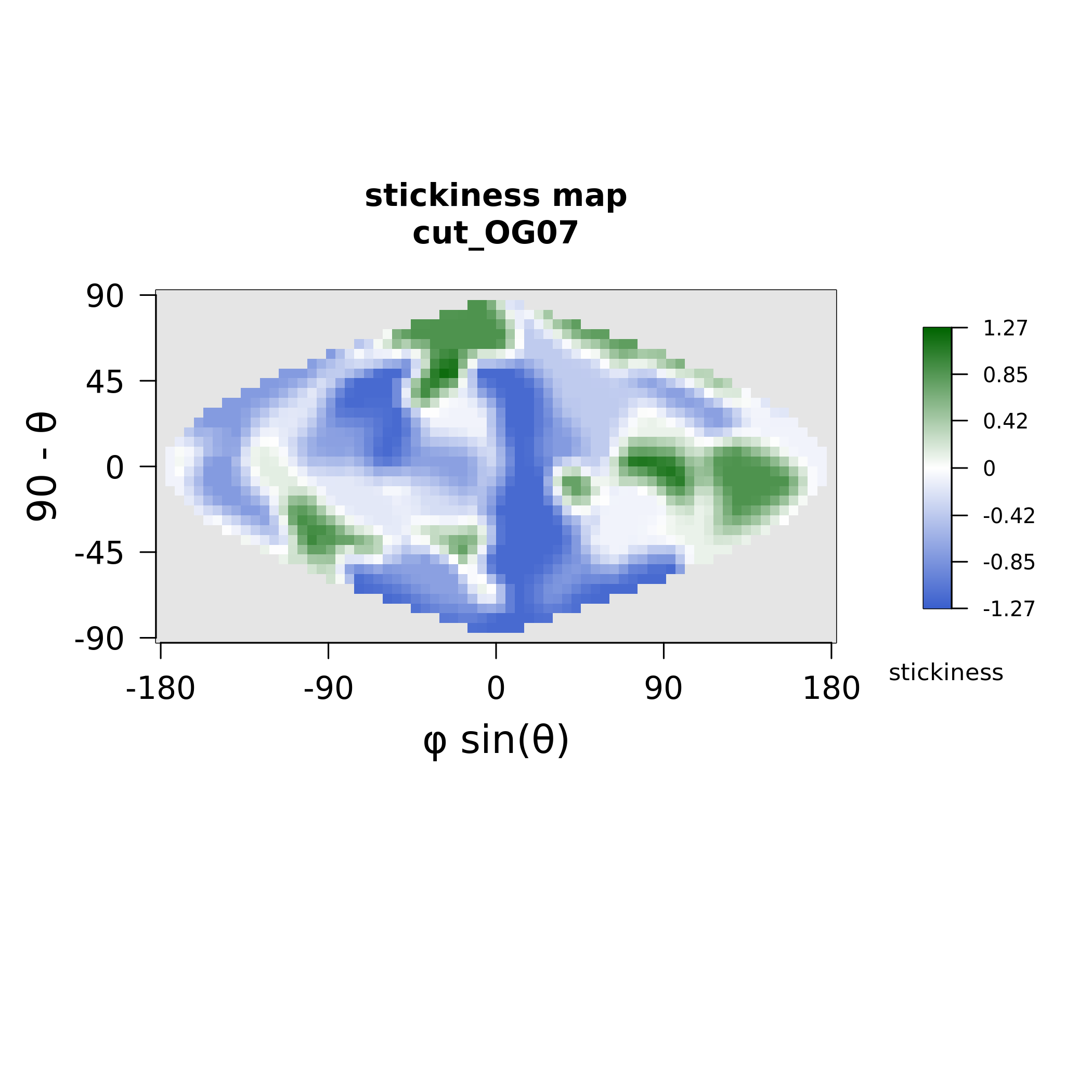

Supplement: S2 File — (ZIP) [file ppat.1012176.s019.zip › S2_File/STICKINESS/MAX07_stickiness.png]

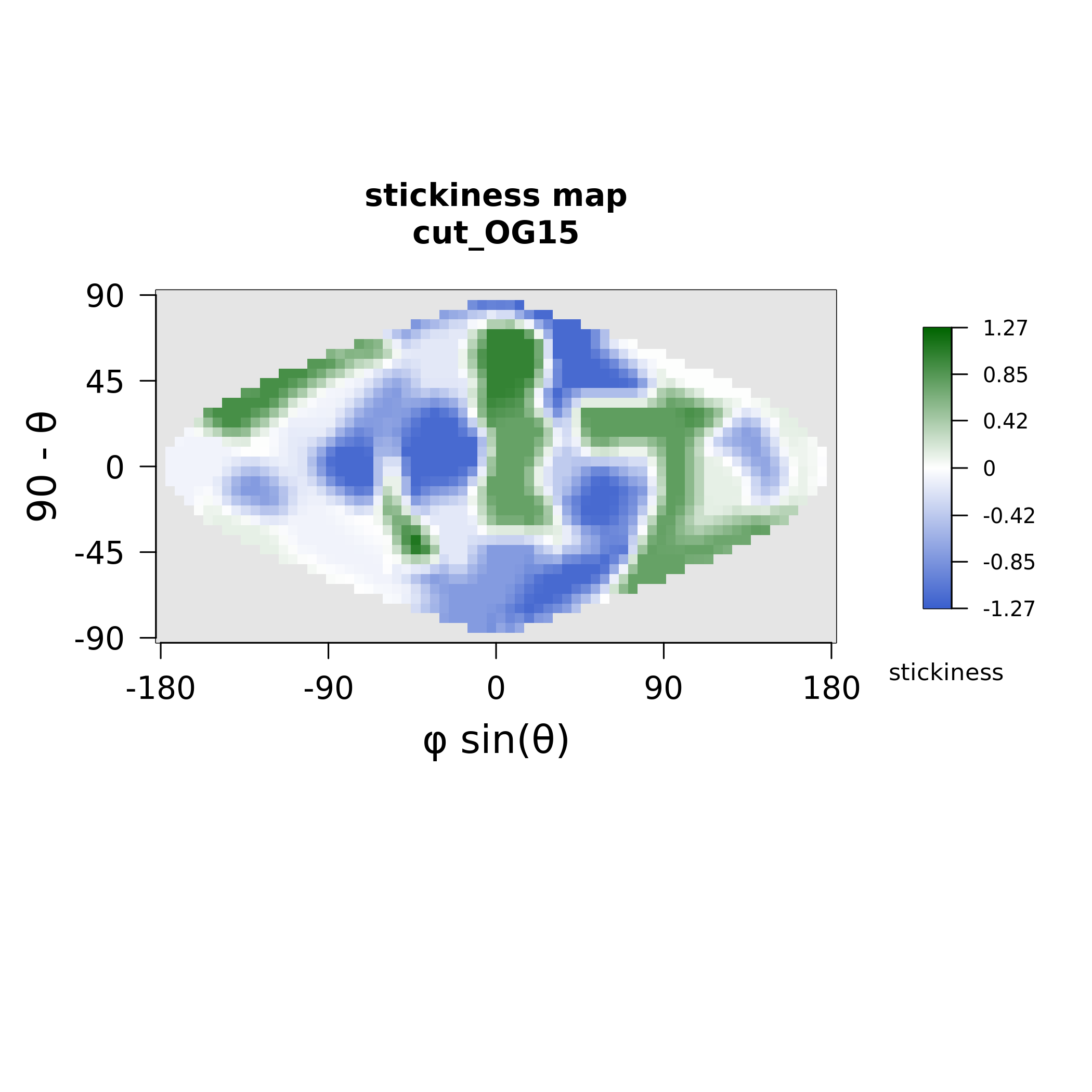

Supplement: S2 File — (ZIP) [file ppat.1012176.s019.zip › S2_File/STICKINESS/MAX15_stickiness.png]

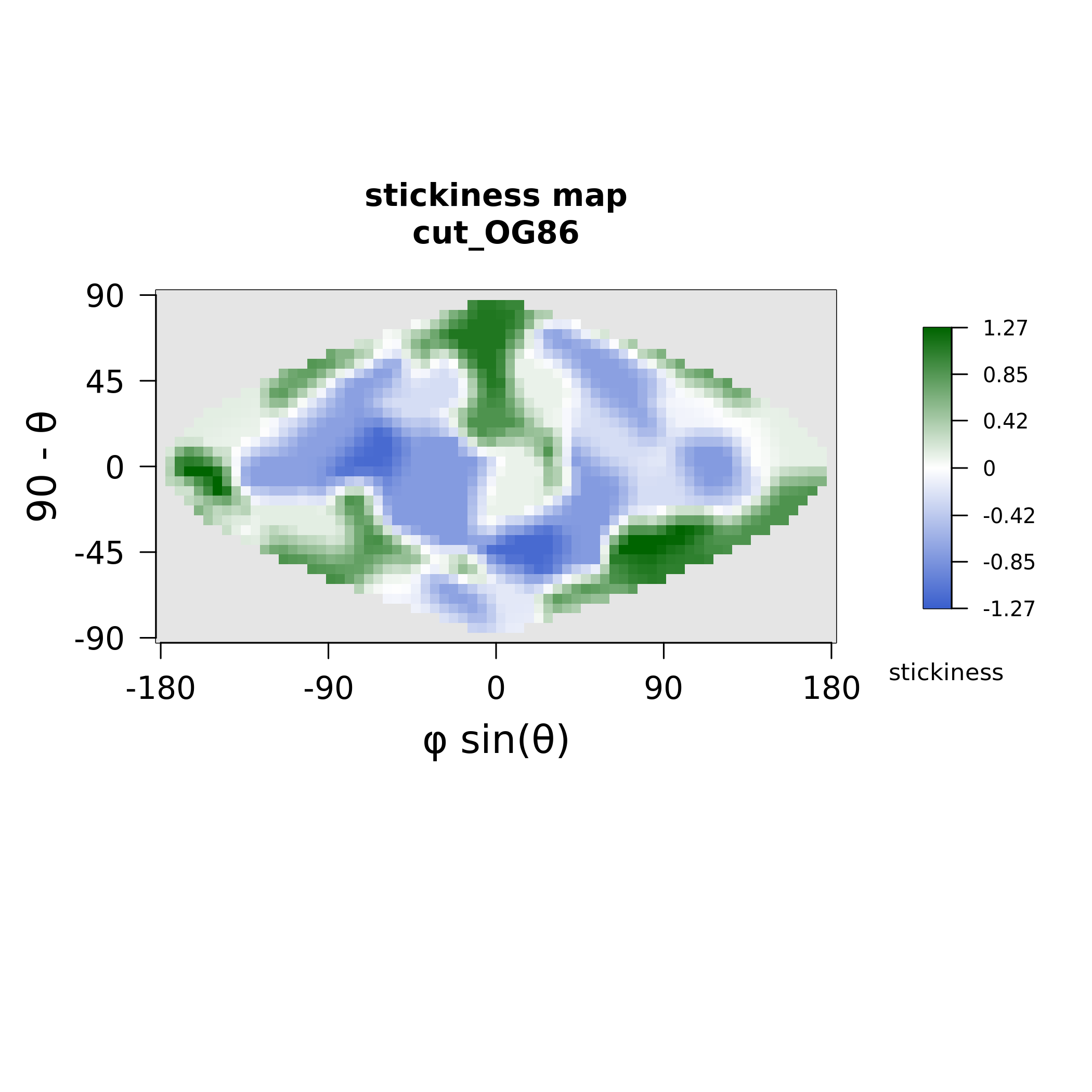

Supplement: S2 File — (ZIP) [file ppat.1012176.s019.zip › S2_File/STICKINESS/MAX86_stickiness.png]

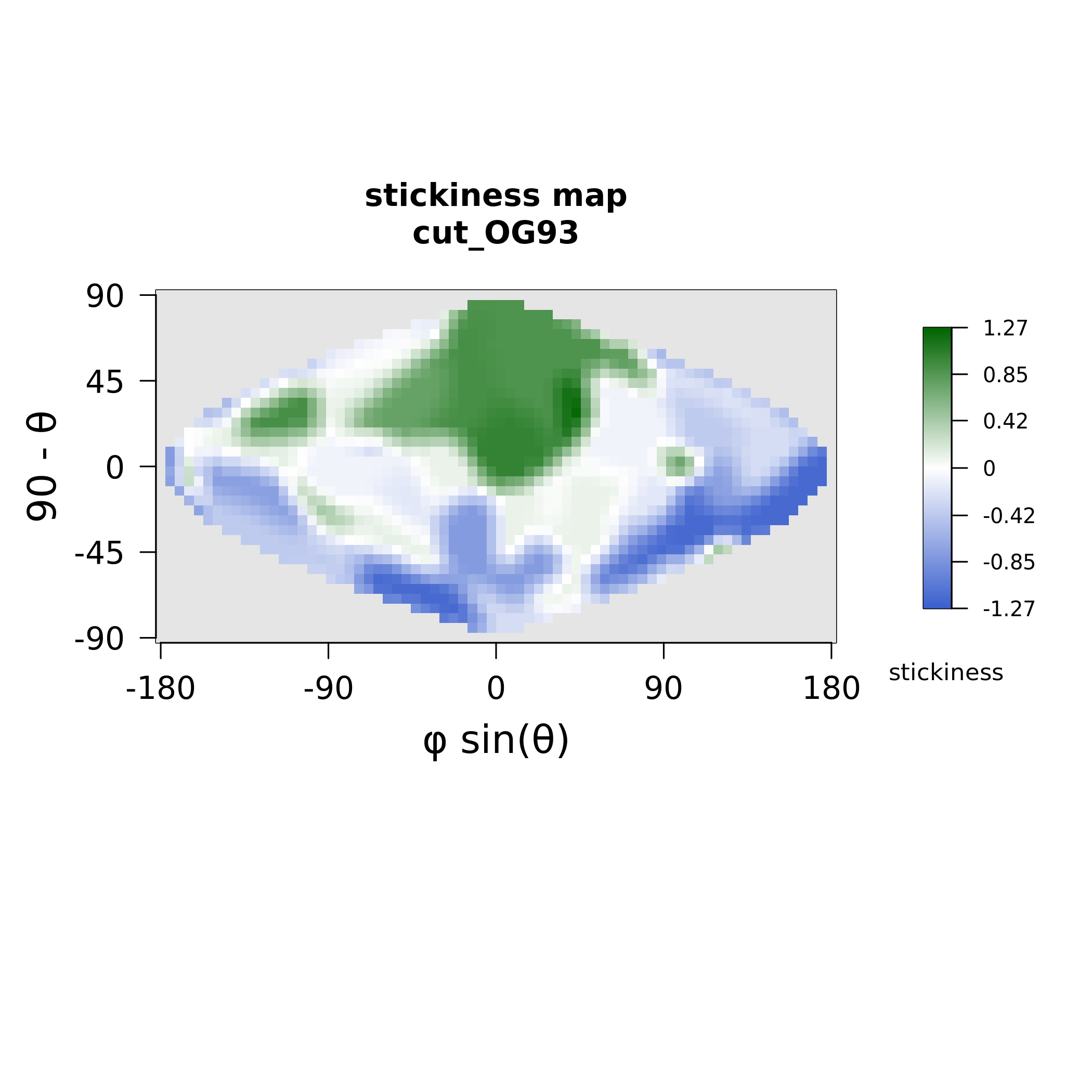

Supplement: S2 File — (ZIP) [file ppat.1012176.s019.zip › S2_File/STICKINESS/MAX93_stickiness.png]

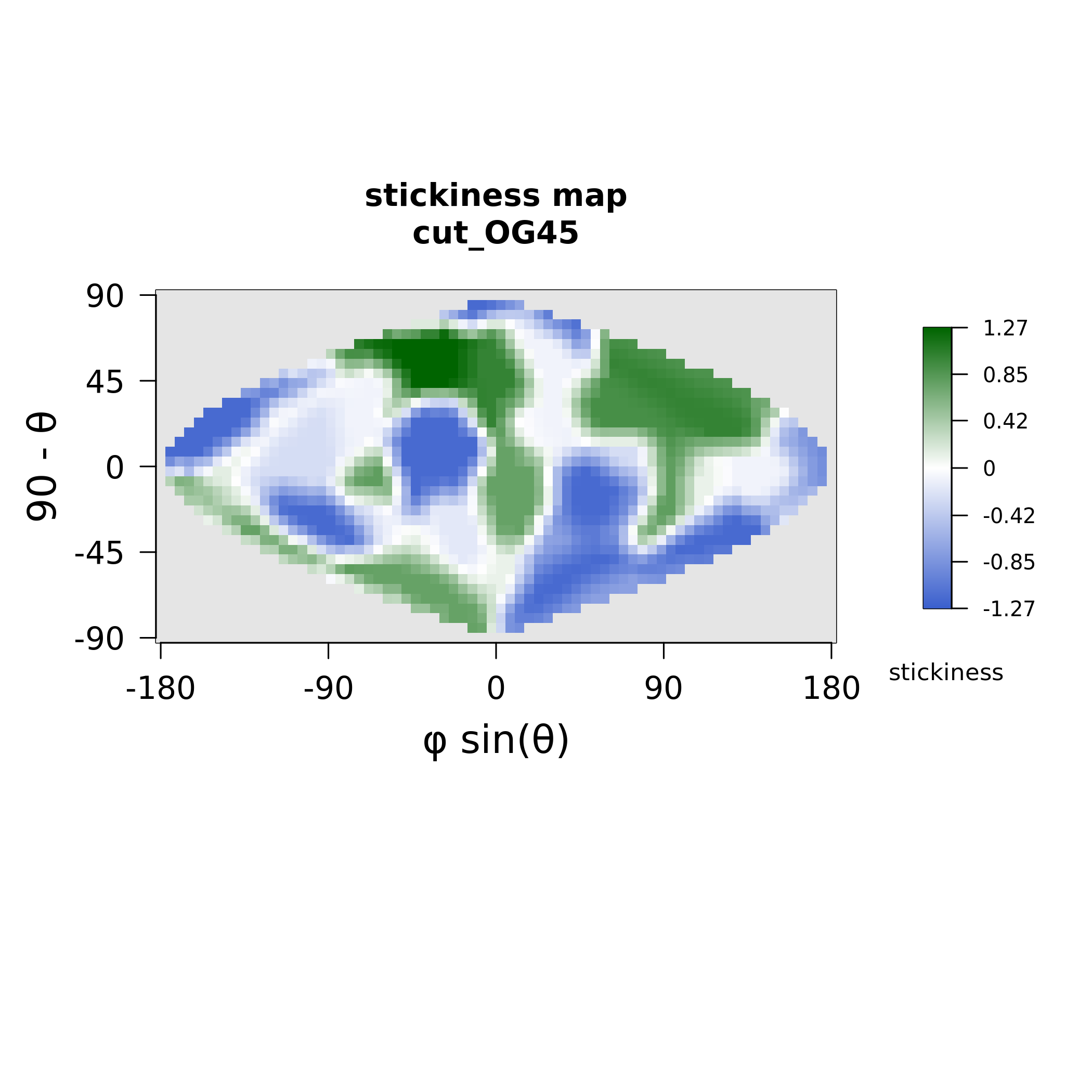

Supplement: S2 File — (ZIP) [file ppat.1012176.s019.zip › S2_File/STICKINESS/MAX45_stickiness.png]

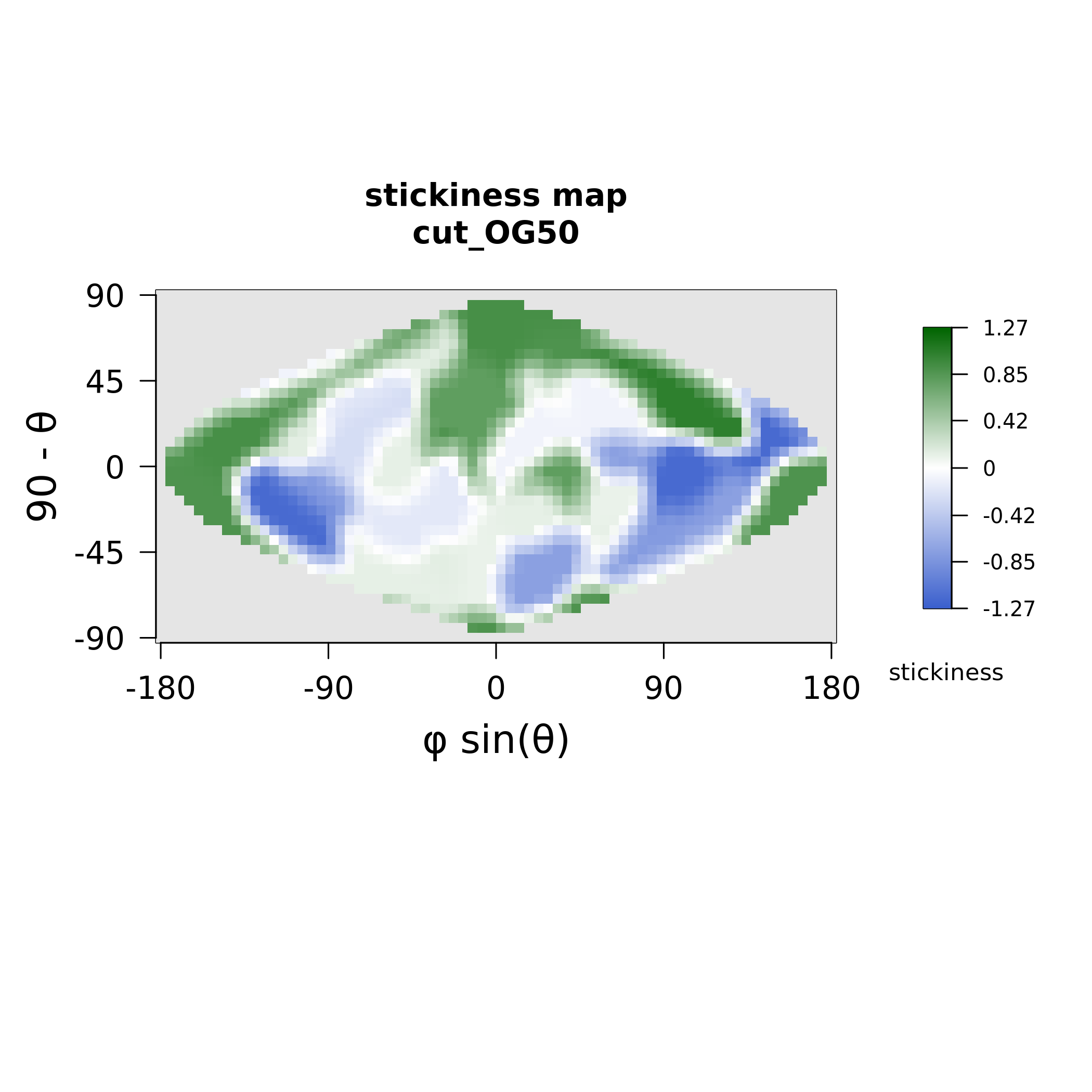

Supplement: S2 File — (ZIP) [file ppat.1012176.s019.zip › S2_File/STICKINESS/MAX50_stickiness.png]

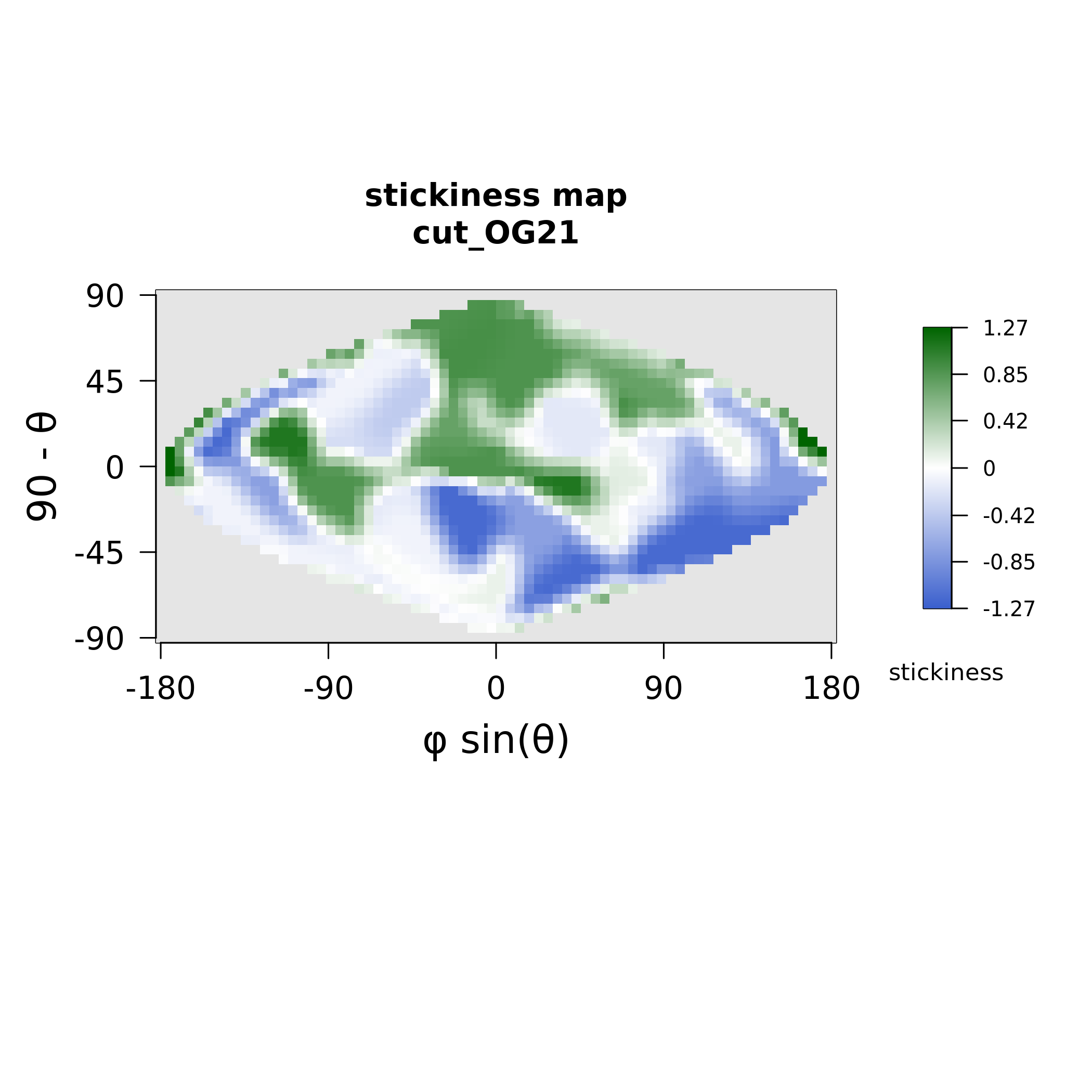

Supplement: S2 File — (ZIP) [file ppat.1012176.s019.zip › S2_File/STICKINESS/MAX21_stickiness.png]

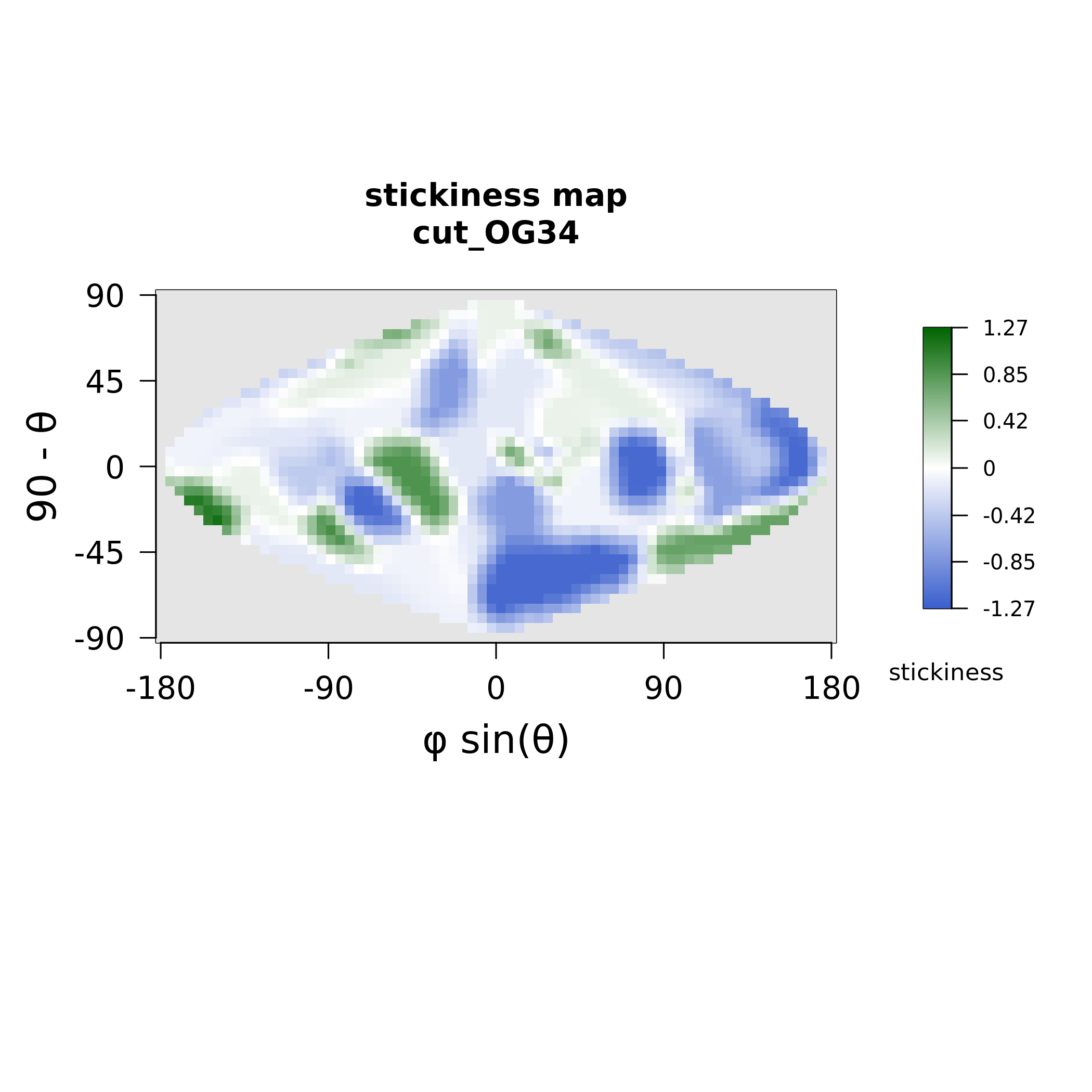

Supplement: S2 File — (ZIP) [file ppat.1012176.s019.zip › S2_File/STICKINESS/MAX34_stickiness.png]

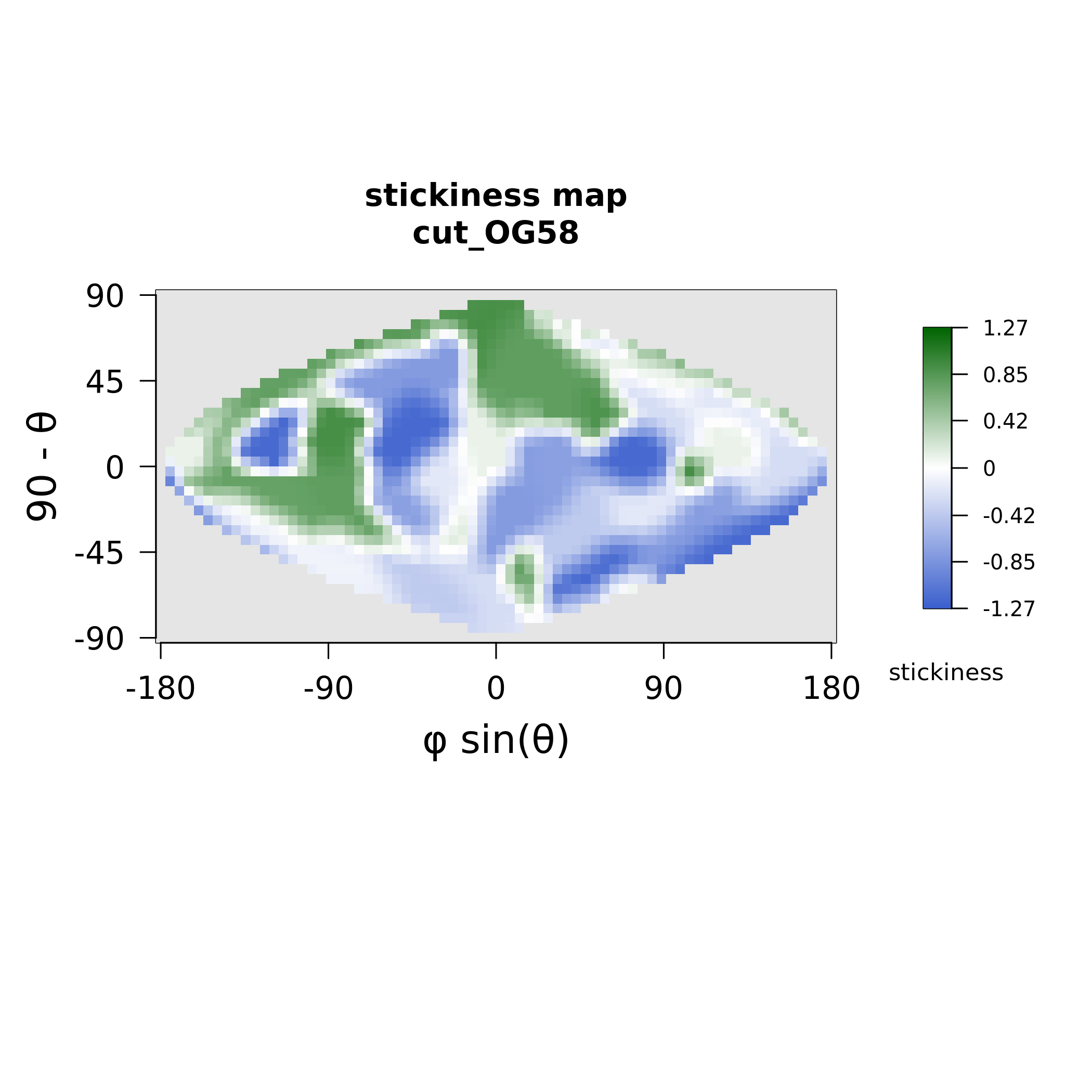

Supplement: S2 File — (ZIP) [file ppat.1012176.s019.zip › S2_File/STICKINESS/MAX58_stickiness.png]

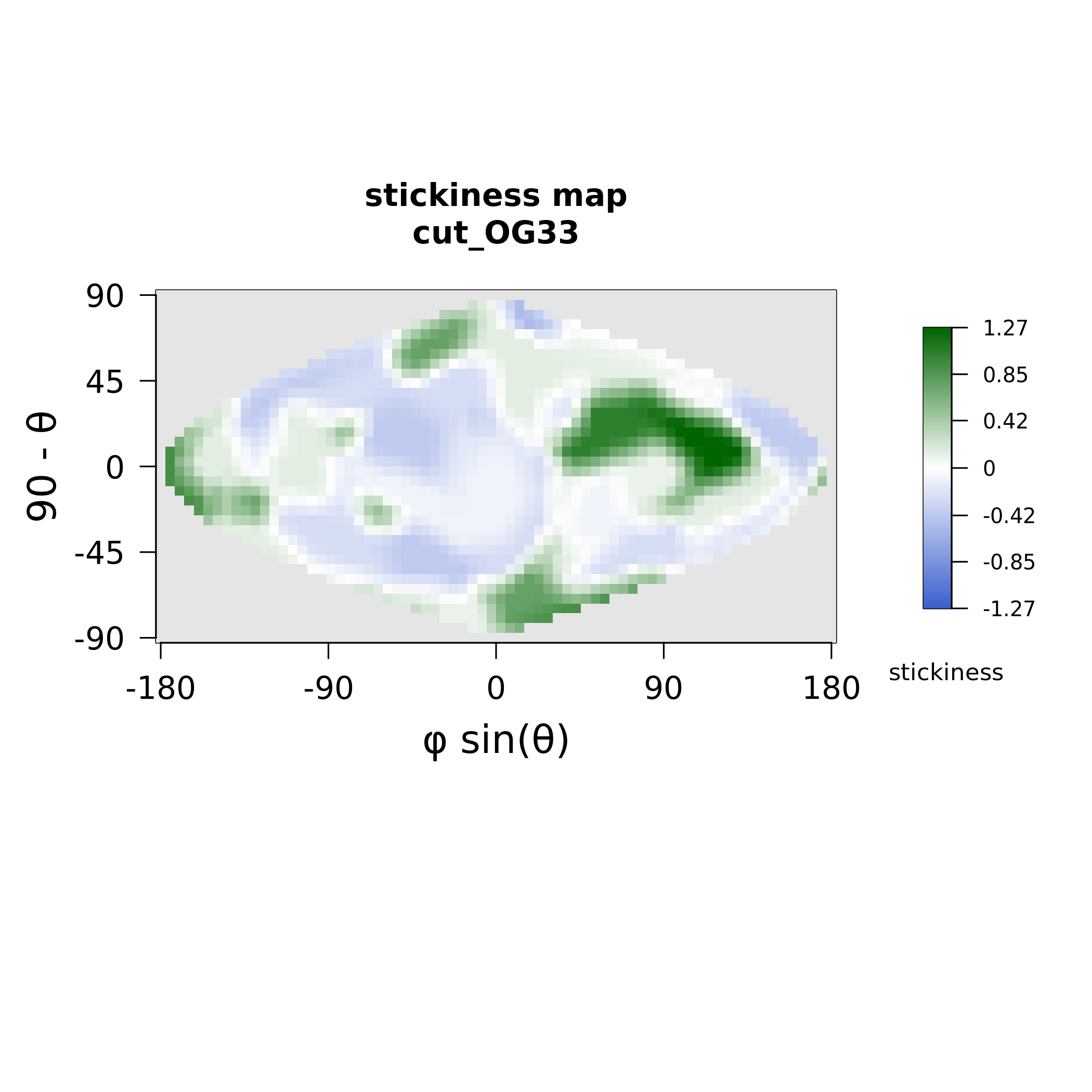

Supplement: S2 File — (ZIP) [file ppat.1012176.s019.zip › S2_File/STICKINESS/MAX33_stickiness.png]

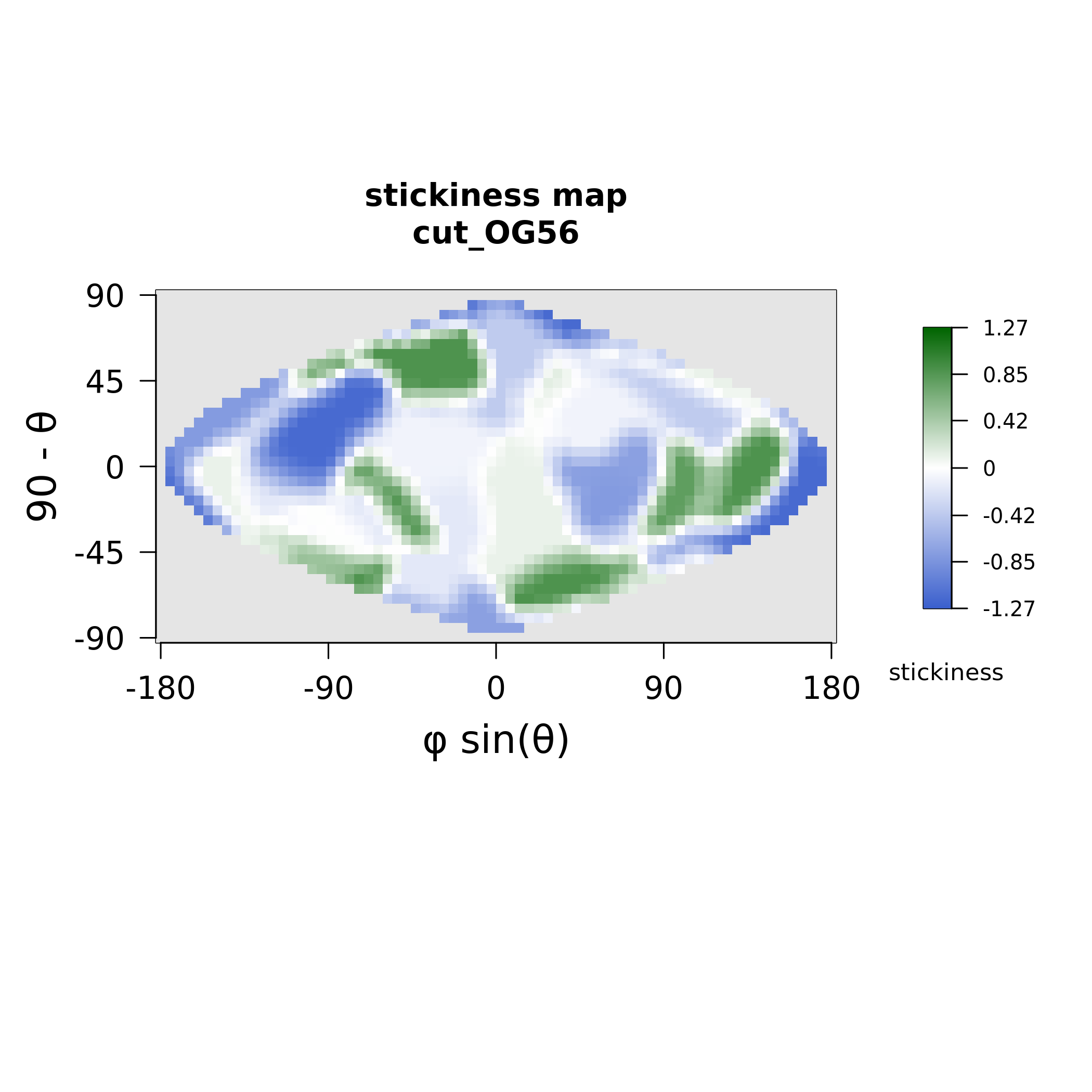

Supplement: S2 File — (ZIP) [file ppat.1012176.s019.zip › S2_File/STICKINESS/MAX56_stickiness.png]

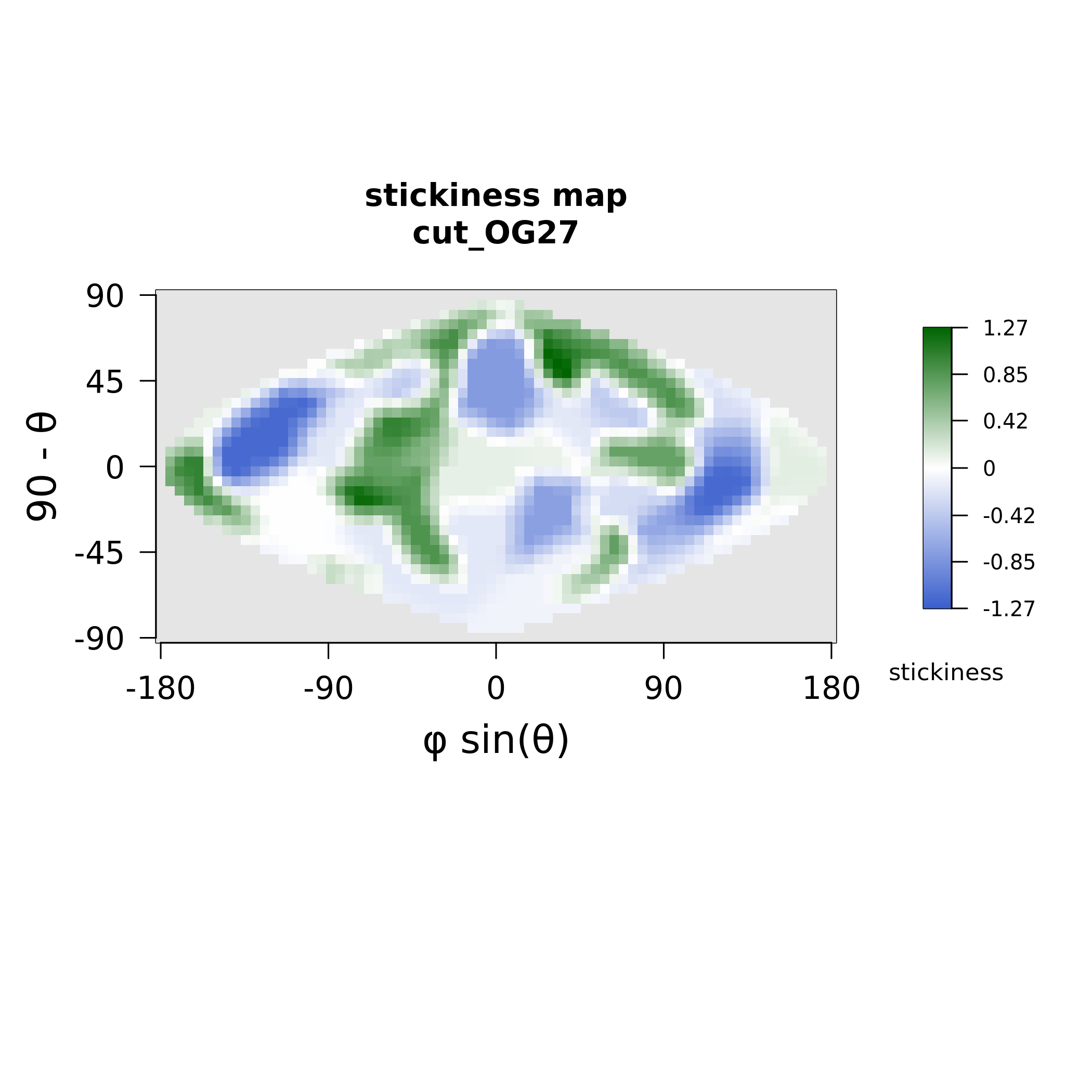

Supplement: S2 File — (ZIP) [file ppat.1012176.s019.zip › S2_File/STICKINESS/MAX27_stickiness.png]

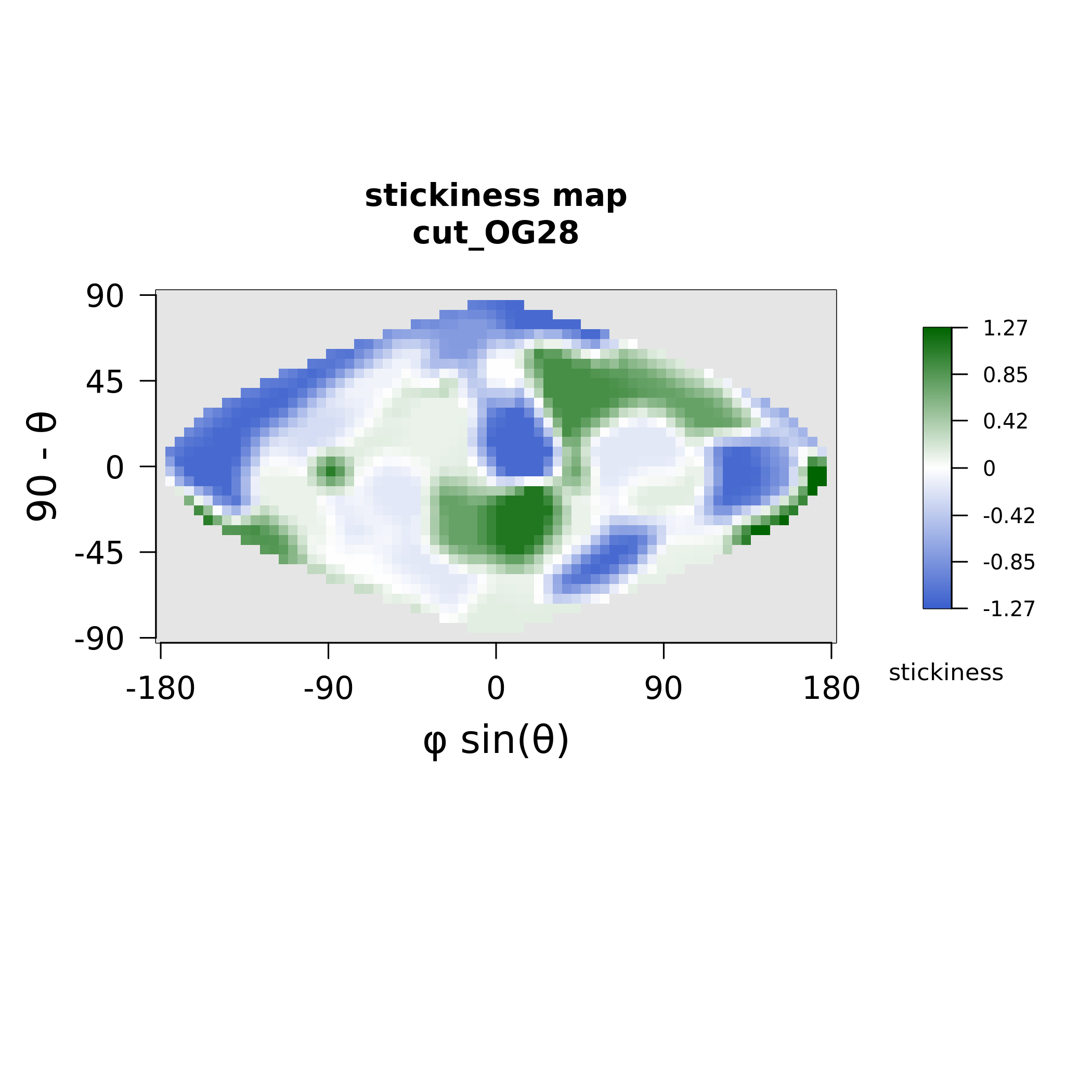

Supplement: S2 File — (ZIP) [file ppat.1012176.s019.zip › S2_File/STICKINESS/MAX28_stickiness.png]

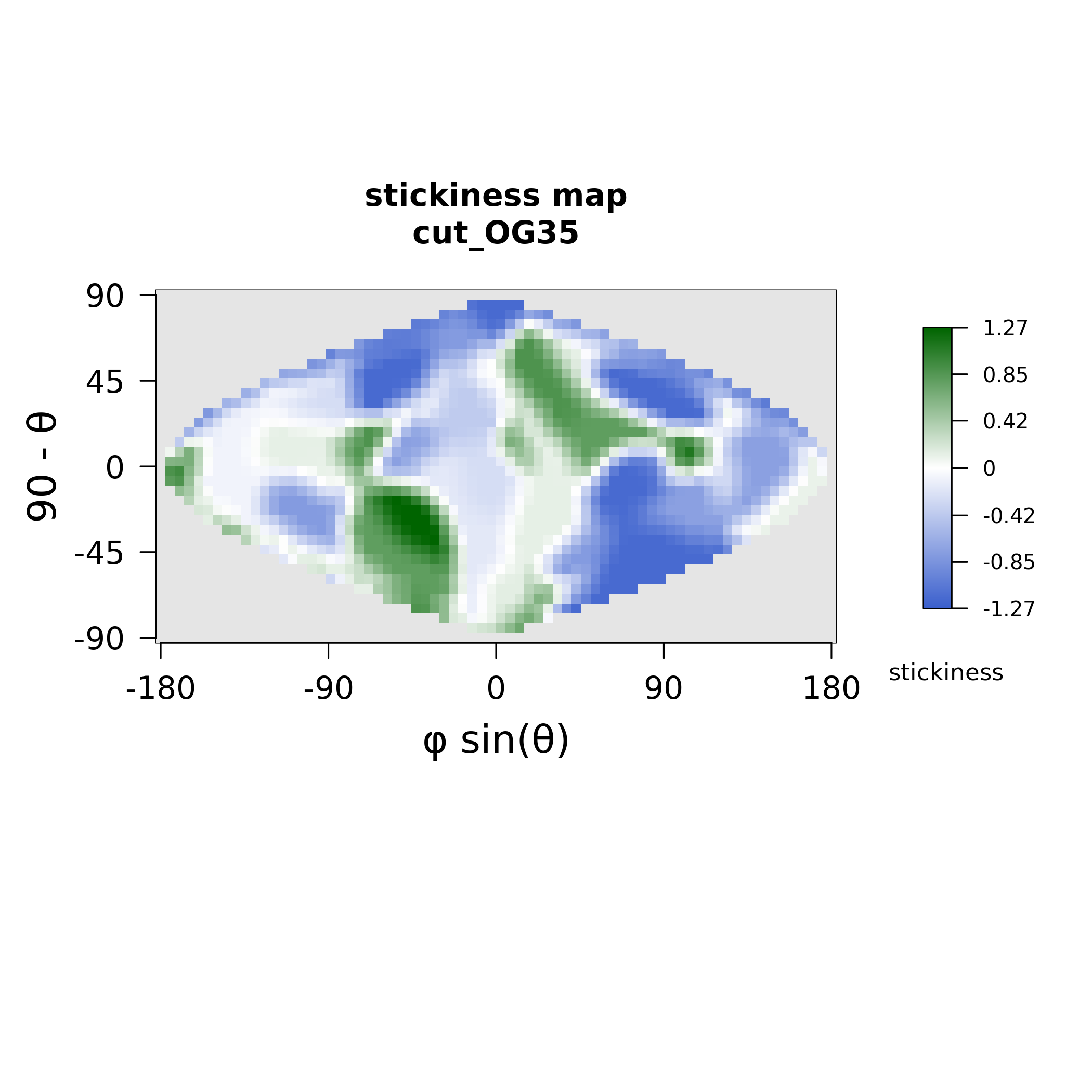

Supplement: S2 File — (ZIP) [file ppat.1012176.s019.zip › S2_File/STICKINESS/MAX35_stickiness.png]

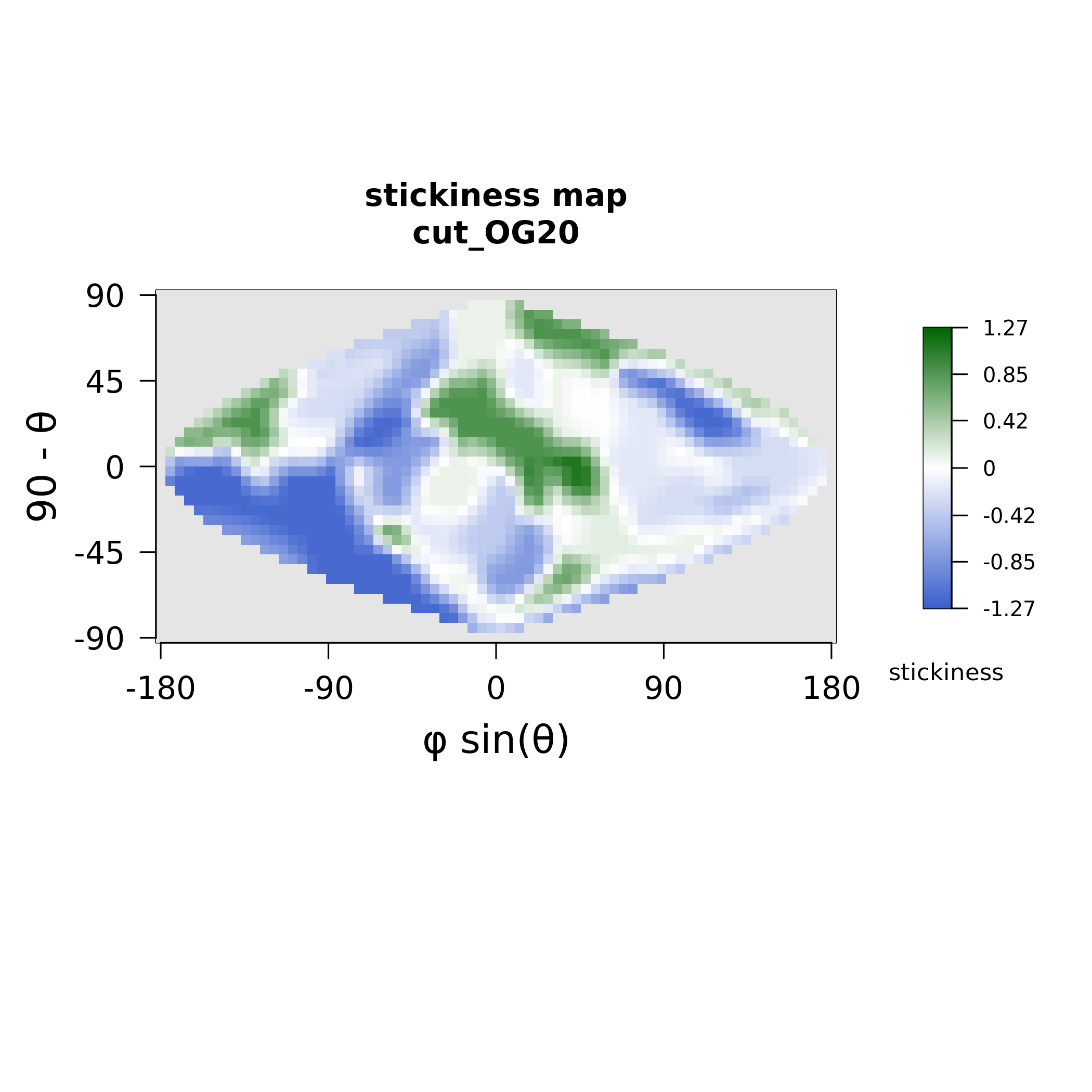

Supplement: S2 File — (ZIP) [file ppat.1012176.s019.zip › S2_File/STICKINESS/MAX20_stickiness.png]

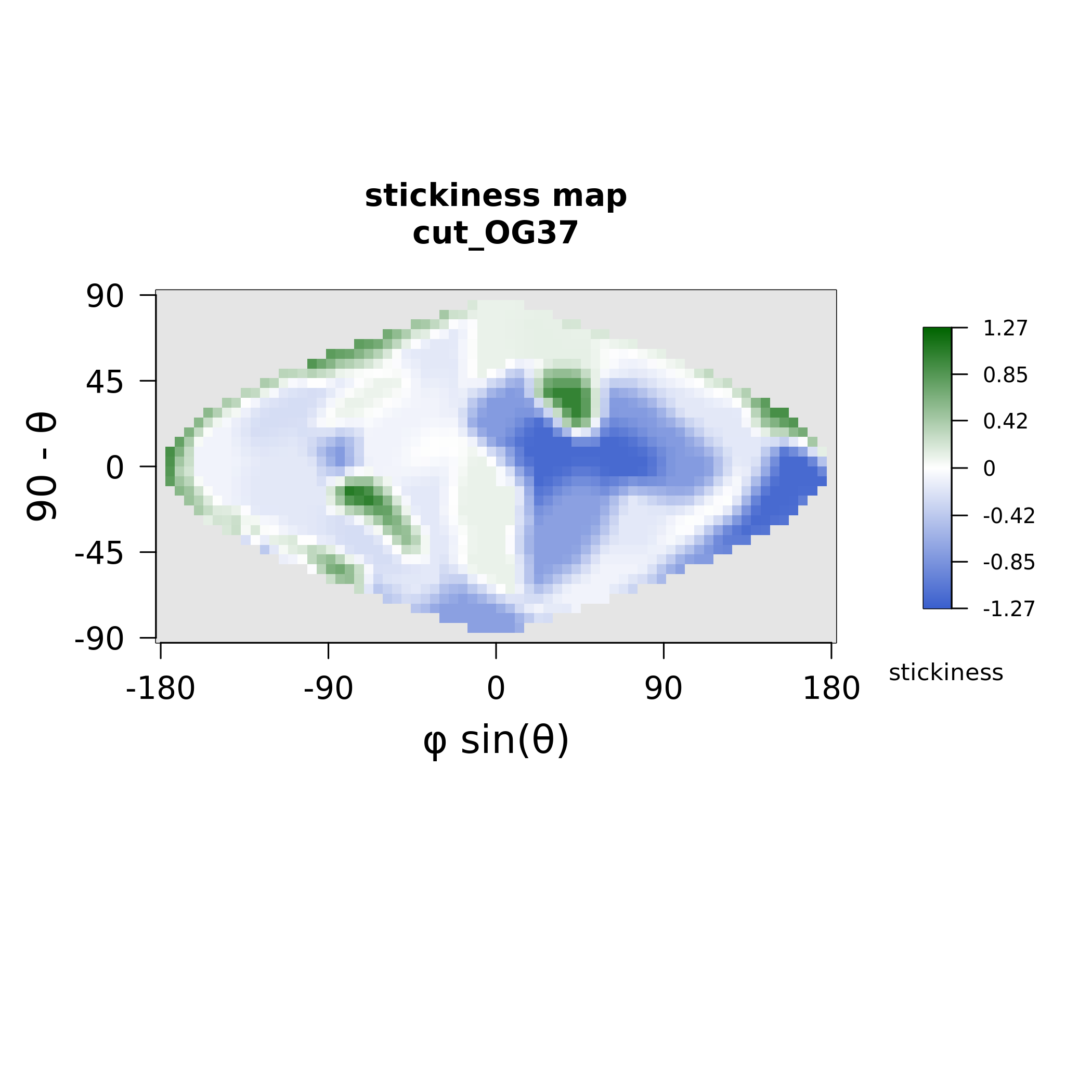

Supplement: S2 File — (ZIP) [file ppat.1012176.s019.zip › S2_File/STICKINESS/MAX37_stickiness.png]

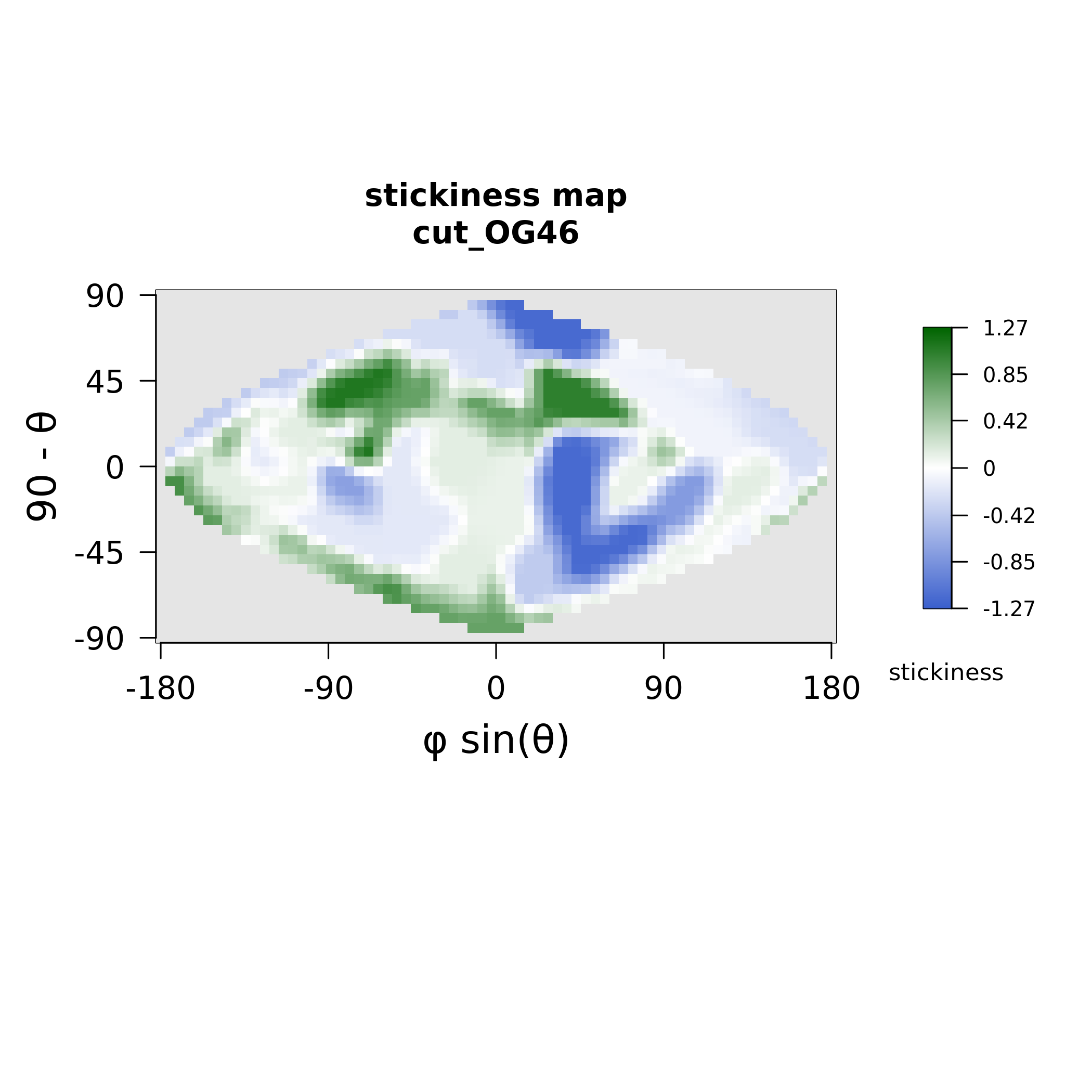

Supplement: S2 File — (ZIP) [file ppat.1012176.s019.zip › S2_File/STICKINESS/MAX46_stickiness.png]

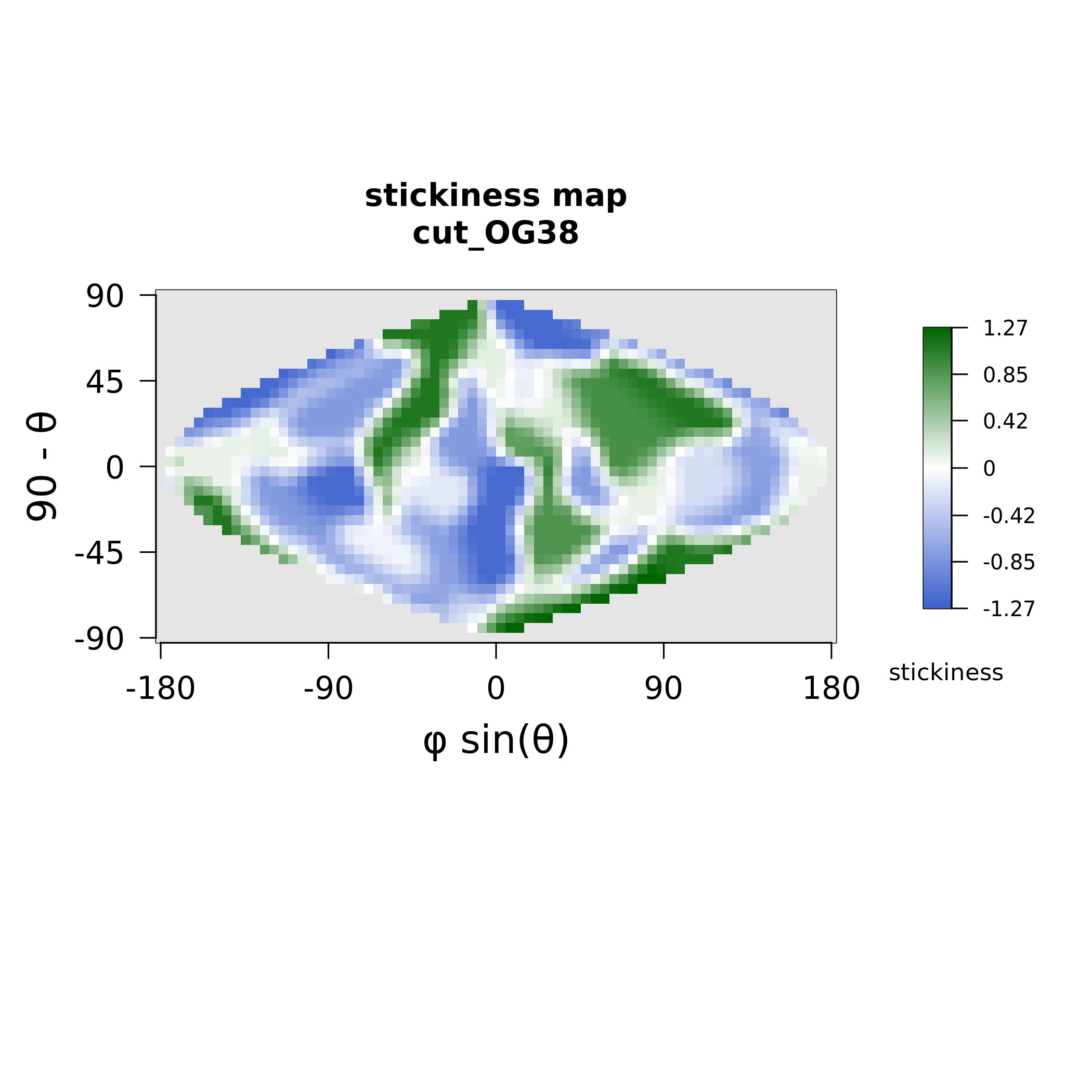

Supplement: S2 File — (ZIP) [file ppat.1012176.s019.zip › S2_File/STICKINESS/MAX38_stickiness.png]

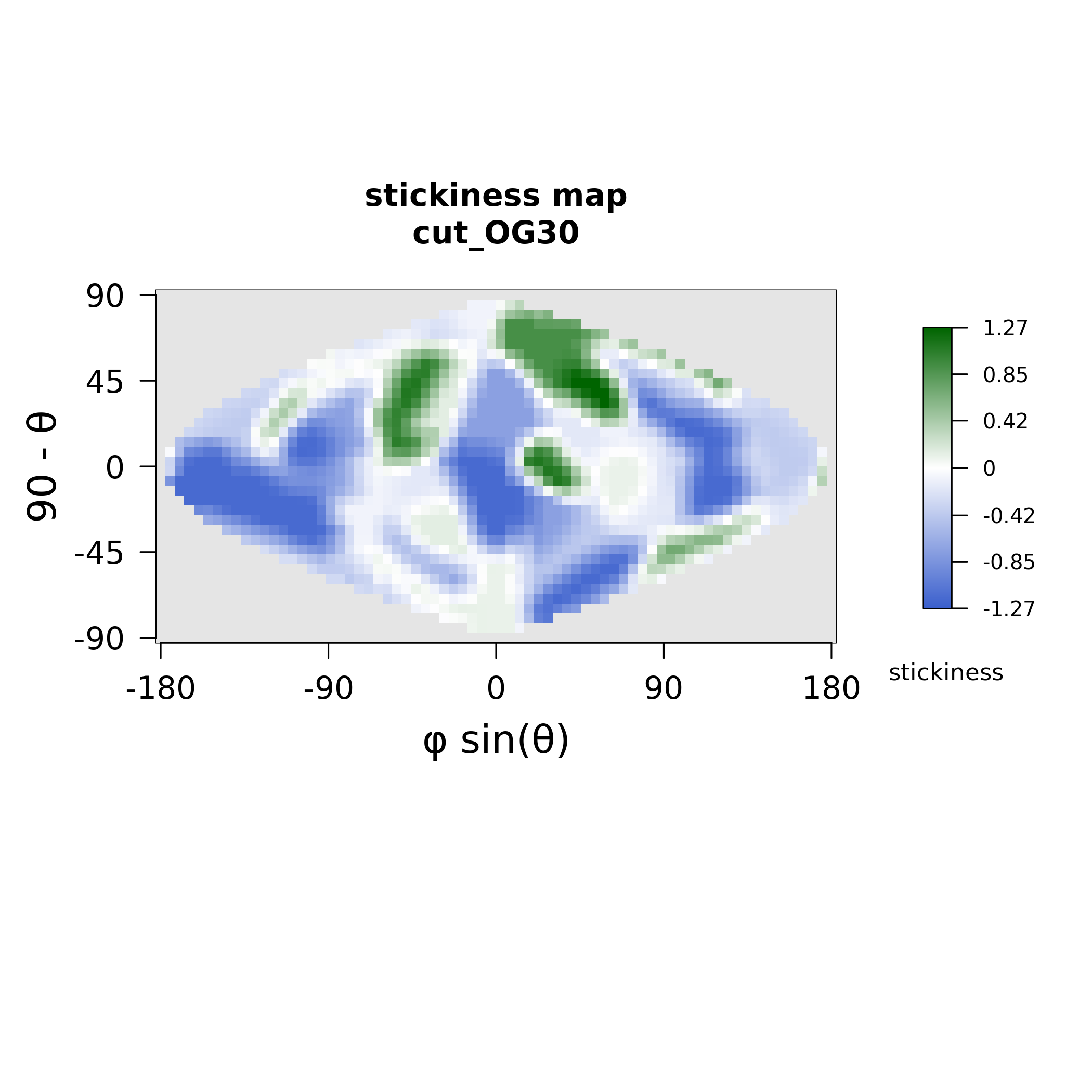

Supplement: S2 File — (ZIP) [file ppat.1012176.s019.zip › S2_File/STICKINESS/MAX30_stickiness.png]

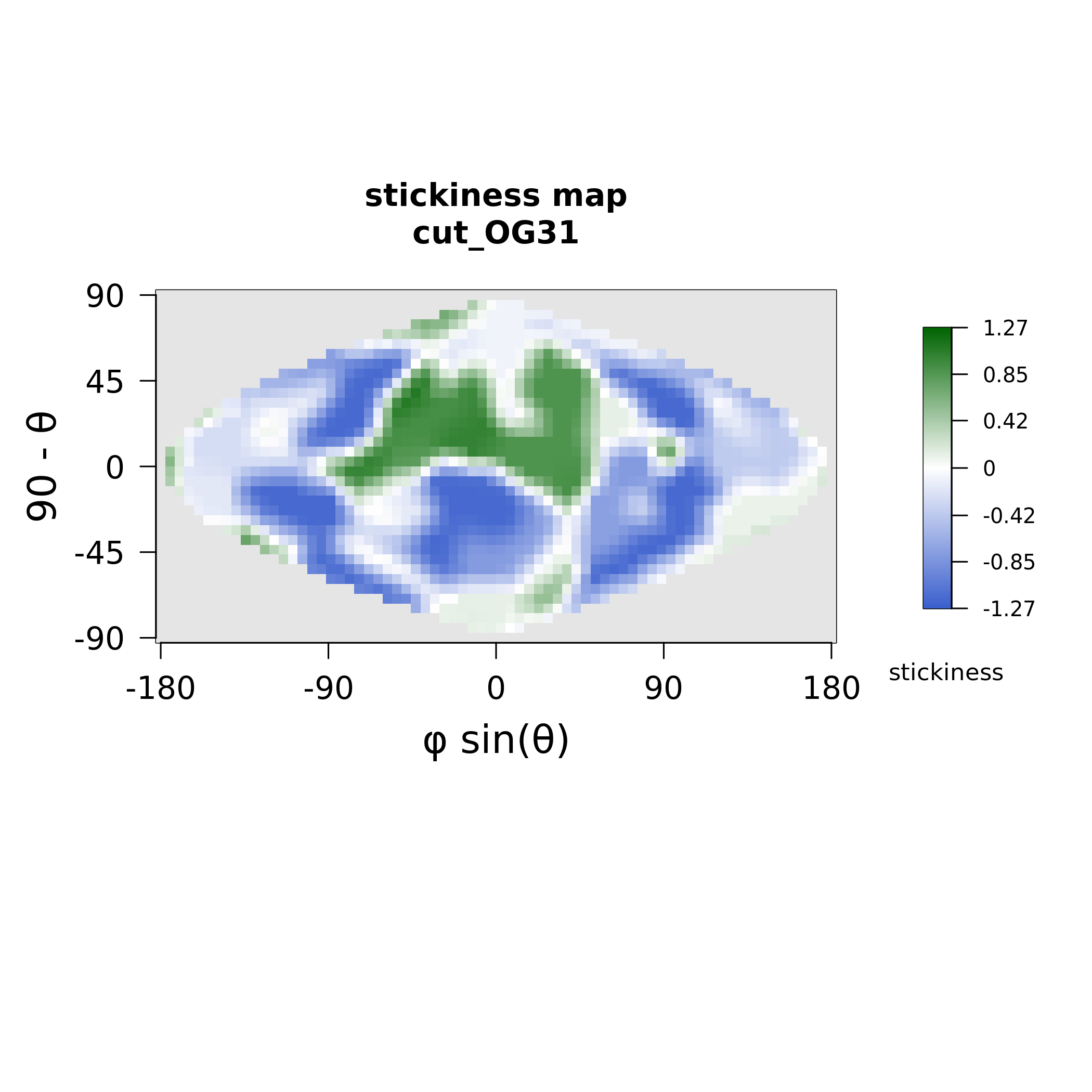

Supplement: S2 File — (ZIP) [file ppat.1012176.s019.zip › S2_File/STICKINESS/MAX31_stickiness.png]

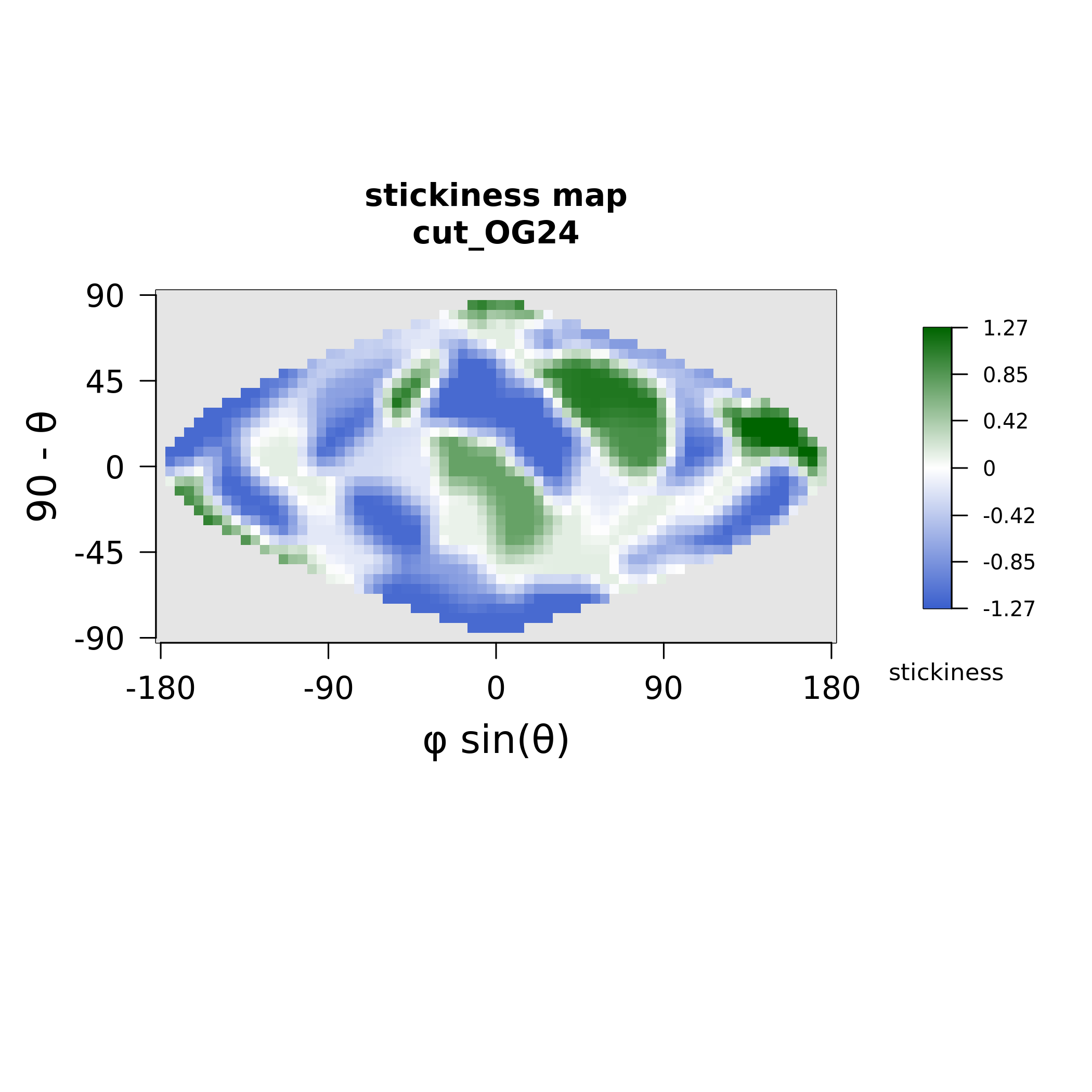

Supplement: S2 File — (ZIP) [file ppat.1012176.s019.zip › S2_File/STICKINESS/MAX24_stickiness.png]

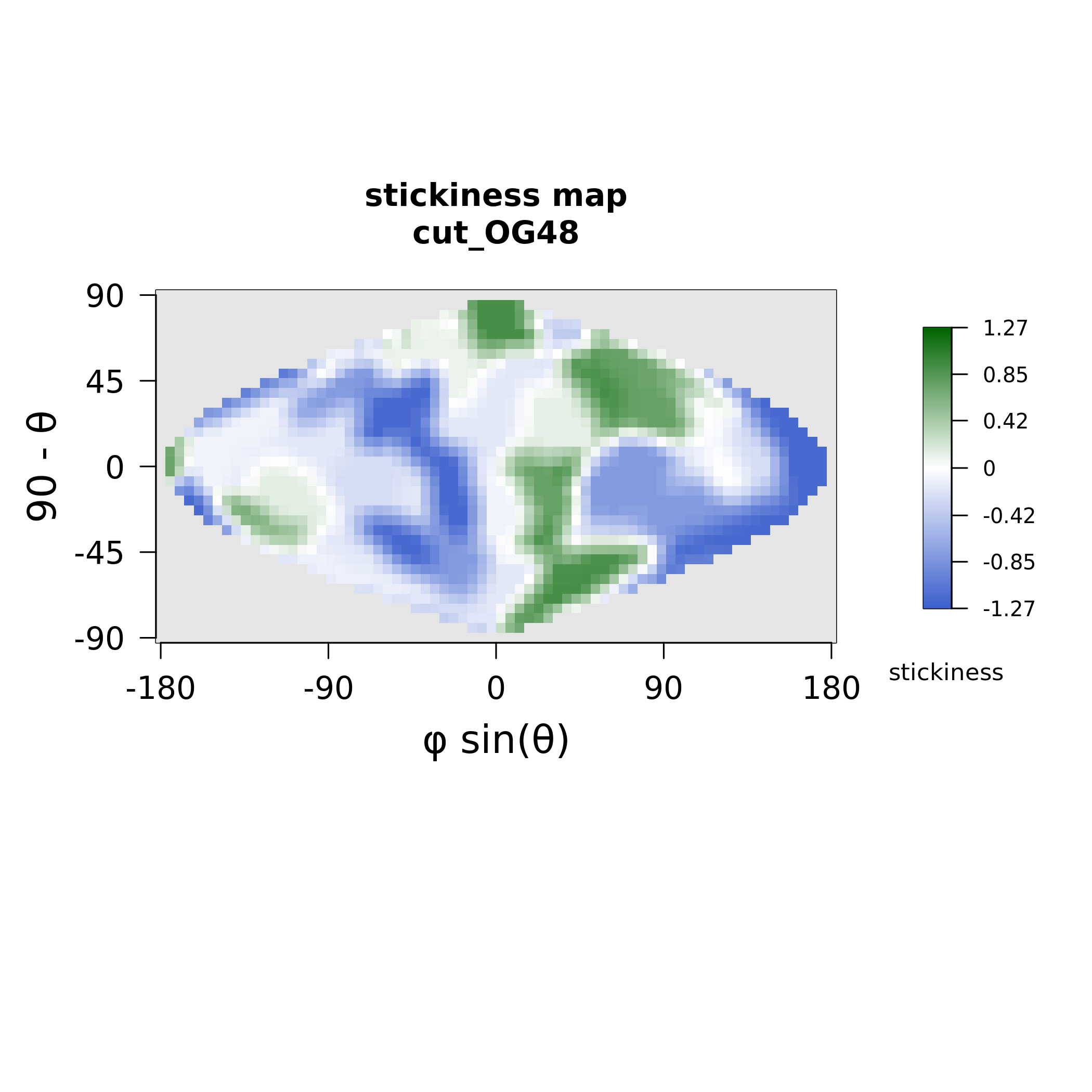

Supplement: S2 File — (ZIP) [file ppat.1012176.s019.zip › S2_File/STICKINESS/MAX48_stickiness.png]

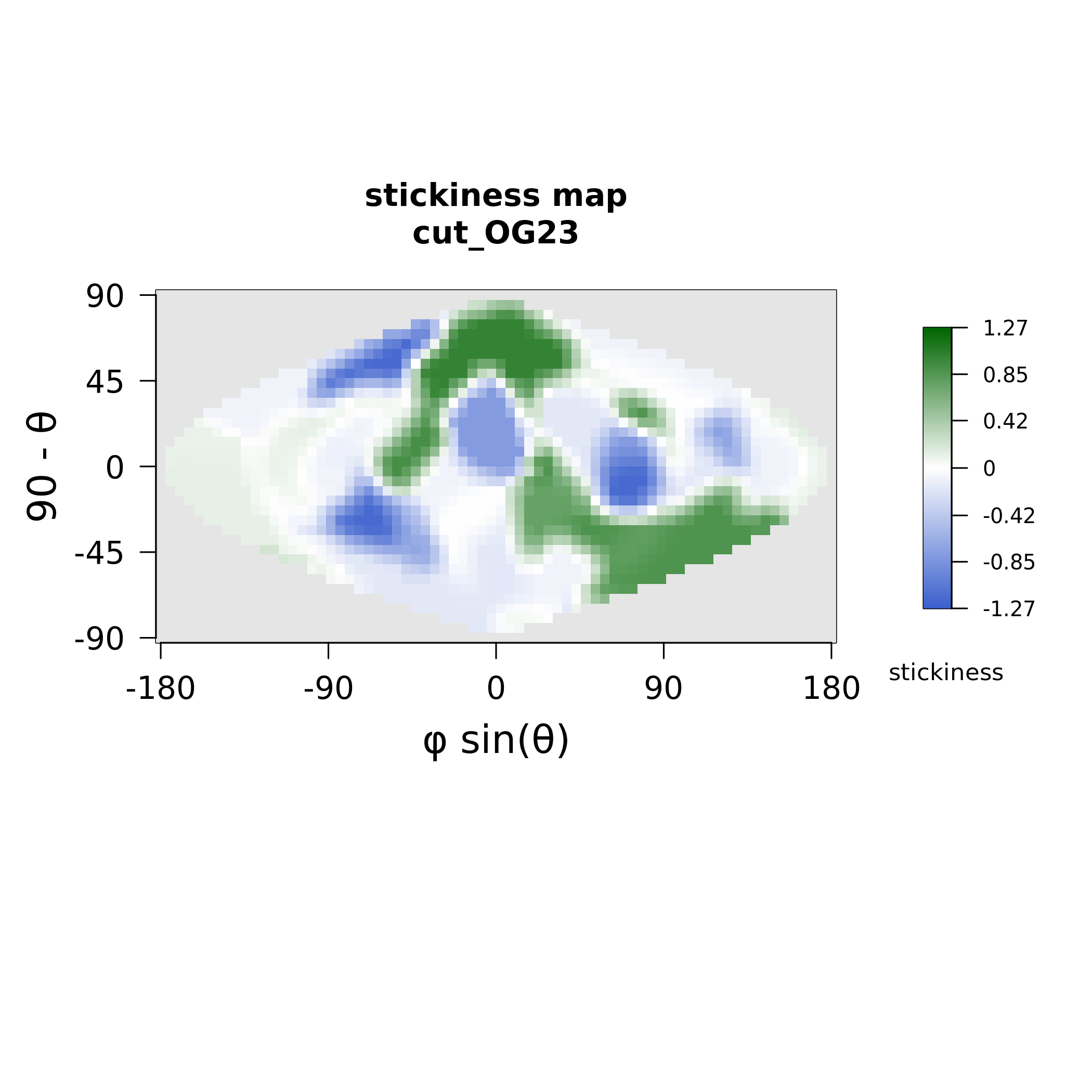

Supplement: S2 File — (ZIP) [file ppat.1012176.s019.zip › S2_File/STICKINESS/MAX23_stickiness.png]

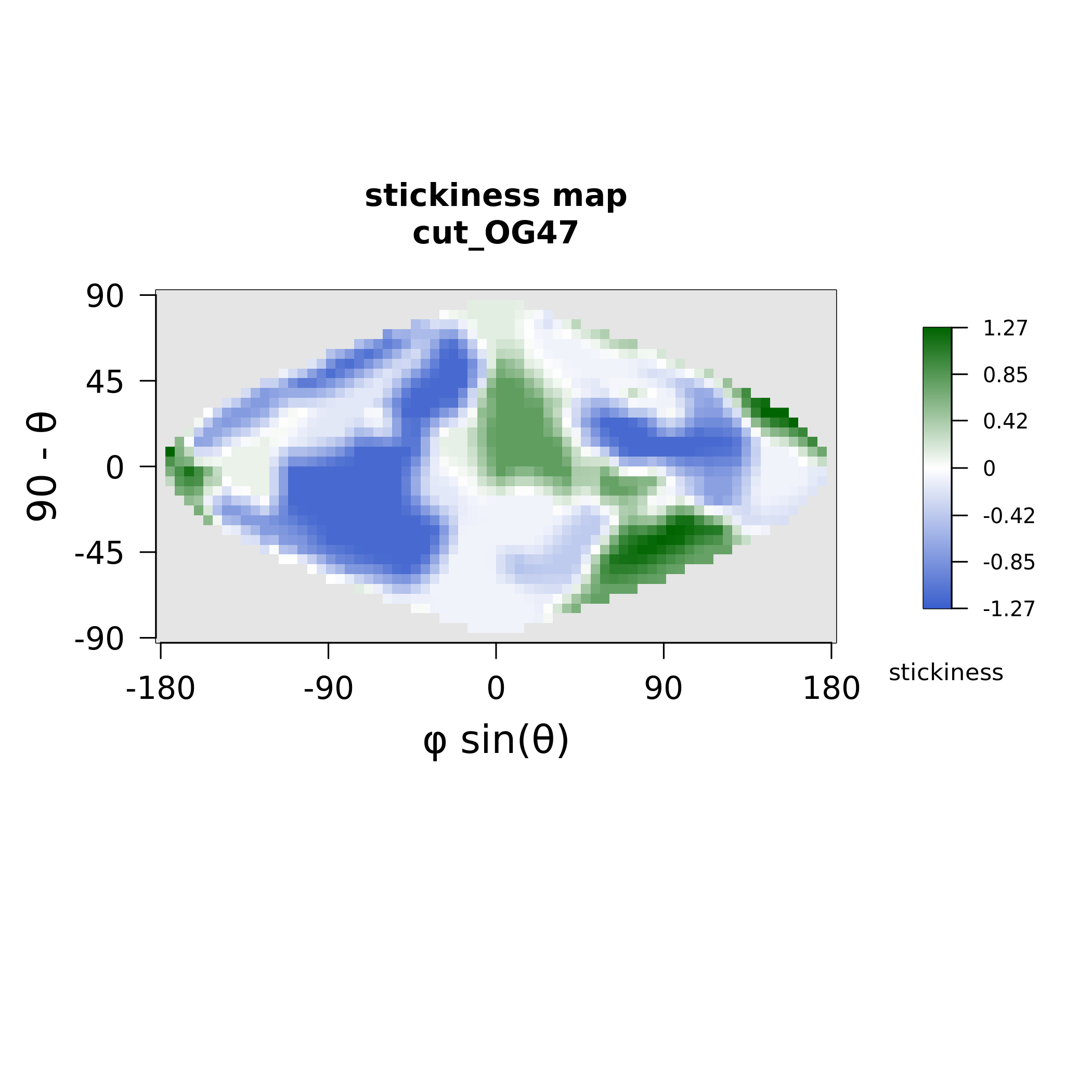

Supplement: S2 File — (ZIP) [file ppat.1012176.s019.zip › S2_File/STICKINESS/MAX47_stickiness.png]

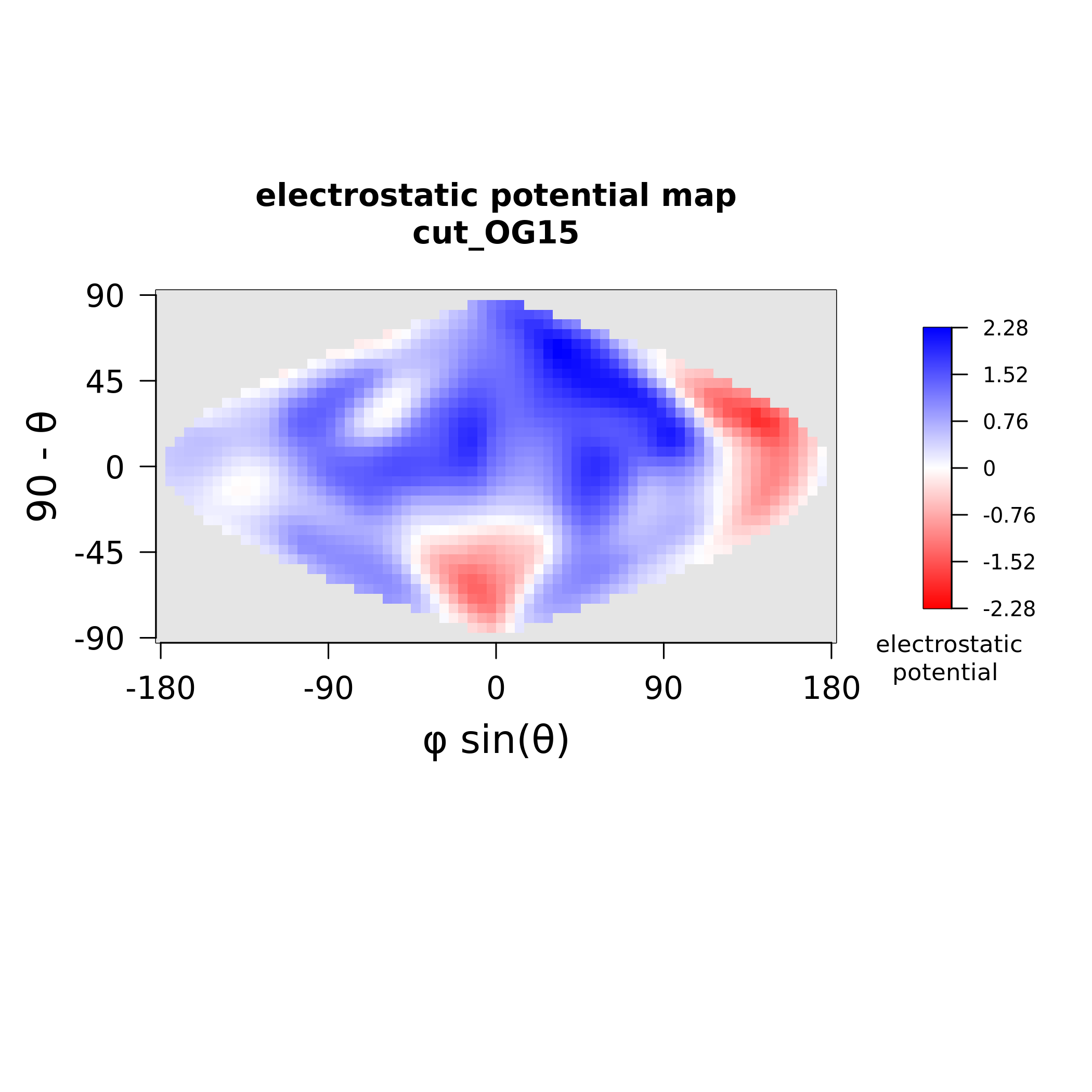

Supplement: S2 File — (ZIP) [file ppat.1012176.s019.zip › S2_File/ELECTROSTATICS/MAX15_electrostatics.png]

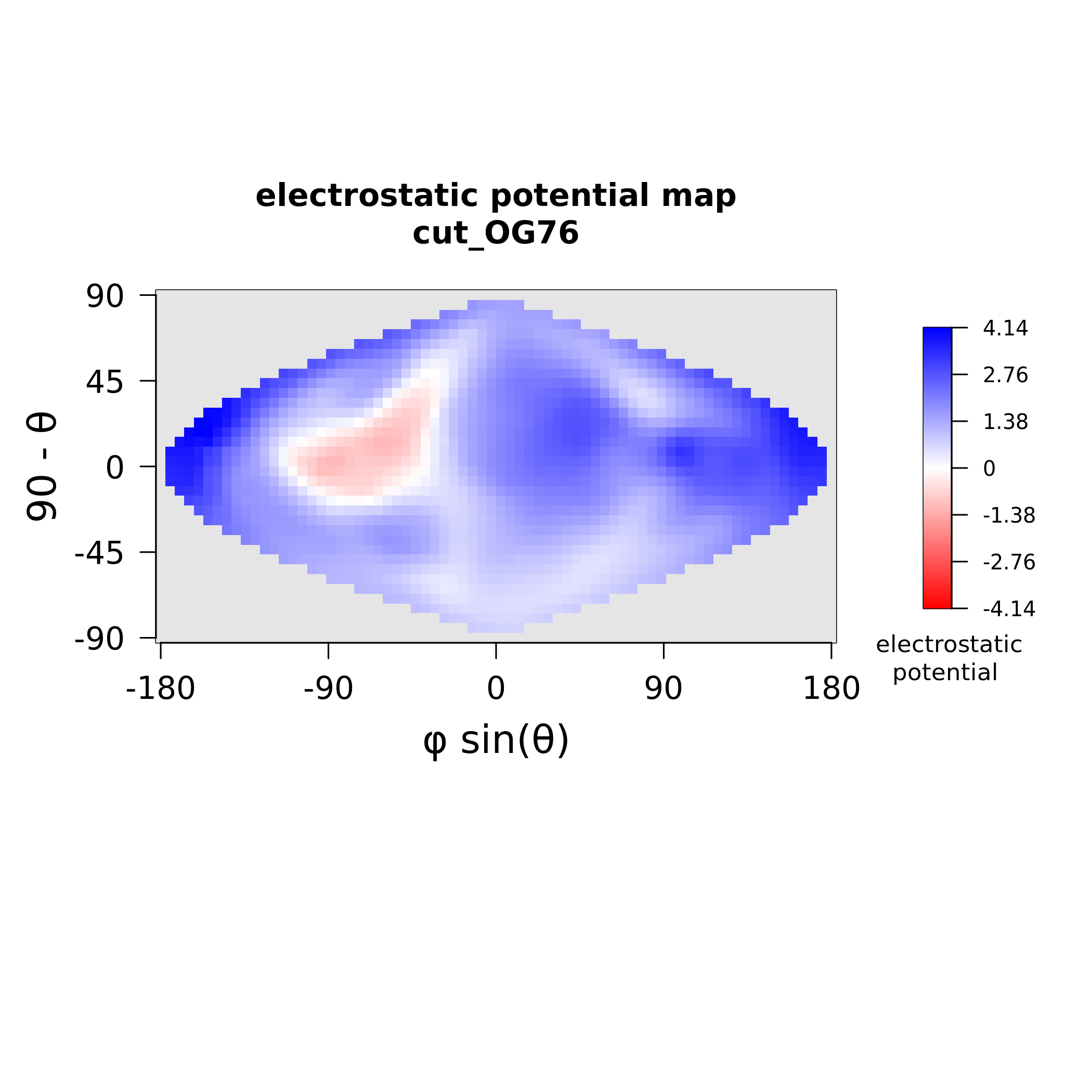

Supplement: S2 File — (ZIP) [file ppat.1012176.s019.zip › S2_File/ELECTROSTATICS/MAX76_electrostatics.png]

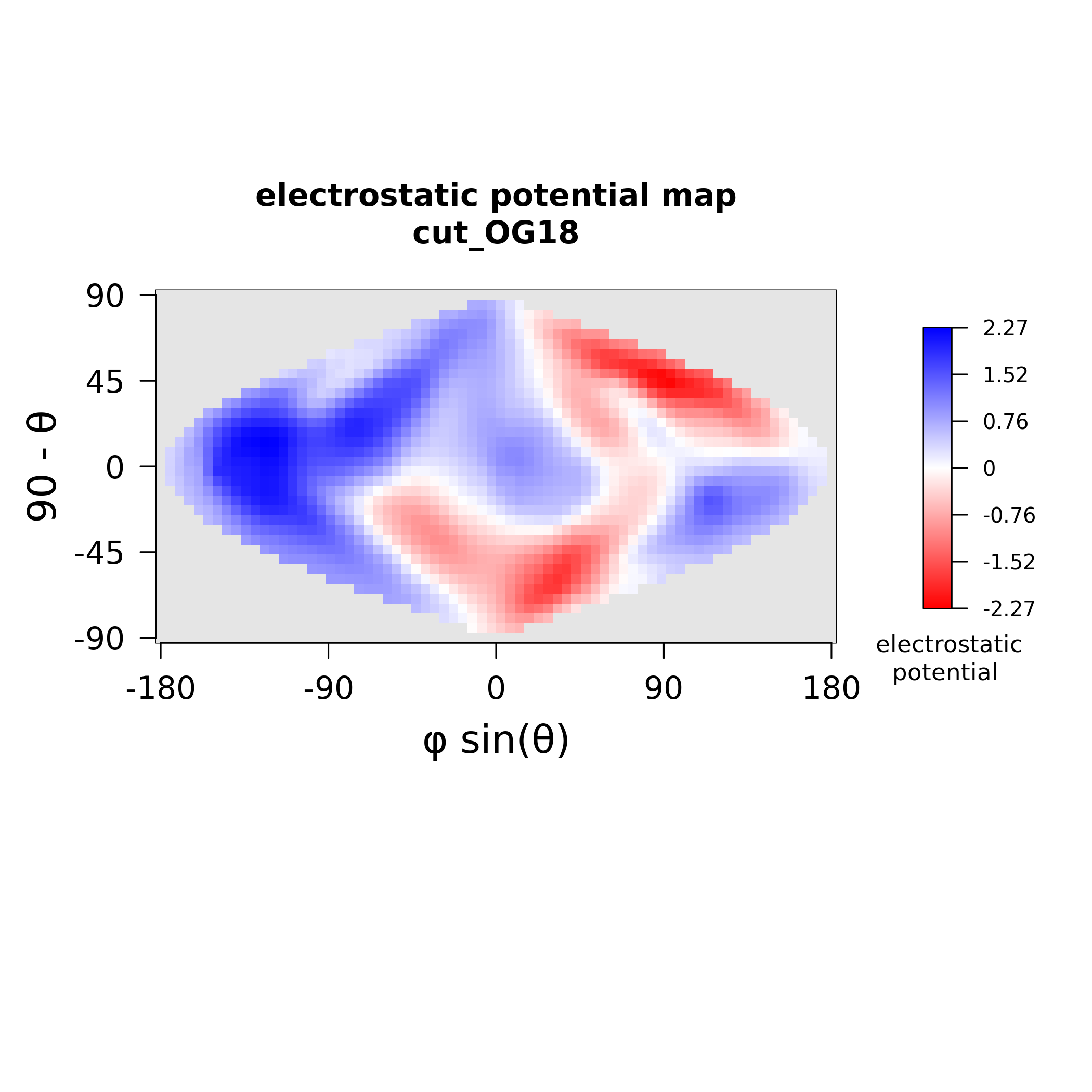

Supplement: S2 File — (ZIP) [file ppat.1012176.s019.zip › S2_File/ELECTROSTATICS/MAX18_electrostatics.png]

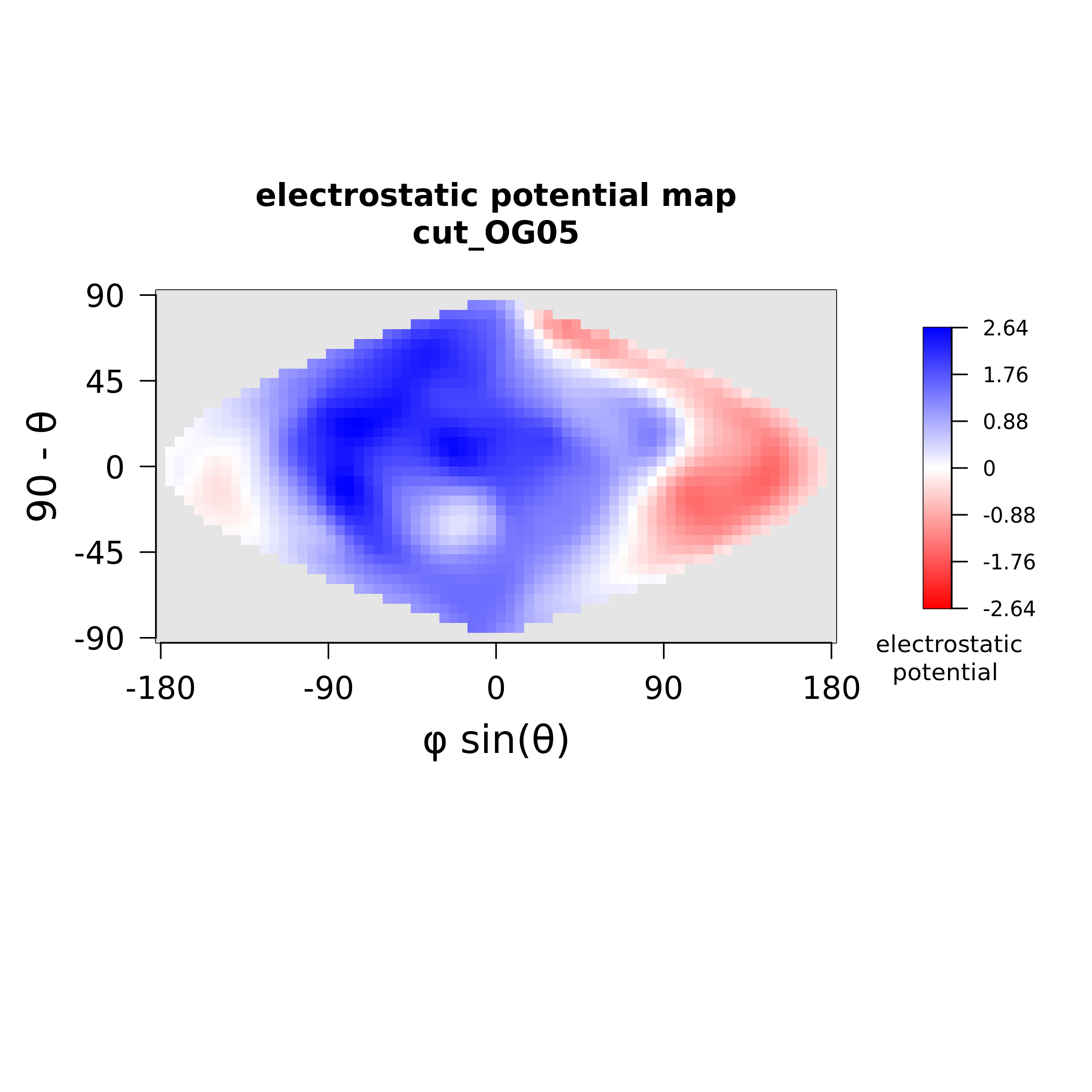

Supplement: S2 File — (ZIP) [file ppat.1012176.s019.zip › S2_File/ELECTROSTATICS/MAX05_electrostatics.png]

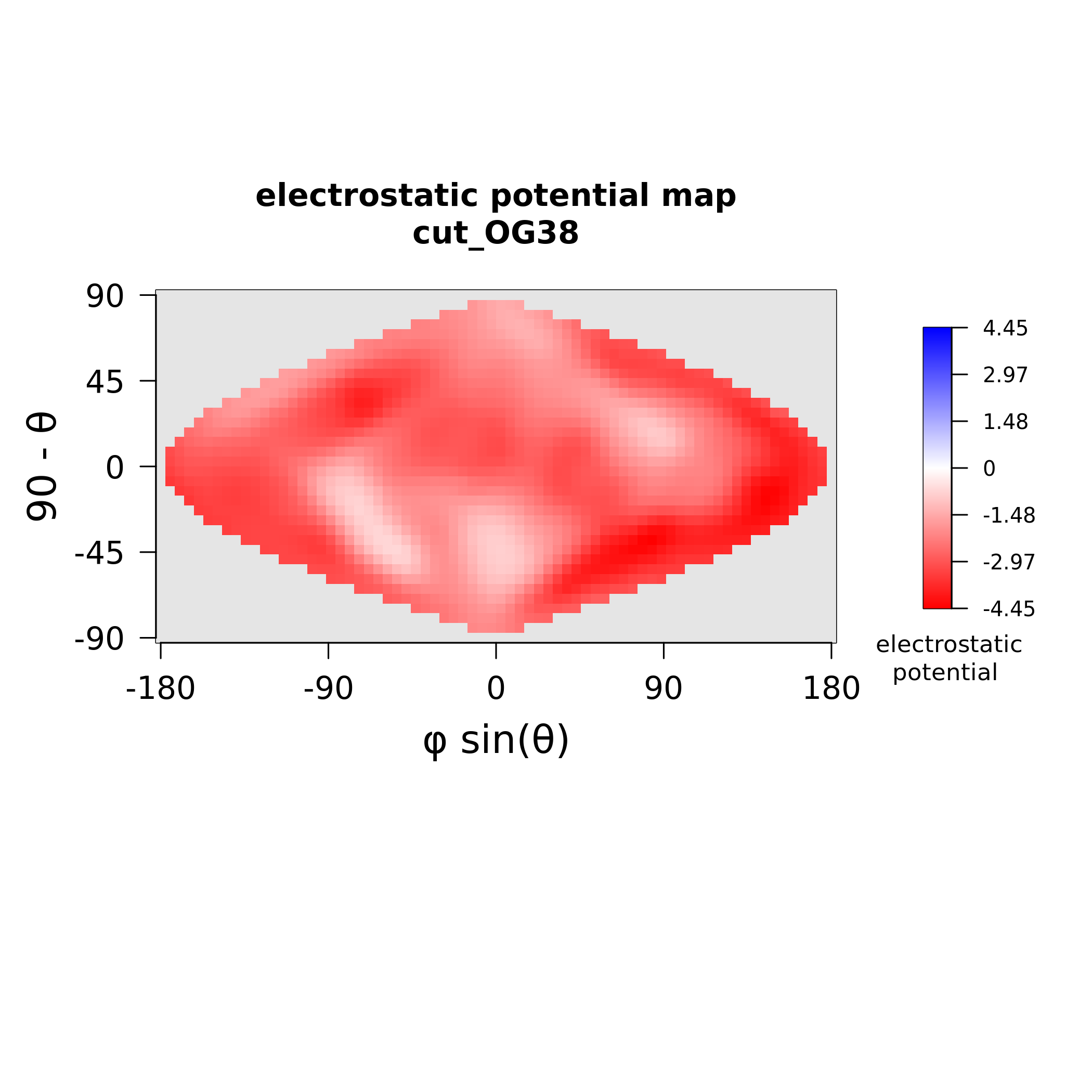

Supplement: S2 File — (ZIP) [file ppat.1012176.s019.zip › S2_File/ELECTROSTATICS/MAX38_electrostatics.png]

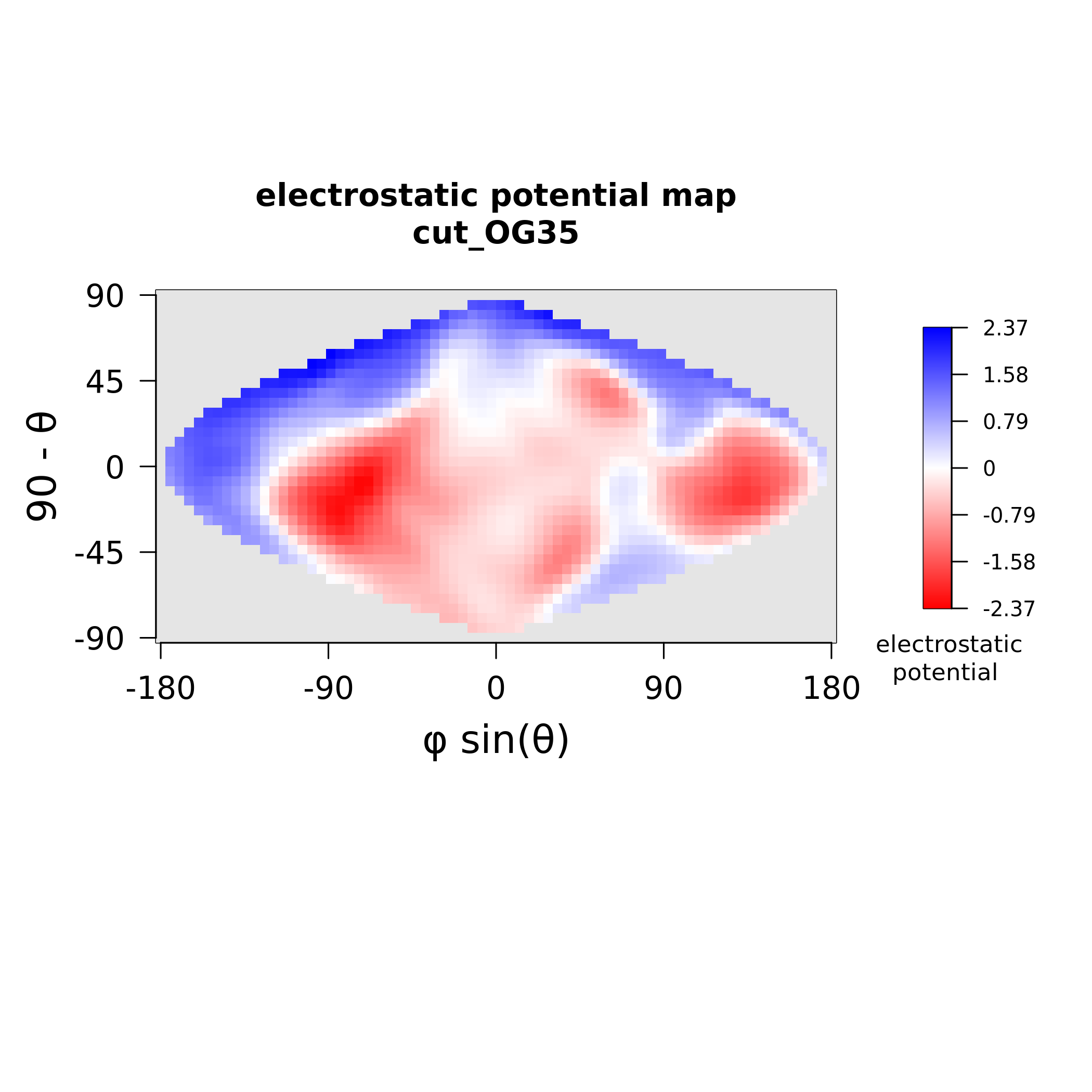

Supplement: S2 File — (ZIP) [file ppat.1012176.s019.zip › S2_File/ELECTROSTATICS/MAX35_electrostatics.png]

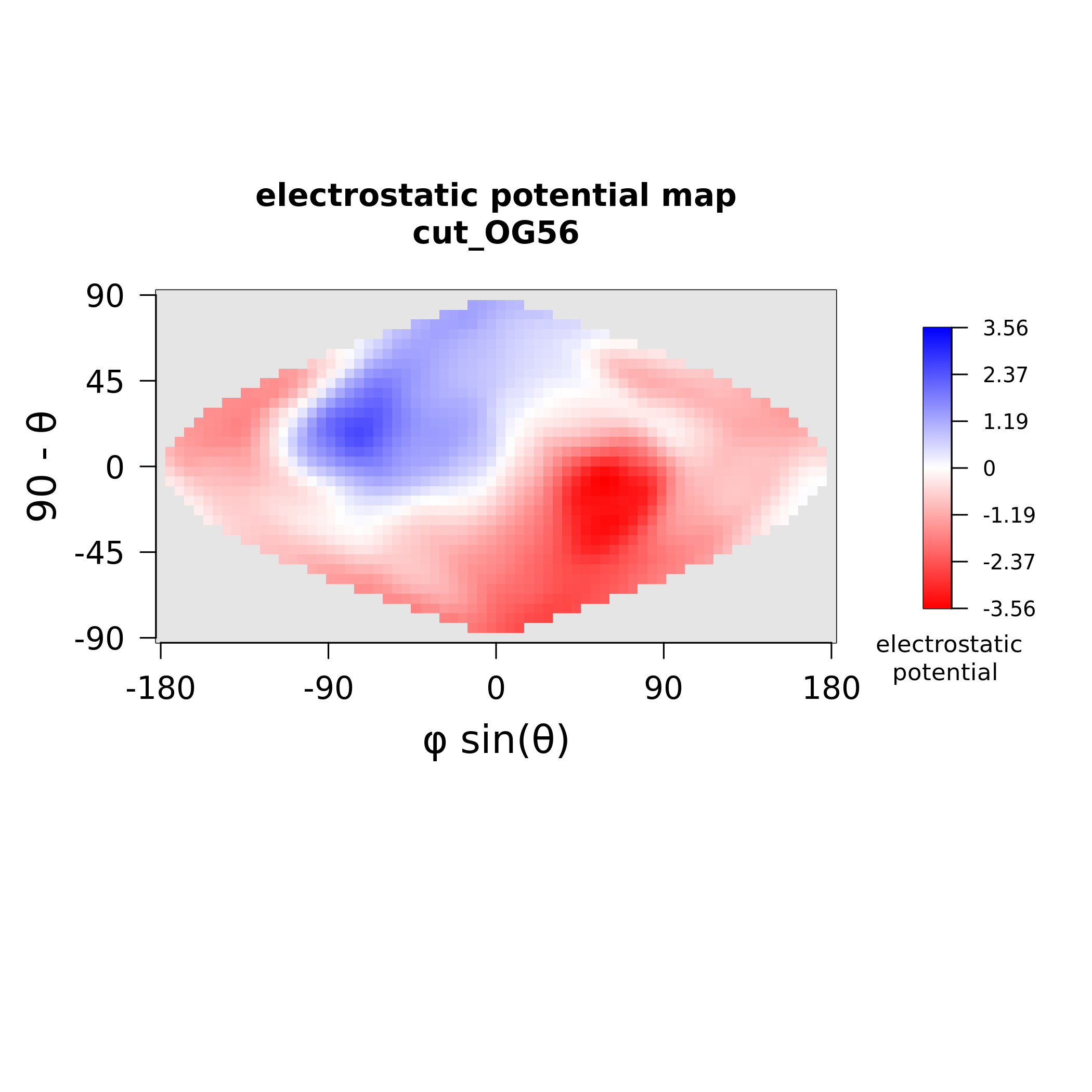

Supplement: S2 File — (ZIP) [file ppat.1012176.s019.zip › S2_File/ELECTROSTATICS/MAX56_electrostatics.png]

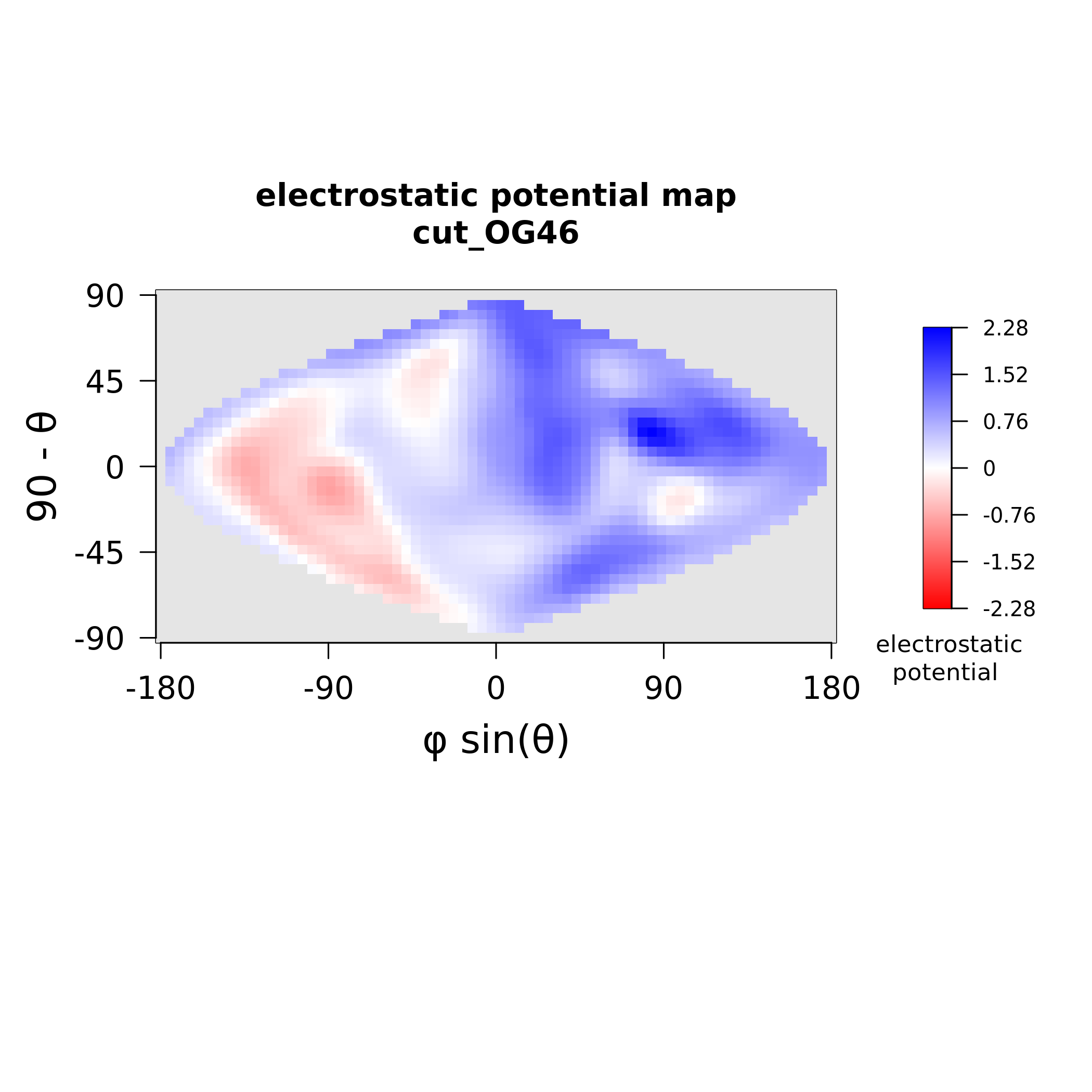

Supplement: S2 File — (ZIP) [file ppat.1012176.s019.zip › S2_File/ELECTROSTATICS/MAX46_electrostatics.png]

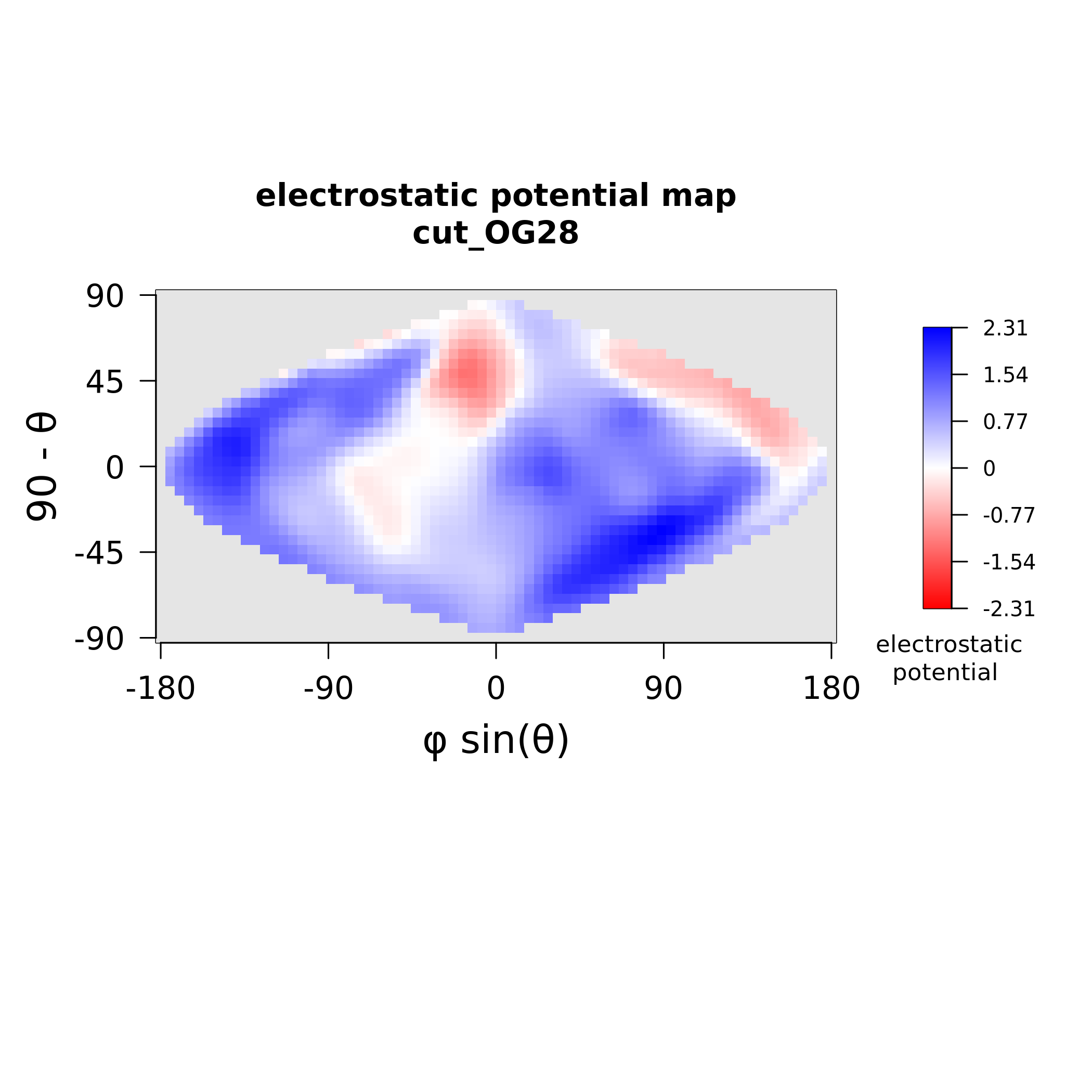

Supplement: S2 File — (ZIP) [file ppat.1012176.s019.zip › S2_File/ELECTROSTATICS/MAX28_electrostatics.png]

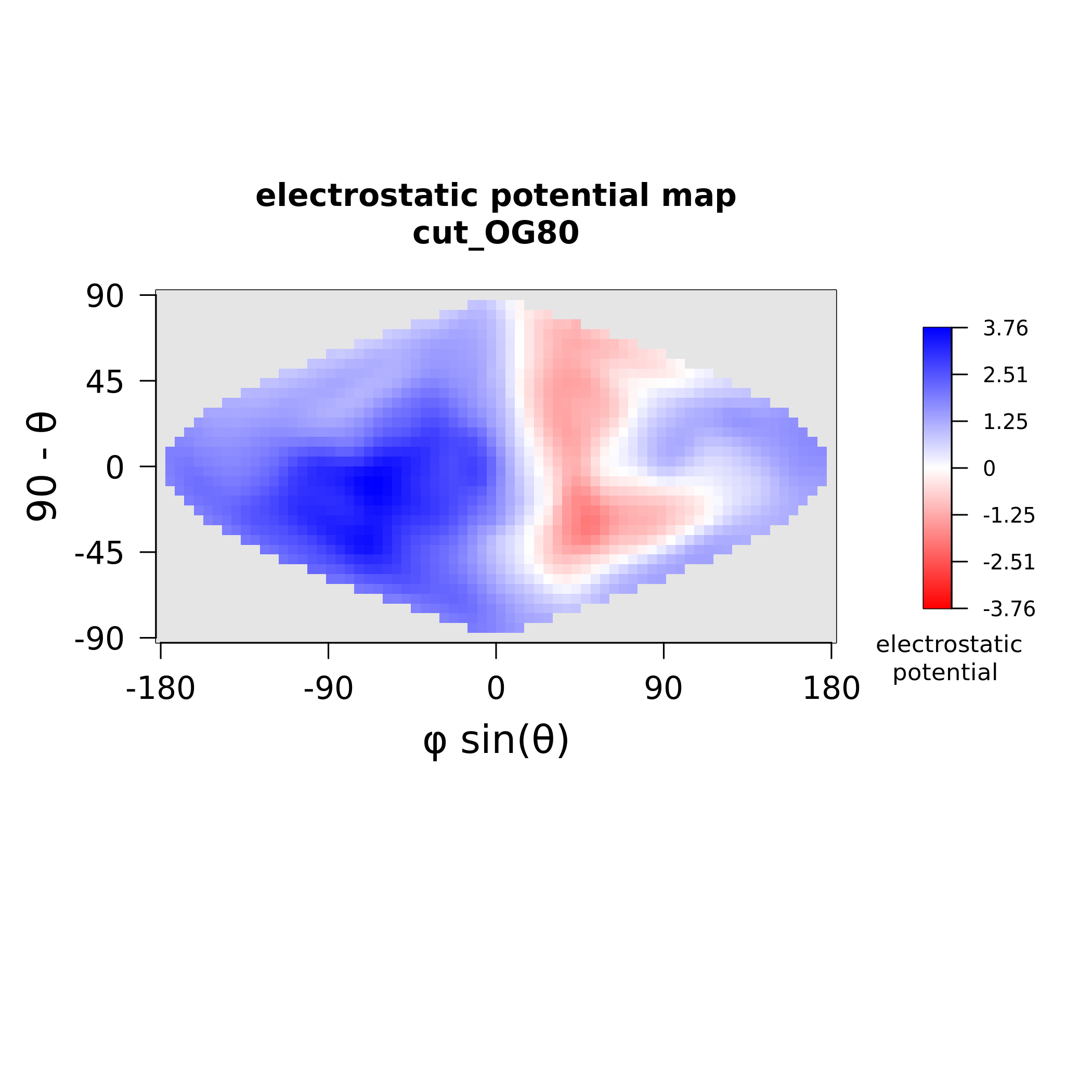

Supplement: S2 File — (ZIP) [file ppat.1012176.s019.zip › S2_File/ELECTROSTATICS/MAX80_electrostatics.png]

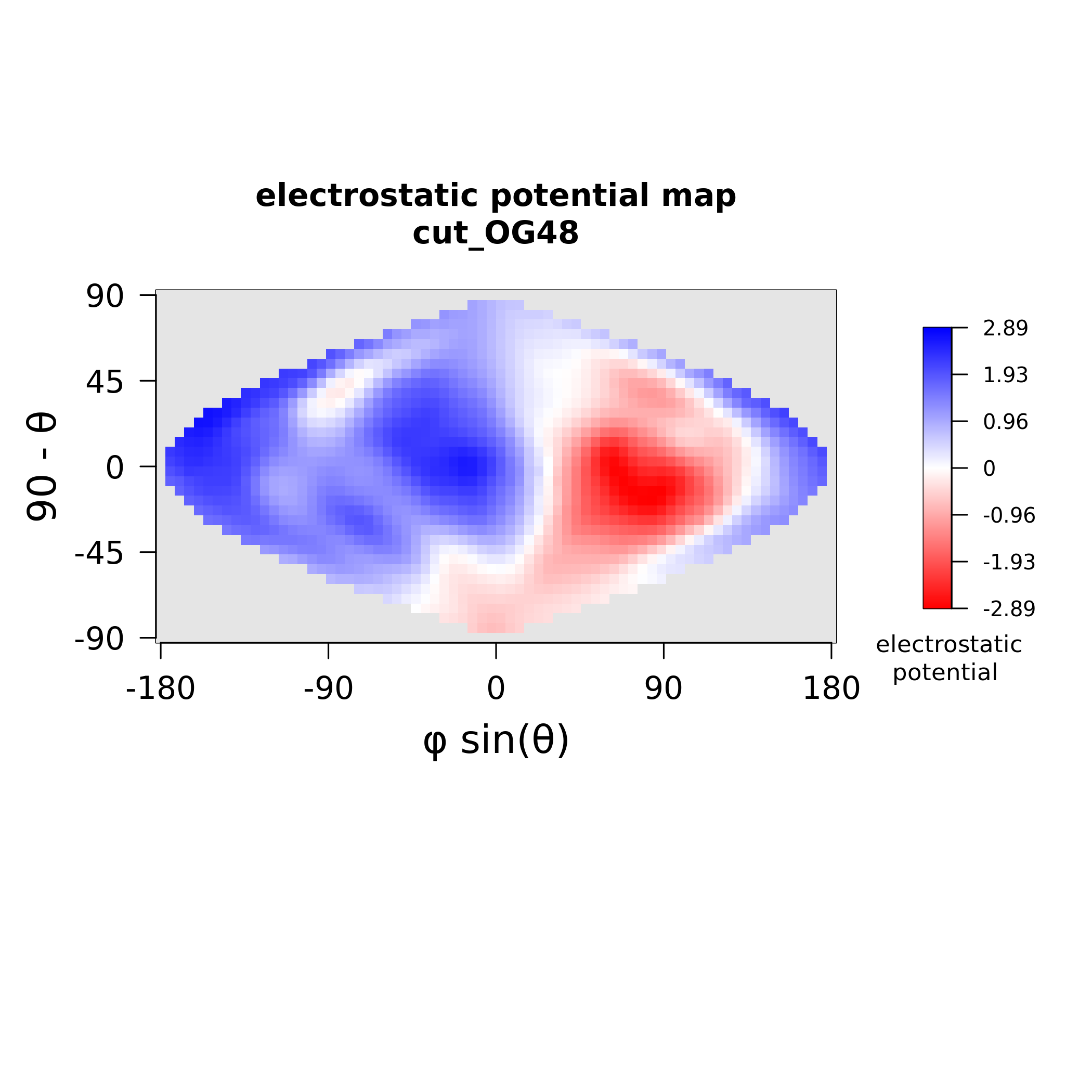

Supplement: S2 File — (ZIP) [file ppat.1012176.s019.zip › S2_File/ELECTROSTATICS/MAX48_electrostatics.png]

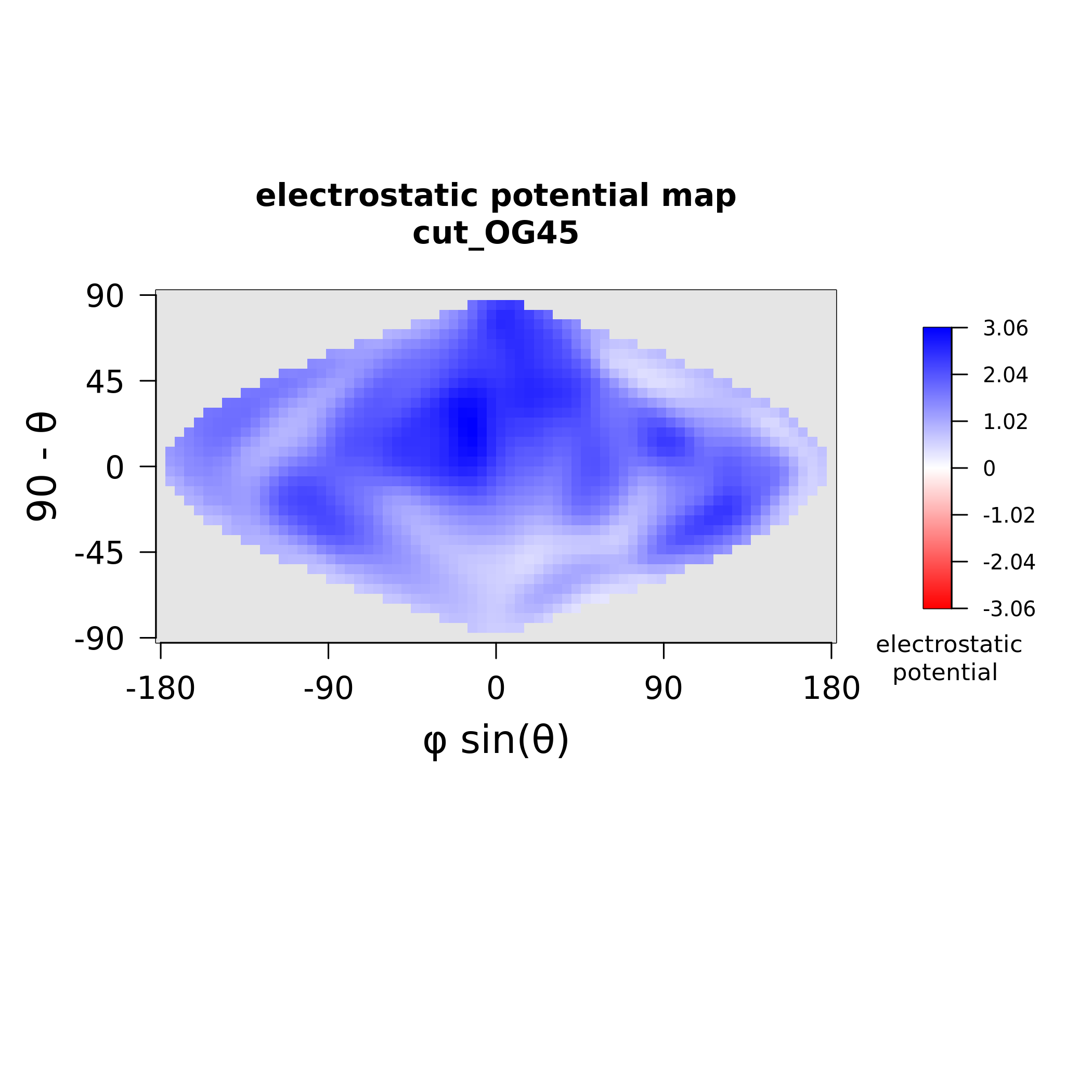

Supplement: S2 File — (ZIP) [file ppat.1012176.s019.zip › S2_File/ELECTROSTATICS/MAX45_electrostatics.png]

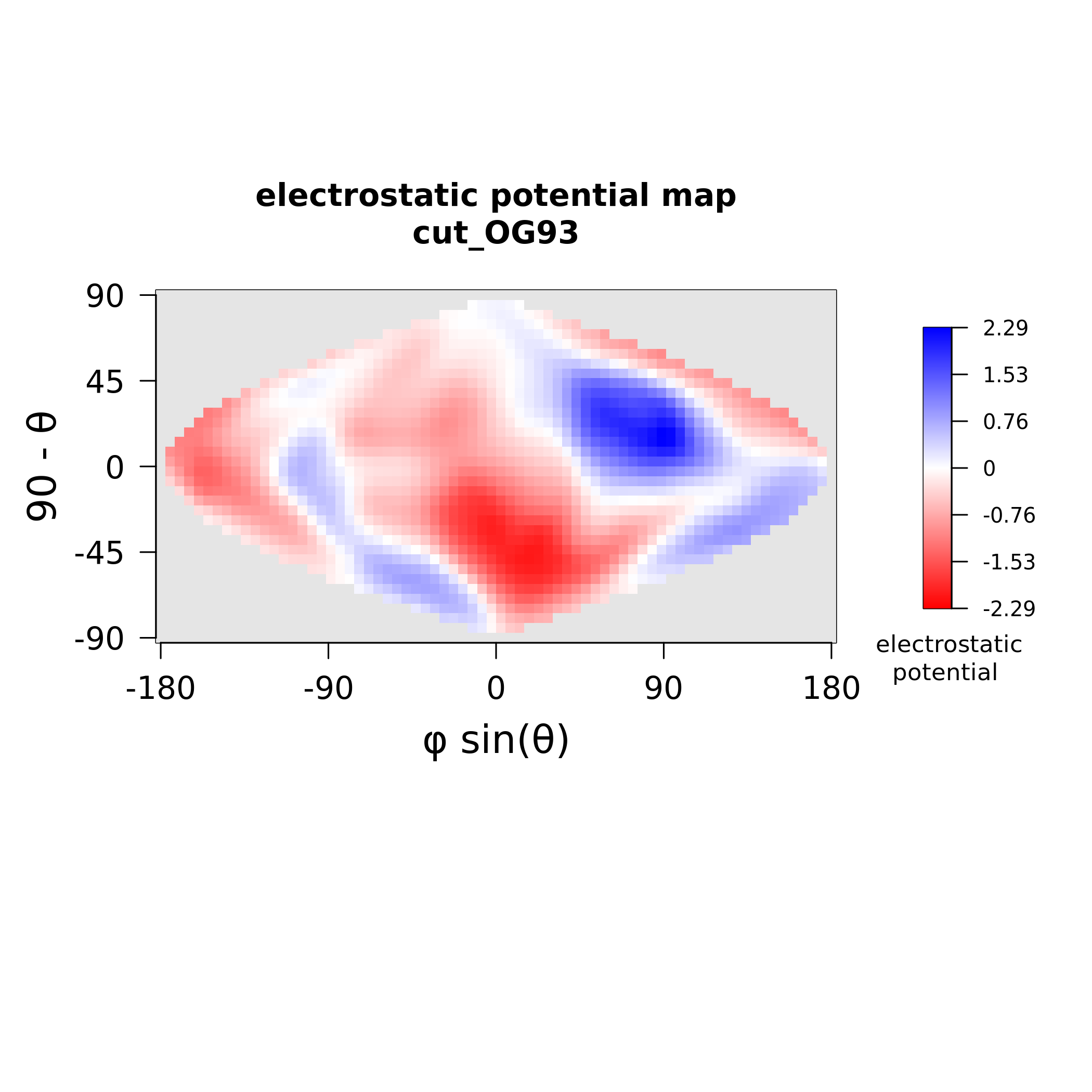

Supplement: S2 File — (ZIP) [file ppat.1012176.s019.zip › S2_File/ELECTROSTATICS/MAX93_electrostatics.png]

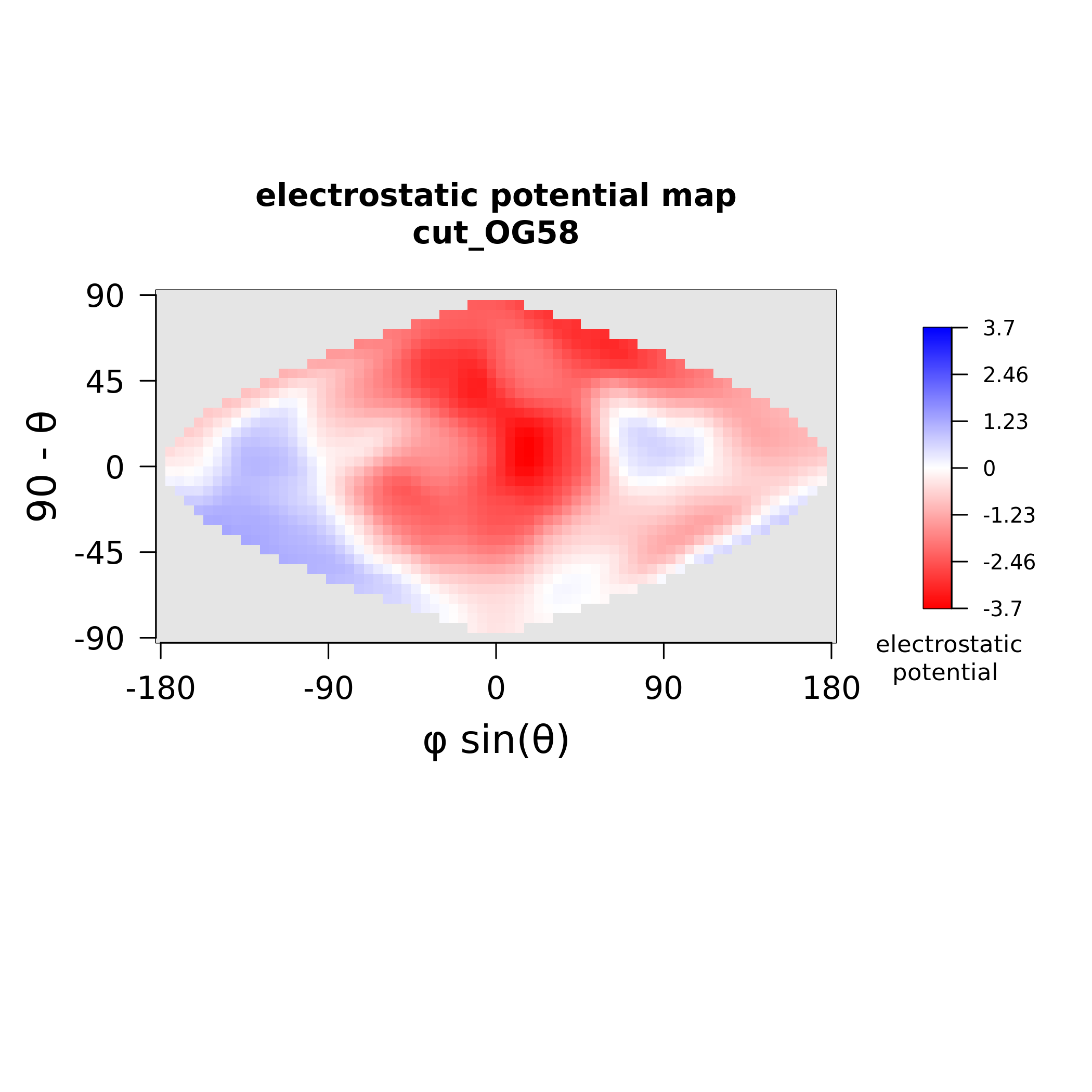

Supplement: S2 File — (ZIP) [file ppat.1012176.s019.zip › S2_File/ELECTROSTATICS/MAX58_electrostatics.png]

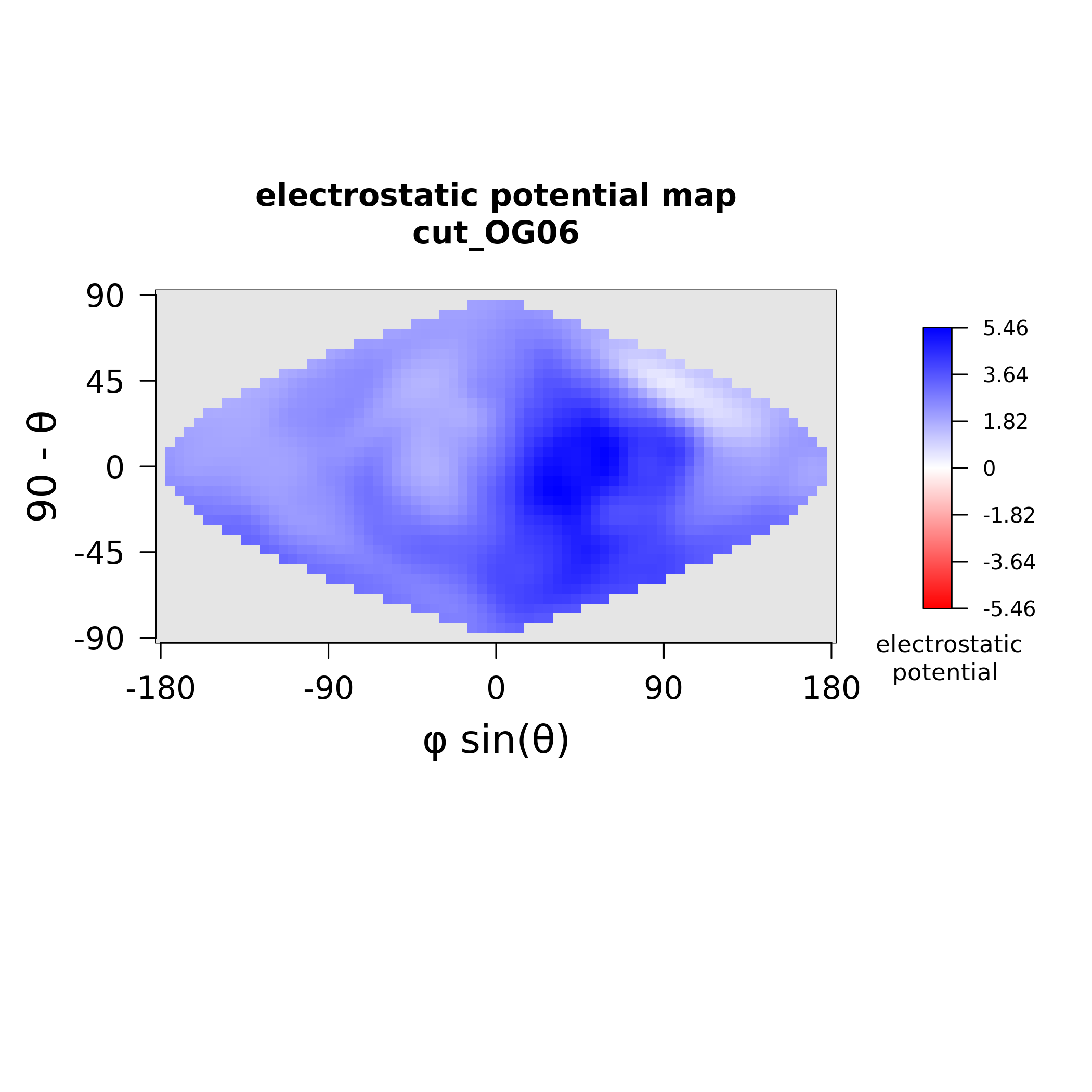

Supplement: S2 File — (ZIP) [file ppat.1012176.s019.zip › S2_File/ELECTROSTATICS/MAX06_electrostatics.png]

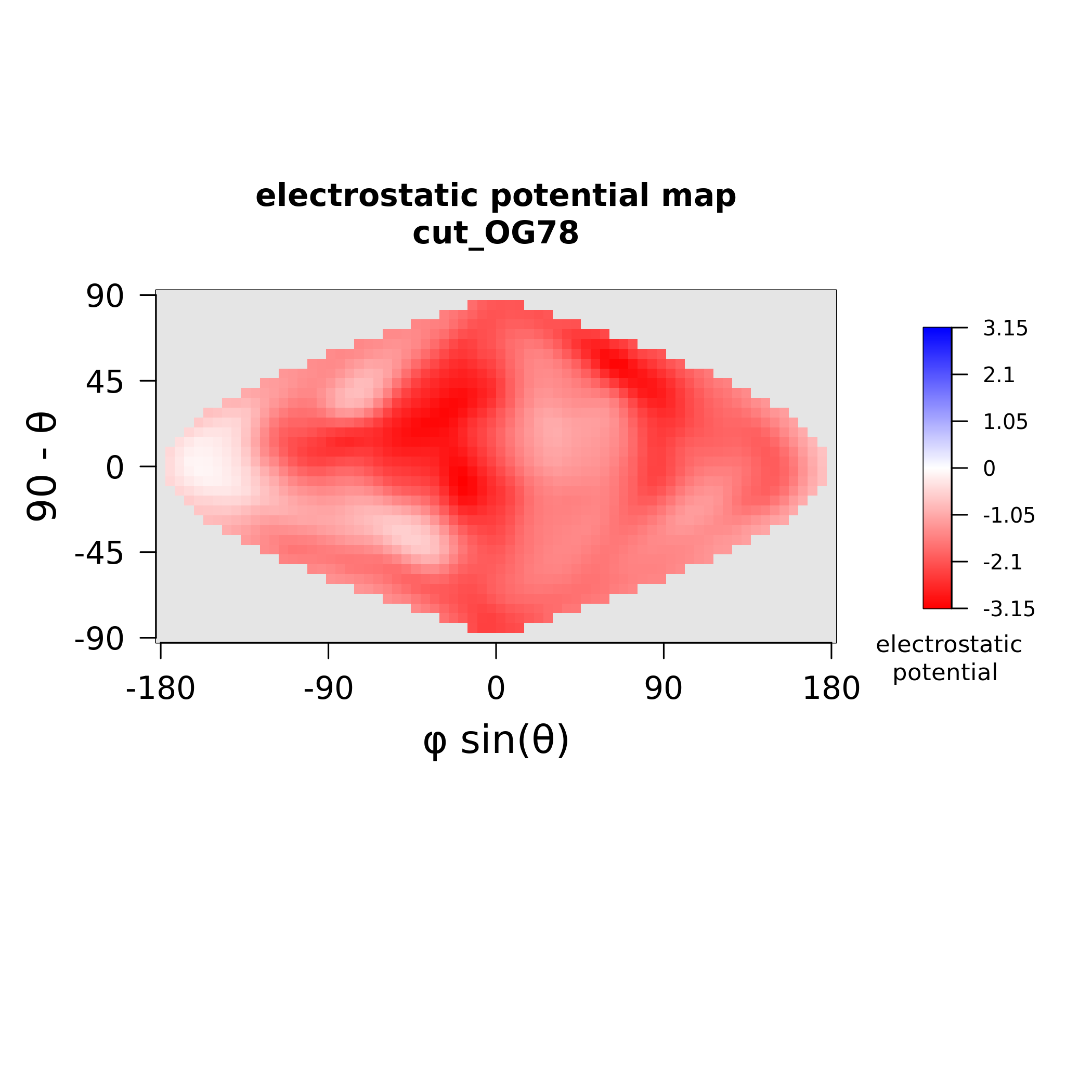

Supplement: S2 File — (ZIP) [file ppat.1012176.s019.zip › S2_File/ELECTROSTATICS/MAX78_electrostatics.png]

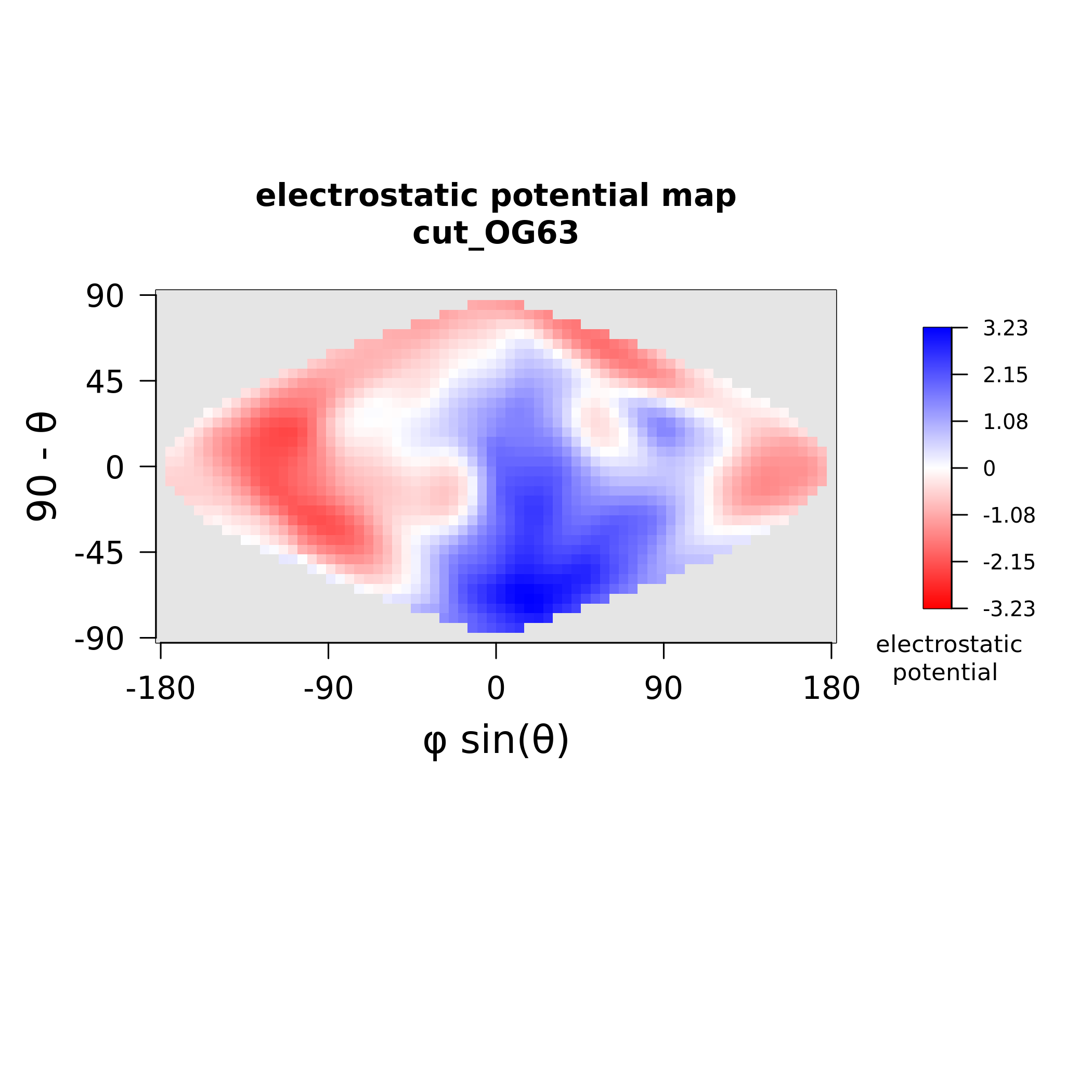

Supplement: S2 File — (ZIP) [file ppat.1012176.s019.zip › S2_File/ELECTROSTATICS/MAX63_electrostatics.png]

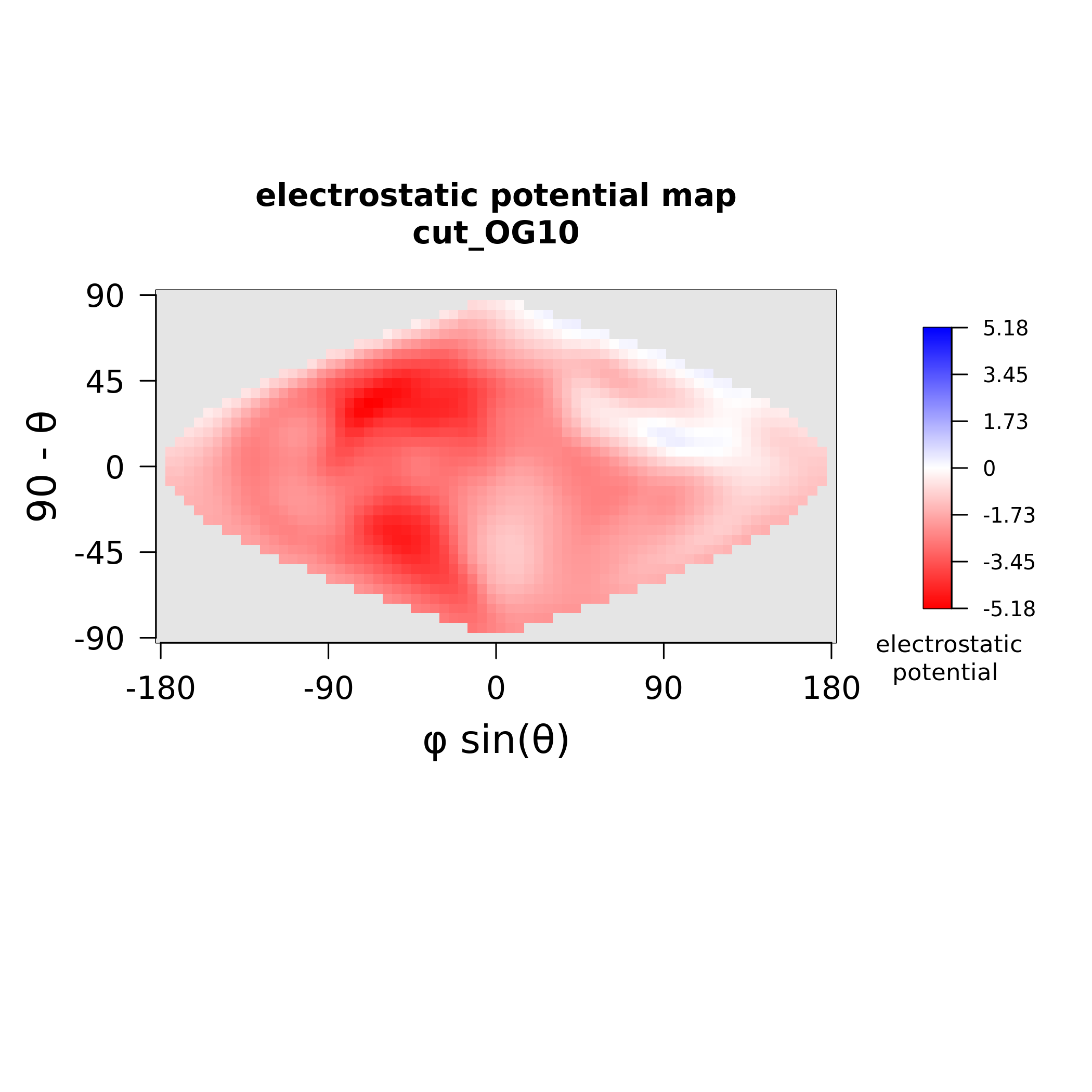

Supplement: S2 File — (ZIP) [file ppat.1012176.s019.zip › S2_File/ELECTROSTATICS/MAX10_electrostatics.png]

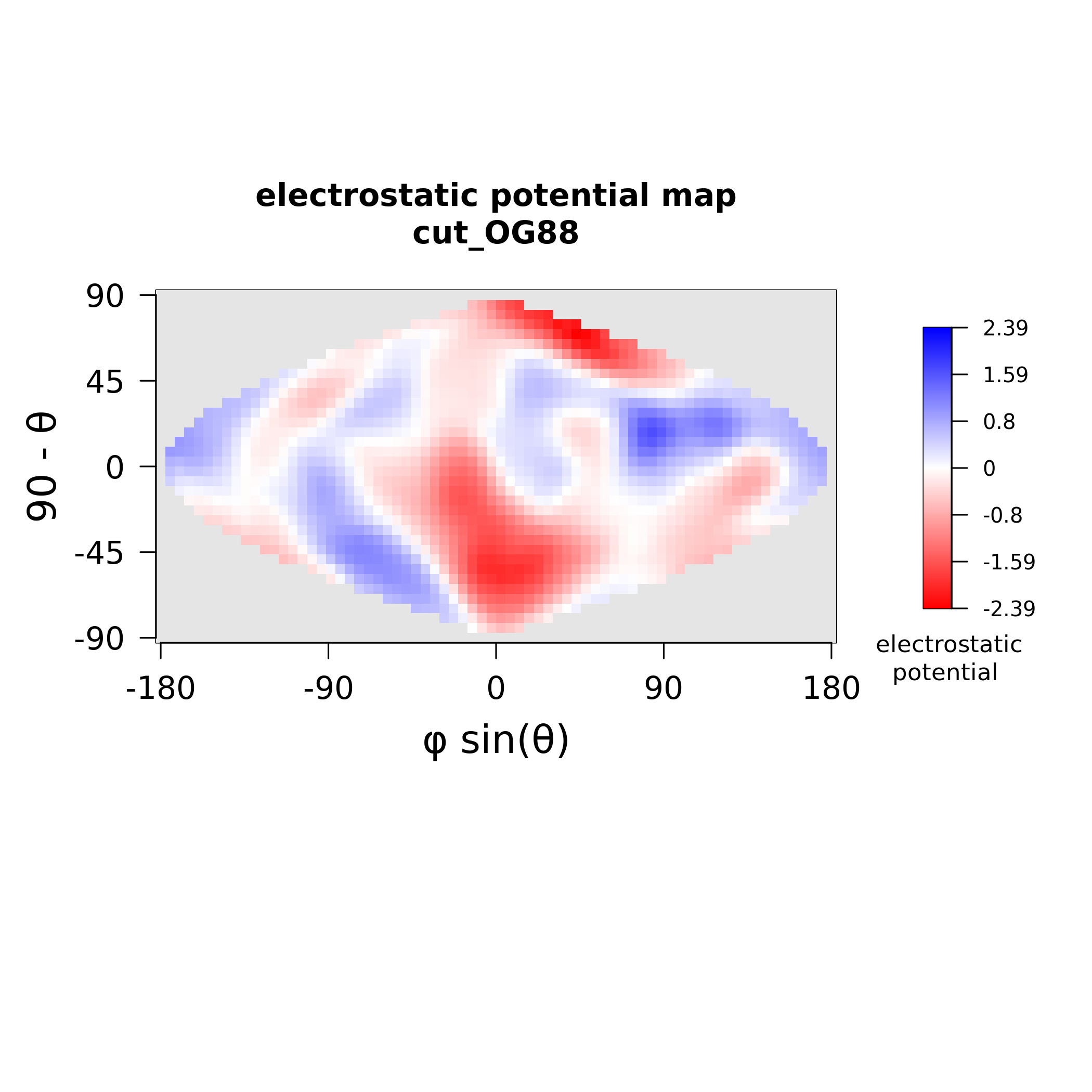

Supplement: S2 File — (ZIP) [file ppat.1012176.s019.zip › S2_File/ELECTROSTATICS/MAX88_electrostatics.png]

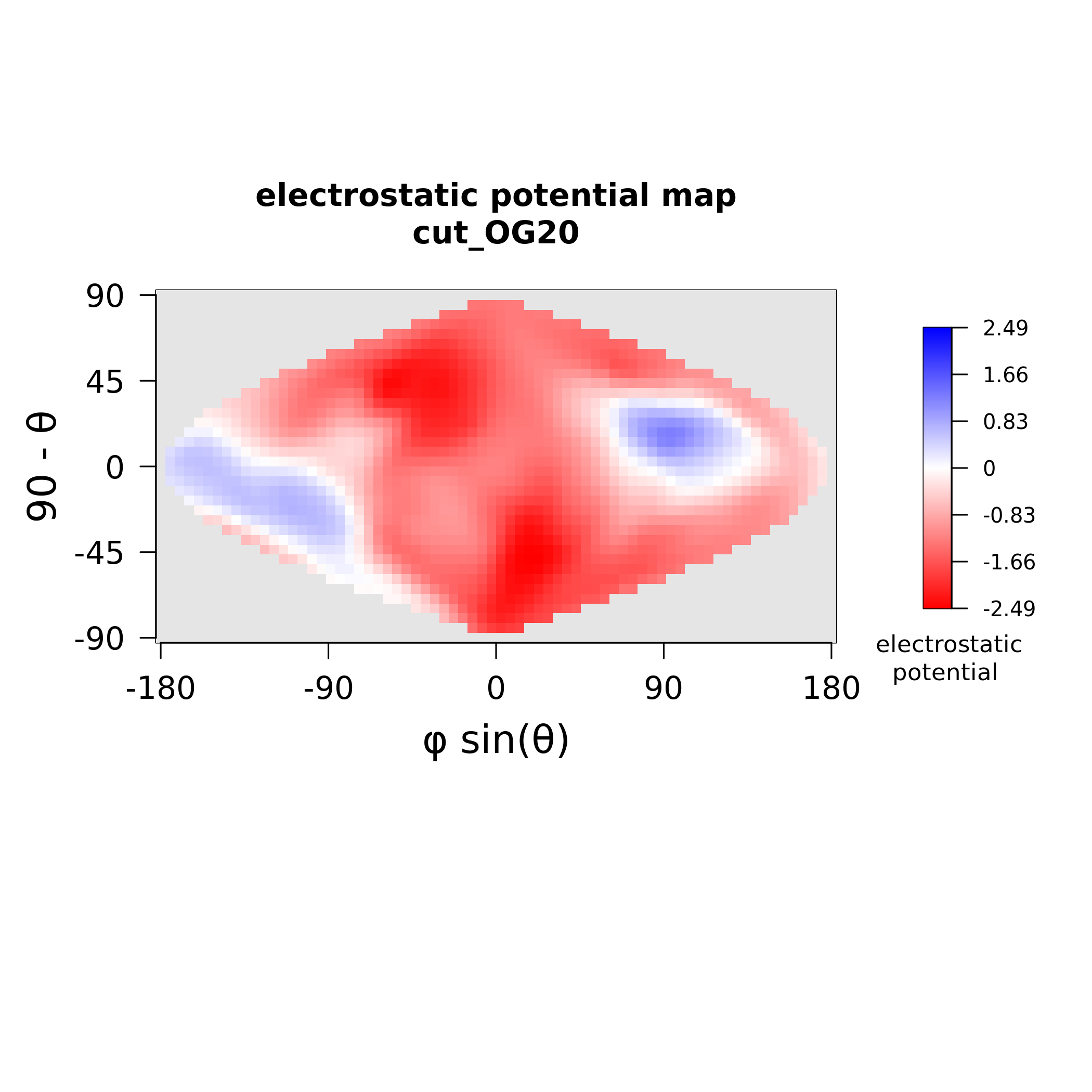

Supplement: S2 File — (ZIP) [file ppat.1012176.s019.zip › S2_File/ELECTROSTATICS/MAX20_electrostatics.png]

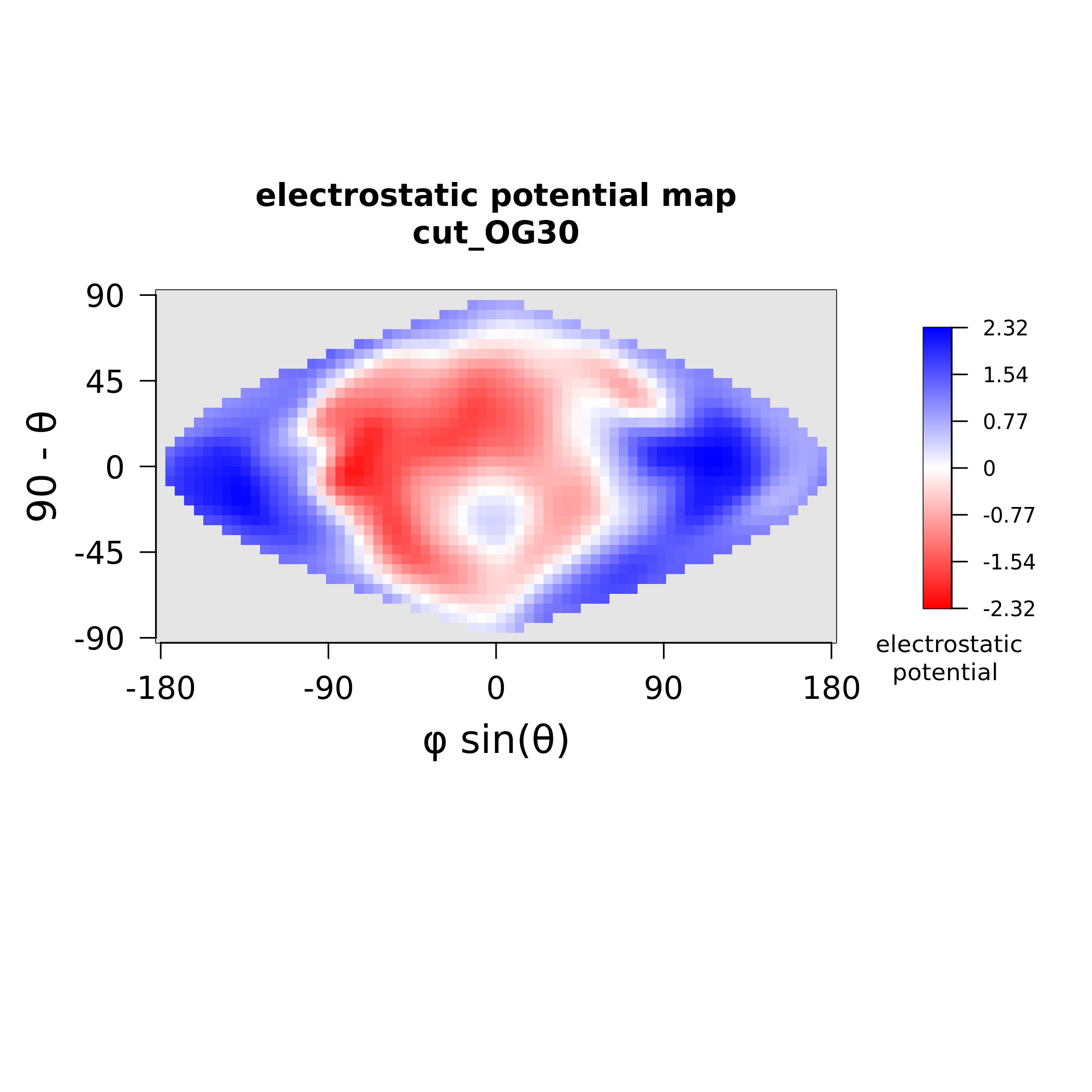

Supplement: S2 File — (ZIP) [file ppat.1012176.s019.zip › S2_File/ELECTROSTATICS/MAX30_electrostatics.png]

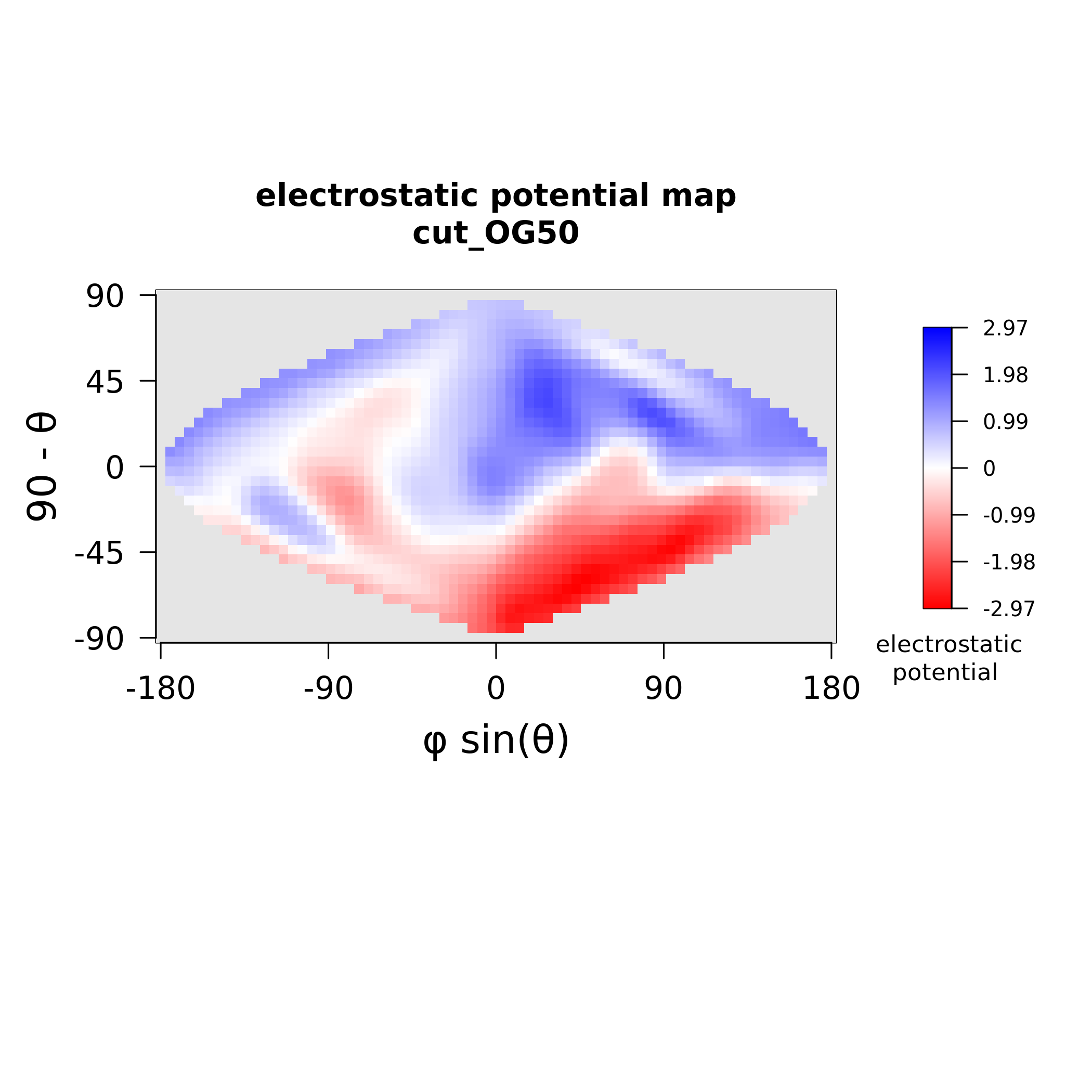

Supplement: S2 File — (ZIP) [file ppat.1012176.s019.zip › S2_File/ELECTROSTATICS/MAX50_electrostatics.png]

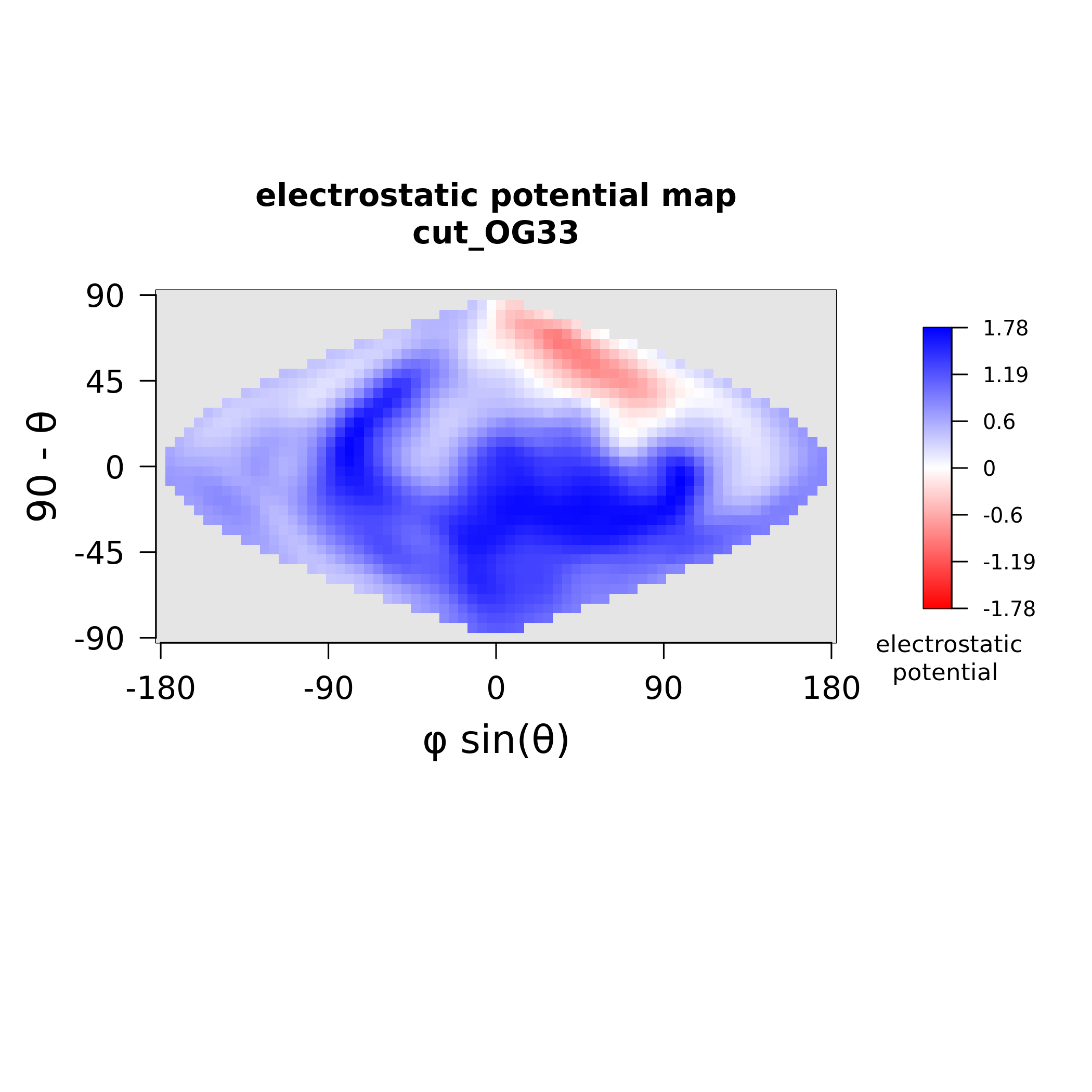

Supplement: S2 File — (ZIP) [file ppat.1012176.s019.zip › S2_File/ELECTROSTATICS/MAX33_electrostatics.png]

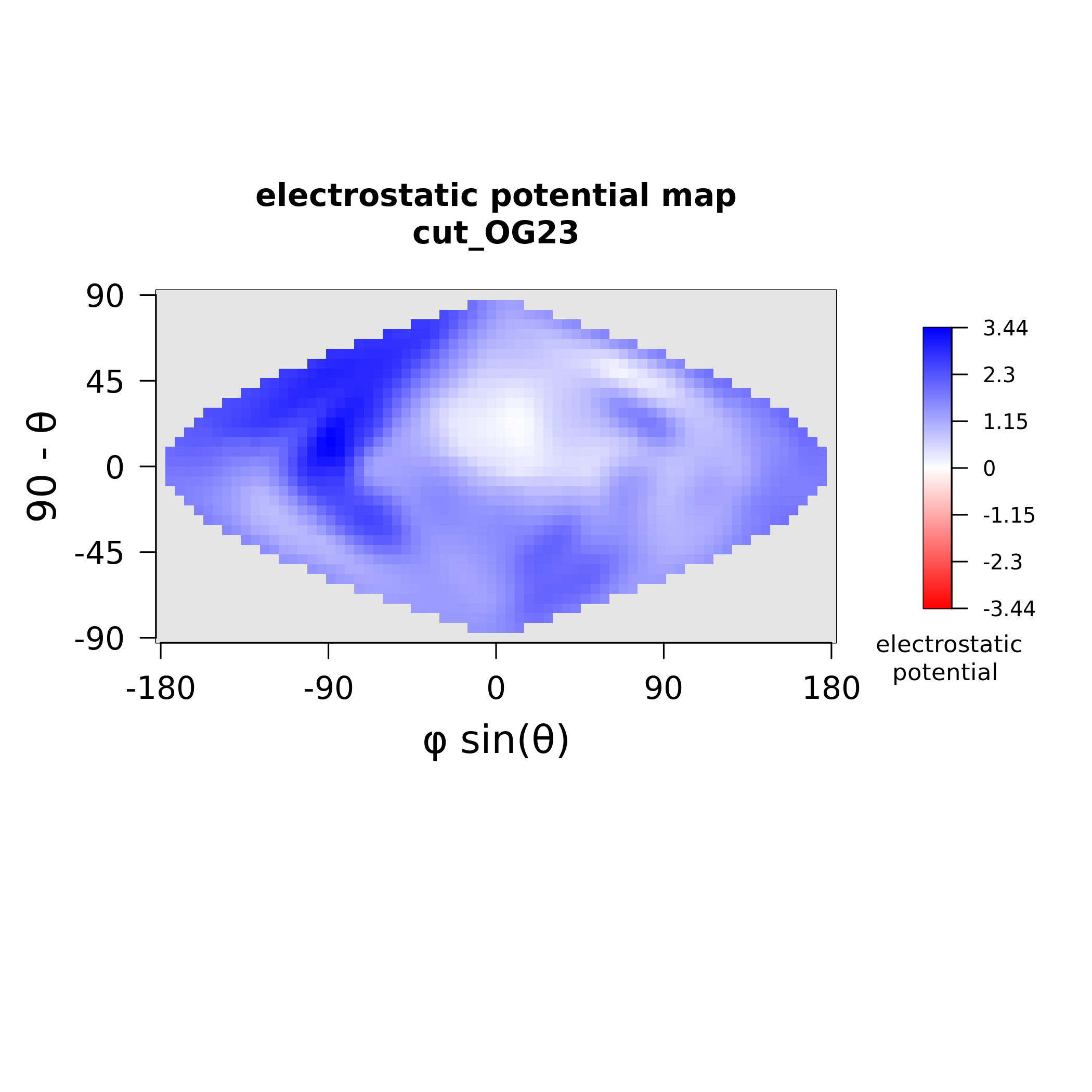

Supplement: S2 File — (ZIP) [file ppat.1012176.s019.zip › S2_File/ELECTROSTATICS/MAX23_electrostatics.png]

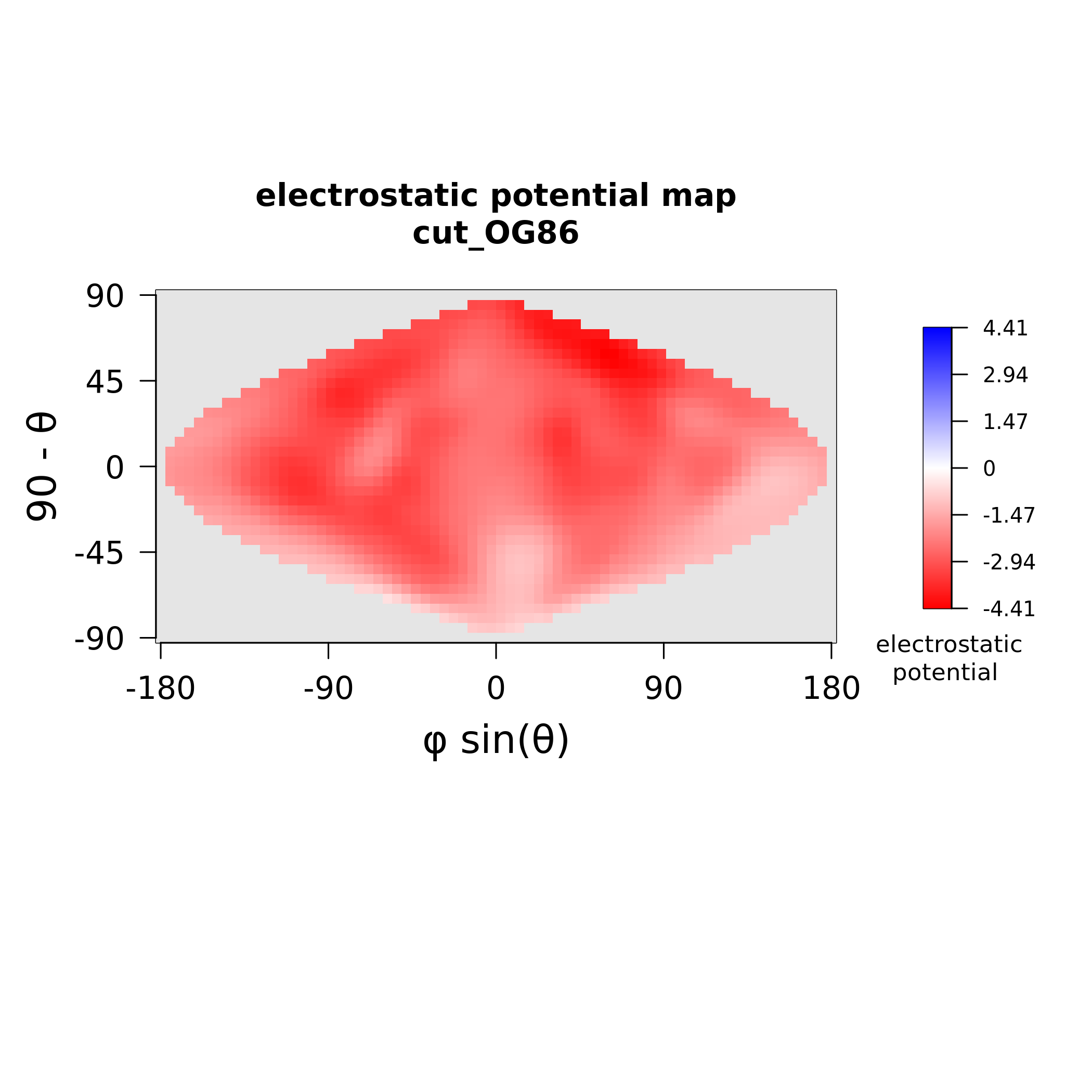

Supplement: S2 File — (ZIP) [file ppat.1012176.s019.zip › S2_File/ELECTROSTATICS/MAX86_electrostatics.png]

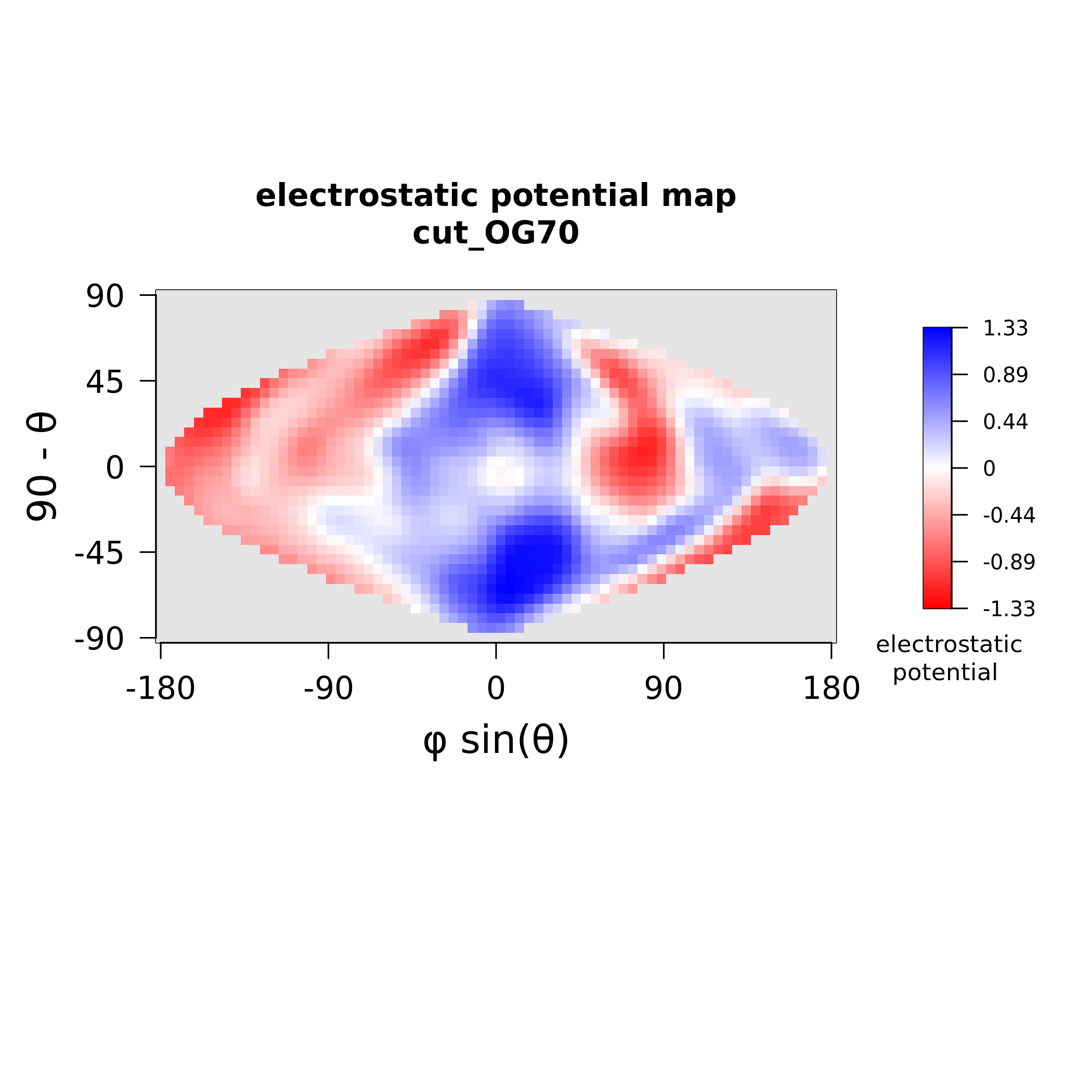

Supplement: S2 File — (ZIP) [file ppat.1012176.s019.zip › S2_File/ELECTROSTATICS/MAX70_electrostatics.png]

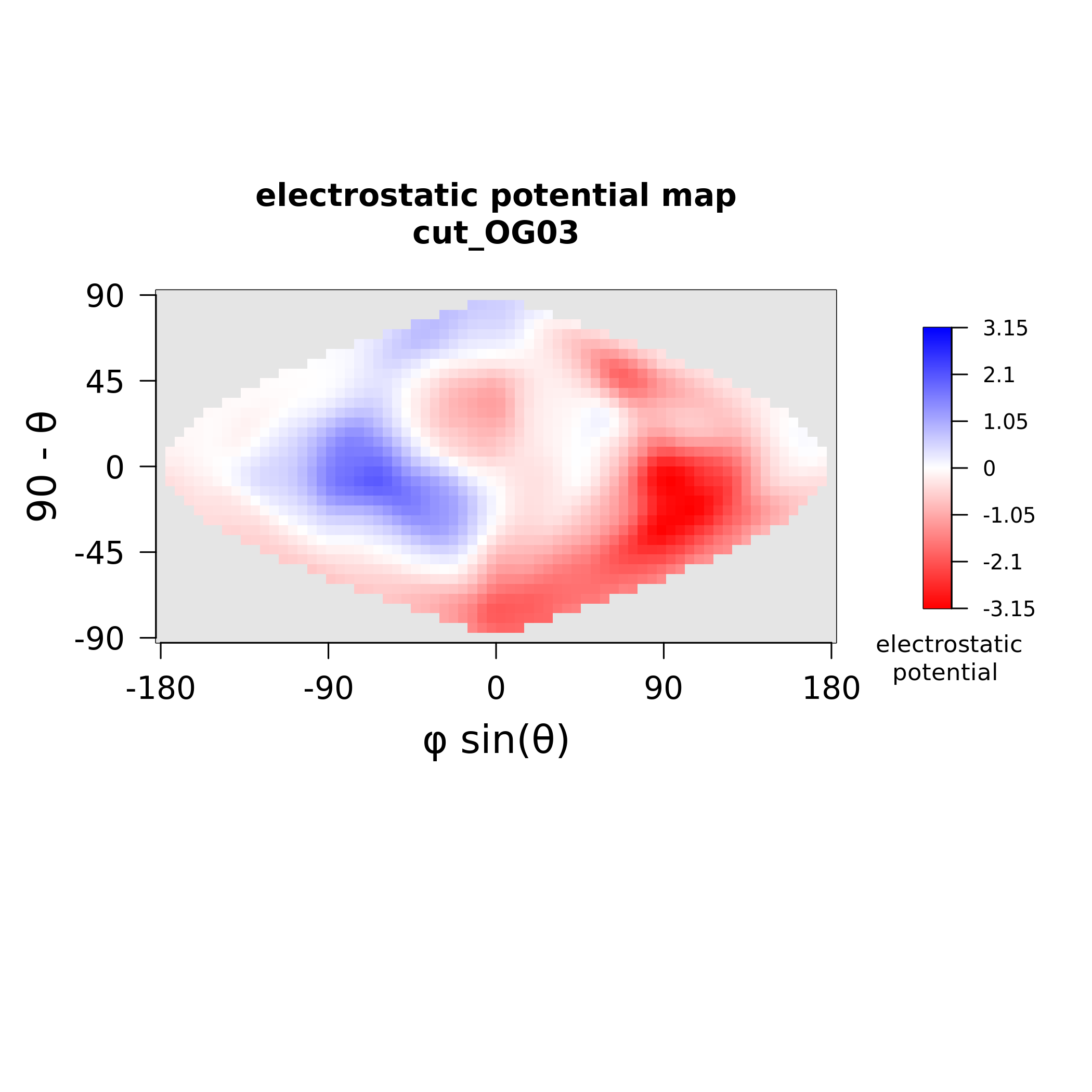

Supplement: S2 File — (ZIP) [file ppat.1012176.s019.zip › S2_File/ELECTROSTATICS/MAX03_electrostatics.png]

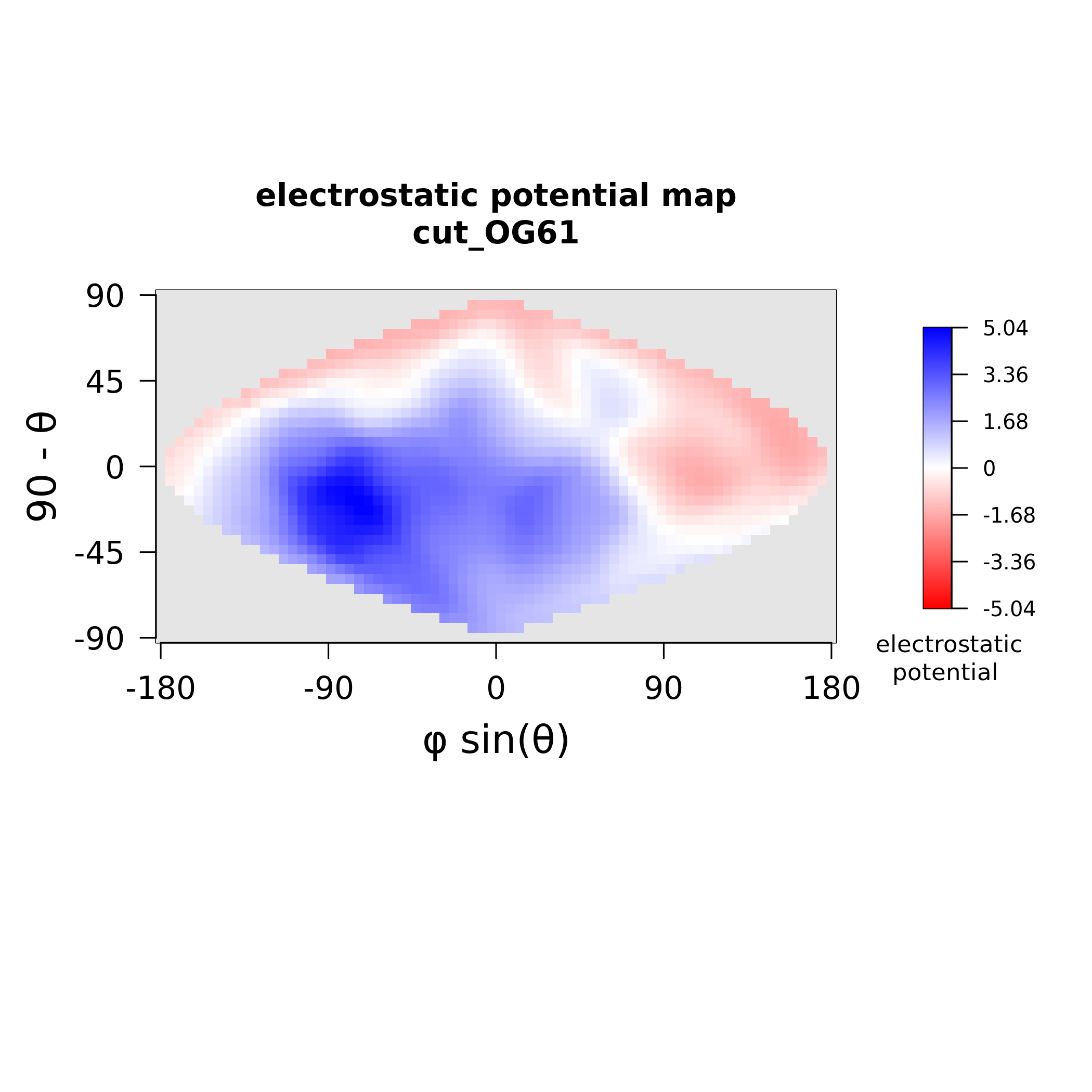

Supplement: S2 File — (ZIP) [file ppat.1012176.s019.zip › S2_File/ELECTROSTATICS/MAX61_electrostatics.png]

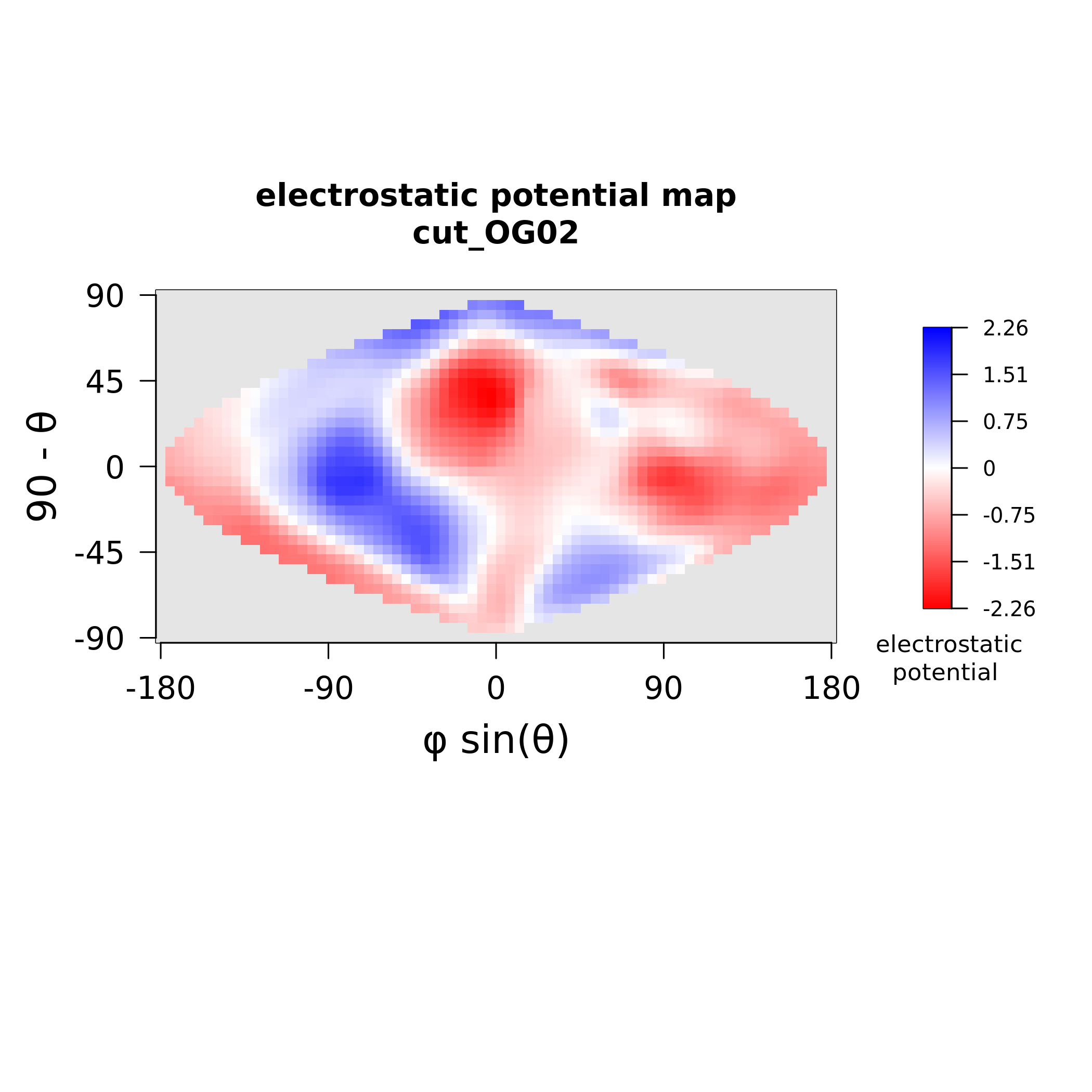

Supplement: S2 File — (ZIP) [file ppat.1012176.s019.zip › S2_File/ELECTROSTATICS/MAX02_electrostatics.png]

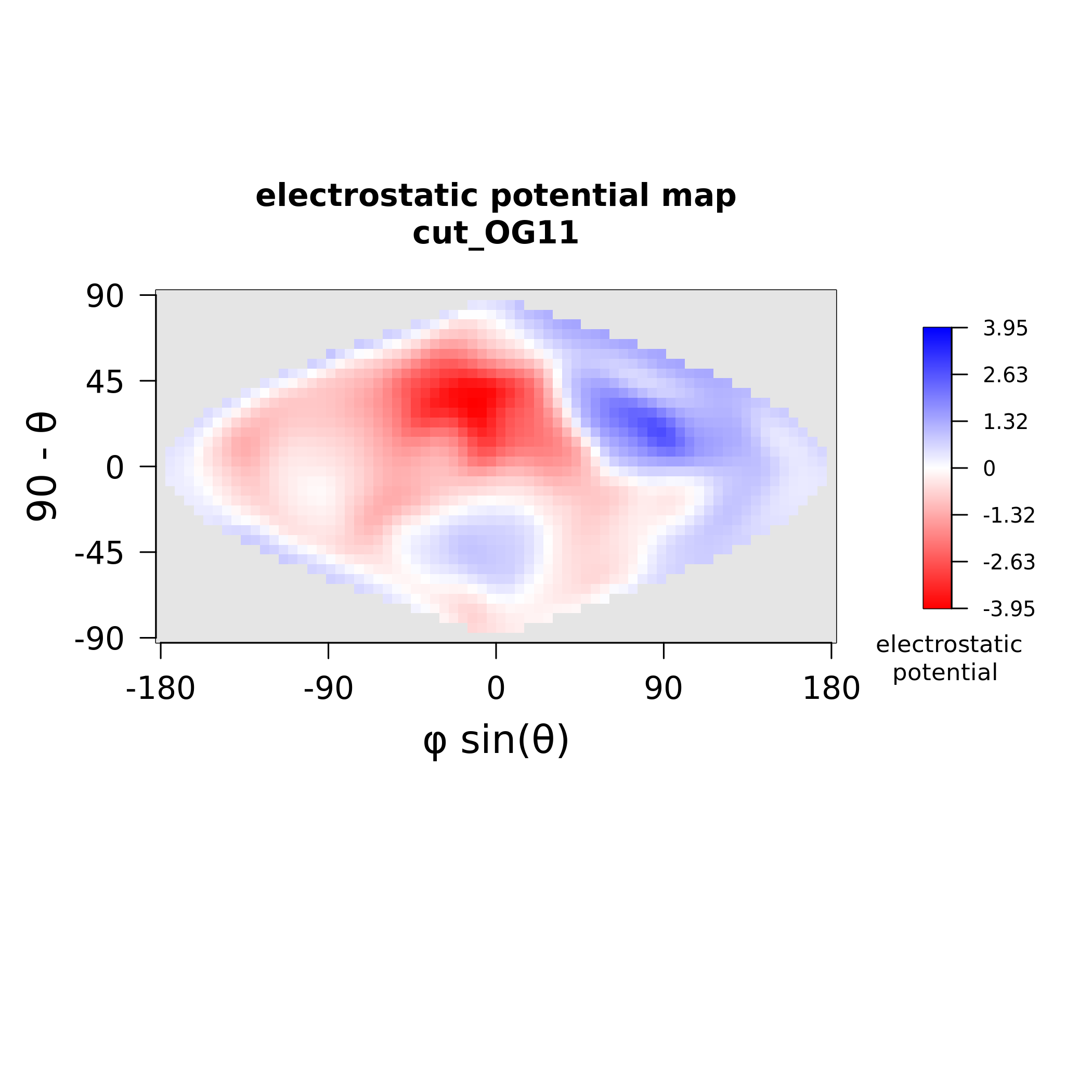

Supplement: S2 File — (ZIP) [file ppat.1012176.s019.zip › S2_File/ELECTROSTATICS/MAX11_electrostatics.png]

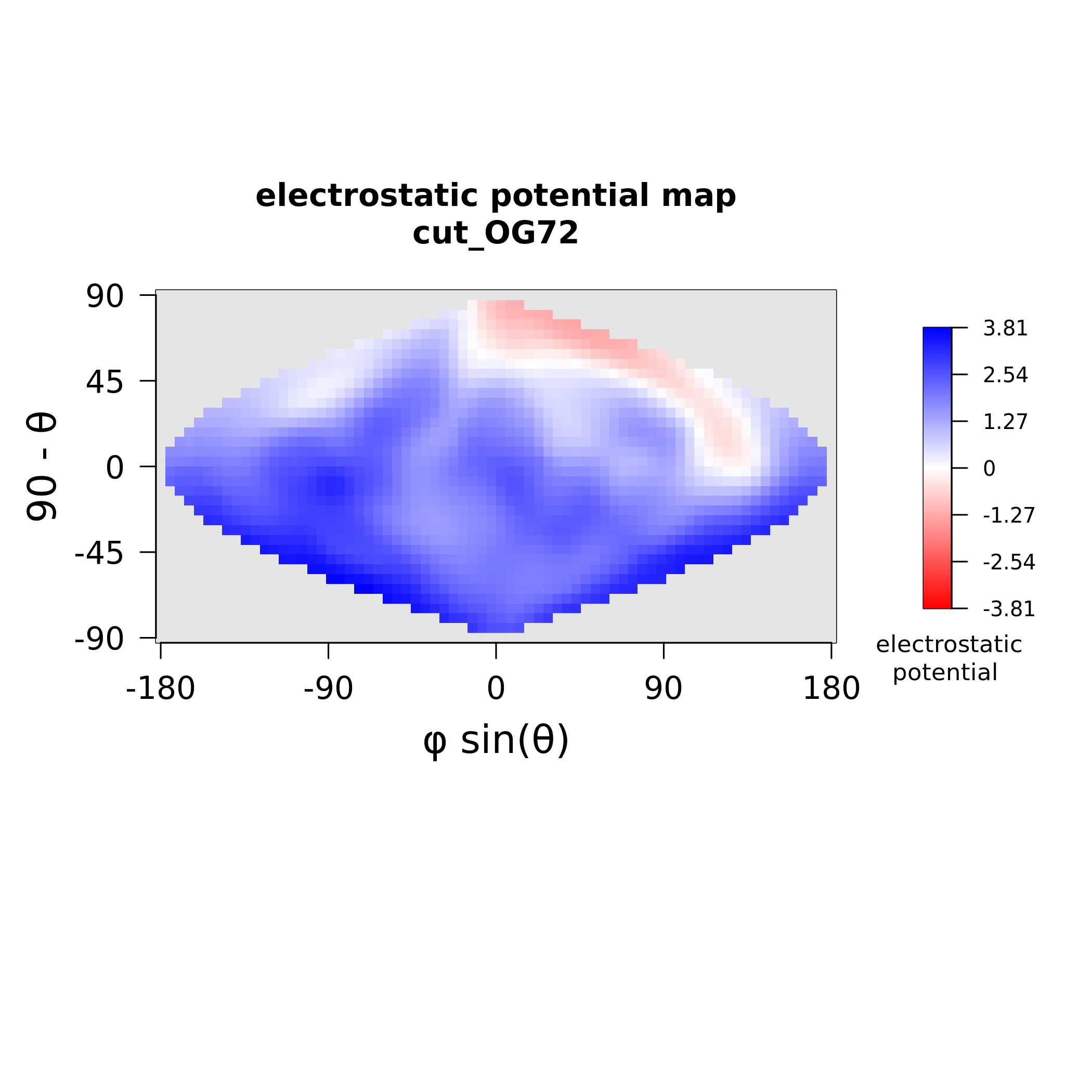

Supplement: S2 File — (ZIP) [file ppat.1012176.s019.zip › S2_File/ELECTROSTATICS/MAX72_electrostatics.png]

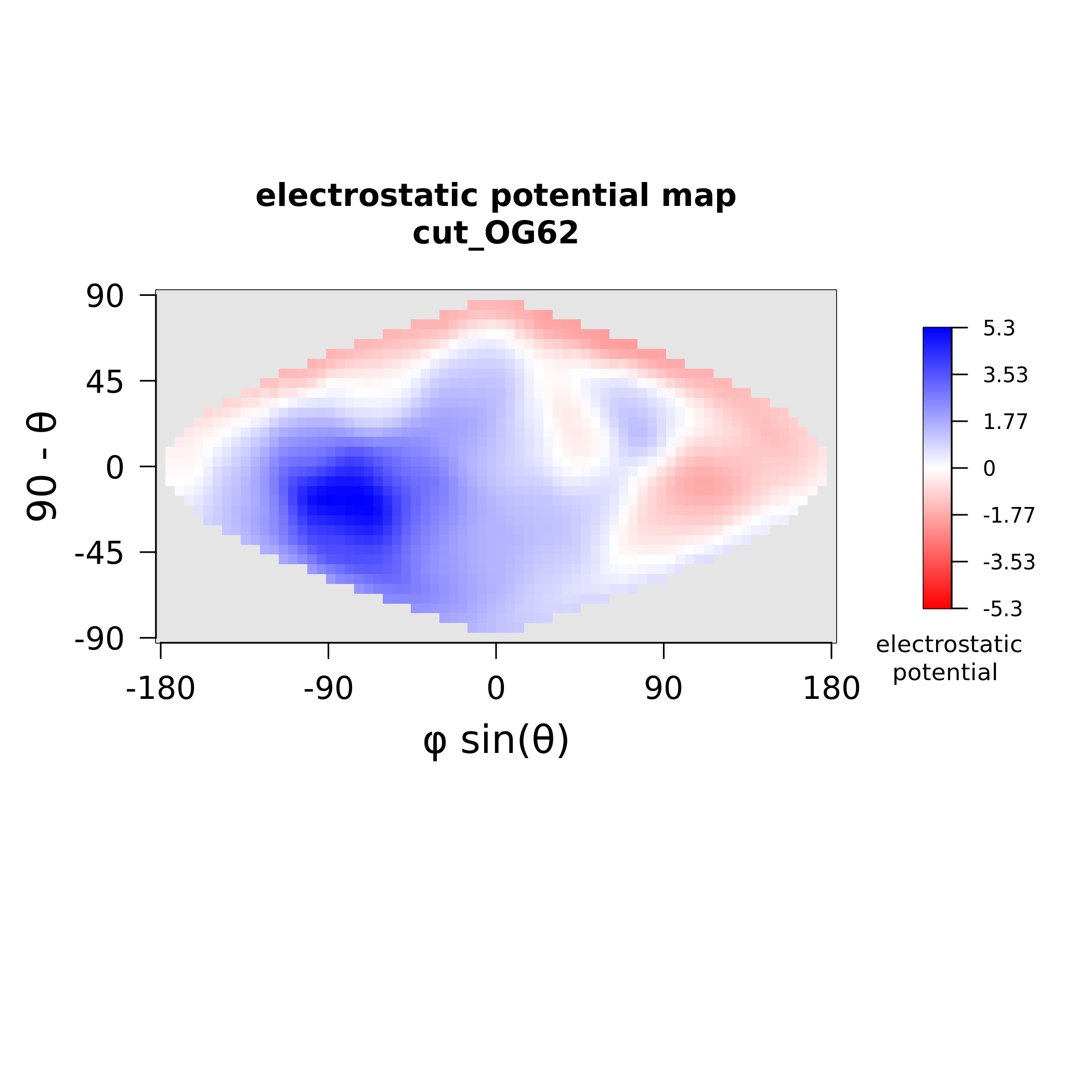

Supplement: S2 File — (ZIP) [file ppat.1012176.s019.zip › S2_File/ELECTROSTATICS/MAX62_electrostatics.png]

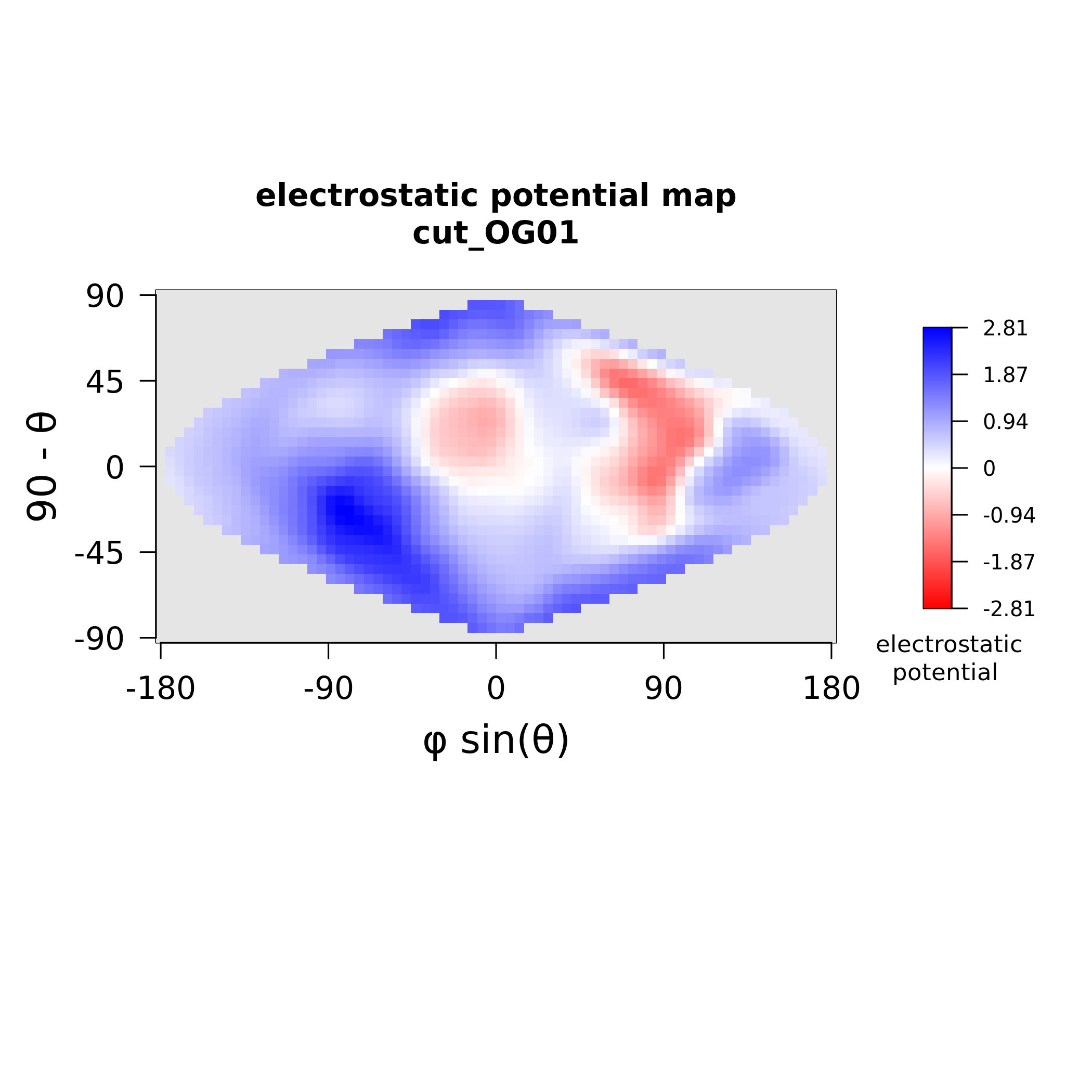

Supplement: S2 File — (ZIP) [file ppat.1012176.s019.zip › S2_File/ELECTROSTATICS/MAX01_electrostatics.png]

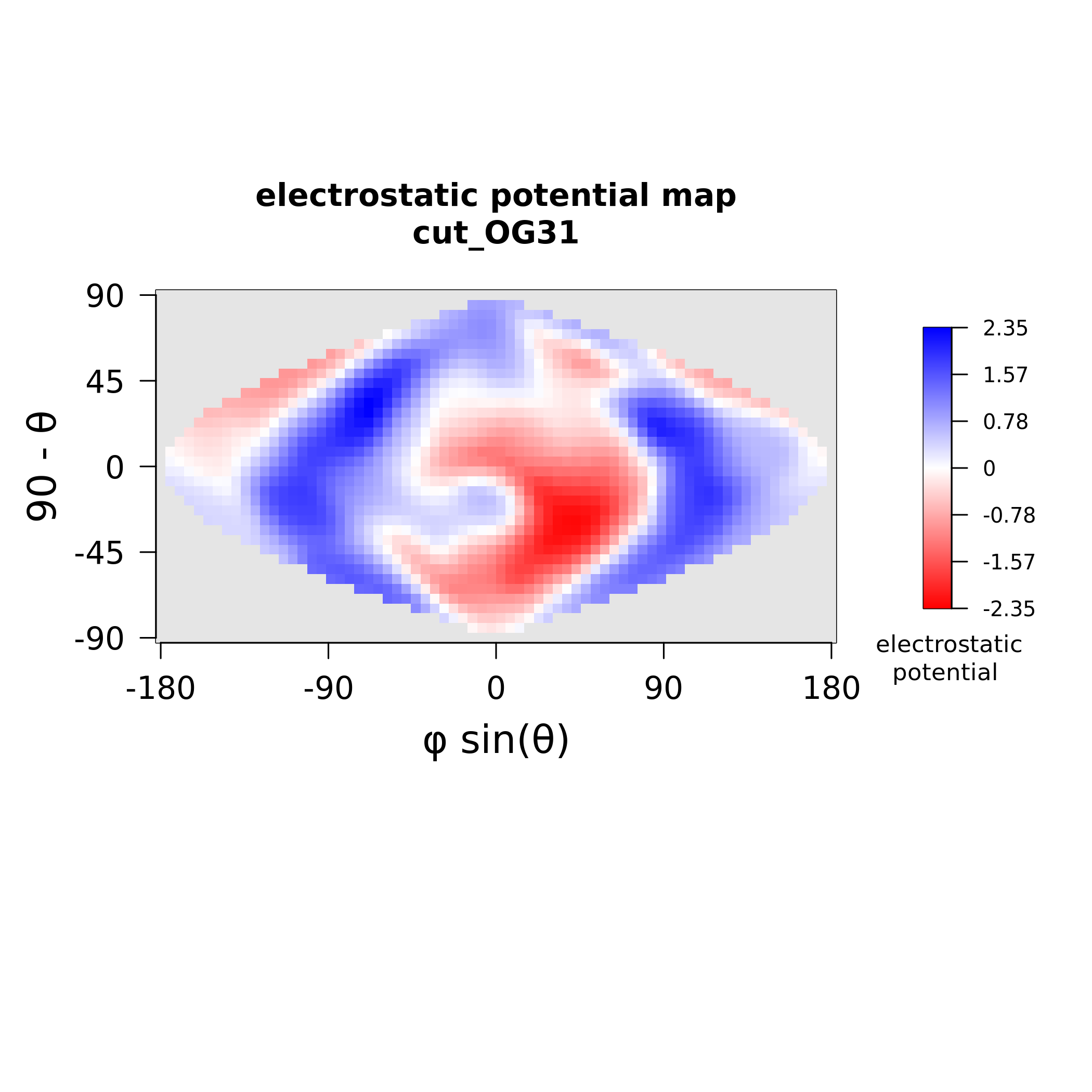

Supplement: S2 File — (ZIP) [file ppat.1012176.s019.zip › S2_File/ELECTROSTATICS/MAX31_electrostatics.png]

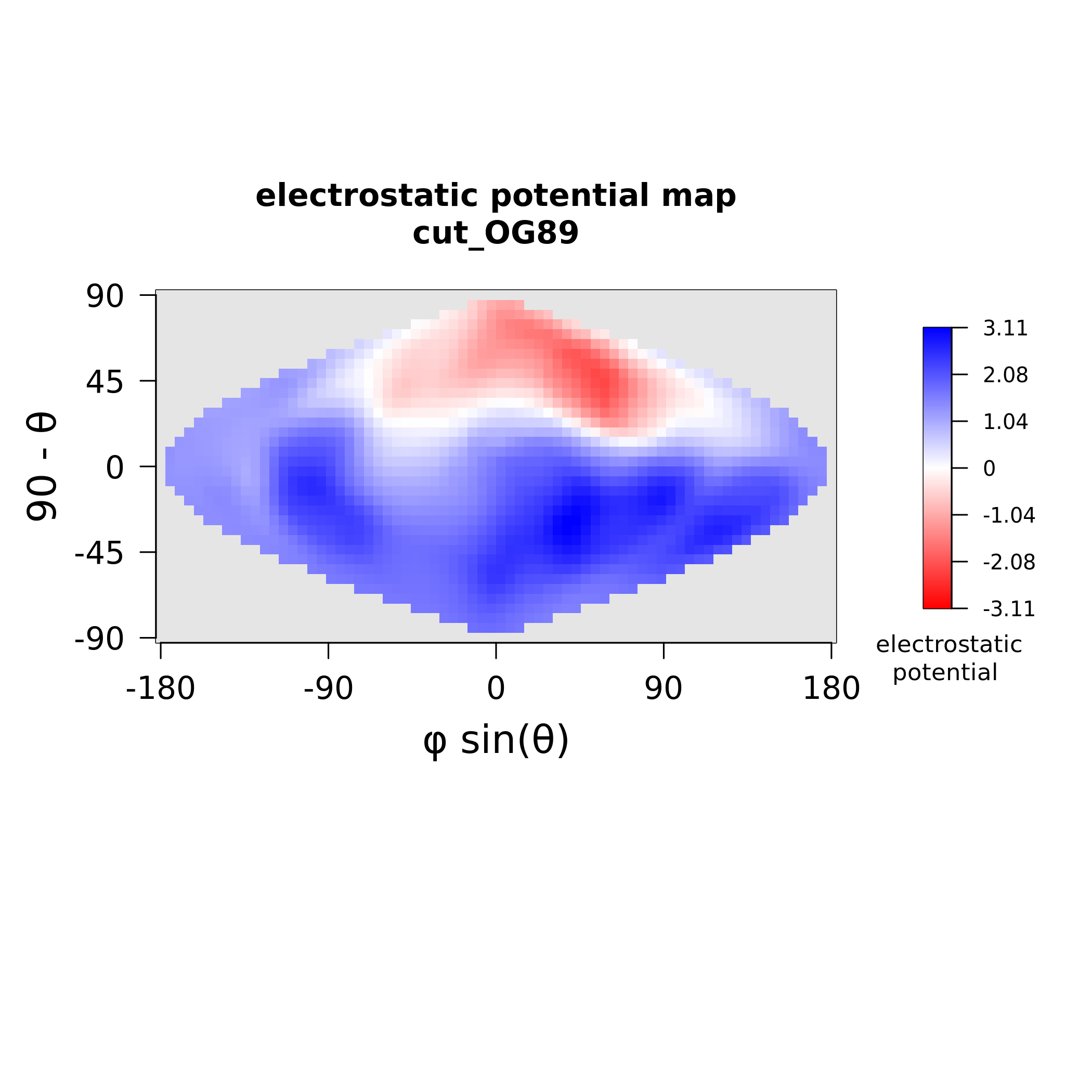

Supplement: S2 File — (ZIP) [file ppat.1012176.s019.zip › S2_File/ELECTROSTATICS/MAX89_electrostatics.png]

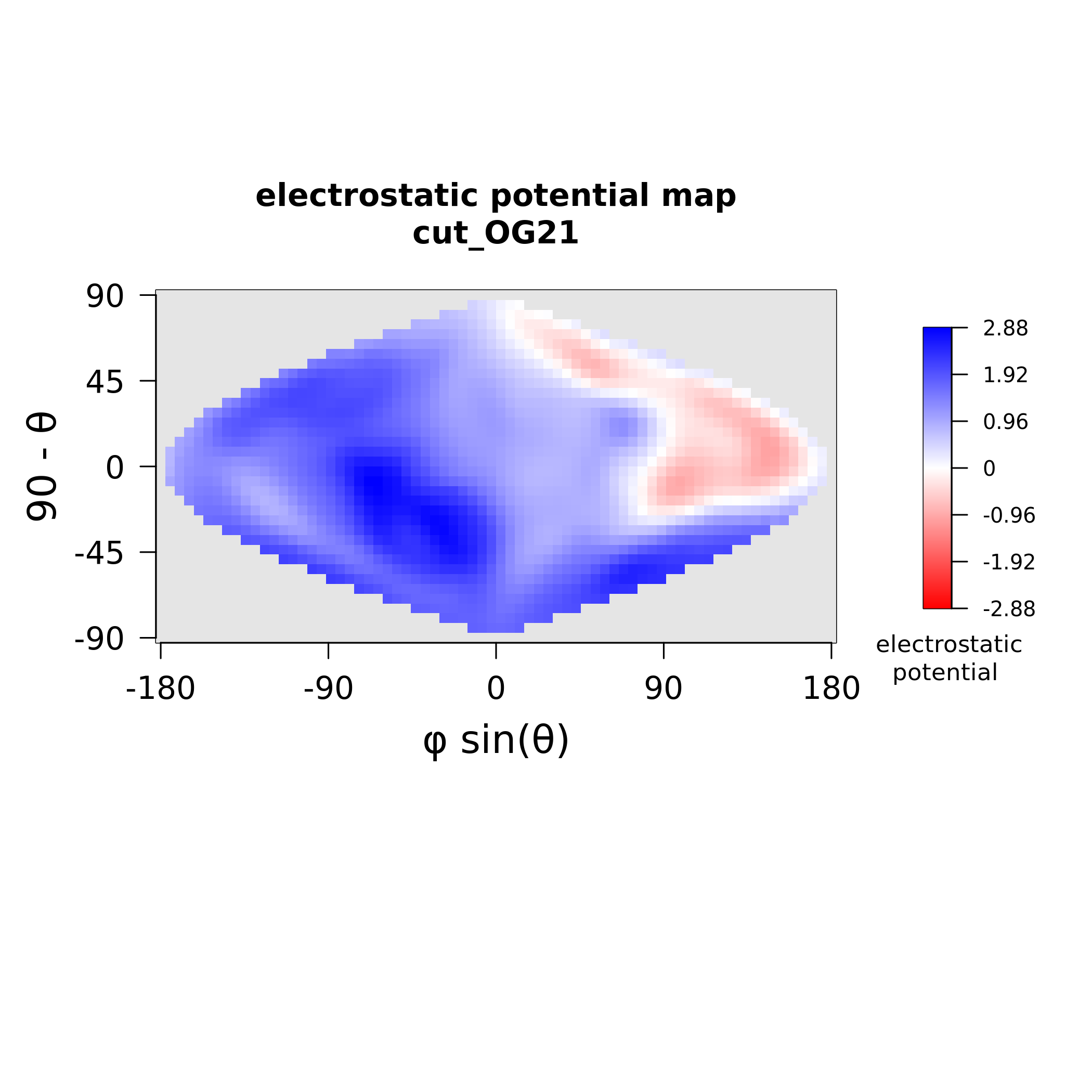

Supplement: S2 File — (ZIP) [file ppat.1012176.s019.zip › S2_File/ELECTROSTATICS/MAX21_electrostatics.png]

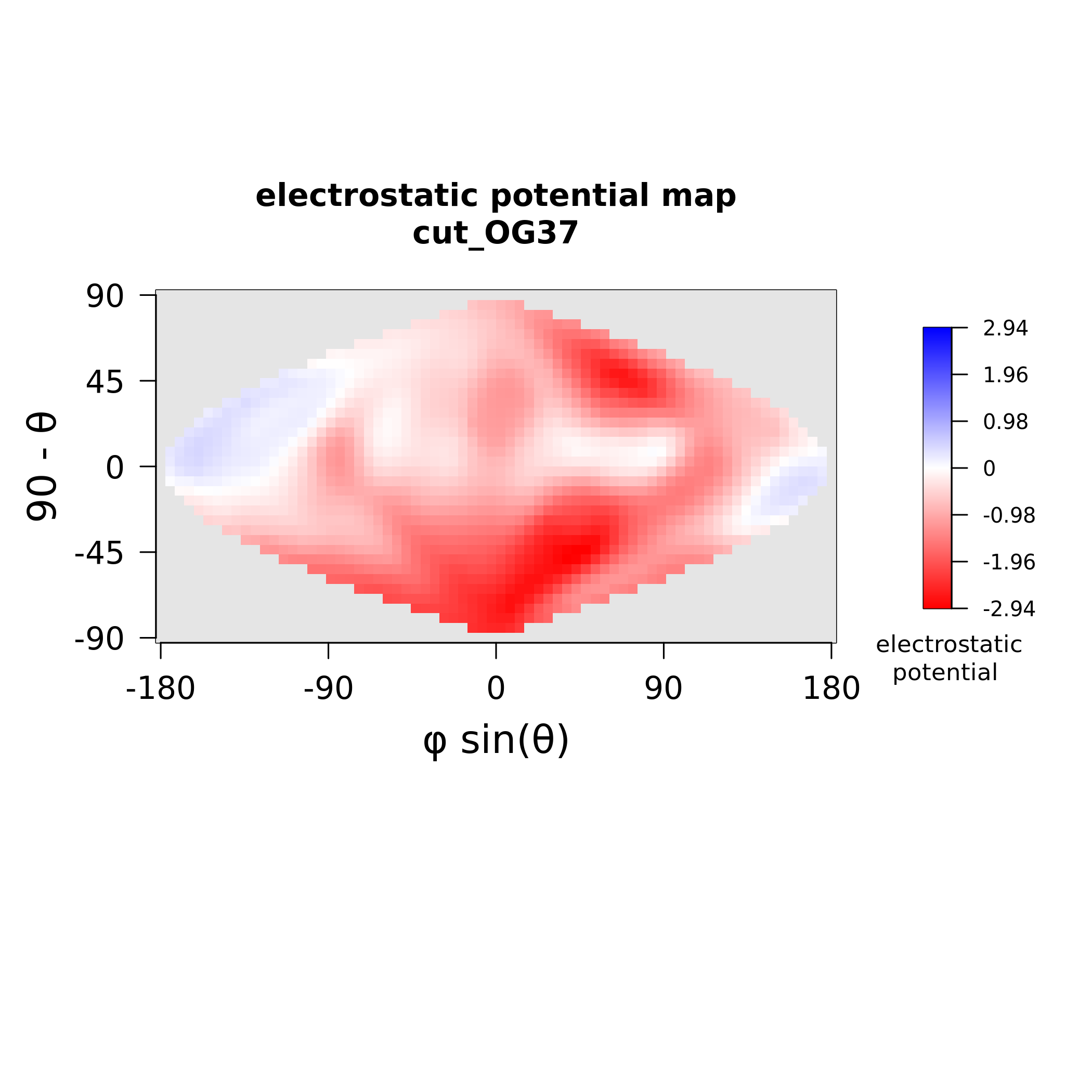

Supplement: S2 File — (ZIP) [file ppat.1012176.s019.zip › S2_File/ELECTROSTATICS/MAX37_electrostatics.png]

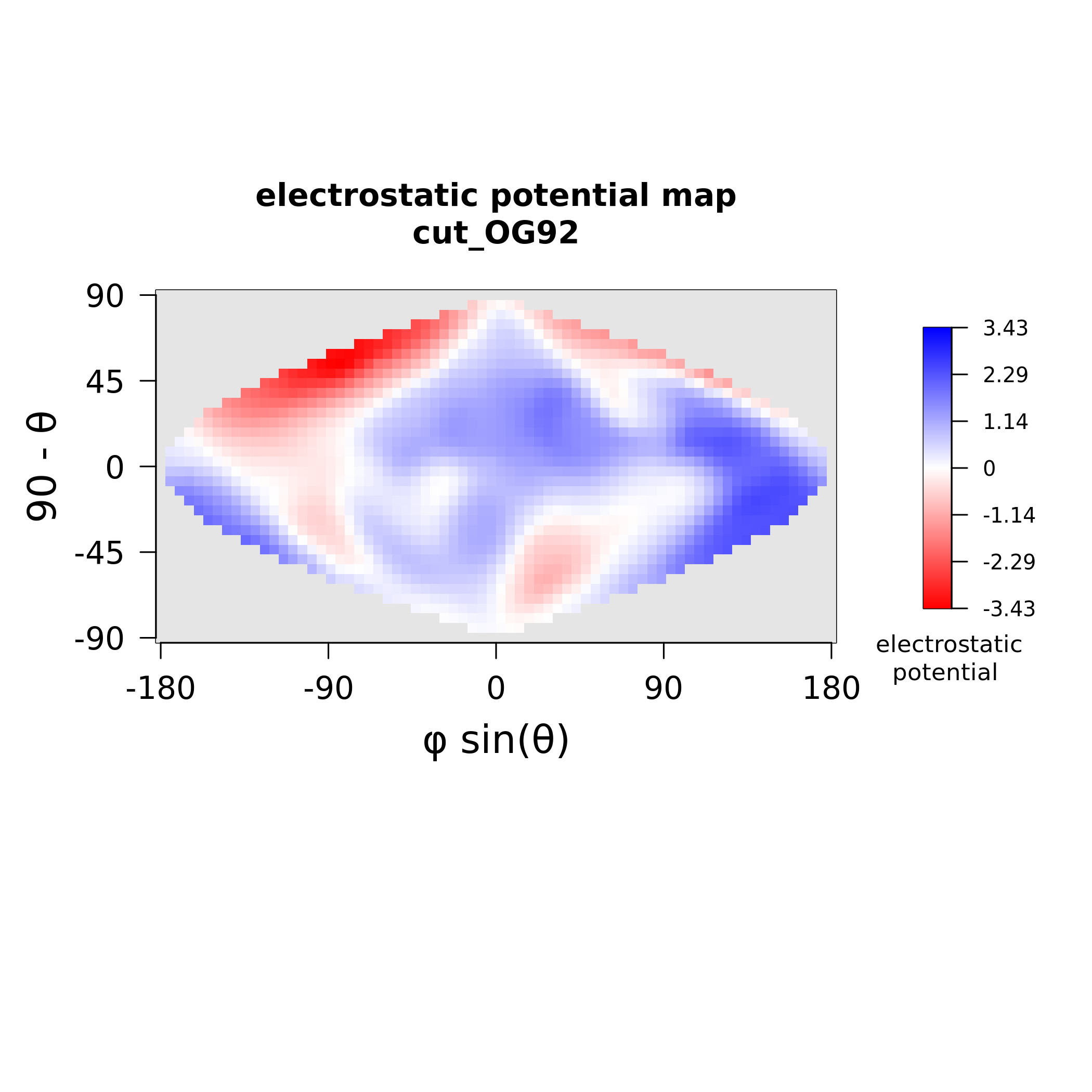

Supplement: S2 File — (ZIP) [file ppat.1012176.s019.zip › S2_File/ELECTROSTATICS/MAX92_electrostatics.png]

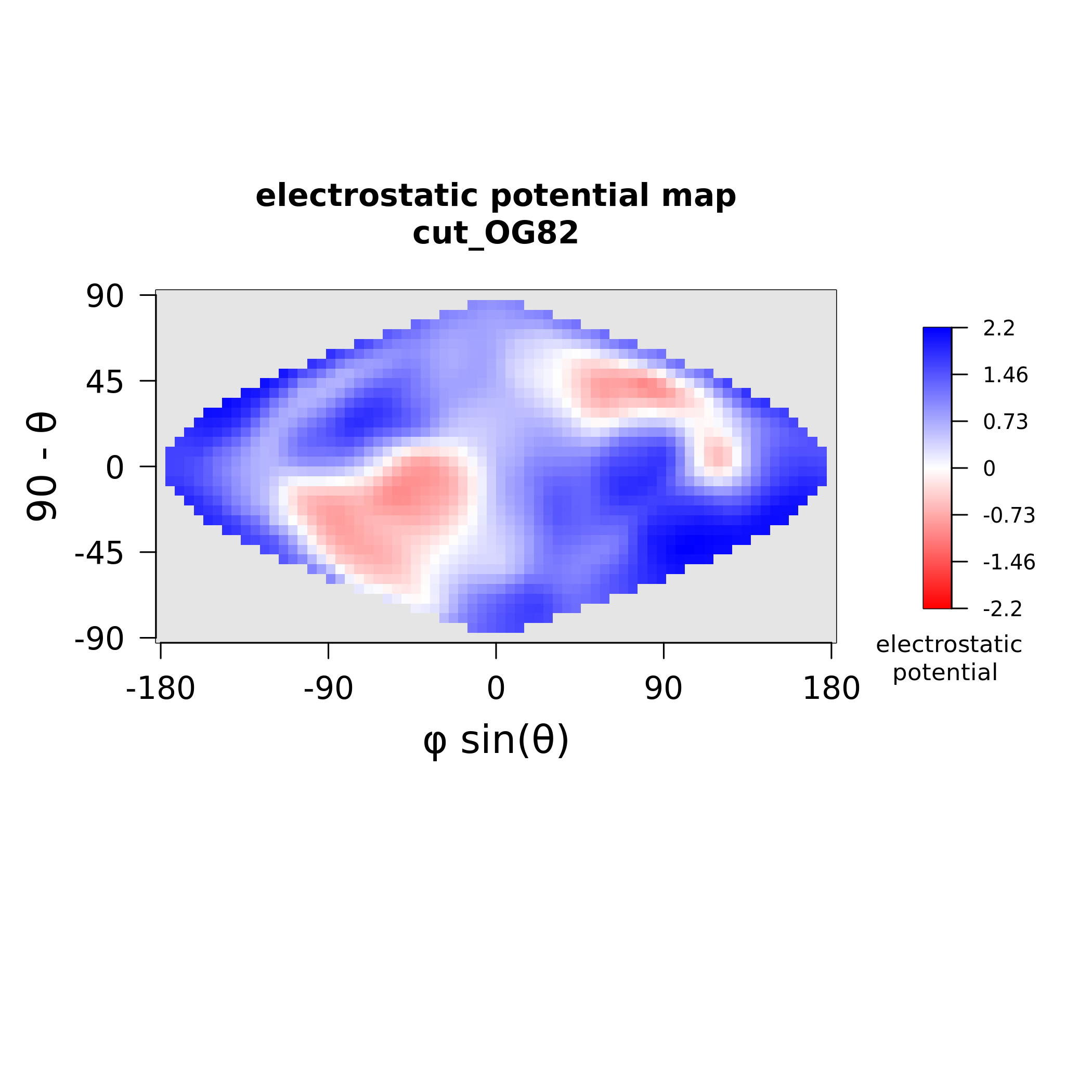

Supplement: S2 File — (ZIP) [file ppat.1012176.s019.zip › S2_File/ELECTROSTATICS/MAX82_electrostatics.png]

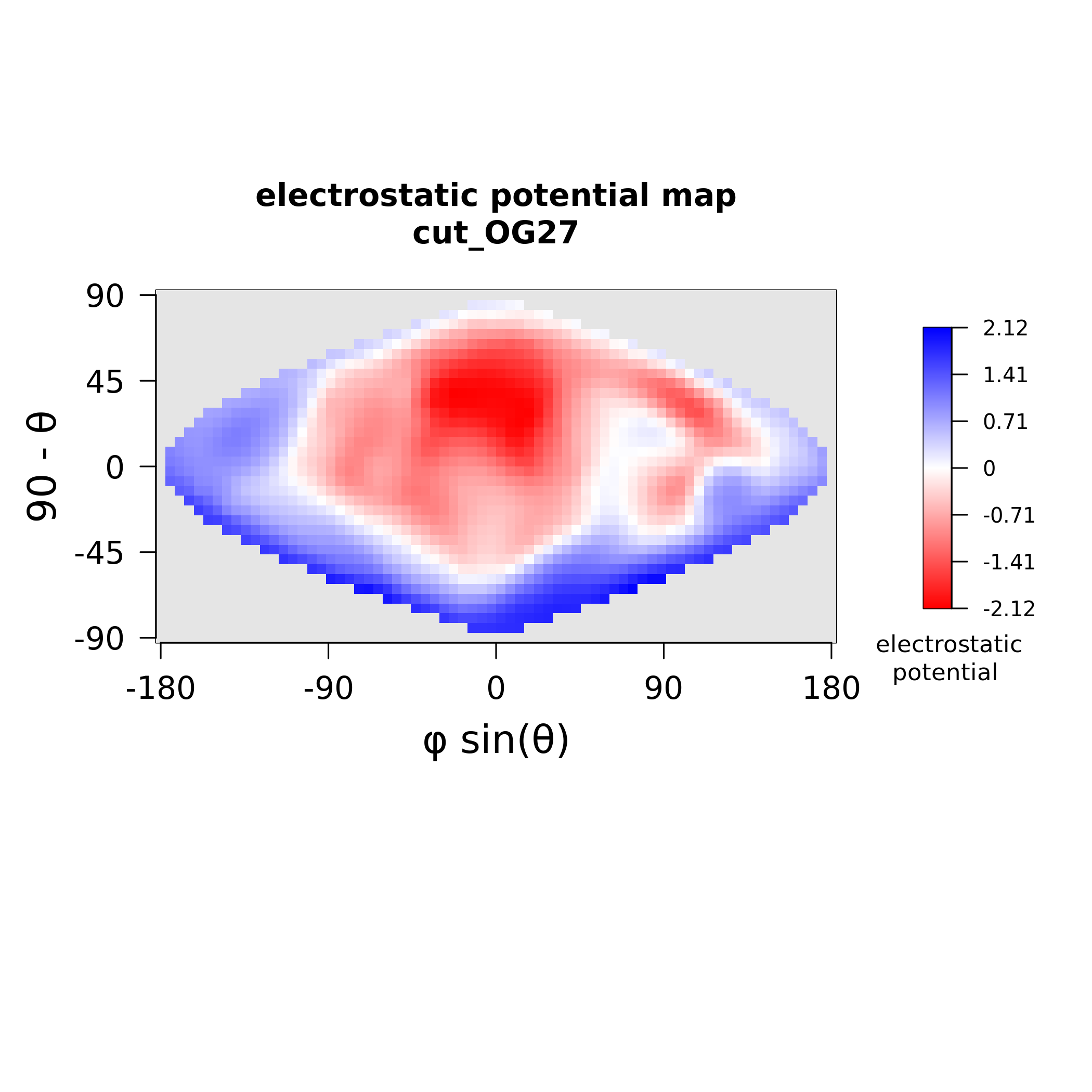

Supplement: S2 File — (ZIP) [file ppat.1012176.s019.zip › S2_File/ELECTROSTATICS/MAX27_electrostatics.png]

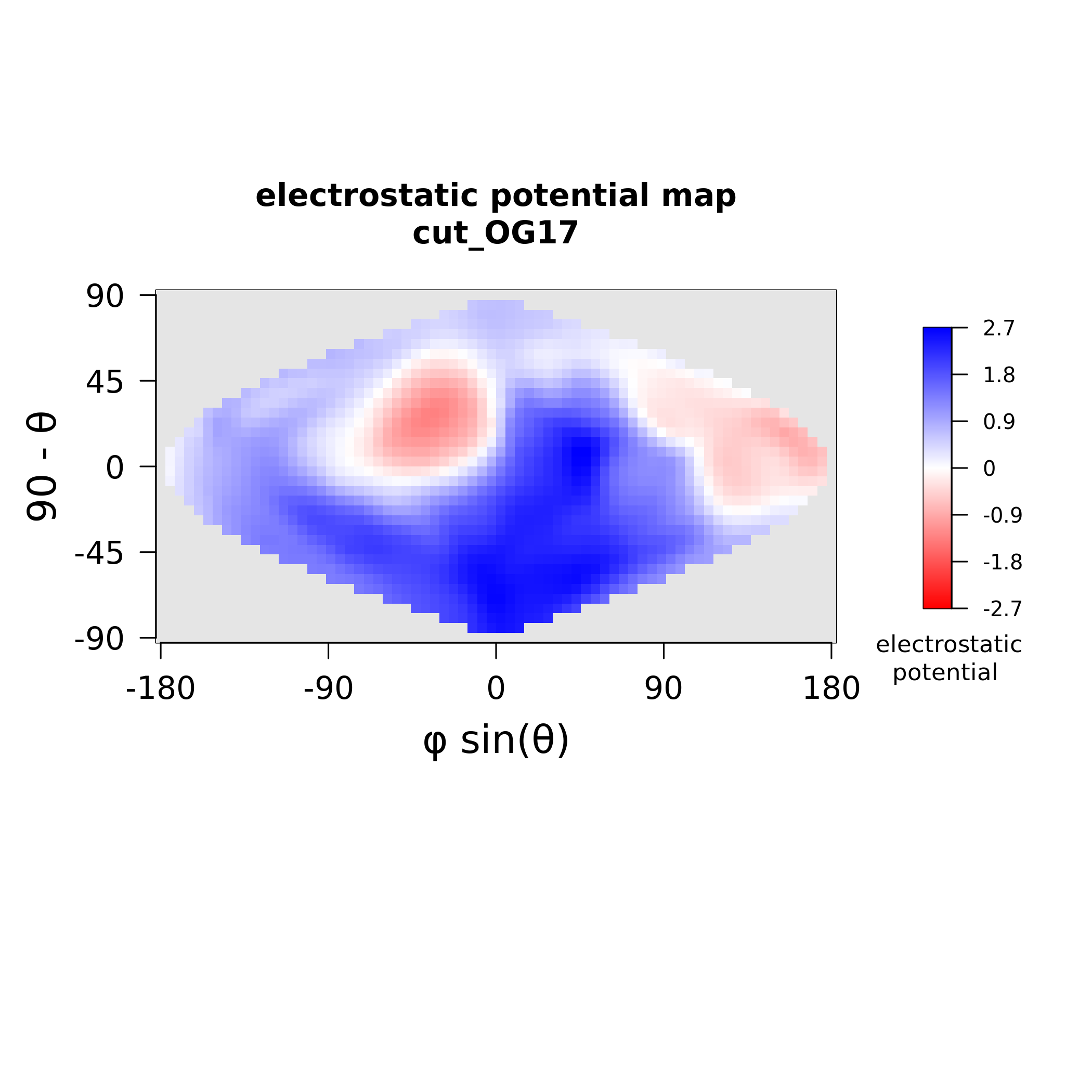

Supplement: S2 File — (ZIP) [file ppat.1012176.s019.zip › S2_File/ELECTROSTATICS/MAX17_electrostatics.png]

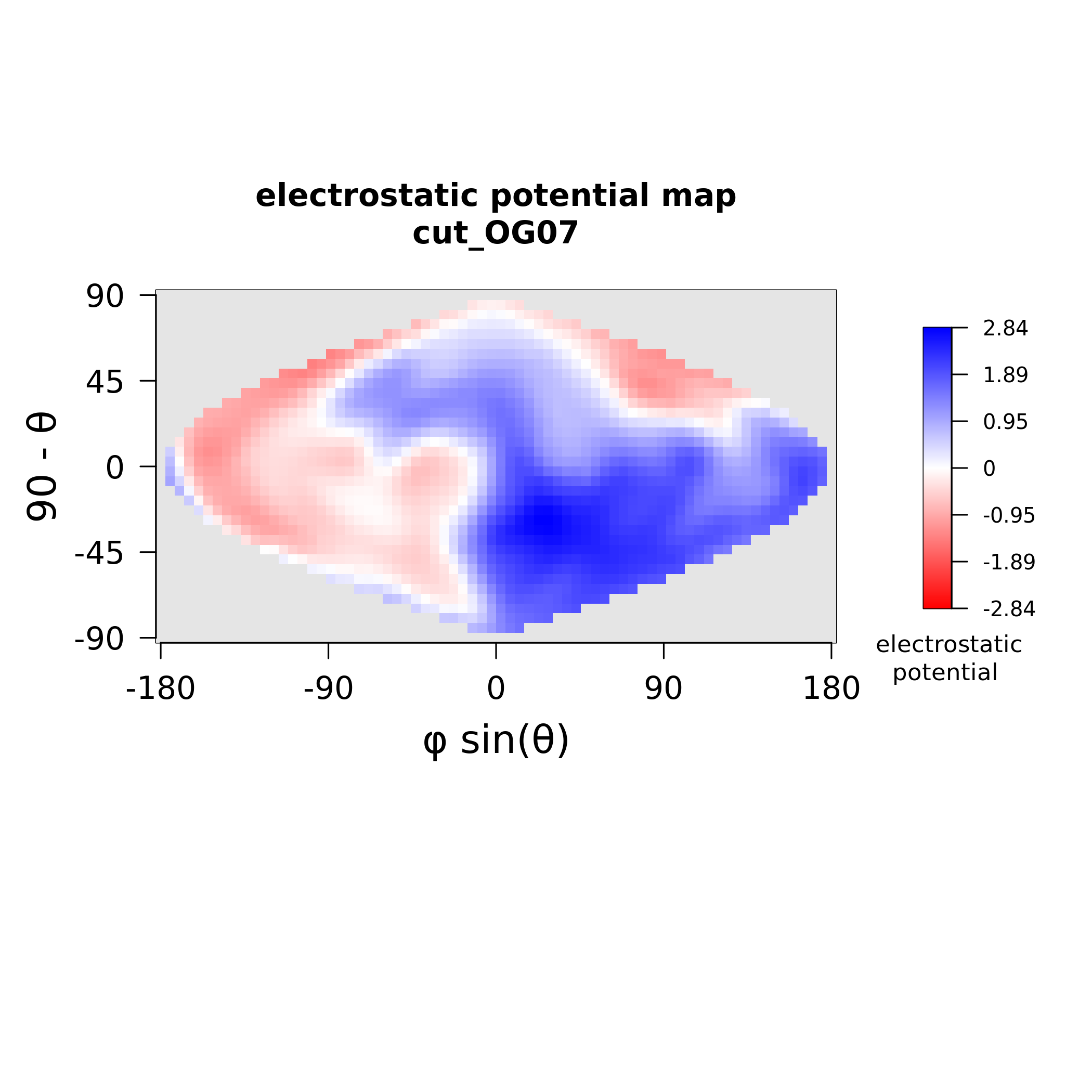

Supplement: S2 File — (ZIP) [file ppat.1012176.s019.zip › S2_File/ELECTROSTATICS/MAX07_electrostatics.png]

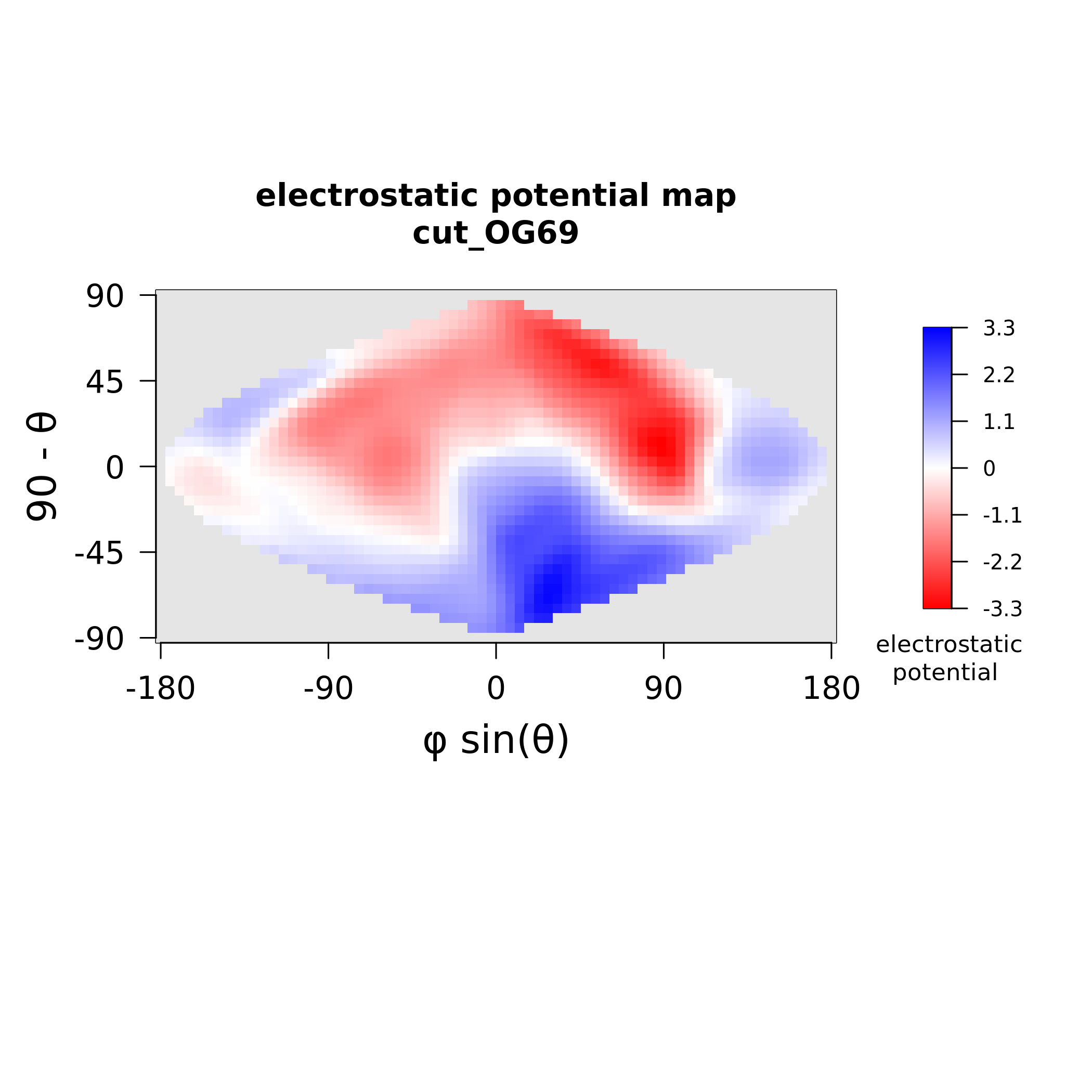

Supplement: S2 File — (ZIP) [file ppat.1012176.s019.zip › S2_File/ELECTROSTATICS/MAX69_electrostatics.png]

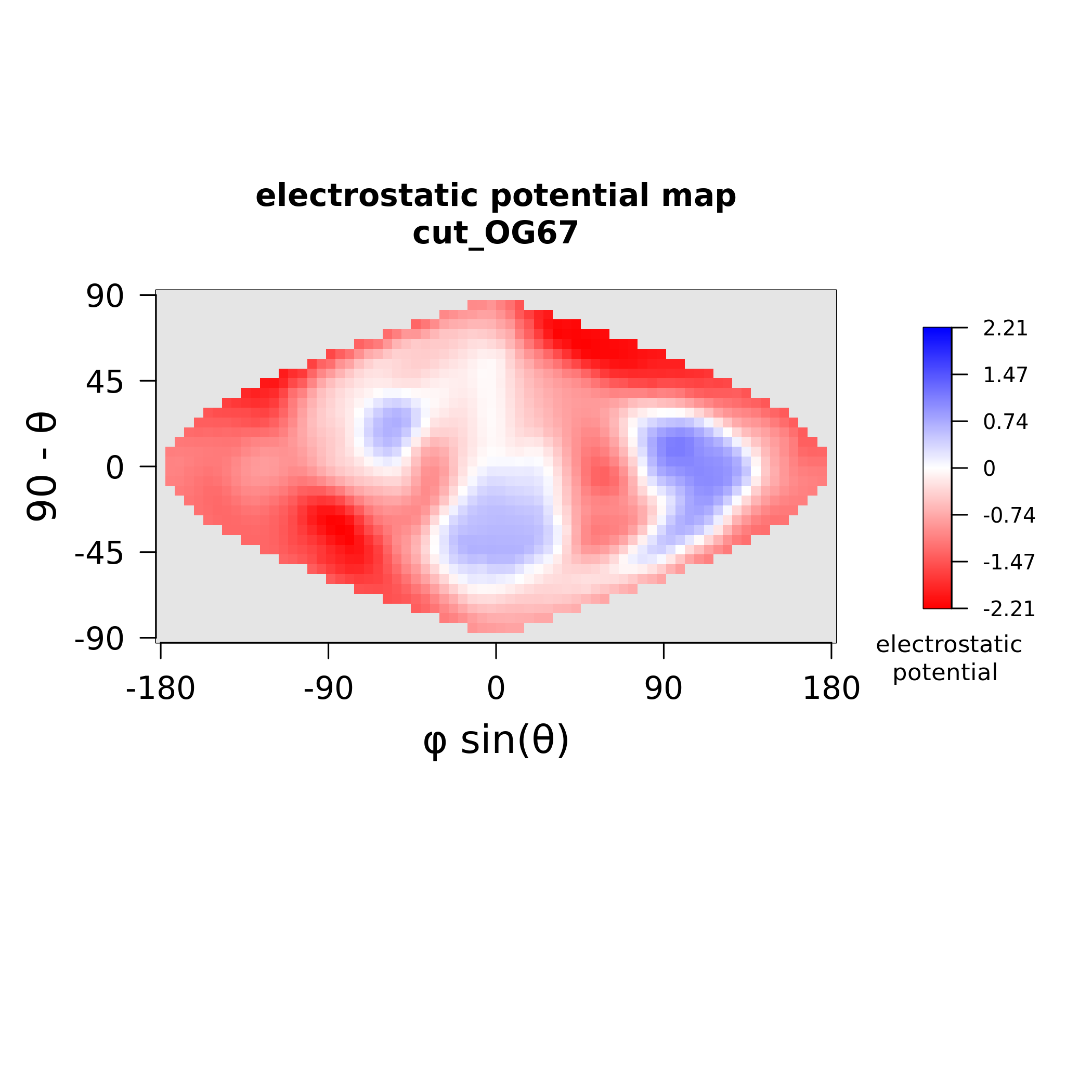

Supplement: S2 File — (ZIP) [file ppat.1012176.s019.zip › S2_File/ELECTROSTATICS/MAX67_electrostatics.png]

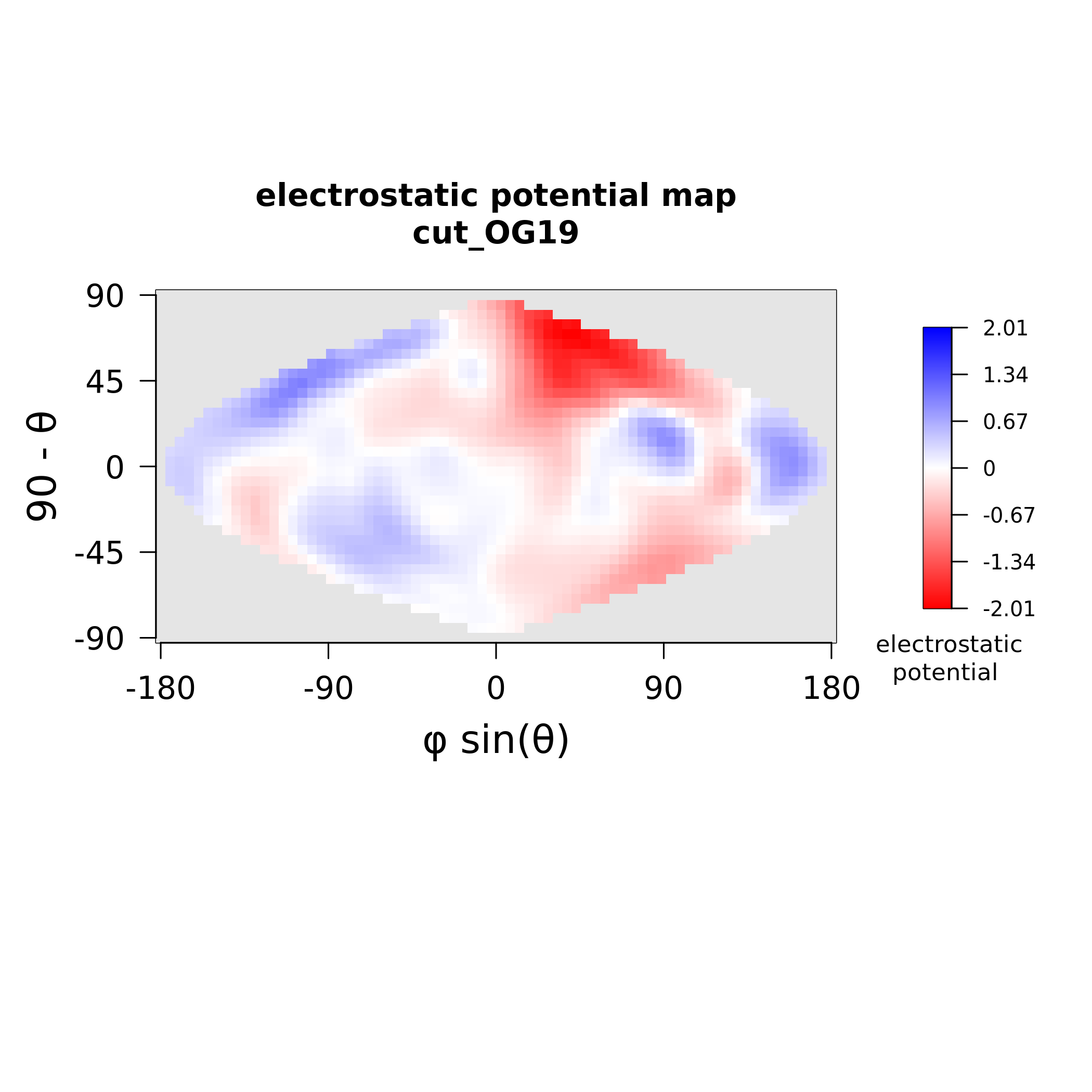

Supplement: S2 File — (ZIP) [file ppat.1012176.s019.zip › S2_File/ELECTROSTATICS/MAX19_electrostatics.png]

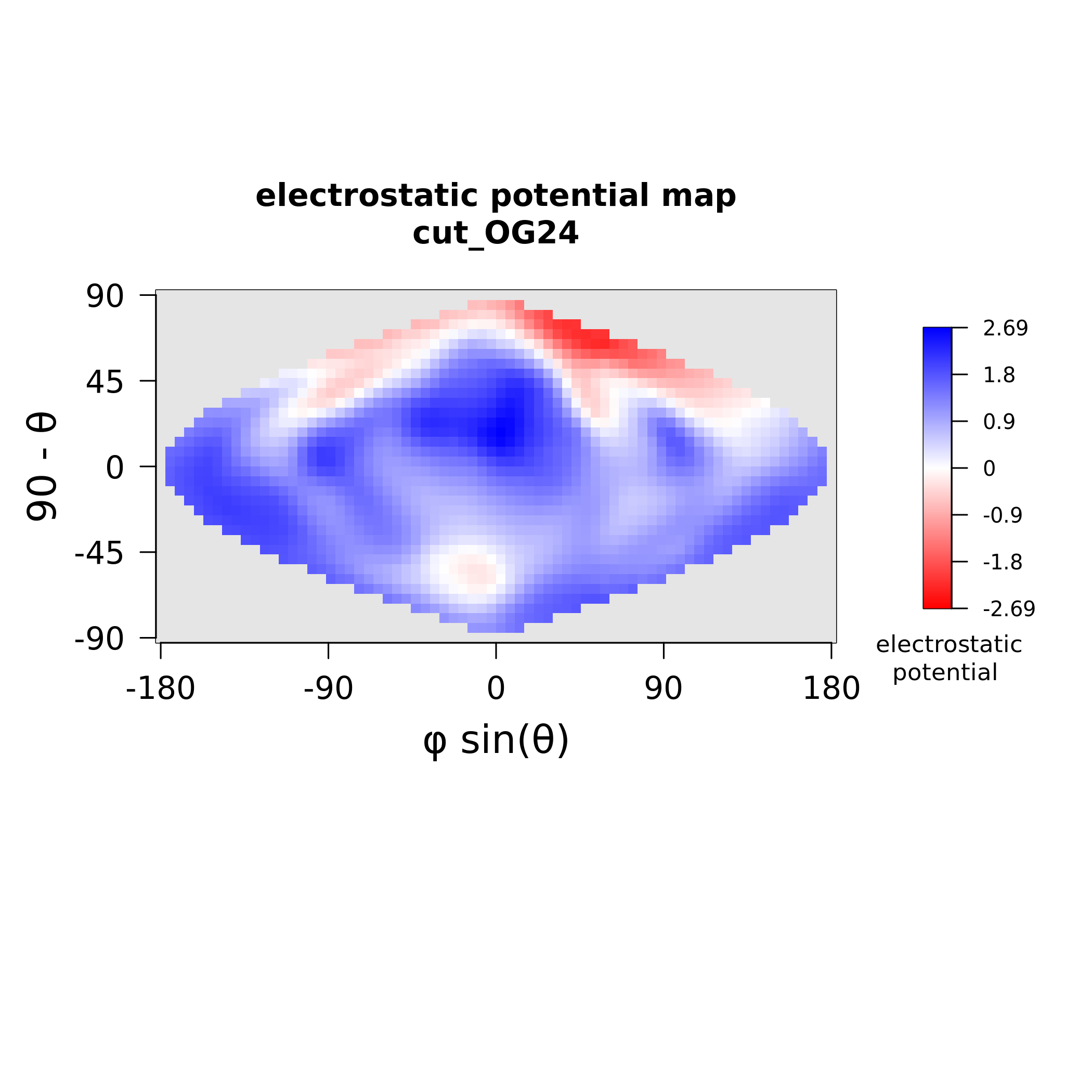

Supplement: S2 File — (ZIP) [file ppat.1012176.s019.zip › S2_File/ELECTROSTATICS/MAX24_electrostatics.png]

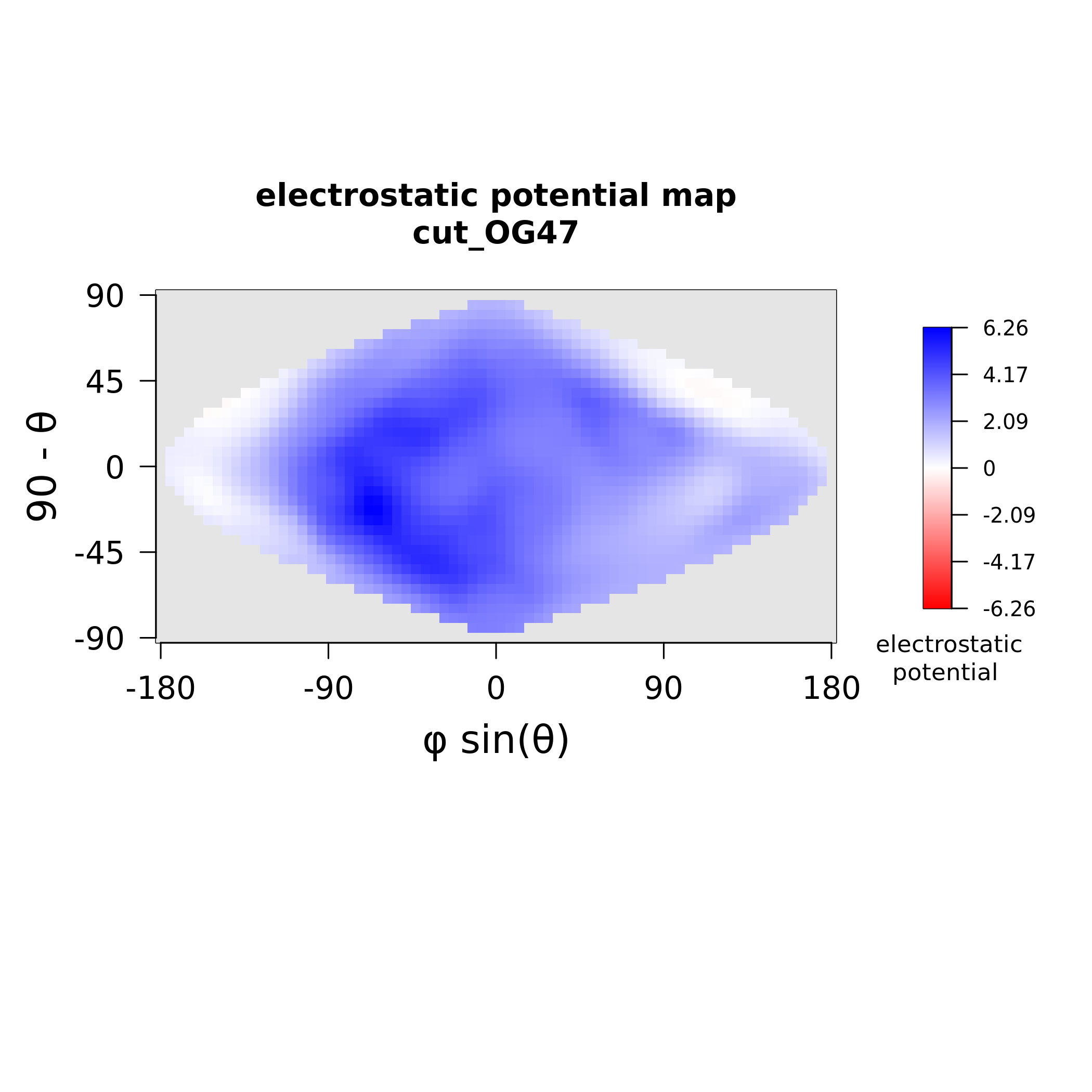

Supplement: S2 File — (ZIP) [file ppat.1012176.s019.zip › S2_File/ELECTROSTATICS/MAX47_electrostatics.png]

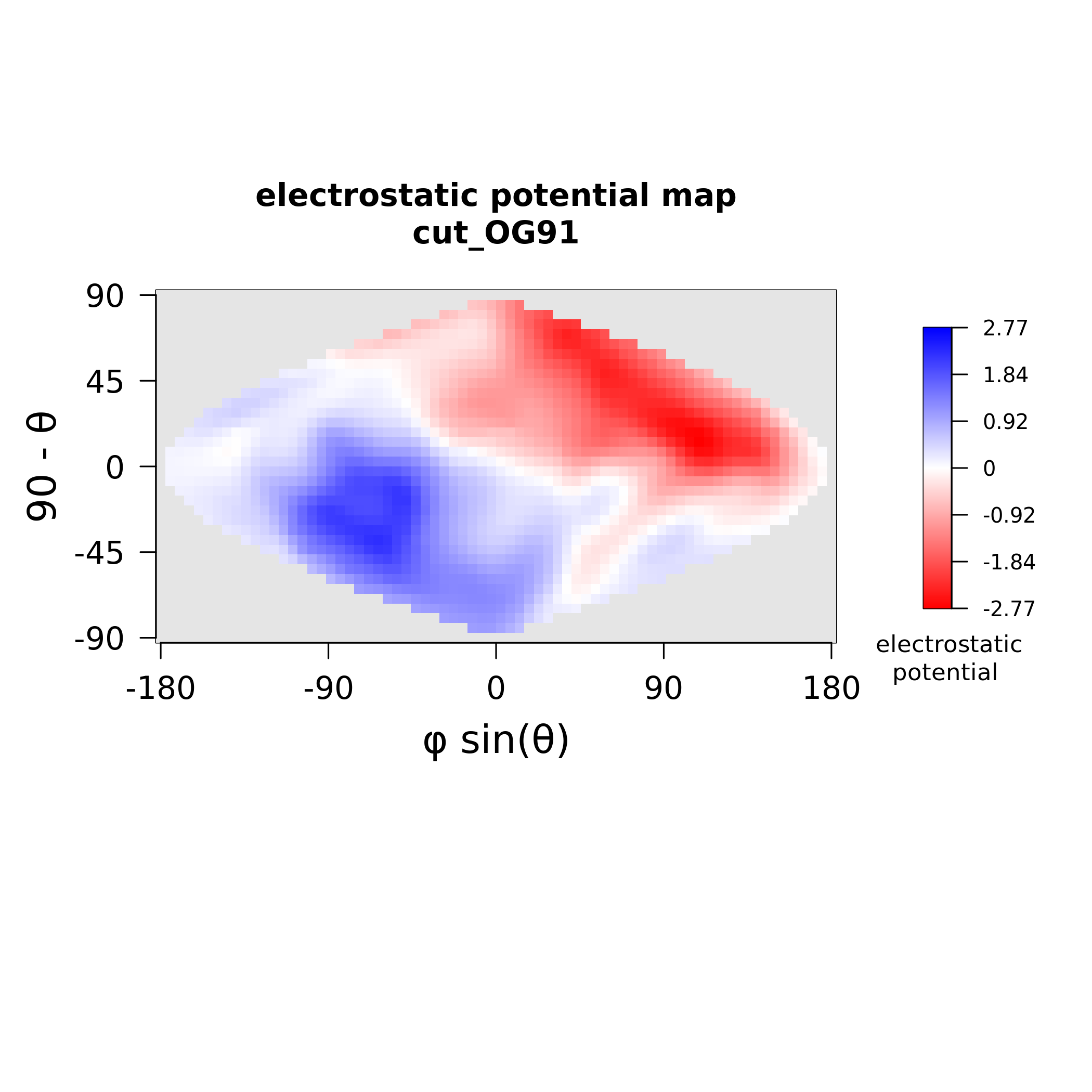

Supplement: S2 File — (ZIP) [file ppat.1012176.s019.zip › S2_File/ELECTROSTATICS/MAX91_electrostatics.png]

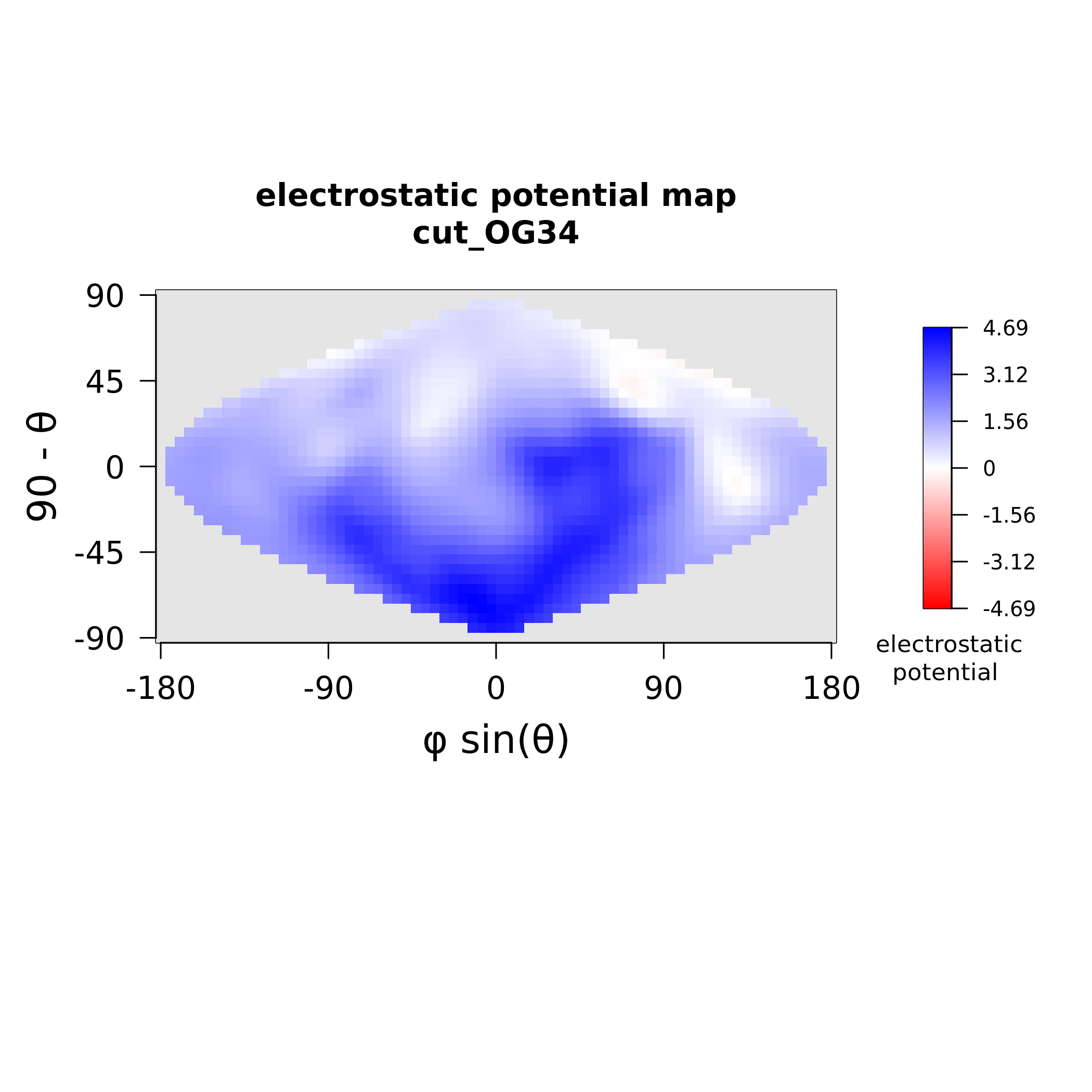

Supplement: S2 File — (ZIP) [file ppat.1012176.s019.zip › S2_File/ELECTROSTATICS/MAX34_electrostatics.png]

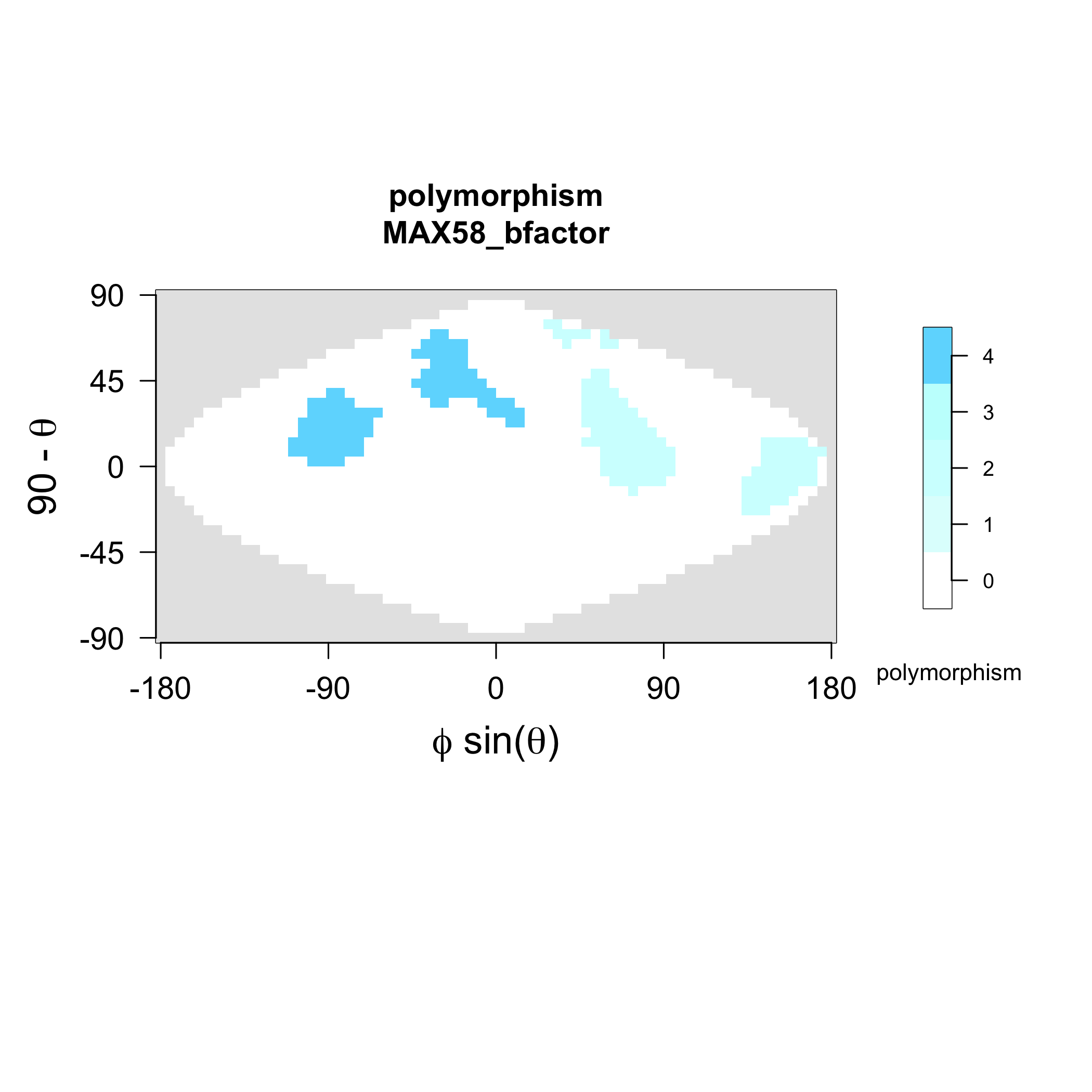

Supplement: S2 File — (ZIP) [file ppat.1012176.s019.zip › S2_File/POLYMORPHISM/MAX58_polymorphism.png]

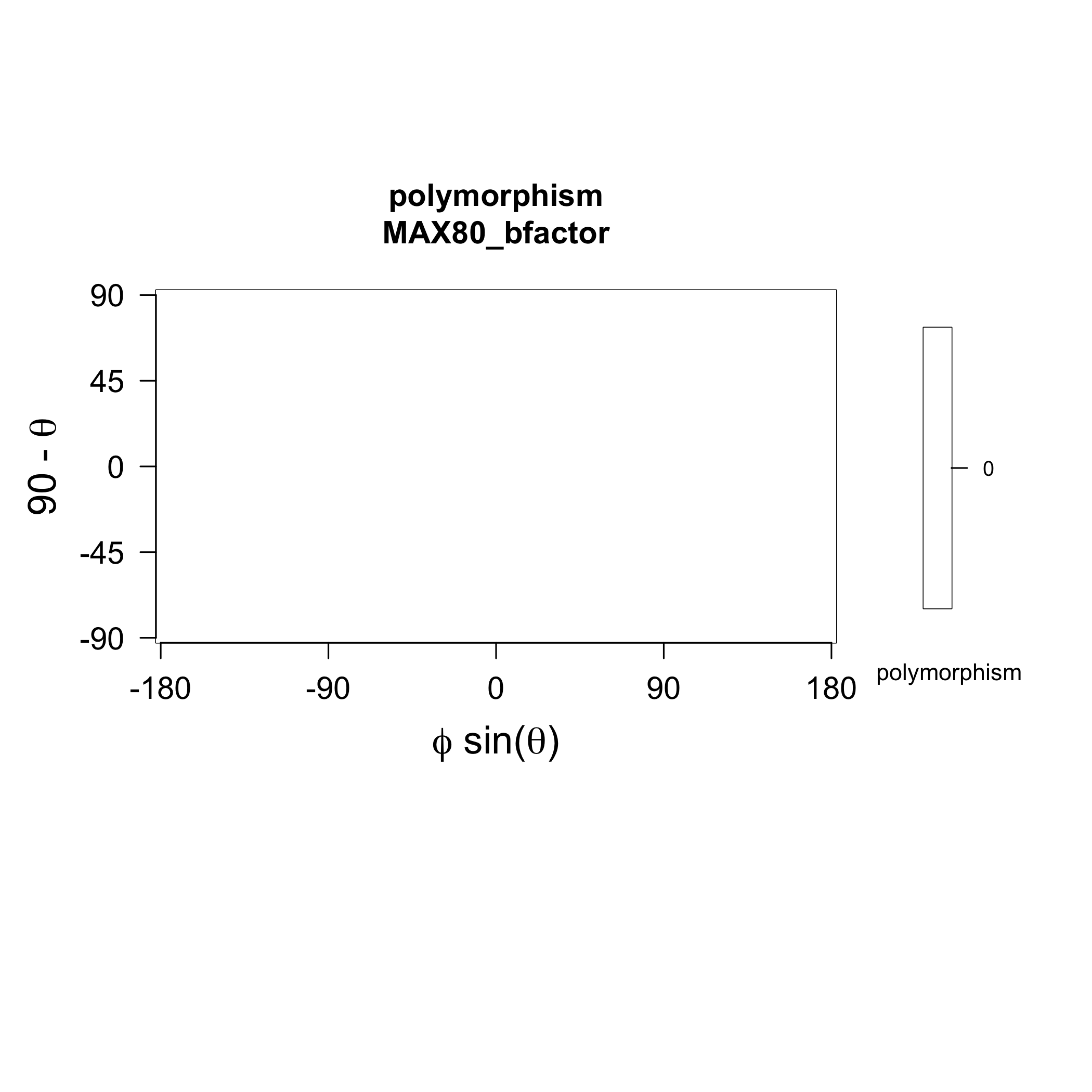

Supplement: S2 File — (ZIP) [file ppat.1012176.s019.zip › S2_File/POLYMORPHISM/MAX80_polymorphism.png]
